# Conformationally Controlled sp^3^‐Hydrocarbon‐Based α‐Helix Mimetics

**DOI:** 10.1002/anie.202301209

**Published:** 2023-05-02

**Authors:** Lydia I. Dewis, Madhavachary Rudrakshula, Christopher Williams, Elisabetta Chiarparin, Eddie L. Myers, Craig P. Butts, Varinder K. Aggarwal

**Affiliations:** ^1^ School of Chemistry University of Bristol Cantock's Close Bristol BS8 1TS UK; ^2^ Chemistry R&D Oncology AstraZeneca Cambridge CB4 0QA UK; ^3^ School of Biological and Chemical Sciences University of Galway University Road Galway Ireland

**Keywords:** Conformation Control, Organoboron, Protein-Protein Interactions, Syn-Pentane Interactions, α-Helix Mimetics

## Abstract

With over 60 % of protein–protein interfaces featuring an α‐helix, the use of α‐helix mimetics as inhibitors of these interactions is a prevalent therapeutic strategy. However, methods to control the conformation of mimetics, thus enabling maximum efficacy, can be restrictive. Alternatively, conformation can be controlled through the introduction of destabilizing *syn*‐pentane interactions. This tactic, which is often adopted by Nature, is not a common feature of lead optimization owing to the significant synthetic effort required. Through assembly‐line synthesis with NMR and computational analysis, we have shown that alternating *syn*–*anti* configured contiguously substituted hydrocarbons, by avoiding *syn*‐pentane interactions, adopt well‐defined conformations that present functional groups in an arrangement that mimics the α‐helix. The design of a p53 mimetic that binds to Mdm2 with moderate to good affinity, demonstrates the therapeutic promise of these scaffolds.

## Graphical abstract

Aberrant protein–protein interactions (PPIs) in disease can be disrupted through α‐helix mimetics. However, methods to control the conformation of mimetics for maximum efficacy can be restrictive. Here we show that destabilising *syn*‐pentane interactions allow substituted hydrocarbons to adopt well‐defined conformations to present functional groups in a way that mimics the α‐helix, thus allowing them to be used as scaffolds for inhibitor design.

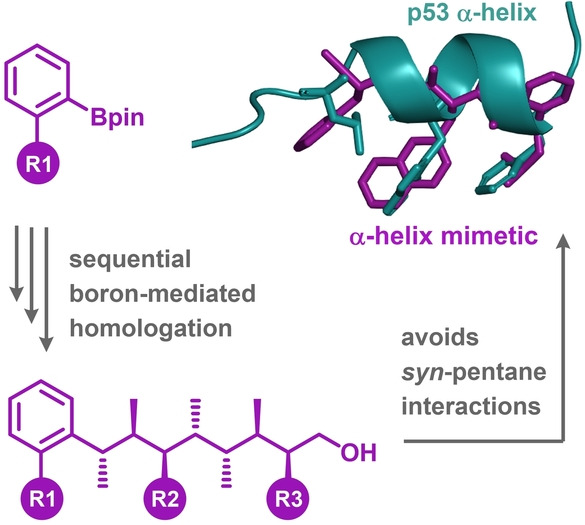

## Introduction

The prevalence of the α‐helix in protein secondary structure, and as recognition motifs in over 60 % of all protein–protein interactions (PPIs), makes them sought‐after targets in the design of protein mimetics and inhibitors.^[1]^ Good α‐helix mimetics have conformationally biased scaffolds that display an arrangement of functional groups with distance and angular relationships that match those of the biologically relevant amino‐acid side‐chains, thus ensuring strong binding.^[2]^

The design of short peptides that are conformationally constrained through hydrocarbon stapling, cyclisation, and the introduction of hydrogen‐bonding motifs has been successful.^[3]^ However, the therapeutic utility of peptides can be limited by metabolic instability.^[4]^ Peptoids, which are oligomers of N‐substituted glycines (oligo‐NSGs), show significantly increased metabolic stability, but they exhibit low conformational control that can be difficult to address.^[5]^ Interestingly, oligomers of N‐substituted alanine (oligo‐NSAs) display 300‐fold superior biological activity as mimics of the p53 α‐helix in the inhibition of the p53–Mdm2 PPI, a characteristic that originates from the adoption of a highly populated extended linear conformation, which avoids pseudo 1,3‐allylic strain involving adjacent methyl groups.^[6]^

Hamilton et al. investigated *ortho*‐substituted terphenyl derivatives as non‐peptidic α‐helix mimetics.^[2a]^ The phenyl groups adopt a staggered conformation to reduce steric interactions between the *ortho* substituents, which are thus projected in an arrangement with angular relationships and distances corresponding to the *i*, *i*+4 and *i*+7 residues of an α‐helix.^[7]^ Low conformational control and low solubility in aqueous solution have been addressed through the introduction of heteroatoms (terpyridines and imidazole‐phenyl‐thiazoles),^[8]^ motifs for intramolecular hydrogen‐bonding (trisbenzamides, the enaminones and benzoylurea oligomers)^[9]^ and covalent bridges.^[10]^

**Figure 1:**
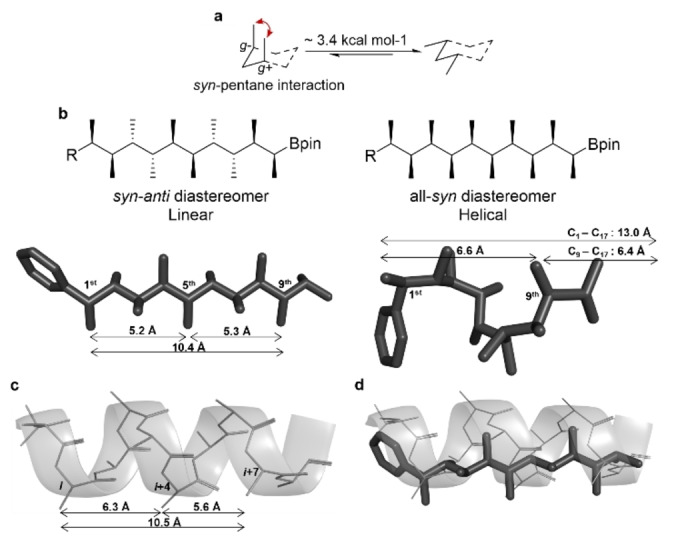
a) The *syn*‐pentane interaction. b) Contiguously‐methyl substituted *syn*–*anti* (**1**) and all‐*syn* (**2**) hydrocarbons. The distance between methyl groups on one face of the scaffolds closely match the distance between residues on one face of an α‐helix. c) An α‐helical polyalanine showing distances for *i*, *i*+4 and *i*+7 residues. d) An overlay of *syn*‐anti diastereomer **1** and α‐helical polyalanine showing overlap of the 1^st^, 5^th^ and 9^th^ methyl groups with the *i*, *i*+4 and *i*+7 residues of the α‐helix.

Reminiscent of the above‐described oligo‐NSAs, which are conformationally biased through the avoidance of 1,3‐allylic strain, we wondered whether the avoidance of the *syn*‐pentane interaction could be used to similar effect in the context of an sp^3^ hydrocarbon backbone. First reported by Still, the *syn*‐pentane interaction is established when a hydrocarbon chain adopts consecutive *g*+ (+60°) and *g*− (−60°) dihedral angles, thus bringing two backbone carbon units, four bonds away, within 2.5 Å of each other and incurring an energy penalty of 3.3–3.7 kcal mol^−1^ (Figure 1a).^[11]^ Still and Hoffmann demonstrated that the avoidance of *syn*‐pentane interactions could be used to control the conformation of acyclic carbon chains through the introduction of methyl substituents with a 1,3‐positional relationship along the chain.^[12]^ We have recently demonstrated that contiguous methyl substitution leads to even greater conformational control. For example, an all‐*syn* contiguously methyl‐substituted hydrocarbon avoids *syn*‐pentane interactions through adopting alternating *g*+/−, *t* (±60°, 180°) dihedral angles along the hydrocarbon backbone, resulting in a helical conformation; similarly, alternating *syn*–anti contiguously methyl‐substituted hydrocarbon adopts contiguous *t* (180°) dihedral angles, resulting in a linear zig–zag conformation (Figure 1b).^[13]^

**Figure 2:**
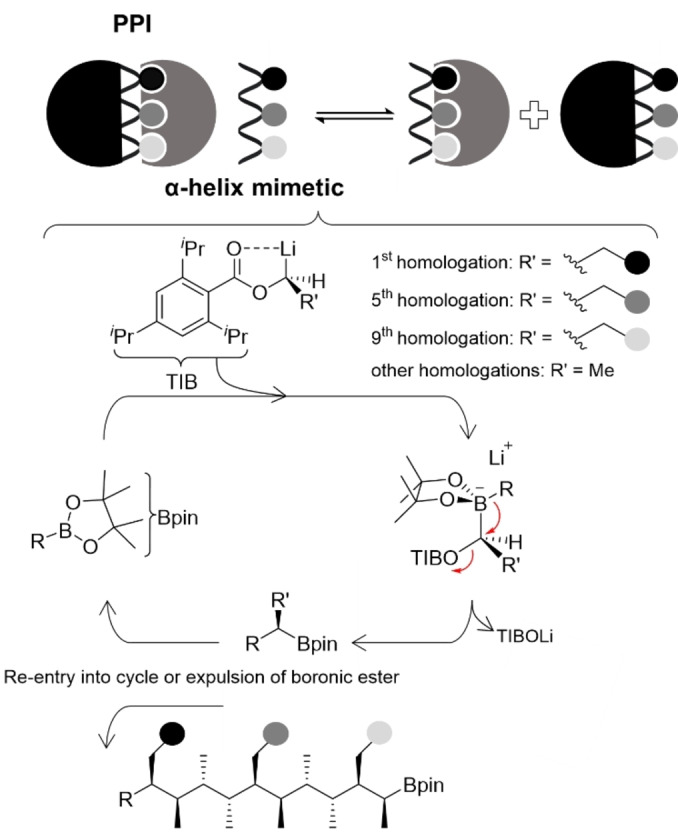
Design of an α‐helix mimetic PPI inhibitor based on linear hydrocarbon **1**. The shaded circles represent the hot‐spot residues identified for the PPI in question. The hot‐spot groups can be incorporated by using benzoate ester building blocks bearing the desired residue.

Examination of both the linear (alternating *syn*–anti, **1**) and helical (all‐*syn*, **2**) methyl‐substituted hydrocarbons revealed that the distances between methyl groups on one face of the scaffold mimic those between residues located at the *i*, *i*+4 and *i*+7 positions of an α‐helical peptide (5–7 Å, Figure 1c). For example, super‐imposing the linear scaffold (**1**) on a representative polyalanine α‐helix shows that the 1^st^, 5^th^ and 9^th^ positions of the linear scaffold closely match the distances and angular relationships of the *i*, *i*+4 and *i*+7 positions of the α‐helical polyalanine (Figure 1d). These contiguously methyl‐substituted hydrocarbons were prepared by iterative stereospecific homologation of boronic esters by using highly enantiomerically enriched α‐lithiated ethyl benzoate esters (R′=Me, Figure 2) as building blocks, which were generated in situ through Sn–Li exchange.^[14]^ To generate an α‐helix mimetic, one would simply need to replace the α‐lithiated ethyl benzoate at judicious positions in the synthetic sequence with derivatives bearing the desired side‐chains of the hot‐spot residues (R′ ≠ Me, Figure 2).

Herein, through synthesis, computation and biophysical studies, we show that linear hydrocarbon scaffold (**1**) can mimic the *i*, *i*+4 and *i*+7 positions of an α‐helix peptide. Our design was based on the mimicry of p53, which through a leucine residue, a tryptophan residue and a phenylalanine residue at the *i*, *i*+4 and *i*+7 positions, binds to a hydrophobic pocket in Mdm2.^[15]^

As misregulation of the p53–Mdm2 PPI is implicated in over 50 % of all human cancers, this interaction is the subject of a large number of studies, with the resulting wealth of knowledge making p53–Mdm2 a useful platform to showcase novel therapeutic design.^[16]^

## Results and Discussion

### Choice of Scaffold

First, we needed to decide which of our two hydrocarbon scaffolds—the linear alternating *syn*–*anti* isomer (**1**) or the helical all‐*syn* isomer (**2**)—present substituents in an arrangement that most closely matches that of the *i*, *i*+4 and *i*+7 residues of an α‐helix. The relative positions of the C_β_ atoms of the *i*, *i*+4 and *i*+7 residues of a representative all‐Ala α‐helix can be defined by the following distances: C_β(*i*)_–C_β(*i*+4)_, 6.3 Å; C_β(*i*)_–C_β(*i*+7)_, 10.5 Å; C_β(*i*+4)_–C_β(*i*+7)_, 5.6 Å (Figure 1c). The corresponding distances involving the carbon atoms of the methyl substituents at the 1^st^, 5^th^ and 9^th^ positions of the linear scaffold **1** were a close match with a maximum deviation of 16 % between two matching distances: C_methyl(1st)_–C_methyl(5th)_, 5.2 Å; C_methyl(1st)_–C_methyl(9th)_, 10.4 Å; C_methyl(5th)_–C_methyl(9th)_, 5.3 Å (Figure 1b). In comparison, the distances for the helical scaffold **2** were up to 25 % too long (C_methyl(1st)_–C_methyl(9th)_, 6.6 Å; C_methyl(1st)_–C_methyl(17th)_, 13.0 Å; C_methyl(9th)_–C_methyl(17th)_, 6.4 Å). Furthermore, the RMSD between the relevant C_β_ atoms of the linear scaffold **1** and those of the all‐Ala α‐helix was 0.91 Å whereas that for the same comparison involving the helical scaffold **2** was 1.27 Å. An overlay of the linear scaffold with the all‐Ala α‐helix also confirmed that the C_backbone_–C_methyl_ torsions (C_backbone_–C_methyl−1st_, −60.4°; C_backbone_–C_methyl−5th_, 56.5°; C_backbone_–C_methyl−9th_, 57.7°) closely matched those of the corresponding *χ*_1_ torsions (*χ*1 *i*, −55.8°; *χ*1 *i*+4, 55.2°; *χ*1 *i*+7, 53.7°) (Figure 1d). The linear scaffold also offers the considerable advantage that fewer homologations will be needed to replicate the *i*, *i*+4 and *i*+7 residues of an α‐helix, thus reducing the molecular weight, the number of rotatable bonds and length of the synthetic sequence.

### Computational Design of an α‐Helix Mimetic Scaffold

We then needed to confirm that replacing the methyl substituents at the 1^st^, 5^th^ and 9^th^ positions with larger substituents did not erode the conformational bias. A simple diamond lattice analysis of the *syn*‐*anti* isomer (**1**) show that high‐energy *syn*‐pentane interactions are only avoided when the molecule adopts the linear conformation, hence the high conformation control. However, the introduction of ethyl groups at the 1^st^, 5^th^ and 9^th^ positions, whilst maintaining the superimposition of all bonds on the idealized diamond lattice, necessarily introduces two *syn*‐pentane interactions, thus potentially leading to a loss of conformational control. However, we have recently shown through synthesis, computation and NMR analysis that *syn*‐pentane interactions are reduced by the 1,3‐positioned large substituents (acetoxyethyl groups) adopting an eclipsed conformation with respect to the main chain, whilst allowing the main chain to retain the linear conformation.^[17]^

**Figure 3:**
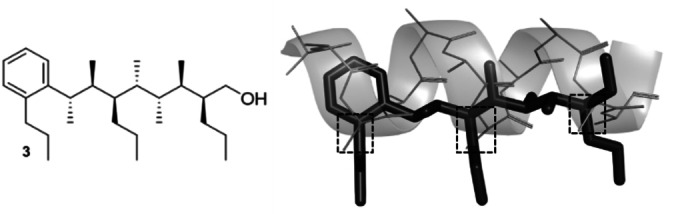
Overlay of scaffold **3** with a representative all‐Ala α‐helix. The *ortho* substituent, the 3^rd^ and the 7^th^ positions of the scaffold are shown to overlay well with the *i*, *i*+4 and *i*+7 residues of the α‐helix.

Molecular mechanics (MM) conformational search calculations employing the Monte Carlo multiple minimum (MCMM) search method, was used to confirm that extending the necessary side‐chains for mimicry of the *i*, *i*+4 and *i*+7 positions did not destroy the conformational bias of the backbone. Three propyl side‐chains were added onto the linear hydrocarbon scaffold, in place of a methyl group, and conformation assessed by MM conformational search. To minimise molecular weight, we considered incorporating a phenyl group at one terminus, thus allowing positioning of the *i* hotspot group mimetic at the associated *ortho* position rather than on the main sp^3^ backbone. Rotation of the phenyl group with respect to the hydrocarbon chain is restricted owing to *syn‐*pentane control, thus allowing the phenyl ring to adopt the orientation that places the side‐chain in the desired position. Additionally, we have also taken advantage of the reduced degrees of freedom of an sp^2^‐hybridised center over an sp^3^‐hybridised center whilst simultaneously reducing the number of homologations required. A conformational search of the resulting structure (**3**, Figure 3) revealed that 94 % of conformers adopted the linear form. An offset of ≈40° between the two groups that would have been eclipsed in a *syn*‐pentane interaction was observed. This distortion reduces the energy penalty incurred from the *syn*‐pentane interactions when adopting the linear conformation. Overlaying scaffold **3** with the representative all‐Ala α‐helix shows excellent mimicry, with an RMSD between the relevant C_β_ atoms of 1.19 Å (Figure 3).

**Figure 4:**
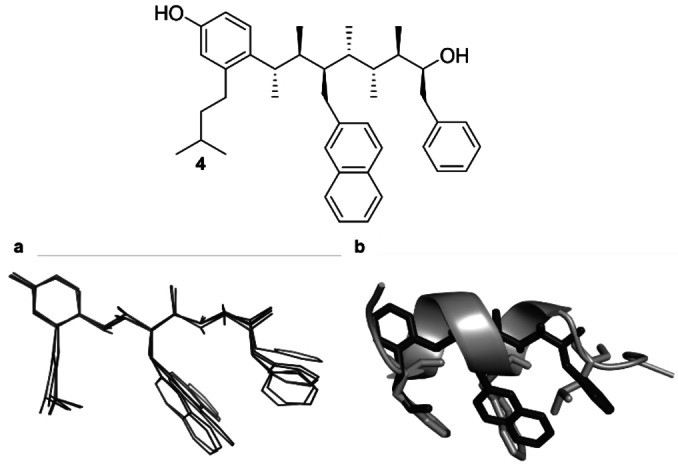
**a)** The five lowest‐energy conformers of mimetic **4** identified through an MM conformational search. **b)** An overlay of mimetic **4** with p53, representing the orientation predicted by molecular docking calculations (see below).

With confirmation that larger substituents can be accommodated without significantly perturbing the linear conformational bias, we turned our attention to the design of a p53 mimetic. Isopentyl, 2‐naphthylmethyl and benzyl groups, representing the leucine (*i*), tryptophan (*i*+4) and phenylalanine (*i*+7) hotspot residues of p53, were added to the *ortho* position of the phenyl terminus, the 3^rd^ sp^3^ carbon atom and the 7^th^ sp^3^ carbon atoms, respectively. Although the indole of Trp in p53 engages in a hydrogen bond with Leu54 on Mdm2, Hamilton et al. have reported that the interaction is not necessary for competitive inhibition of the p53–Mdm2 PPI and that a naphthyl group is a suitable mimetic of the indole group of Trp.^[7b]^ Phenolic and secondary alcohol OH groups were added at the termini to provide handles for functionalisation to improve aqueous solubility (**4**, Figure 4). An MM conformational search of **4** revealed that the addition of the hotspot groups did not significantly perturb the linear conformational bias, with over 77 % of conformers adopting a linear conformation. The RMSD between the C_β_ positions of the *i*, *i*+4 and *i*+7 residues of p53 and the relevant carbon atoms of the p53 mimetic was 0.864 Å, suggesting that target **4** was a structural mimetic of p53.

**Figure 5:**
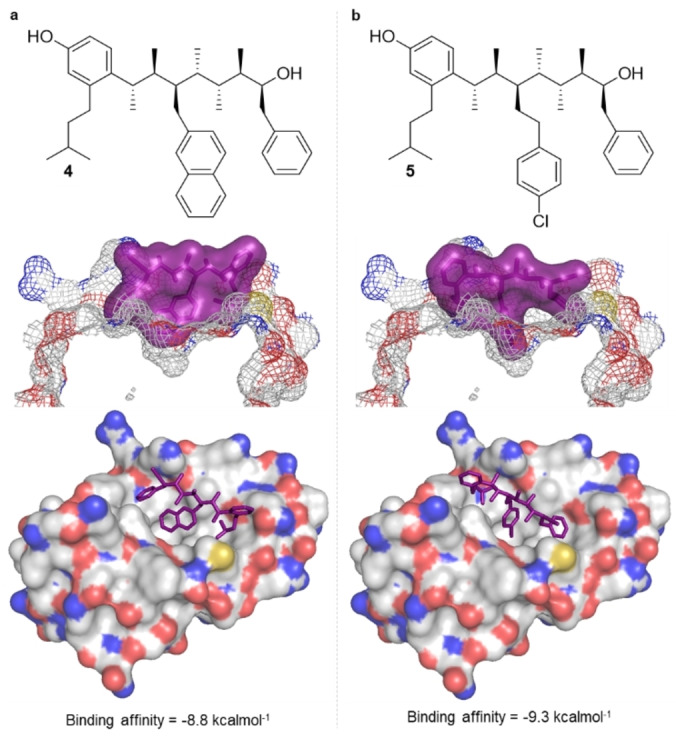
Molecular docking using AutoDock Vina. **a**) The predicted binding pose of mimetic **4** within the p53 pocket of Mdm2. **b**) The predicted binding pose of mimetic **5** within the p53 pocket of Mdm2.

Molecular docking, using AutoDock Vina, was used to confirm that the designed p53 mimetic binds to Mdm2 at the p53 binding site and that the preferred conformation for the mimetic was a linear conformation. The lowest‐energy conformer of mimetic **4**, as obtained from the MM conformational search, was docked into Mdm2, the structure of which was extracted from the crystal structure of the Mdm2–p53 complex (PDB:1YCR). All rotatable bonds within the molecule were allowed to rotate so as to sample different conformations of mimetic **4**. The highest‐affinity binding pose places the molecule in the desired p53 binding domain of Mdm2, but with the isopentyl, 2‐naphthylmethyl and benzyl groups occupying the pockets corresponding to the *i*+7, *i*+4 and *i* residues of p53, respectively (Figure 5a). The direction of the pose is opposite to what was expected (the benzyl group was binding to the Leu pocket). However, this sense of binding is consistent with that of other Mdm2 inhibitors, which incorporate an aromatic group designed to bind to the Leu pocket where it would engage in a favorable π–π interaction with a His residue.^[15e]^ The binding affinity of the designed p53 mimetic **4** was calculated as −8.8 kcal mol^−1^.

Competitive inhibition of the p53–Mdm2 PPI has been found to be more effective when using a mimetic bearing a chlorine group that can occupy a deep void in the Trp23 pocket of Mdm2.^[18]^ We investigated this design by replacing the 2‐naphthylmethyl group with a 4‐chlorophenylethyl group. The resulting structure **5**, which retains a linear conformational bias, binds to Mdm2 in a pose that not only places the chlorine atom in the desired deep void, but also places the isopentyl and benzyl groups in the expected *i* and *i+7* pockets, resulting in a higher binding affinity of −9.3 kcal mol^−1^ (Figure 5b).

### Synthesis of p53 Mimetics

**Scheme 1:**
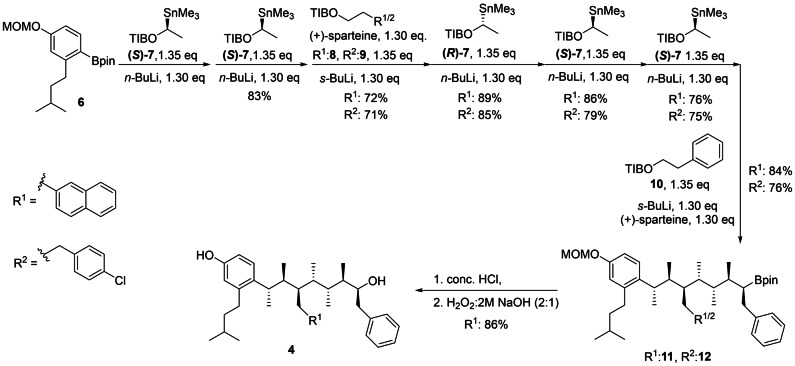
Assembly line synthesis of boronic ester derivatives **11** and **12**, which were converted into p53 mimetics **4** and **5**, respectively. MOM=methyoxymethyl; TIB=2,4,6‐triisopropylbenzoate; pin=pinacol.

The p53 mimetics, **4** and **5**, were prepared by using assembly line synthesis starting from a methoxymethyl‐protected aryl boronic ester bearing an isopentyl group at the *ortho* position (**6**, see the Supporting Information for details).^[13]^ Sequential homologation of boronic ester **6** was initiated by adding the boronic ester to a −78 °C ethereal solution of enantiopure lithium carbenoid, which was generated in situ through enantiospecific tin–lithium exchange of enantiopure α‐stannyl benzoate (*S*)‐**7**. Stannane **7** is a crystalline solid that can be prepared in >99.9:0.1 e.r., a level of enrichment that can be achieved through several rounds of recrystallisation.^[13]^ Notably, we have recently shown that these stannanes can be substituted for the corresponding sulfoxides, which are less toxic more regulatory friendly building blocks.^[19]^ Warming the solution of the resulting boronate to room temperature, effected stereospecific 1,2‐metallate rearrangement with elimination of benzoate to give the enantiopure one‐carbon extended boronic ester. The process was repeated until all seven substituted carbon atoms were incorporated (Scheme 1). For the third and seventh methine positions, which bear the 2‐naphthylmethyl (or 4‐chlorophenylethyl) and benzyl substituents, respectively, the requisite lithium carbenoid was generated through (+)‐sparteine‐mediated enantioselective deprotonation of the corresponding benzoate rather than by tin‐lithium exchange. Here, the corresponding α‐stannyl benzoates, which were generated through sparteine‐mediated deprotonation and stannylation, were oils rather than crystalline solids. Therefore, because further enantioenrichment through recrystallisation was not possible, the sparteine‐ligated lithium carbenoids, which were generated in situ with high levels of enantiopurity (>97 : 3), were used directly. Intermediate boronic esters were purified by column chromatography in high yields (>70 %) after each homologation, to remove excess building blocks. The overall yield of the lithiation–borylation sequence was 27 % for target **11** (the boronic ester derivative of mimetic **4**) and 24 % for target **12** (the boronic ester derivative of mimetic **5**). The boronic ester **11** was subjected to aqueous acid followed by basic hydrogen peroxide to reveal the phenolic and secondary OH groups of p53 mimetic **4**, which could then be subjected to NMR conformational analysis.

**Scheme 2:**
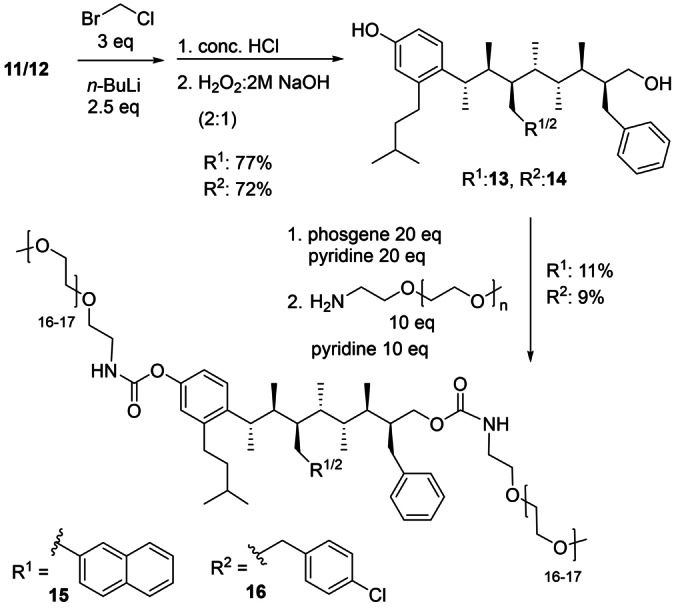
Synthesis of PEGylated derivatives for improved aqueous solubility.

To increase solubility for biophysical analysis, boronic esters **11** and **12** were subjected to further derivatisation. First, Matteson homologation of the boronic esters introduced an additional methylene spacer to reduce the likelihood of the solubilising groups interfering with the binding of the benzyl group.^[20]^ The spacer also facilitates derivatisation because it ultimately leads to a primary alcohol rather than a more sterically hindered secondary alcohol. Subjecting the resulting primary boronic esters to aqueous acid followed by basic hydrogen peroxide revealed the phenolic and primary OH groups of mimetics **13** and **14**. Polyethylene glycol (PEG) chains, which are routinely used in the pharmaceutical industry to improve aqueous solubility, were introduced through carbamoylation of the terminal OH groups by using phosgene and the methoxypolyethylene glycol amine (average *M*_w_=750).^[21]^ The resulting PEG derivatives **15** and **16** were purified by RP‐HPLC (Scheme 2).

**Scheme 3:**
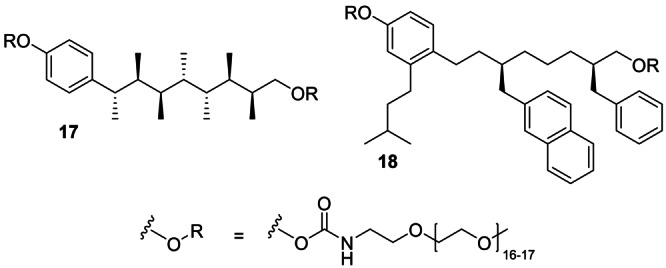
Control molecules **17** and **18**.

Using similar protocols, two control molecules were synthesised, both of which are expected to bind weakly to Mdm2 (Scheme 3). Control molecule **17**, the parent contiguously methyl‐substituted hydrocarbon, would be used to qualitatively assess the contribution of the hotspot groups to the affinity of the mimetic for Mdm2. Control molecule **18** contains the isopentyl, 2‐naphthylmethyl and benzyl hotspot groups but separated by simple methylene spacers, which would lead to poor conformational control, as confirmed by MM conformational analysis. Therefore, control molecule **18** would be used to qualitatively assess the contribution of conformational control to the affinity of the mimetic for Mdm2.

### NMR and Computational Conformational Analysis

Although MM conformational searching indicated that the designed p53 mimetics should preferentially adopt the linear conformation that is conducive for strong binding to Mdm2, we needed experimental evidence to support the conformational bias in solution. Such evidence could be provided by comparing experimentally measured NMR properties, including ^*n*^ *J*_HH_ and ^*n*^ *J*_CH_ scalar coupling constants, ^1^H and ^13^C chemical shifts and interproton distances derived from 1D‐NOESY experiments, to those obtained from DFT calculations on the calculated distribution of conformers.^[22]^ A strong correlation between the experimental and calculated values would provide a high level of confidence that the calculated conformational ensemble accurately represents the solution state conformational ensemble. Our comparative analysis was based on hydrocarbon **4** because NMR analysis revealed that among all other derivatives, it exhibits the lowest level of peak broadening and all backbone CH ^1^H resonances are either fully or partially resolved enabling analysis by selective NMR experiments. Specifically, NMR conformational analysis of a solution of hydrocarbon **4** (CDCl_3_, conc. 21 mM) was performed on a Bruker 500 MHz NMR spectrometer fitted with a 5 mm DCH ^13^C‐^1^H/D cryoprobe at 21 °C. The large PEG derivatives were unsuitable owing to the unacceptable level of computational resource that would be required and the slow molecular tumbling, which lead to significant broadening of resonances in the ^1^H NMR spectrum. Furthermore, as the molecular size and rotational correlation time increases, NOE intensities decrease to zero and become negative.

We first examined the ^3^ *J*_HH_ values for hydrocarbon **4** owing to their relationship with dihedral angle, as described by the Karplus equation.^[23]^ In the linear conformation, the backbone HCCH dihedral angles alternate between 180° and 60°, i.e. *anti* and *gauche*, giving rise to alternating large (>8 Hz) and small (<4 Hz) ^3^ *J*_HH_ values, respectively. Although the ^1^H resonances in hydrocarbon **4** are well dispersed, the multiplets were broadened by fast *T*_2_ relaxation. Consequently, full spin simulations of all couplings for all signals in the spectrum was used to extract experimental coupling constants by modelling the multiplet shapes based on estimated coupling constants, linewidths and the exact chemical shift (the latter obtained from pureshift ^1^H spectra). Once the simulated peak shapes for the multiplets match the experimental spectra, the estimated coupling constants can be considered accurately defined.

The experimental values of ^3^ *J*_HH_ measured from spin simulation were compared to those calculated by DFT methods. An initial geometry optimisation and frequency calculation, using the mPW1PW91 functional and the 6‐31G(d) basis set, was performed on a subset of conformers from the initial MM conformational search. The calculations were all performed in chloroform by using the IEFPCM solvation model. The optimized geometries were subjected to a single point energy and frequency calculation using mPW1PW91/6‐311G(d,p) with the resulting Gibbs energies being used to calculate the Boltzmann populations of conformers. NMR scalar coupling constants and magnetic shielding tensors (MSTs) were calculated for 99 % of those conformers (based on their Boltzmann populations) using the GIAO method with mPW1PW91/6‐311G(d,p). All calculated NMR parameters were Boltzmann averaged according to their calculated Boltzmann populations.

**Figure 6:**
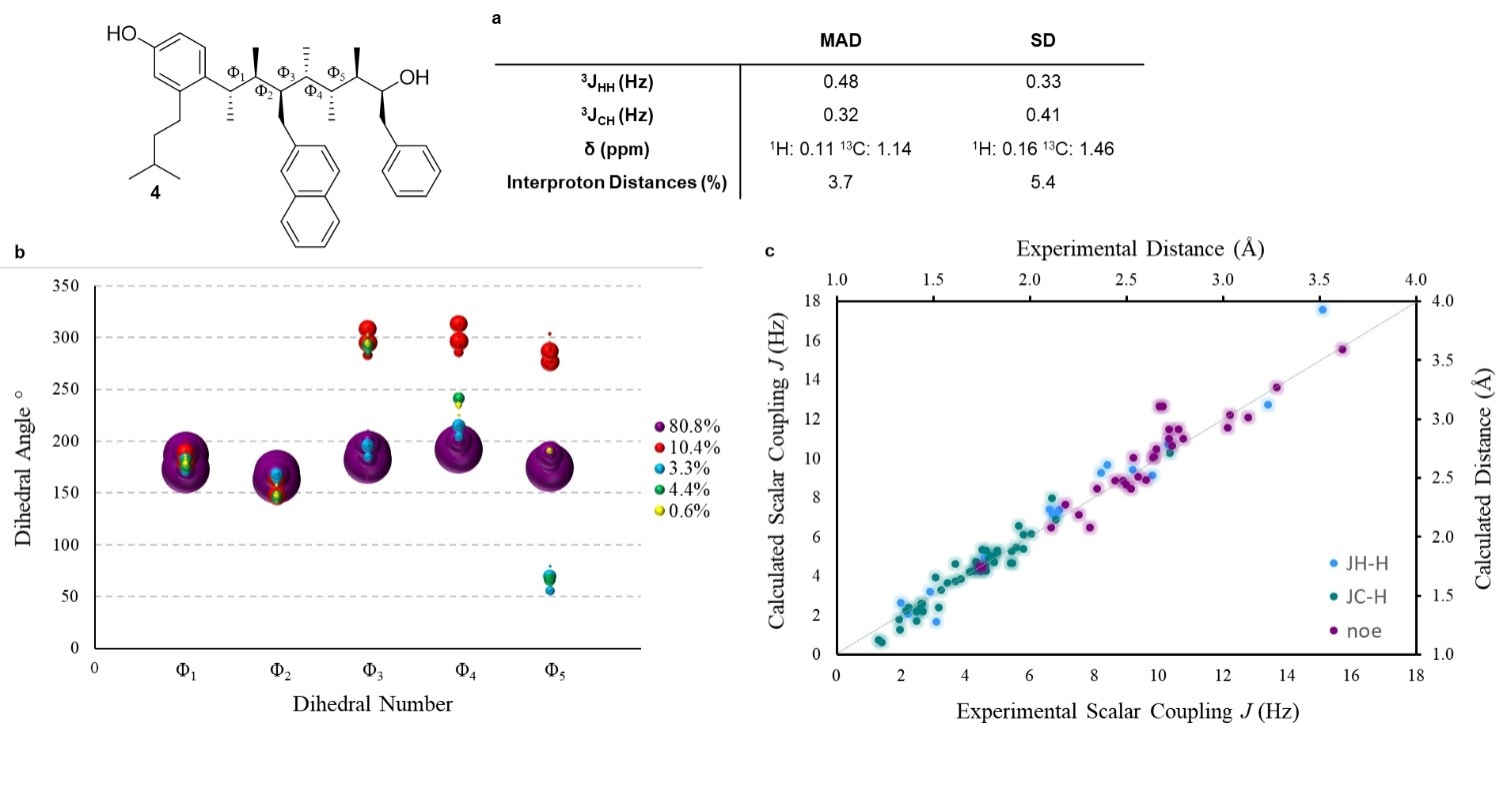
Conformational analysis of mimetic **4**. (CDCl_3_. 21 mM) **a)** MADs and SDs from comparison of experimental and calculated NMR parameters. **b)** A ‘bubble plot’ showing the major clusters of conformers as determined by DFT. The major cluster corresponds to a linear conformation, which exhibits dihedral angles of 180° along the backbone. The size of each ‘bubble’ represents the Boltzmann population of that conformer. **c)** A graph showing the correlation between experimental interproton distances/scalar coupling constants and those calculated by DFT.

The experimental ^3^ *J*_HH_ values alternate between large and small, consistent with alternating *anti* and *gauche* dihedral angles, with mean absolute deviation (MAD) of 0.48 Hz and standard deviation (SD) of 0.33 Hz between the experimental and calculated values. The MAD values for hydrocarbon **4** are similar to those observed previously for molecules of similar structural complexity and flexibility.^[13]^ ^3^ *J*_CH_ values, which have the same dihedral angle dependency as ^3^ *J*_HH_ values, were also measured experimentally and compared to the DFT‐calculated values. ^3^ *J*_CH_ values were measured by using IPAP–HSQMBC, which, as reported by Dickson et al., is the most appropriate method when a global analysis of ^3^ *J*_CH_ values is required.^[24]^ Again an excellent correlation between experimental and DFT‐calculated ^3^ *J*_CH_ values was observed (MAD=0.32 Hz, SD=0.41 Hz, Figure 6). The accuracy and validity of this hybrid NMR‐computation approach for analysing molecular conformation can be increased by incorporating more NMR parameters into the analysis. Therefore, we also compared experimental ^1^H and ^13^C chemical shifts, which were measured directly from 1D spectra, to those derived from DFT MSTs. Again, an excellent correlation between experiment and computation was observed (^1^H: MAD=0.11 ppm, SD=0.16 ppm; ^13^C: MAD=1.14 ppm, SD=1.46 ppm, Figure 6).

**Figure 7:**
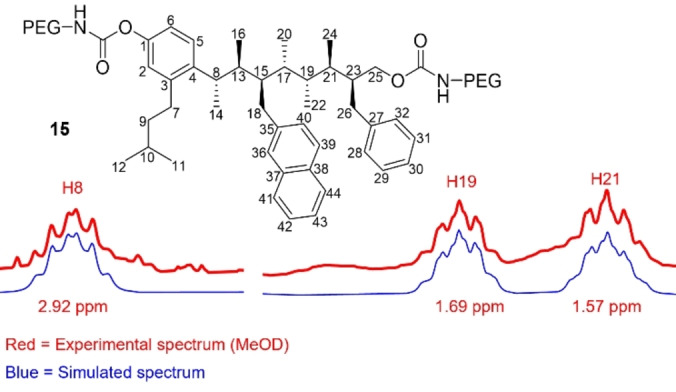
Simulated and experimental (MeOD, 500 MHz) ^1^H NMR backbone methine peaks of PEG derivative **15** showing that PEGylation does not disrupt backbone conformation, which remains linear.

The above experimental NMR parameters, which correlate well to those calculated by DFT, are consistent with a preferred linear conformation in solution. However, these NMR parameters are not sensitive to low levels of high‐energy conformations. Interproton distances, derived from NOESY experiments, is much more discriminating and has been used to identify conformers with populations as low as 2 %.[^25^, ^26^] Interproton distances were derived from 1D‐NOE and CSSF–NOE measurements and analysed using the PANIC method, as described by Macura and Krishnamurthy.^[27]^ The PANIC method corrects for different forms of cross relaxation by standardisation of the irradiated peak, thus allowing NOE intensities obtained from separate experiments to be grouped. A total of 29 NOE‐derived interproton distances were obtained from selective NOE experiments and compared to those calculated by DFT. Again, an excellent correlation between experimental and calculated values was observed (MAD=3.7 % SD=5.4 %, Figure 6) further confirming that the linear conformations identified by computation are also those that are highly populated in solution. Examination of ^1^H spectra of PEG derivatives **15** and **16** revealed well dispersed peaks that closely matched the peak shapes of their alcohol derivatives, suggesting that derivatization does not perturb the conformational landscape of the main scaffold. Additionally, a small number of ^3^ *J*_HH_ values associated with backbone dihedral angles were successfully simulated and all showed the expected magnitude (i.e. large for *anti* dihedral angles or small for *gauche* dihedral angles, Figure 7).

### ^1^H‐^15^N HSQC Analysis of Mimetic Binding

With conformationally controlled p53 mimetics in hand, their binding to Mdm2 was explored by using NMR spectroscopy. Protein‐ligand binding is commonly explored by using 2D ^1^H–^15^N HSQC spectroscopy owing to the high sensitivity of amide resonances to molecular environment. The binding of a ligand to a receptor leads to chemical shift perturbations (CSPs) of both amino acid residues that interact with the ligand and regions of the protein that undergo a conformational change.^[28]^ A ^1^H–^15^N TROSY spectrum of ^15^N‐Mdm2 was acquired on a 700 MHz spectrometer fitted with a 1.7 mm inverse triple‐resonance micro‐cryo probe. The protein was solubilised at a concentration of 50 μM in 20 mM tris(hydroxymethyl)aminomethane (Tris), 250 mM NaCl and 1 mM tris(2‐carboxyethyl)phosphine (TCEP) at pH 7.4. NMR studies have shown that Mdm2, which is prone to aggregation, is stable for up to one week when prepared under these conditions. Previously reported assignments of Mdm2 (Biological Magnetic Resonance Bank (BMRB) ID: 2410^[29]^ and 15945^[30]^) were used to partially assign the NH amide backbone peaks. For solubility, p53 mimetics were added as solutions in acetone‐*d*_6_ leading to 9 : 1 D_2_O/acetone solvent mixtures. A control ^1^H–^15^N TROSY experiment of Mdm2 with 10 % acetone‐*d*_6_ revealed that the presence of acetone did not compromise the stability of Mdm2 or induce any CSPs.

**Figure 8:**
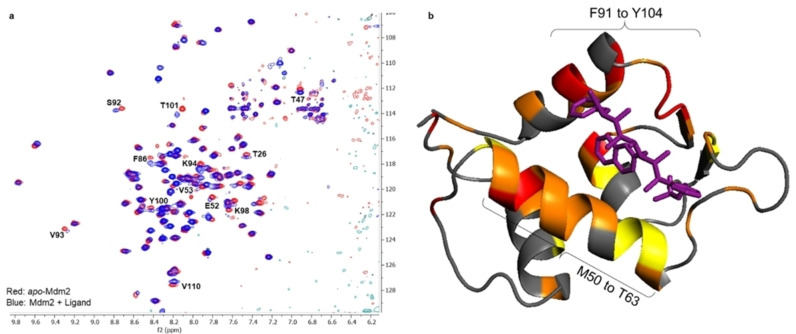
**a)** The ^1^H‐^15^N TROSY spectra of *apo*‐Mdm2 (red) and Mdm2+mimetic **15** (blue). Residues that exhibited a CSP>2σ upon binding of mimetic **15** have been marked. **b)** Mapping the location of binding of mimetic **15** to Mdm2 from the CSPs observed in the ^1^H–^15^N TROSY. Residues that induce a CSP>2σ are shown in red, CSP>σ are shown in orange and CSP<σ are shown in yellow.

A ^1^H–^15^N TROSY spectrum of a solution of Mdm2 (50 μM) and p53 mimetic **15** (70 μM) revealed CSPs for residues primarily located in or on the periphery of the Mdm2 binding pocket. CSPs associated with a small number of residues located sporadically at other regions of Mdm2 also were observed and were attributed to slight conformational changes of Mdm2 upon binding of p53 mimetic **15**. The CSPs were calculated as a Euclidean distance and all CSPs greater than twice the standard deviation (σ) of all measured CSPs were considered significant in binding.^[31]^ The largest chemical shift changes were observed in the Leu26 pocket (residues associated with p53 ligand and Mdm2 protein are labelled with the three‐letter and single‐letter amino acid codes, respectively), with all three residues (Y100, T101 and V53) that define the Leu26 pocket exhibiting a CSP>2σ. CSPs>2σ were also observed for V93 and S92, which are located in the Phe19 pocket. Of the remaining residues that exhibit a CSP>2σ, F86, K98, K94, T26 and E52 are located on the periphery of the binding pocket and Y110 and T47 are located in other regions; however, the latter residues have previously been reported to shift upon binding of a small molecule inhibitor to Mdm2 and are attributed to conformational changes to Mdm2 upon inhibitor binding (Figure 8a).^[32]^ By mapping the CSPs onto the surface of Mdm2 it is clear that p53 mimetic **15** occupies the p53 binding pocket, with the most perturbed regions of Mdm2 being an α‐helical domain that forms the side of the Phe19 pocket (F91–Y104), and another α‐helical domain that extends the length of the p53 binding pocket (M50‐T63) (Figure 8b).

^1^H–^15^N HSQC spectroscopy was also used to estimate the dissociation constant for the **15**–Mdm2 complex. The CSPs were tracked as p53 mimetic **15** was titrated into the solution of Mdm2 allowing a *K*_d_ to be obtained from the resulting binding curves, which were generated by using Mbinding from MestreNova. In this experiment, ^1^H–^15^N SOFAST HMQC, which employs short inter‐scan delays, was used to reduce experimental time. A total of six titration points were obtained and an average *K*_d_ was calculated based on the CSPs of twenty amino acids. For p53 mimetic **15**, an average *K*_d_ of 17.2±4.5 μM was obtained, suggesting that it binds to Mdm2 with moderate affinity. Limitations in ligand solubility prevented data from ligand concentrations >350 μM being gathered. Because saturation of Mdm2 was likely not achieved, the predicted *δ*_max_ and *K*_d_ should be used with care.

By using similar experimental protocols and analysis, the average dissociation constant for the **16**–Mdm2 complex was measured as 8.8±3.1 μM, which, within error, has similar affinity to that of p53 mimetic **15**. However, again saturation was not reached due to limited solubility and the predicted *K*_d_ should be used with care. The ^1^H–^15^N HSQC spectra revealed that the binding of p53 mimetic **16** gave rise to a similar set of significant CSPs, suggesting that the mimetics bind to the same pocket. The significance of the chlorine atom was confirmed by examining the L85, G83 and L82 residues, which are located deep in the Trp23 pocket. All three amino acid residues show more significant CSPs upon binding of p53 mimetic **16** to Mdm2 compared to the binding of p53 mimetic **15**, suggesting that the 4‐chloro‐phenylethyl substituent is a better fit for the Trp23 pocket.

Finally, the binding of control molecules **17** and **18** (Scheme 3) was explored using the same method. Both molecules did not induce significant CSPs suggesting that they bind to Mdm2 with weak affinity. The lack of significant CSPs and the fact that saturation of Mdm2 was not achieved during titration experiments prevented the measurement of accurate dissociation constants for the control measurements. The poor CSPs induced by the control molecules confirm the contribution of both the presence of hotspot groups and conformational preorganisation to the binding affinity.

## Conclusion

We have presented a new class of conformationally controlled sp^3^‐hydrocarbon‐based α‐helix mimetics, controlled through the avoidance of destabilising *syn*‐pentane interactions. Through the judicious placement of methyl groups along a flexible acyclic carbon chain, it is possible to bias the molecule to adopt a well‐defined conformation. When the methyl groups exhibit an alternating *syn*‐*anti* stereochemical relationship, the hydrocarbon chain adopts a linear (zig–zag) conformation. These scaffolds are suitable α‐helix mimetics as the distance and orientation between side chains at the 1^st^, 5^th^ and 9^th^ positions closely match those between the *i*, *i*+4 and *i*+7 residues of an α‐helical peptide. Using iterative lithiation‐borylation reactions it is possible to extend the hydrocarbon chain one carbon at a time, with full stereochemical control, by using the requisite substituted carbenoid building block. This method enables full flexibility over the side chains incorporated at the required positions for hot‐spot mimicry.

To demonstrate the possible therapeutic relevance of these scaffolds, we have designed and validated a scaffold that presents substituents in a pattern that corresponds to that formed by the *i*, *i*+4 and *i*+7 residues of the Mdm2 binding domain of p53. We have incorporated the specific hot‐spot residues of p53 on a hydrocarbon chain that adopts a linear conformation, and found that the distance and orientation of the hot‐spot residues correspond closely to that of the α‐helical p53 peptide, as confirmed through NMR and computational analysis. Our p53 mimetics bind to Mdm2 with moderate affinity (*K*_d_=8.8–17.2 μM) when compared to that of wild‐type p53 (*K*_d_=0.09–0.1 μM) and the known small‐molecule inhibitor, nutlin‐3a (*K*_d_=0.02–0.03 μM).^[33]^

## Conflict of interest

The authors declare no conflict of interest.

1

## Supporting information

As a service to our authors and readers, this journal provides supporting information supplied by the authors. Such materials are peer reviewed and may be re‐organized for online delivery, but are not copy‐edited or typeset. Technical support issues arising from supporting information (other than missing files) should be addressed to the authors.

Supporting Information

## Data Availability

The data that support the findings of this study are available in the supplementary material of this article.
