# Supplementary material for: Conformationally Controlled sp3‐Hydrocarbon‐Based α‐Helix Mimetics
Source: Angew Chem Int Ed Engl. 2023 May 2;62(23):e202301209. doi: 10.1002/anie.202301209 (PMC10953326; doi:10.1002/anie.202301209)
Supplement: Supplementary file 1 — Supporting Information [file ANIE-62-0-s001.pdf]

Angewandte Chemie
Eine Zeitschrift der Gesellschaft Deutscher Chemiker

Supporting Information

Conformationally Controlled sp^3-Hydrocarbon-Based α-Helix Mimetics

L. I. Dewis, M. Rudrakshula, C. Williams, E. Chiarparin, E. L. Myers, C. P. Butts*, V. K. Aggarwal**

Table of Contents

1. Molecular Mechanics Conformational Searching 4

1.1 General Informational 4
1.2 Conformational Search Results 6
1.2.1 The conformational search of **19** 6
1.2.2 The conformational search of **4** 7
1.2.3 The conformational search of **5** 8
1.2.4 The conformational search of **13** 9
1.2.4 The conformational search of **14** 10

2. Molecular Docking using AutoDock Vina 13

2.1 The binding of Nutlin-2 by AutoDock Vina 14
2.2 The binding of **4** by AutoDock Vina 15
2.3 The binding of **5** by AutoDock Vina 17

3. Synthetic Procedures 19

3.1 General Synthetic Information 19
3.2 General Synthetic Procedures 21
3.3 Preparation of Compounds 23

4. The Calculation of Gibbs Free Energies, Boltzmann Populations and Boltzmann Averaged NMR Parameters using Density Functional Theory 77

4.1 General Computational Information 78
4.2 The Calculation of Boltzmann Averaged Chemical Shifts 79
4.3 The Calculation of Boltzmann Averaged Scalar Coupling Constants 79
4.4 The Calculation of Boltzmann Averaged Interproton Distances 80
4.5 The DFT Calculated Gibbs free energies and Boltzmann Populations for Conformers of **4** 81

5. The Acquisition of NMR Parameters in Conformational Analysis 83

5.1 The Measurement of $^{n}J_{HH}$ Scalar Coupling Constants 83
5.2 The Measurement of $^{n}J_{CH}$ Scalar Coupling Constants 87
5.3 The Measurement of Quantitative Interproton Distances 90

6. The Conformational Analysis of **4** using NMR Spectroscopy and Computation 92

6.1 A Comparison of Experimental and Calculated $^{n}J_{HH}$ Scalar Coupling Constants 92
6.2 A Comparison of Experimental and Calculated $^{n}J_{CH}$ Scalar Coupling Constants 93
6.3 A Comparison of Experimental and Calculated Interproton Distances 95
6.4 A Comparison of Experimental and Calculated Chemical Shifts 96

7. The Binding of the Designed Inhibitors to Mdm2 using ^{1}H-^{15}N HSQC Spectroscopy 98

7.1 The Expression and Purification of ^{15}N-Mdm2 98
7.2 The binding of **15** by TROSY spectroscopy 107
7.3 The Estimation of K_d using ^{1}H-^{15}N SOFAST HMQC for **15** 109

7.4 The Estimation of K_d using ^{1}H-^{15}N SOFAST HMQC for **16** 114
7.5 The Estimation of K_d using ^{1}H-^{15}N SOFAST HMQC for **17** 117
7.6 The Estimation of K_d using ^{1}H-^{15}N SOFAST HMQC for **18** 120

8.0 NMR Spectra 123

9.0 References 143

1. Molecular Mechanics Conformational Searching

1.1 General Informational

Molecular Mechanics Conformational Search Calculations were performed using the MacroModel[1] Software package (version 9.9) accessed through Maestro (version 9.2)[2]. All MM calculations were performed using Grendel, the Unix computational resource of the School of Chemistry, University of Bristol.

Conformational searching was performed using the Monte Carlo Multiple Minimum (MCMM)[3] conformational search method. The Merck Molecular Force Field (MMFFs)[4] was used with 500,000 steps in the gas phase. Conformers within 21 kJ mol^{-1} of the global energy minima were retained, unless stated otherwise. Structures were minimised using the Truncated Newton Conjugate Gradient (TNCG) method with 1000 iterations and a convergence criterion of 0.05 Å. If poor convergence was observed, noted by a low number of times the low energy conformers were found, the number of iterations was increased to 5000.

Following the conformational search, a redundant conformer elimination was performed using MacroModel. In this study, conformers are deemed redundant and eliminated using maximum atom deviations. This considers a conformer to be unique if the maximum atom deviation for any pair of corresponding atoms exceeds 0.5 Å. The atoms along the backbone and the first carbon atom of the side chains were selected for comparison (**Figure 1**). All conformers under 21 kJ mol^{-1} were retained.

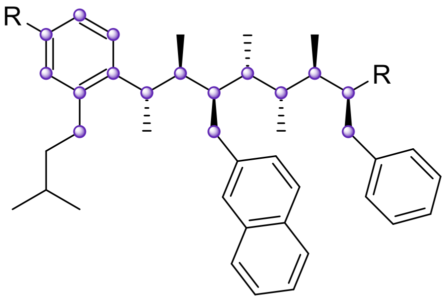

Figure 1. The atoms considered for comparison during the redundant conformer elimination.

To generate the bubble plots, the dihedral angles along the backbone of each conformer were measured in MacroModel. The estimated relative potential energies of each conformer from MM was used to calculate the Boltzmann distribution of conformers according to Equation 1. A 3D plot was then created using the measured dihedral angles, the dihedral number and the conformer populations.

$$p_i = \frac{g e^{-\frac{\mathrm{D}E}{RT}}}{\sum_{i=1}^{n} g e^{-\frac{\mathrm{D}E}{RT}}}$$

Equation 1. The Boltzmann Distribution

Where ΔE is the estimated potential energy, relative to the global minimum, in kJ mol^{-1} of conformer i. R is the ideal gas constant (8.314×10^{-3} kJ mol^{-1}), T is the temperature of the system (298 K) and g is the degeneracy of conformer i (which is 1 for all conformers studied).

1.2 Conformational Search Results

1.2.1 The conformational search of 19

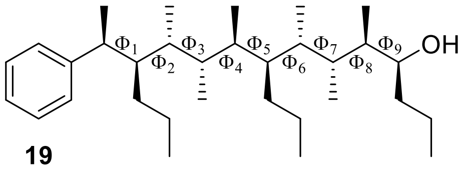

19

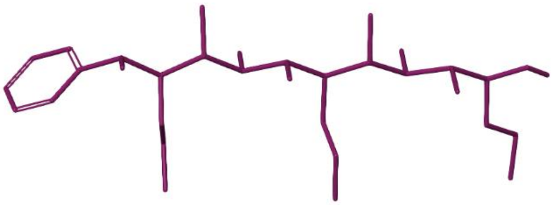

Figure 2. The lowest energy conformer of **19**

Table 1. The MM conformational search results of **19**

Number of Conformers within 21 kJ mol^{-1}	Number of Conformers after Redundant Conformer Elimination	% of Linear Structures.
2716	198	73%

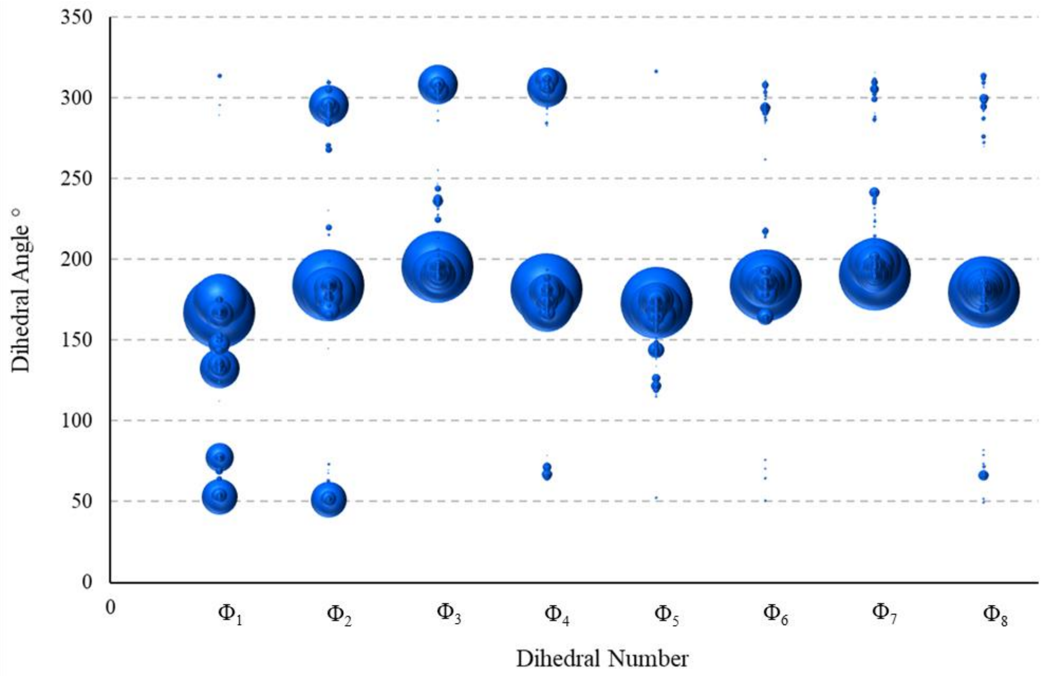

Figure 3. The Bubble plot of **19**, summarising the results of the conformational search. The size of the bubble is representative of the population of the conformer contributing to that dihedral angle. Dihedral angles of 180° can be seen along the backbone of the molecules, indicating a linear conformation.

1.2.2 The conformational search of 4

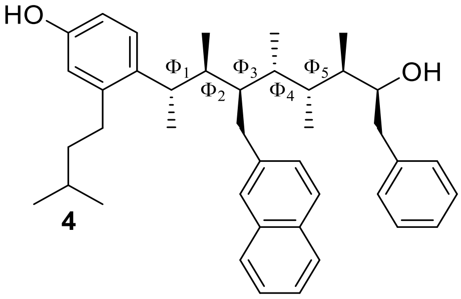

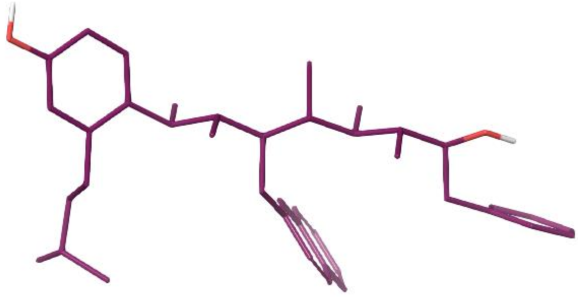

Figure 4. The lowest energy conformer of **4**

Table 2. The MM conformational search results of **4**

Number of Conformers within 30 kJ mol^{-1}	Number of Conformers after Redundant Conformer Elimination	% of Linear Structures.
17380	1115	77%

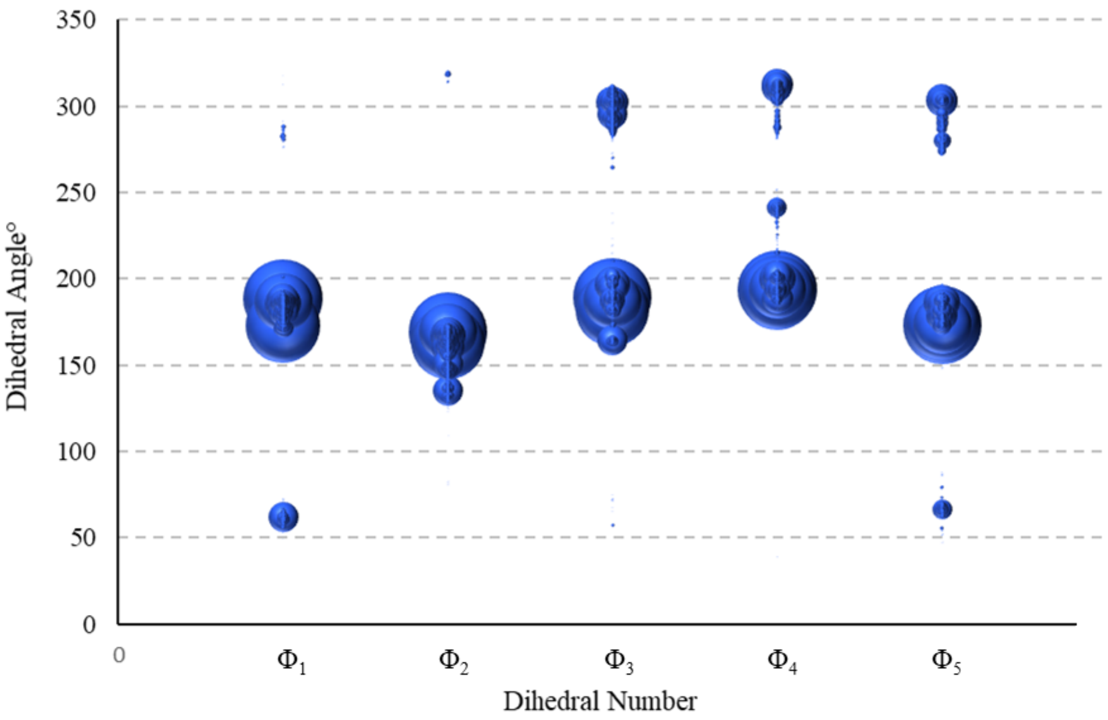

Figure 5. The Bubble plot of **4**, summarising the results of the conformational search. The size of the bubble is representative of the population of the conformer contributing to that dihedral angle.

1.2.3 The conformational search of 5

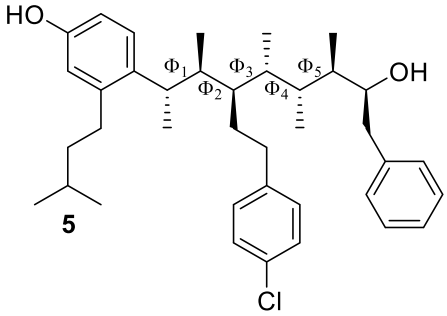

5

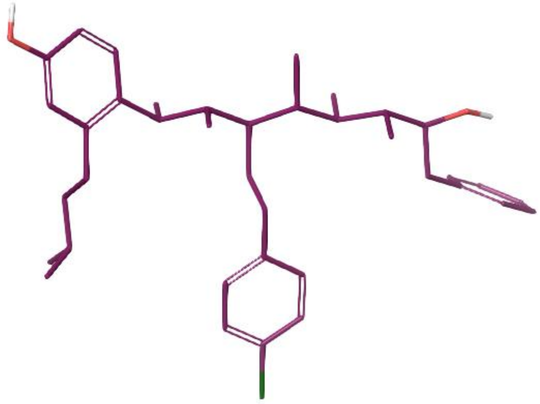

Figure 6. The lowest energy conformer of **5**

Table 3. The MM conformational search results of **5**

Number of Conformers within 21 kJ mol^{-1}	Number of Conformers after Redundant Conformer Elimination	% of Linear Structures.
6656	369	80%

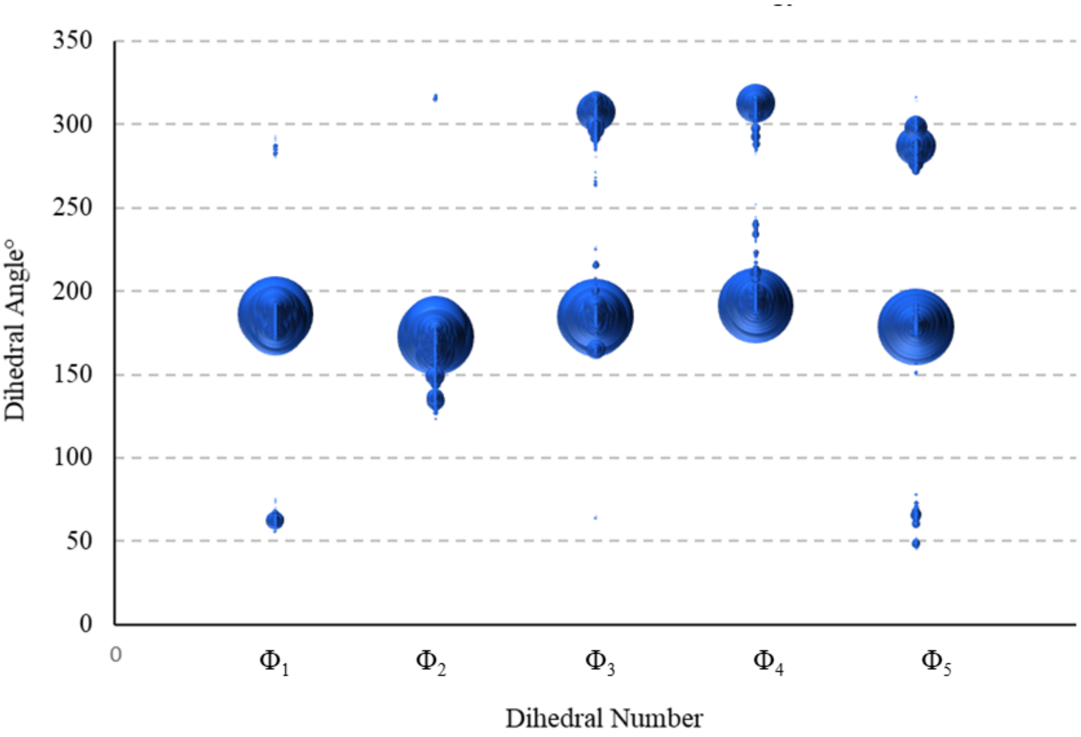

Figure 7. The Bubble plot of **5**, summarising the results of the conformational search. The size of the bubble is representative of the population of the conformer contributing to that dihedral angle.

1.2.4 The conformational search of 13

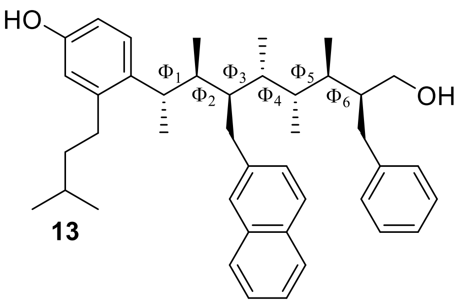

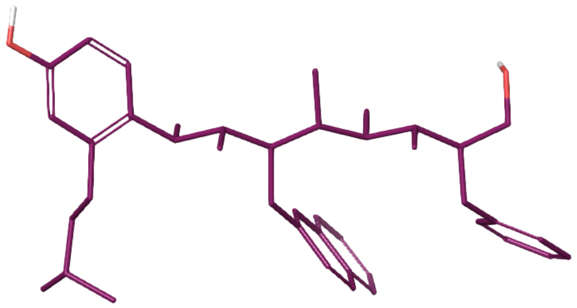

Figure 8. The lowest energy conformer of **13**

Table 4. The MM conformational search results of **13**

Number of Conformers within 21 kJ mol^{-1}	Number of Conformers after Redundant Conformer Elimination	% of Linear Structures.
9577	200	72%

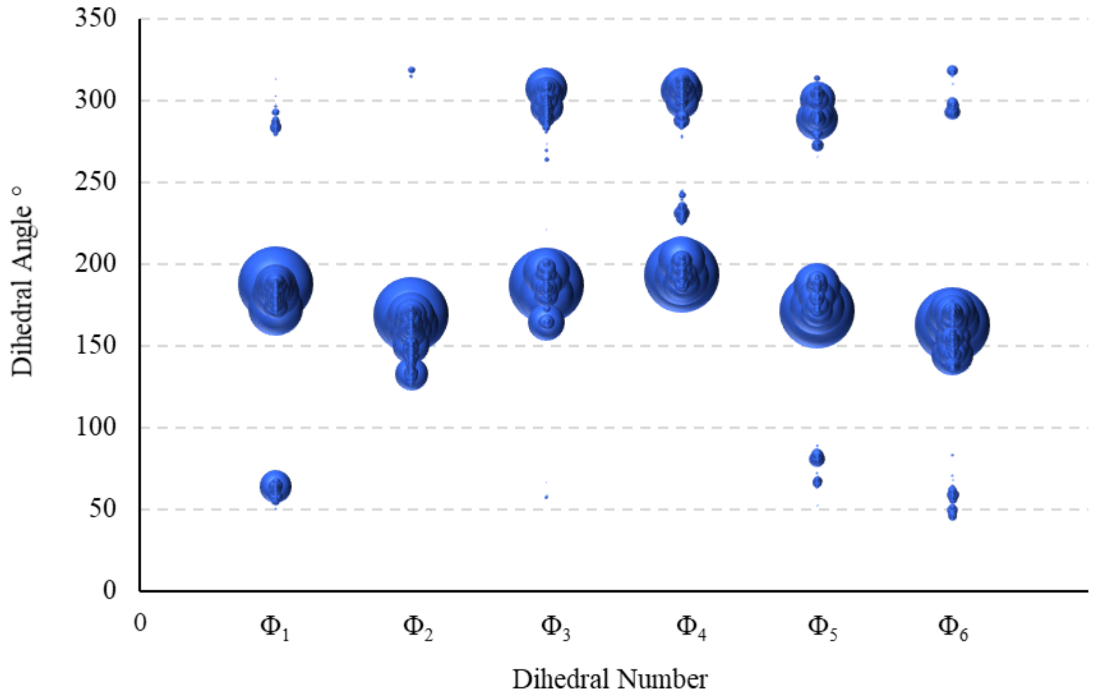

Figure 9. The Bubble plot of **13**, summarising the results of the conformational search. The size of the bubble is representative of the population of the conformer contributing to that dihedral angle.

1.2.4 The conformational search of 14

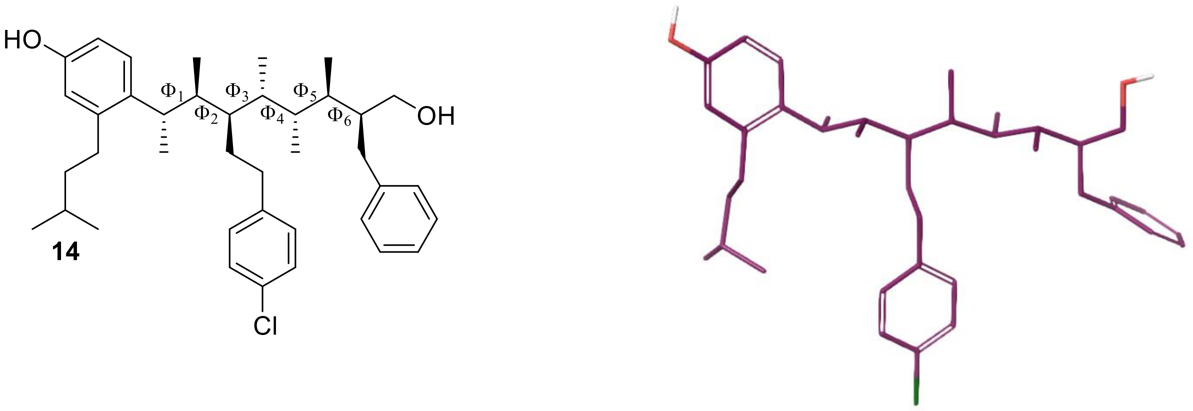

Figure 10. The lowest energy conformer of **14**

Table 5. The MM conformational search results of **14**

Number of Conformers within 21 kJ mol^{-1}	Number of Conformers after Redundant Conformer Elimination	% of Linear Structures.
6277	188	75%

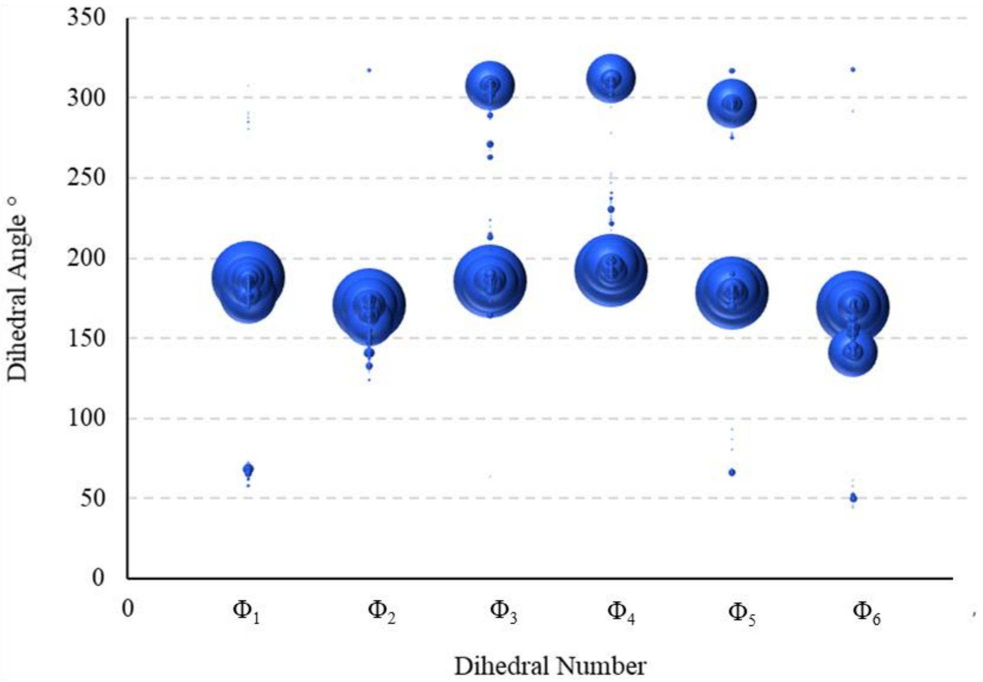

Figure 11. The Bubble plot of **14**, summarising the results of the conformational search. The size of the bubble is representative of the population of the conformer contributing to that dihedral angle.

1.2.5 The conformational search of 40

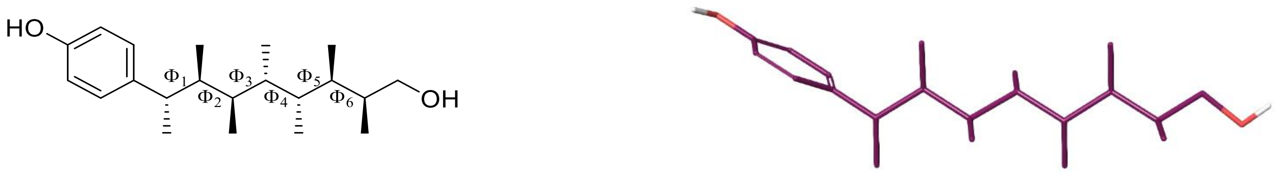

Figure 12. The lowest energy conformer of **40**

Table 6. The MM conformational search results of **40**

Number of Conformers within 21 kJ mol^{-1}	Number of Conformers after Redundant Conformer Elimination	% of Linear Structures.
130	n/a	98%

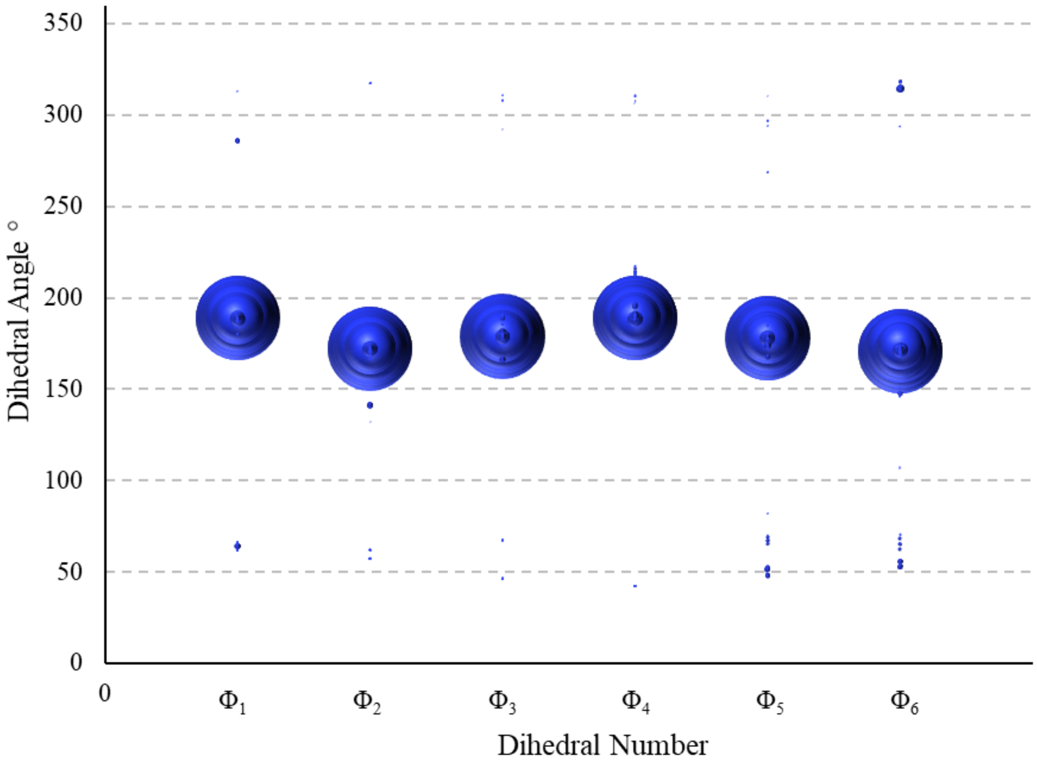

Figure 13. The Bubble plot of **40**, summarising the results of the conformational search. The size of the bubble is representative of the population of the conformer contributing to that dihedral angle.

1.2.6 The conformational search of 47

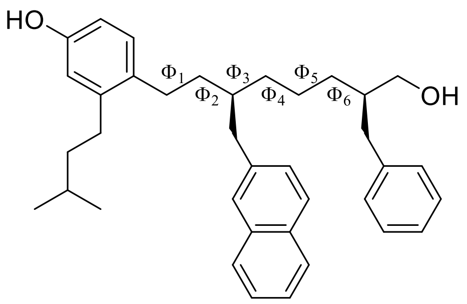

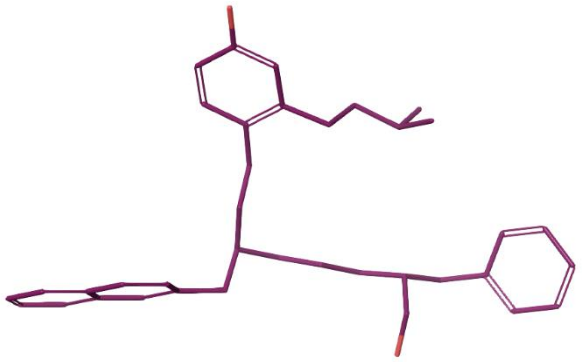

Figure 14. The lowest energy conformer of **47**

Table 7. The MM conformational search results of **47**

Number of Conformers within 21 kJ mol^{-1}	Number of Conformers after Redundant Conformer Elimination	% of Linear Structures.
86446	1581	<0.1%

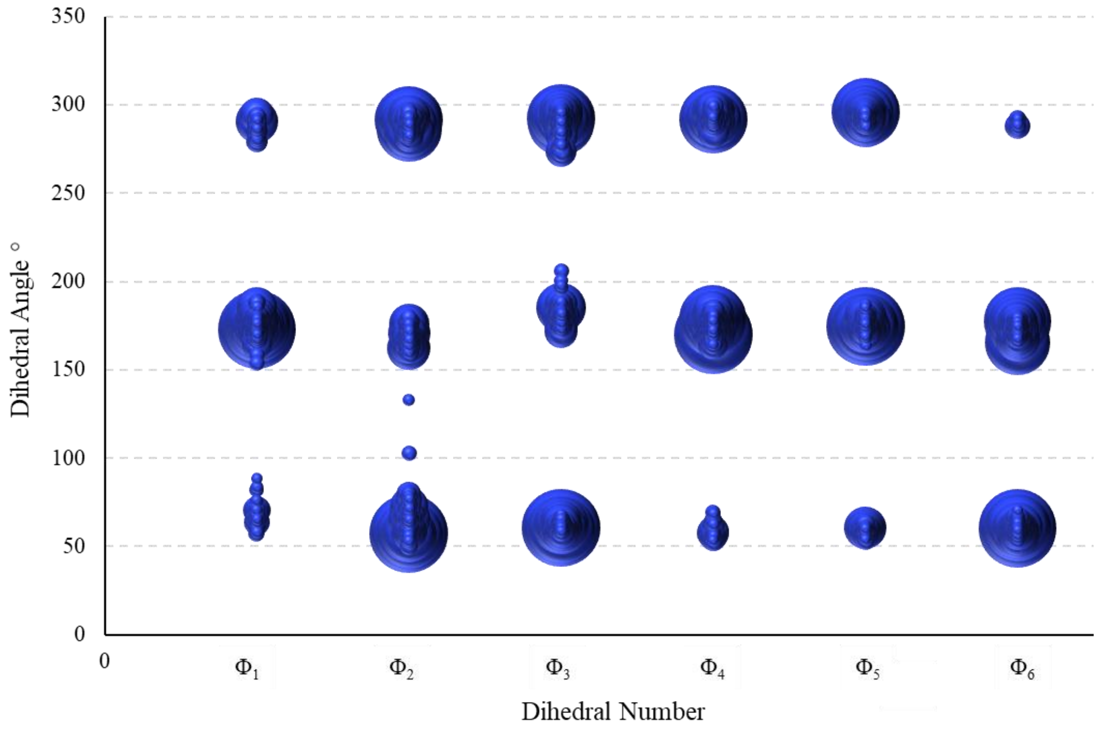

Figure 15. The Bubble plot of **47**, summarising the results of the conformational search. The size of the bubble is representative of the population of the conformer contributing to that dihedral angle.

2. Molecular Docking using AutoDock Vina

Molecular Docking was performed using AutoDock Vina, version 1.1.2.[5] All procedure for the docking experiment were followed as described in the user manual for AutoDock Vina with the exception of the exhaustiveness, which was increased to 24. The ligand and receptor were parameterised using Autodock Tools. Docked conformations were ranked automatically by AutoDock Vina using a force field scoring function. The parameters (centre point of binding and size of the search space) used in the calculation are:

center_x = 30.804
center_y = -18.102
center_z = -3.946
size_x = 30.0
size_y = 30.0
size_z = 24.0

2.1 The binding of Nutlin-2 by AutoDock Vina

Mdm2 was taken from the crystal structure 1YCR and the lowest energy conformer of Nutlin-2, found from a MM conformational search, was used as the ligand. The best binding mode gave a predicted binding affinity of -9.9 kcal mol^{-1}.

Table 8. The top five binding poses of Nutlin-2 bound to Mdm2 found by AutoDock-Vina.

Mode	Affinity (kcal mol^{-1})	Distance from Best Mode (Å)
1	-9.9	0.00
2	-9.5	7.94
3	-9.0	7.46
4	-8.1	4.95
5	-7.9	7.15

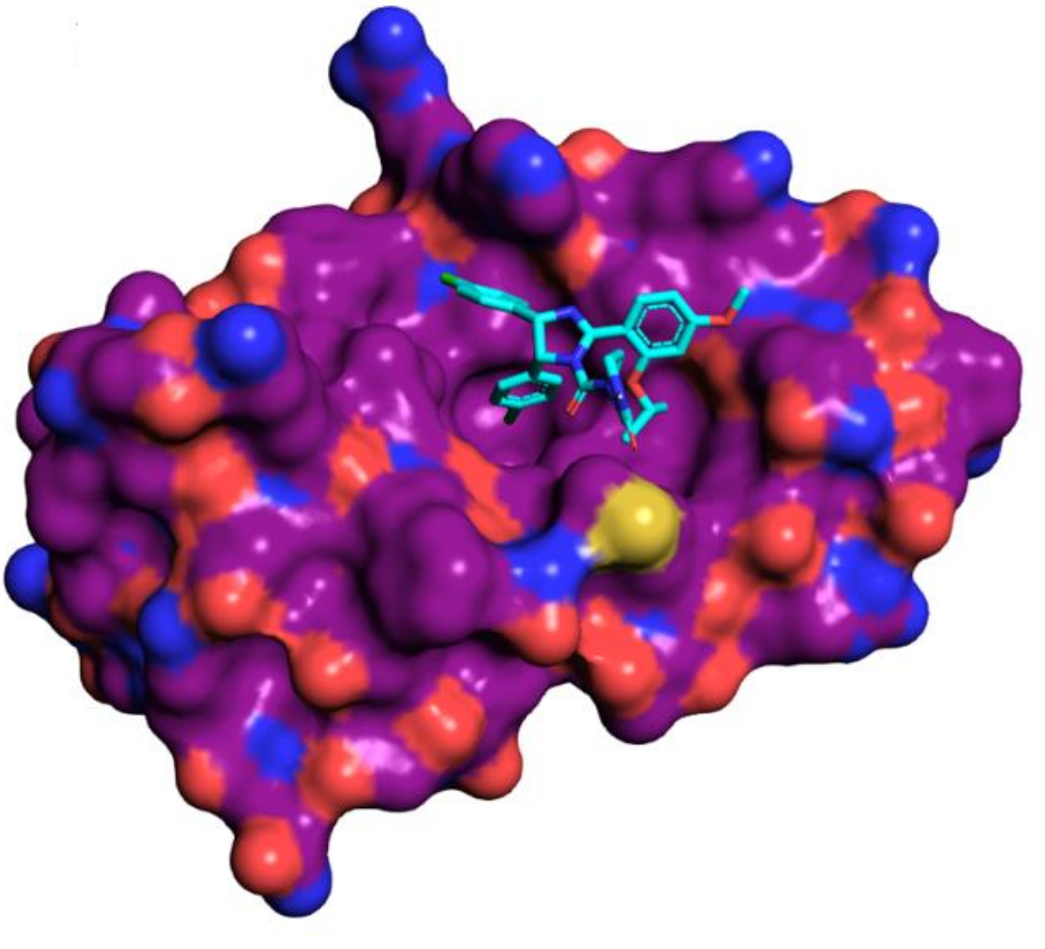

Figure 16. The top ranked binding pose of Nutlin-2 bound to Mdm2 found by AutoDock Vina.

2.2 The binding of 4 by AutoDock Vina

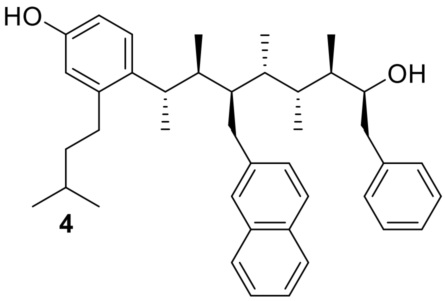

4

Mdm2 was taken from the crystal structure 1YCR and the lowest energy conformer of **4**, found from a MM conformational search, was used as the ligand. The best binding mode gave a predicted binding affinity of -8.8 kcal mol^{-1}.

Table 9. The top five binding poses of **4** bound to Mdm2 found by AutoDock-Vina.

Mode	Affinity (kcal mol^{-1})	Distance from Best Mode (Å)
1	-8.8	0.00
2	-8.2	2.45
3	-7.8	11.19
4	-7.7	8.13
5	-7.6	9.69

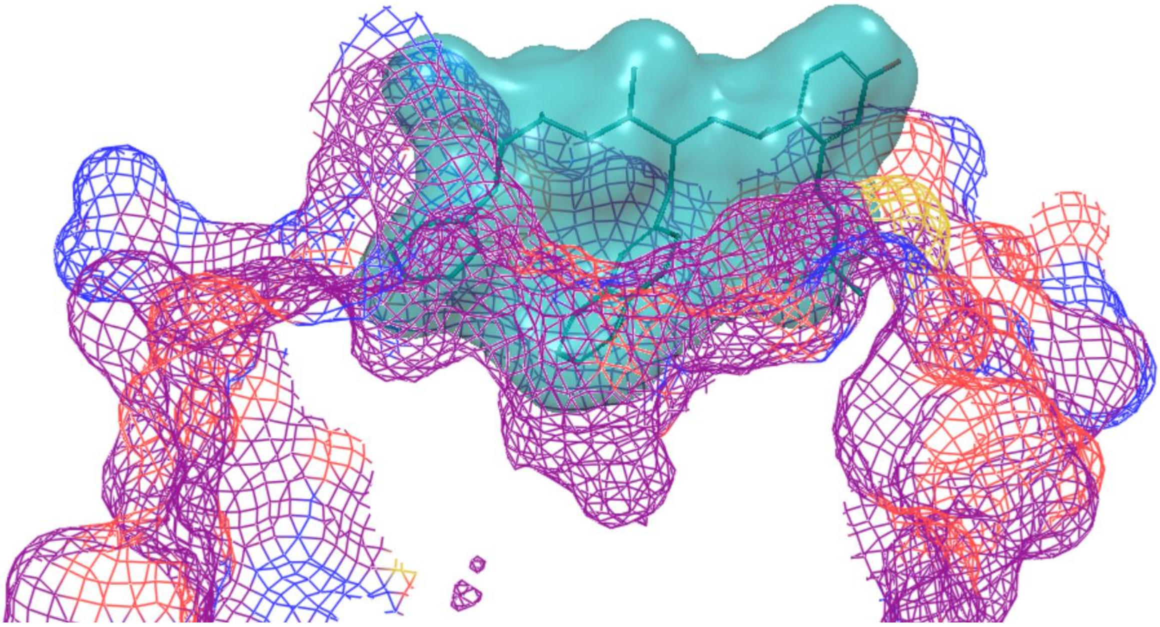

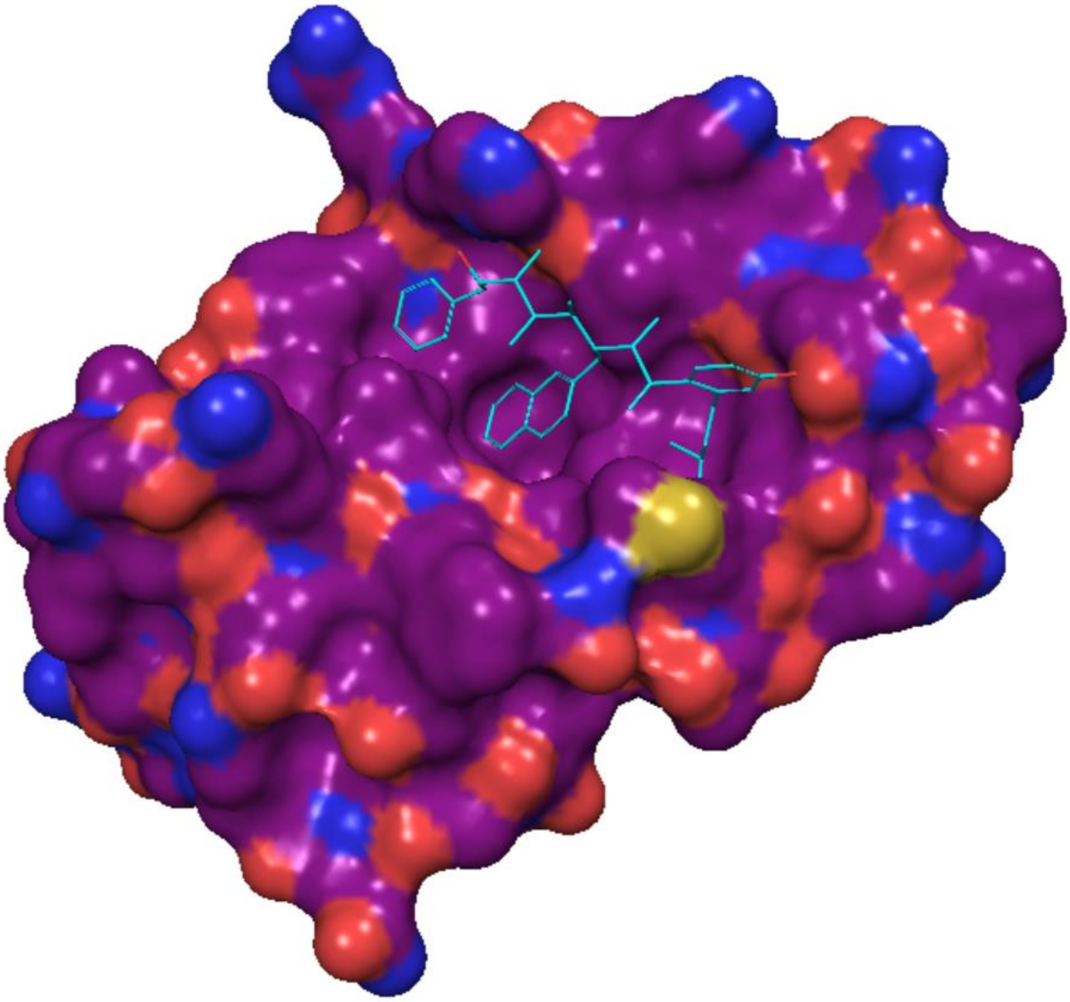

Figure 17. The top ranked binding pose of **4** bound to Mdm2 found by AutoDock Vina. A surface representation of **4** bound to Mdm2 is shown to display the occupancy of the p53 pocket on Mdm2 by **4**.

2.3 The binding of 5 by AutoDock Vina

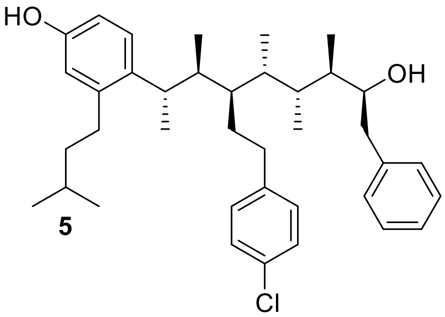

5

Mdm2 was taken from the crystal structure 1YCR and the lowest energy conformer of **5**, found from a MM conformational search, was used as the ligand. The best binding mode gave a predicted binding affinity of -9.3 kcal mol^{-1}.

Table 10. The top five binding poses of **5** bound to Mdm2 found by AutoDock-Vina.

Mode	Affinity (kcal mol^{-1})	Distance from Best Mode (Å)
1	-9.3	0.00
2	-8.9	2.38
3	-8.6	2.23
4	-8.5	2.59
5	-8.1	4.33

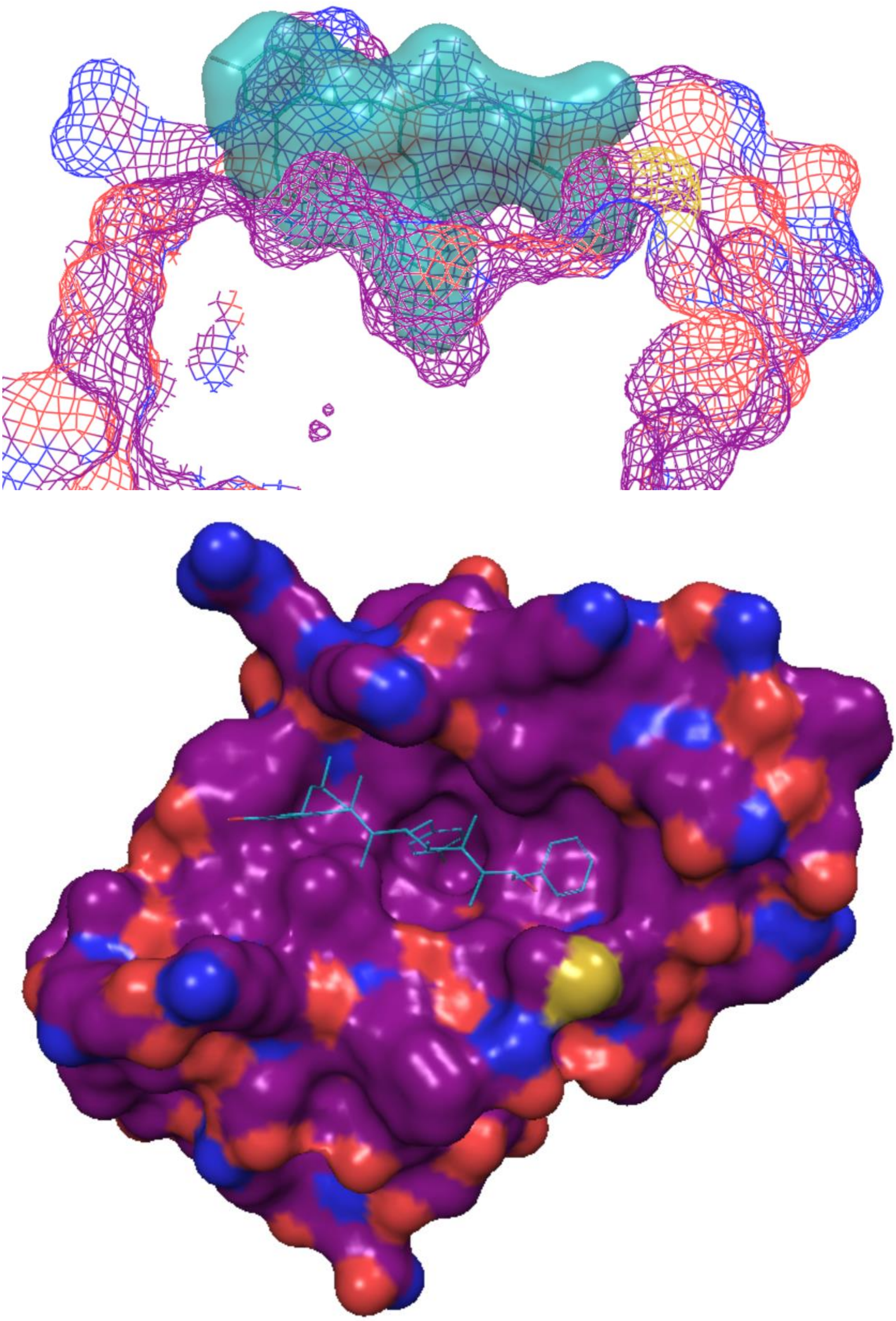

Figure 18. The top ranked binding pose of **5** bound to Mdm2 found by AutoDock Vina. A surface representation of **5** bound to Mdm2 is shown to display the occupancy of the p53 pocket on Mdm2 by **5**.

3. Synthetic Procedures

3.1 General Synthetic Information

All required fine chemicals were purchased from Acros Organics, Alfa Aesar, Fischer Scientific or Sigma-Aldrich and used as received unless otherwise specified. *s*-butyllithium (*s*-BuLi and *n*-butyllithium (*n*-BuLi) were received from Acros Organics and the molarity was verified by titration with N-benzylbenzamide.[6] (–)-sparteine and (+)-sparteine were distilled over CaH_2 and stored in a young's tube under Argon/N_2 at -5 °C to prevent absorption of atmospheric CO_2. Anhydrous solvents were dried by passing through a modified Grubbs system of alumina columns, manufactured by Anhydrous Engineering, stored over 3Å molecular sieves (25% of total volume) and transferred under N_2 *via* syringe. Ethyl 2,4,6-triisopropylbenzoate was provided by Dr. Johan Pradeilles, a previous member of the Aggarwal group.

All air- and water- sensitive reactions were carried out in flame dried glassware under a N_2 atmosphere using standard Schlenk manifold techniques with magnetic stirring. Where reactions were monitored using TLC, aluminium backed plates pre-coated (0.25 mm) with Merck Silica Gel 60 F254 were used and compounds were visualised by exposure to UV light or stained using a 5% solution of phosphomolybdic acid (PMA) in EtOH followed by heating. Where compounds were either purified by flash column chromatography or passed through a plug of silica, Merck Silica Gel 60 (40-63 μm) was used. All mixed solvent eluents are reported as v/v solutions.

^{1}H- and ^{13}C- NMR spectra were recorded on either a JEOL ECZ 300 MHZ, JEOL ECS 400 MHz, Varian 400 MHz, Varian VNMRS 500 MHz NMR spectrometers equipped with direct observe two channel probes, a Bruker AVANCE III HD 500 MHz NMR spectrometer with a 5 mm DCH ^{13}C-^{1}H/D Cryo Probe or a Bruker AVANCE III HD 700 MHz NMR Spectrometer with a 1.7 mm inverse triple resonance micro-Cryo Probe. Spectra were visualised and processed using MestreNova version 14.0. Chemical shifts (δ) are quoted in parts per millions (ppm) and are referenced to the residual proton signals of the solvent. ^{1}H NMR coupling constants are reported in Hz. Data are reported as follows: chemical shift, multiplicity (s = singlet, br. s = broad singlet, d = doublet, t = triplet, q = quartet, quin = quintet, sext = sextet, sept = septet, m = multiplet, dd = doublet of doublets etc.), coupling constant, integration and assignment. Assignment of signals in ^{1}H and ^{13}C NMR spectra was performed using ^{1}H-^{1}H COSY, ^{1}H-^{1}H-gDQFCOSY, DEPT, ^{1}H-^{13}C HSQC, ^{1}H-^{13}C pureshift-HSQC, ^{1}H-^{13}C HMBC, ^{1}H-^{13}C H2BC experiments where appropriate. ^{11}B NMR spectra were measured using Norell S-200-QTZ quartz NMR tubes at 96 or 128 MHz with complete proton decoupling. It should be noted that ^{13}C signals adjacent to boron are generally not observed due to quadrupolar relaxation.

GCMS was performed using an Agilent HP-5MS column (15 m × 0.250 mm), an Agilent 6890 GC, and Agilent 5973 MS system. Compounds were identified through extracted ion chromatogram and molecular ion analysis. Method 70-1X: Inlet temperature 250 °C; Flow rate: 1.0 mL/min; hold at 70 °C for 0 min; ramp 20 °C/min to 200 °C; ramp 45.0 °C/min to 300 °C; hold at 300 °C for 2 min.

Chiral high pressure liquid chromatography (HPLC) separations were performed on an Agilent 1100 Series HPLC unit equipped with UV-vis diode-array detector monitored at 210.8 nm, using Daicel Chiralpak IA, IB or IC columns (4.6 × 250 mm2, 5 μm) fitted with respective guards (4 × 10 mm2).

High resolution mass spectra were recorded by the University of Bristol, School of Chemistry departmental mass spectrometry service using electron spray ionisation (ESI) or matrix assisted laser desorption (MALDI). HRMS ESI was performed on either a Bruker Daltonics Apex IV, 7-Tesla FT-ICR or microTOF II. MALDI was performed in an Applied Biosystems 4700 Proteomics Analyser Instrument. Samples were submitted in either EtOAc or $CHCl_3$.

All Infrared spectra were recorded on the neat compounds using a PerkinElmer Spectrum One FT-IR spectrometer, irradiating between 4000 cm-1 and 600 cm-1. Only strong and selective absorbances above 1400 cm^{-1} are reported. Melting points were measured with a Stuart SMP30 melting point apparatus and are uncorrected. Optical rotations were obtained on a Bellingham + Stanley Ltd. ADP220 polarimeter at 589 nm in a cell with a path length of 1dm. Specific rotations are given in (deg mL)/(g dm). Compound names are those generated by ChemBioDraw 13.0 (PerkinElmer), following the IUPAC nomenclature

Reverse-phase preparative high performance liquid chromatography (HPLC) was performed on an Agilent 1260 Infinity II system with an Agilent 5 Prep-C18 column (50 × 10.0 mm, 5 μm) and acetonitrile/water mobile phase.

3.2 General Synthetic Procedures

1. The Homologation of Boronic Esters using Stannanes (GP1)

A solution of stannane **7** (1.35 eq) in a Schlenk reaction vessel was dissolved in anhydrous Et_2O (0.2 M) under an atmosphere of nitrogen. The reaction mixture was cooled to -78 °C and *n*-BuLi (1.5 – 1.6 M in hexanes, 1.30 eq) was added dropwise to the reaction mixture. The reaction mixture was stirred for 1 h at -78 °C after which the reaction mixture has become a translucent pale yellow solution with no white stannane precipitate remaining. The boronic ester (0.3 M in anhydrous Et_2O, 1 eq) was added dropwise at -78 °C. The reaction mixture was stirred at -78 °C until the pale yellow solution loses its colour on the addition of the boronic ester. The reaction mixture was then warmed to room temperature, and the 1,2- metallate rearrangement was monitored by ^{11}B NMR. Once the 1,2-metallate rearrangement was complete a white precipitate was formed and filtered through a 1cm plug of silica, to give a pale yellow translucent solution. The silica was washed with Et_2O and the solvent removed (*in situ*) under reduced pressure to give the crude boronic ester. The crude boronic ester was then either purified by flash column chromatography or used directly in subsequent homologations.

2. The Homologation of Boronic Esters using Primary Benzoates (GP2)

A solution of benzoate **8**, **9** or **10** (1.35 eq) and (+)-sparteine (1.30 eq) in a Schlenk reaction vessel was dissolved in anhydrous Et_2O (0.2 M) under an atmosphere of nitrogen. The reaction mixture was cooled to -78 °C and *s*-BuLi (1.3 - 1.4 M in hexanes, 1.30 eq) was added dropwise to the reaction mixture. The reaction mixture was stirred for 1 h at -78 °C during which it became dark red in colour. The boronic ester (0.5 M in anhydrous Et_2O, 1 eq) was added dropwise at -78 °C. The reaction mixture was stirred at -78 °C until the dark red solution loses its colour and becomes yellow. The reaction mixture was then warmed to room temperature, and the 1,2-metallate rearrangement was monitored by ^{11}B NMR. The reaction mixture was then quenched with 2M HCl and the aqueous layer was washed with Et_2O three times. The organic layers were combined, dried using $MgSO_4$, filtered and concentrated in vacuo. The crude boronic ester was then purified by flash column chromatography.

3. The Matteson Homologation of Boronic Esters (GP3)

A solution of boronic ester (1.0 eq) and bromochloromethane (2.5 eq) in an oven-dried Schlenk reaction vessel was dissolved in anhydrous Et_2O (0.2 M) under an atmosphere of nitrogen. The reaction mixture was cooled to -78 °C and *n*-BuLi (1.6 M in hexanes, 2.5 eq) was added dropwise at a rate of approximately 40 $\mu L/min^{-1}$. The reaction mixture was stirred for 30 min at -78 °C after which it was stirred at room temperature for 1 h. The reaction mixture was filtered through silica [~10 mm depth of wetted (Et2O) silica, using a filter frit connected directly to an oven-dried receiving vessel] to give a colourless to pale yellow translucent solution. The silica was washed with Et_2O, the filter frit was removed and solvent was evaporated (*in situ*) under reduced pressure

to give the crude boronic ester. The crude boronic ester was dried under high vacuum and used in further homologations.

4. The PEGylation of the bis-alcohols

In an oven dried Schlenk reaction vessel, a solution of bis-alcohol (1.0 eq) in anhydrous DCM was cooled to 0 °C. Pyridine (10.0 eq) was added dropwise followed by the addition of phosgene (15 wt. % in toluene, 10.0 eq) dropwise to the solution. The reaction mixture was stirred for 30 minutes at 0 °C after which it was warmed to 37 °C and stirred for another 12 h. The reaction was monitored by TLC and after consumption of the starting bis-alcohol, a further 10 eq of pyridine were added followed by a solution of methoxypolyethylene glycol amine (average Mw of 750, 10.0 eq) in dry DCM. The reaction mixture was stirred at 37 °C for a further 12 h. The reaction mixture was concentrated *in situ* and the crude material passed through a plug of silica [~30 mm depth of wetted (Hexane) silica]. The PEGylated products were removed by washing with 2:8 MeOH:DCM and concentrated. The crude PEGylated products were finally purified by RP-HPLC (1:10 AcCN:H_2O) to yield the bis-PEGylated products.

3.3 Preparation of Compounds

1-(Trimethylstannyl)ethyl 2,4,6-triisopropylbenzoate, (*S*)-7

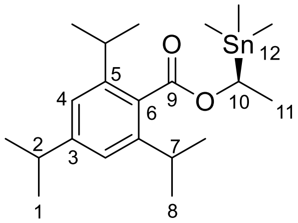

An oven dried 1 L, three neck flash, equipped with a 40 mm magnetic stirrer bar was cooled to rt under vacuum. The reaction flask was evacuated and refilled with N_2 (3x). The flask was charged with ethyl 2,4,6-triisopropylbenzoate (7.98 g, 28.9 mmol, 1.0 eq) and (–)-sparteine (8.3 mL, 37.5 mmol, 1.3 eq) followed by the addition of anhydrous Et_2O (135 mL) *via* cannula. The solution was cooled to -78 °C and allowed to equilibrate for 10 mins before the addition of *s*BuLi (1.3 mL in hexanes, 28.8 mL, 37.5 mmol, 1.3 eq) dropwise to the solution. The reaction mixture became dark brown after the addition of *s*-BuLi. The reaction mixture was stirred at -78 °C for 4 h before the addition of Me_3SnCl (1.0 M in hexanes, 37.5 mL, 37.5 mmol, 1.3 eq) to the reaction mixture. The reaction mixture was stirred at -78 °C for 20 mins, after which it had become a yellow solution. It was then warmed to rt and stirred for 1 h. The reaction mixture was diluted with 2 M HCl (60 mL) and stirred for a further 20 mins. The organic and aqueous layers were separated, and the organic layer was washed with 2 M HCl (4 x 60 mL). The combined aqueous layers were extracted with Et_2O (3 x 60 mL). The combined organic layers were dried ($MgSO_4$), filtered and concentrated *in vacuo* to give the crude stannane **(*S*)-7** (12.3 g, e.r. 91:9) as an off white solid.

The opposite enantiomer **(*R*)-7** was synthesised in identical yields and e.r. by substituting (–)-sparteine for (+)-sparteine. Stannanes used in this project were dried under high vacuum (1 mbar) with stirring overnight.

Recrystallisation of (*S*)-7

The crude stannane was dissolved in MeOH (3 mL/g) by bringing the solution to reflux. The solution was then allowed to cool to rt. Crystals appeared after 10 min to 5 h depending on the purity of the stannane. The white crystals were filtered and dried under reduced pressure. The recrystallisation was repeated until the e.r. was >99:1. After two recrystallisations **(*S*)-7** was obtained (6.4 g, 52%, e.r. 99.9:0.1) as a colourless solid.

Sparteine Recovery[7]

The combined aqueous layers were made basic with NaOH (20%). The aqueous phase was washed with Et_2O (3 x 60 mL) and the combined organic layers were dried (K_2CO_3), filtered and concentrated *in vacuo* to give crude sparteine. Distillation over CaH_2 of the crude material gave (–)-sparteine (6.7 mL, 80%) as a colourless oil.

Spectral data were in accordance with the published values[7]

^{1}H NMR (400 MHz, $CDCl_3$) δ: 6.99 (s, 2H, H_4), 5.04 (q, J = 7.4 Hz and quin, J = 7.7 Hz, 1H, H_{10}), 2.79 – 2.92 (m, 3H, H_2 and H_7), 1.59 (d, J = 7.6 Hz and dd, J = 56.5, 7.6 Hz and dd, J = 56.5, 7.6 Hz, 3H, H_{11}), 1.24 (d, J = 6.7 Hz, 18H, H_1 and H_8), 0.18 (s and d, J = 52.7 Hz and d, J = 52.7 Hz, 9H, H_{12})

^{13}C NMR (101 MHz, $CDCl_3$) δ: 171.4 (C_9), 150.0 (Ar), 144.9 (Ar), 130.9 (Ar), 120.9 (C_4), 67.1 (C_{10}), 34.5 (C_2), 31.4 (C_8), 24.4 (C_1 and C_8), 24.2 (C_1 or C_8), 24.0 (C_1 or C_8), 19.3 (C_{11}), -9.8 (d, J = 334 Hz (13C-119Sn) and d, J = 318 Hz (13C-117Sn), C_{12}).

Chiral HPLC: (Daicel Chiralpak-IB column (25 cm) with guard, hexane 0.7 mL/min, rt, 210.8 nm): t_R = 6.4 (*S*), 9.2 (*R*), e.r. = 99.9:0.1

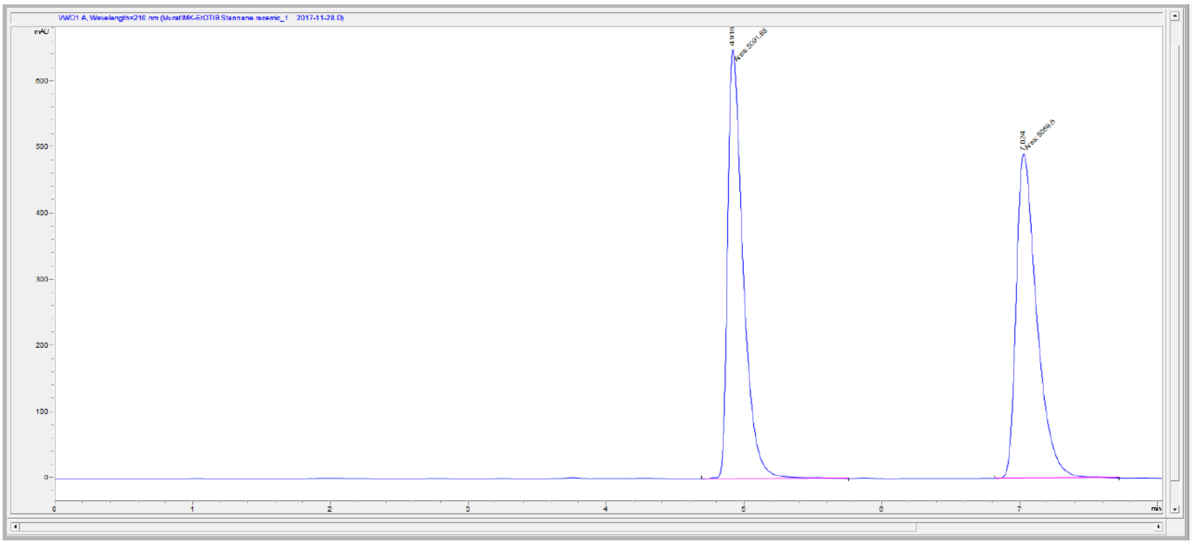

#	Time	Type	Area	Height	Width	Area%	Symmetry
1	4.916	MM	5091.9	649.5	0.1307	50.011	0.516
2	7.024	MM	5089.6	489.9	0.1731	49.989	0.561

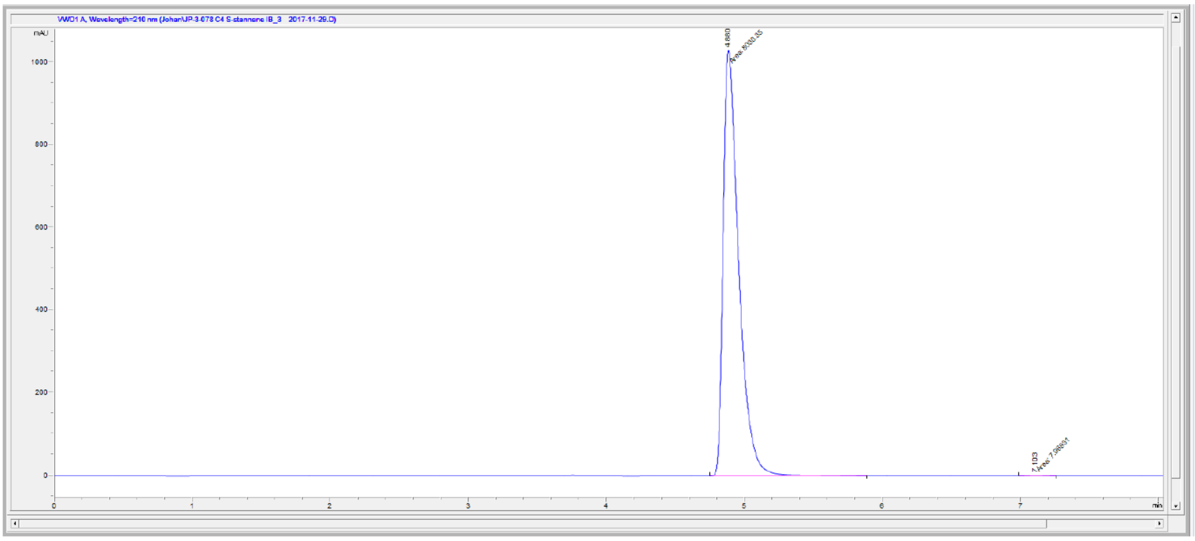

#	Time	Type	Area	Height	Width	Area%	Symmetry
1	4.88	MM	8030.4	1028.8	0.1301	99.901	0.507
2	7.103	MM	8	9.3E-1	0.1422	0.099	0.849

Figure 19. The Chiral-HPLC traces for Racemic **7** (top) and **(*S*)-7** (bottom).

2-(naphthalen-2-yl)ethyl 2,4,6-triisopropylbenzoate, 8

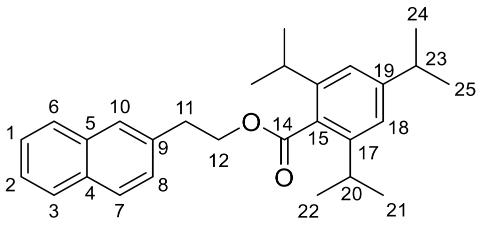

To a stirred solution of PPh_3 (1.67 g, 6.39 mmol, 1.1 eq), 2-naphthalene alcohol (1.00 g, 5.81 mmol, 1 eq) and 2,4,6-triisopropylbenzoic acid (1.66 g, 6.68 mmol, 1.15 eq) in anhydrous THF (8 mL) at 0 °C under an atmosphere of N_2, was added DIAD (1.26 mL, 6.39 mmol, 1.1 eq) dropwise. The reaction mixture was stirred at rt for 4 h, after which the volatiles were removed *in vacuo*. The remaining residue was dissolved in hexane, and the resulting suspension was filtered, and the filter cake washed with hexane. The filtrate was concentrated and the crude product was purified by flash column chromatography (1:10 Et_2O:hexane) to obtain 2.06 g (88%) of **8** as a white solid.

R*f*: 0.42 (1:10 Et_2O:hexane)

FTIR (ν_{max}/cm^{-1}, neat): 2961, 2930, 2867, 1716, 1606, 1460, 1248, 1082, 746

^{1}H NMR (400 MHz, $CDCl_3$) δ: 7.85-7.76 (m, Naph, 3H), 7.72 (s, 1H, H_{10}), 7.50-7.39 (m, 3H, Naph), 6.97 (s, 2H, H_{18}), 4.68 (t, J = 7.1 Hz, 2H, H_{12}), 3.24 (t, J = 7.1 Hz, 2H, H_{11}), 2.87 (sept, J = 6.7 Hz, 1H, H_{23}), 2.74 (t, J = 6.7 Hz, 2H, H_{20}), 2.13 (d, J = 6.7 Hz, 6H, H_{24}, H_{25}), 1.14 (d, J = 6.7 Hz, 12H, H_{21}, H_{22})

^{13}C NMR (101 MHz, $CDCl_3$) δ: 171.0 (C_{14}), 150.2 (C_{19}), 144.9 (C_{17}), 135.2, 133.7, 132.5, 130.6 (C_{15}), 128.3, 127.7, 127.6, 127.4 (C_{10}), 127.2, 126.2, 125.6, 120.9 (C_{18}), 65.1 (C_{12}), 35.3 (C_{11}), 34.5 (C_{23}), 31.6 (C_{20}), 24.2 (C_{21}, C_{22}), 24.1 (C_{25}, C_{24}).

HRMS: (ESI) calcd. for $C_{28}H_{34}O_2Na$ (M+Na$^+$): 425.2451; Found 425.2446

3-(4-chlorophenyl)propyl 2,4,6-triisopropylbenzoate, 9

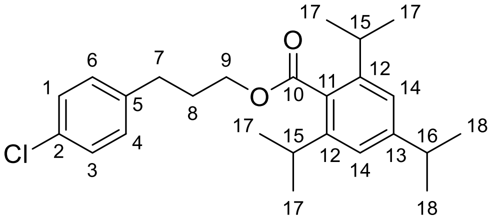

To a stirred solution of PPh_3 (4.7 g, 18.0 mmol, 1.2 eq), 3-(4-chlorophenyl)propan-1-ol (2.5 g, 15.0 mmol, 1.0 eq) and 2,4,6-triisopropylbenzoic acid (4.4 g, 18.0 mmol, 1.2 eq) in anhydrous THF (16 mL) at 0 °C under an atmosphere of N_2, was added DIAD (3.6 g, 18.0 mmol, 1.2 eq) dropwise. The reaction mixture was stirred at rt for 4 h, after which the volatiles were removed *in vacuo*. The remaining residue was dissolved in hexane, and the resulting suspension was filtered, and the filter cake washed with hexane. The filtrate was concentrated and the crude product was purified by flash column chromatography (1:10 Et_2O:hexane) to obtain 5.2 g (86%) of **9** as a colourless oil.

R_f: 0.60 (1:9 Et_2O:hexane)

IR (ν_{max}/cm^{-1}, neat): 2976, 2860, 1732, 1382, 1119, 1077, 845, 678.

^{1}H NMR (500 MHz, $CDCl_3$) δ: 7.33-7.27 (m, 2H, $H_{1,3}$), 7.19-7.15 (m, 2H, $H_{4,6}$), 7.08 (s, 2H, H_{14}), 4.37 (t, J = 6.5 Hz, 2H, H_9), 3.00-2.87 (m, 3H, $H_{15,16}$), 2.80- 2.74 (m, 2H, H_7), 2.12- 2.04 (m, 2H, H_8), 1.32 (d, J = 7.0 Hz, 12H, H_{17}), 1.31 (d, J = 7.0 Hz, 6H, H_{18})

^{13}C NMR (126 MHz, $CDCl_3$) δ: 170.9 (C_{10}), 150.2 (C_{13}), 144.8 (C_{12}), 139. 6 (C_5), 131. 9 (C_2), 130.5 (C_{11}), 129.8 ($C_{4,6}$), 128.6 ($C_{1,3}$), 120.9 (C_{14}), 64.1 (C_9), 34.5 (C_{16}), 31.7 (C_{15}), 31.6 (C_7), 30.3 (C_8), 24.2 (C_{17}), 24.0 (C_{18})

HRMS: (ESI) calcd. for $C_{25}H_{33}ClNaO_2$ ($M+Na^+$): 423.2067; Found 423.2089.

Phenethyl 2,4,6-triisopropylbenzoate, 10

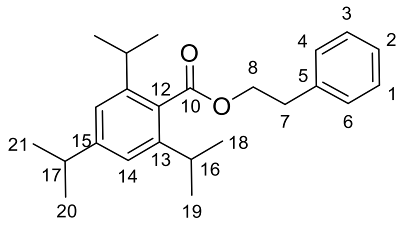

To a stirred solution of PPh_3 (2.89 g, 11 mmol, 1.1 eq), 2-phenethyl alcohol (1.22 g, 10 mmol, 1 eq) and 2,4,6-triisopropylbenzoic acid (2.86 g, 11.5 mmol, 1.15 eq) in anhydrous THF (15 mL) at rt under an atmosphere of N_2, was added DIAD (2.17 mL, 11 mmol, 1.1 eq) dropwise. The reaction mixture was stirred at 0 °C for 4 h, after which the volatiles were removed *in vacuo*. The remaining residue was dissolved in hexane, and the resulting suspension was filtered, and the filter cake washed with hexane. The filtrate was concentrated and the crude product was purified by flash column chromatography (1:10 Et_2O:hexane) to obtain 3.52 g (93%) of **10** as a white solid.

Spectral data were in accordance with the published values.[8]

^{1}H NMR (400 MHz, $CDCl_3$) δ: 7.37-7.20 (m, 5H, $C_{1,2,3,4,6}$), 7.00 (s, 2H, H_{14}), 4.59 (t, J = 7.1 Hz, 2H, H_8), 3.09 (t, J = 7.1 Hz, 2H, H_7), 2.91 (sept, J = 6.8 Hz, 1H, H_{17}), 2.80 (t, J = 6.8 Hz, 2H, H_{16}), 1.27 (d, J = 6.8 Hz, 6H, H_{20}, H_{21}), 1.21 (d, J = 6.8 Hz, 12H, H_{18}, H_{19})

^{13}C NMR (101 MHz, $CDCl_3$) δ: 171.0 (C_{10}), 150.2 (C_{15}), 144.9 (C_{13}), 137.8 (C_5), 130.6 (C_{12}), 128.9 ($C_{1,3}$), 128.6 ($C_{4,6}$), 126.7 (C_2), 120.9 (C_{14}), 65.3 (C_8), 35.1 (C_7), 34.6 (C_{17}), 31.6 (C_{16}), 24.2 (C_{18}, C_{19}), 24.1 (C_{20}, C_{21})

2-Bromo-5-(methoxymethoxy)benzaldehyde, 20

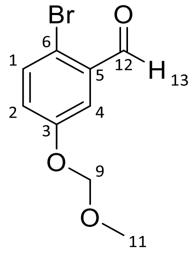

Following a procedure previously reported by Back *et. al.*[9] 2-bromo-5-hydroxybenzaldehyde (5.00 g, 24.9 mmol, 1.0 eq) was dissolved in 83 mL of anhydrous DCM, and the solution was cooled to 0 °C. Diisopropylethylamine (6.49 mL, 37.3 mmol, 1.5 eq) was added, followed by methoxymethyl chloride (2.27 mL, 29.9 mmol, 1.2 eq). The solution was stirred at rt for 24 h and quenched by addition of water (100 mL). The solution was extracted with DCM (3 x 50 mL). The ether extracts were combined, washed with brine (100 mL), dried ($MgSO_4$), and concentrated to afford a brown oil. The crude product was purified by flash column chromatography (2:1 hexane:Et_2O) to afford 5.91 g (96%) of 2-bromo-5-(methoxymethoxy)benzaldehyde **20** as a colourless oil.

Spectral data were in accordance with the published values[9]

^{1}H NMR (400 MHz, CDCl3) δ: 10.29 (s, 1H, H_{13}), 7.56 (d, J = 3.1 Hz, 1H, H_4), 7.53 (d, J = 8.8 Hz, 1H, H_1), 7.14 (dd, J = 8.8, 3.1 Hz, 1H, H_2), 5.19 (s, 2H, H_9), 3.46 (s, 3H, H_{11})

^{13}C NMR (101 MHz, CDCl3) δ: 191.7 (C_{12}), 157.0 (C_3), 134.8 (C_1), 134.3 (C_5), 124.2 (C_2), 118.9 (C_6), 116.6 (C_4), 94.6 (C_9), 56.4 (C_{11})

1-Bromo-4-(methoxymethoxy)-2-(3-methylbut-1-en-1-yl)benzene, 21

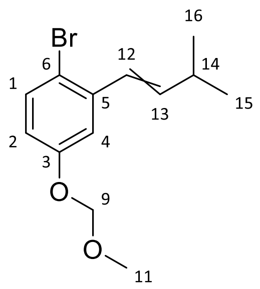

Following a procedure previously reported by Spencer *et. al.*[10] *n*-BuLi (1.6 M in hexanes, 14.61 mL, 23.4 mmol, 1.2 eq) was added to a solution of *iso*butyltriphenylphosphonium bromide (8.97 g, 22.4 mmol, 1.1 eq) in anhydrous THF (125 mL) at 0 °C dropwise. The reaction mixture became dark red in colour. The reaction mixture was maintained at 0 °C and **20** (5.00 g, 20.3 mmol, 1.0 eq) was added. The solution was warmed to rt over 1 h after which the solution had turned dark green. The reaction mixture was diluted with hexane (45 mL) and filtered through a silica plug. The solvent was removed *in vacuo* to afford the product as a pale yellow oil. The crude product was purified by flash column chromatography (3:1 hexane:EtOAc) to afford 5.79 g (93%) of 1-bromo-4-(methoxymethoxy)-2-(3-methylbut-1-en-1-yl)benzene **21** as a colourless oil (*E:Z* 1.09:1.0).

R_f: 0.51 (3:1 hexane:EtOAc)

FTIR (ν_{max}/cm^{-1}, neat): 2958, 2930, 2867, 1647, 1462, 1151, 1001

^{1}H NMR (400 MHz, $CDCl_3$) δ: 7.45 (d, J = 8.7 Hz, 1H, H_1), 7.41 (d, J = 8.7 Hz, 1H, H_1), 7.17 (d, J = 3.0 Hz, 1H, H_4), 6.99 (d, J = 3.0 Hz, 1H, H_4), 6.82 (dd, J = 8.7, 3.0 Hz, 1H, H_2), 6.78 (dd, J = 8.7, 3.0 Hz, 1H, H_2) 6.62 (dd, J = 15.9, 1.3 Hz, 1H, H_{12}), 6.27 (d, J = 11.5 Hz, 1H, H_{12}), 6.13 (dd J = 15.9, 6.9, 1H, H_{13}), 5.56 (dd J = 11.5, 10.5, 1H, H_{13}), 5.16 (s, 2H, H_9), 5.15 (s, 2H, H_9), 3.48 (s, 3H, H_{11}), 3.48 (s, 3H, H_{11}), 2.74-2.62 (m, 1H, H_{14}), 2.51 (m, 1H, H_{14}), 1.11 (d, J = 6.8 Hz, 6H, H_{16} and H_{15}), 1.02 (d, J = 6.8 Hz, 6H, H_{16} and H_{15})

^{13}C NMR (101 MHz, $CDCl_3$) δ: 156.7 (C_3), 156.3 (C_3), 141.5 (C_{13}), 141.4 (C_{13}), 139.0 (C_5), 138.7 (C_5), 133.5 (C_1), 133.1 (C_1), 126.1 (C_{12}), 125.9 (C_{12}), 118.6 (C_4), 116.6 (C_2), 116.4 (C_2), 116.00 (C_6), 115.6 (C_6), 114.5 (C_4), 94.8 (C_9), 94.7 (C_9), 56.2 (C_{11}), 56.1 (C_{11}), 31.8 (C_{14}), 27.5 (C_{14}), 23.1 (C_{15}, C_{16}), 22.5 (C_{15}, C_{16})

HRMS: (ESI) calcd. for $C_{13}H_{17}BrO_2$ (M+): 307.0304; Found 307.0309

2-(4-(methoxymethoxy)-2-(3-methylbut-1-en-1-yl)phenyl)-4,4,5,5-tetramethyl-1,3,2-dioxaborolane, 22

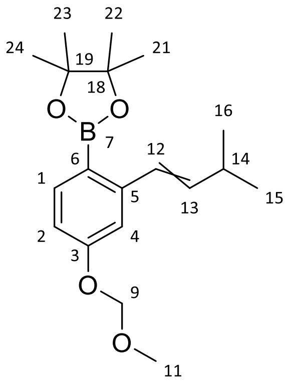

Following a procedure previously reported by Spencer *et. al.*[10] *n*-BuLi (1.6 M in hexanes, 13.13 mL, 21.0 mmol, 1.2 eq) was added to a solution of **21** (5.00 g, 17.5 mmol, 1.0 eq) in 55 mL of anhydrous THF at - 78 °C. The mixture was stirred for 40 minutes after which the solution had turned dark green. 2-Isopropoxy-4,4,5,5-tetramethyl-1,3,2-dioxaborolane (4.30 mL, 21.0 mmol, 1.2 eq) was added dropwise to the reaction mixture, maintaining the temperature at -78 °C. The reaction mixture turned blue on addition and after 10 minutes the reaction was warmed to rt. Once the solution was at rt a yellow solution was obtained. The reaction was quenched with NH_4Cl (50 mL) and extracted with diethyl ether (3 x 50 mL). The combined organic layers were dried ($MgSO_4$) and concentrated to give a yellow oil. The crude product was purified by flash column chromatography (1:5 EtOAc:hexane) to obtain 5.81 g (95%) of 2-(4-(methoxymethoxy)-2-(3-methylbut-1-en-1-yl)phenyl)-4,4,5,5-tetramethyl-1,3,2-dioxaborolane **22** as a colourless oil.

R_f: 0.35 (1:5 EtOAc:hexane)

FTIR (ν_{max}/cm^{-1}, neat): 2959, 2931, 2868, 1596, 1343, 1144, 1007

^{1}H NMR (500 MHz, $CDCl_3$) δ: 7.74 (d, *J* = 8.4 Hz, 1H, H_1), 7.71 (d, *J* = 8.4 Hz, 1H, H_1), 7.21 (d, *J* = 2.5 Hz, 1H, H_4), 7.19 (dd, *J* = 16.0, 1.6 Hz, 1H, H_{12}), 6.97 (d, *J* = 2.5 Hz, 1H, H_4), 6.89 (dd, *J* = 8.4, 2.5 Hz, 1H, H_2) 6.87 (dd, *J* = 8.4, 2.5 Hz, 1H, H_2), 6.78 (d, *J* = 11.7 Hz, 1H, H_{12}), 6.15 (dd *J* = 15.7, 6.3, 1H, H_{13}), 5.43 (dd *J* = 11.7, 10.3, 1H, H_{13}), 5.21 (s, 2H, H_9), 5.19 (s, 2H, H_9), 3.47 (s, 3H, H_{11}), 3.47 (s, 3H, H_{11}), 2.80-2.68 (m, 1H, H_{14}), 2.49 (m, 1H, H_{14}), 1.34 (s, 12H, C_{21-24}), 1.31 (s, 12H, C_{21-24}), 1.11 (d, *J* = 6.8 Hz, 6H, H_{16} and H_{15}), 1.01 (d, *J* = 6.8 Hz, 6H, H_{16} and H_{15})

^{13}C NMR (126 MHz, $CDCl_3$) δ: 159.4 (C_3), 158.9 (C_3), 146.4 (C_5), 146.1 (C_5), 139.2 (C_{13}), 138.7 (C_{13}), 137.7 (C_1), 137.4 (C_1), 127.9 (C_{12}), 127.5 (C_4), 116.6 (C_4), 113.7 (C_2), 113.3 (C_2), 111.8 (C_{12}), 94.2 (C_9), 94.0 (C_9), 83.3 (C_{19}), 83.3 (C_{18}), 56.0 (C_{11}), 55.9 (C_{11}), 31.3 (C_{14}), 27.0 (C_{14}), 24.9 (C_{21-24}), 24.8 (C_{21-24}) 23.2 (C_{15}, C_{16}), 22.2 (C_{15}, C_{16})

HRMS: (ESI) calcd. for $C_{19}H_{29}{}^{11}BO_4Na$ ($M+Na^+$): 355.2051; Found 355.2038

2-(2-isopentyl-4-(methoxymethoxy)phenyl)-4,4,5,5-tetramethyl-1,3,2-dioxaborolane, 6

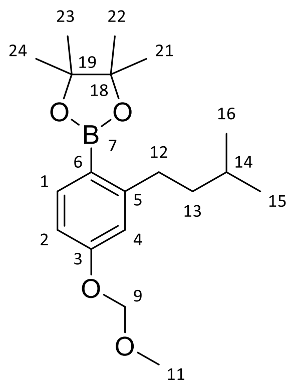

To a solution of Pd/C (10 mol%, 0.005 eq) in EtOH, was added 2-(4-(methoxymethoxy)-2-(3-methylbut-1-en-1-yl)phenyl)-4,4,5,5-tetramethyl-1,3,2-dioxaborolane **22** (2.0 g, 6.0 mmol, 1.0 eq). The reaction was stirred at rt and a balloon of H_2 was secured to a T-connector adaptor that was attached to the reaction vessel. The reaction vessel was evacuated and filled with N_2 (3x) using the T-connector adaptor. This was then repeated, using H_2 in place of N_2. The reaction vessel was left open to H_2 and stirred for 2 h at rt. The reaction mixture was passed through a pad of celite, taking care to not allow it to run dry. The crude NMR revealed complete conversion and the material was used directly in the next step without further purification. 2-(2-isopentyl-4-(methoxymethoxy)phenyl)-4,4,5,5-tetramethyl-1,3,2-dioxaborolane, **6**, was obtained in a 98% yield (1.97 g) as a colourless oil.

R*f*: 0.41 (1:4 Et_2O:hexane)

FTIR (ν_{max}/cm^{-1}, neat): 2954, 2931, 2869, 1600, 1345, 1145, 1006

^{1}H NMR (400 MHz, $CDCl_3$) δ: 7.73 (d, J = 8.9 Hz, 1H, H_1), 6.86-6.82 (m, 2H, H_2 and H_4), 5.19 (s, 2H, H_9), 3.47 (s, 3H, H_{11}), 2.86 (m, 2H, H_{12}), 1.65 (sept, J = 6.6 Hz, 1H, H_{14}), 1.45 (m, 2H, H_{13}), 1.33 (s, 12H, C_{21-24}), 0.95 (d, J = 6.6 Hz, 6H, H_{16} and H_{15})

^{13}C NMR (101 MHz, $CDCl_3$) δ: 159.5 (C_3), 153.0 (C_5), 138.1 (C_1), 116.9 (C_4), 112.4 (C_2), 94.1 (C_9), 83.2 (C_{18}, C_{19}), 56.1 (C_{11}), 42.8 (C_{13}), 34.2 (C_{12}), 28.6 (C_{14}), 24.9 (C_{21-24}), 22.7 (C_{15}, C_{16})

HRMS: (ESI) calcd. for $C_{19}H_{31}{}^{11}BO_4Na$ (M+Na$^+$): 357.2208; Found 357.2205

(R)-2-(1-(2-isopentyl-4-(methoxymethoxy)phenyl)ethyl)-4,4,5,5-tetramethyl-1,3,2-dioxaborolane, 23

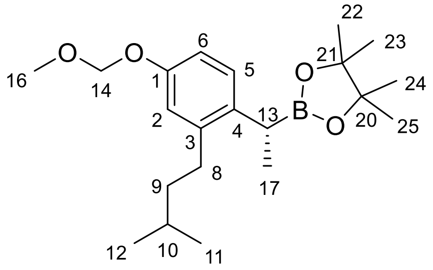

Synthesised according to GP1. Stannane **(*S*)-7** (356 mg, 0.81 mmol, 1.35 eq), *n*-BuLi (1.58 M in hexanes, 0.49 mL, 0.78 mmol, 1.30 eq) and boronic ester **6** (200 mg, 0.6 mmol, 1 eq). Lithiation time of 1 h, borylation time of 30 min, 1,2-metallate rearrangement time of 2 h. The crude boronic ester **23** was not isolated and was used as the crude material in the subsequent homologation. The conversion was monitored by GCMS.

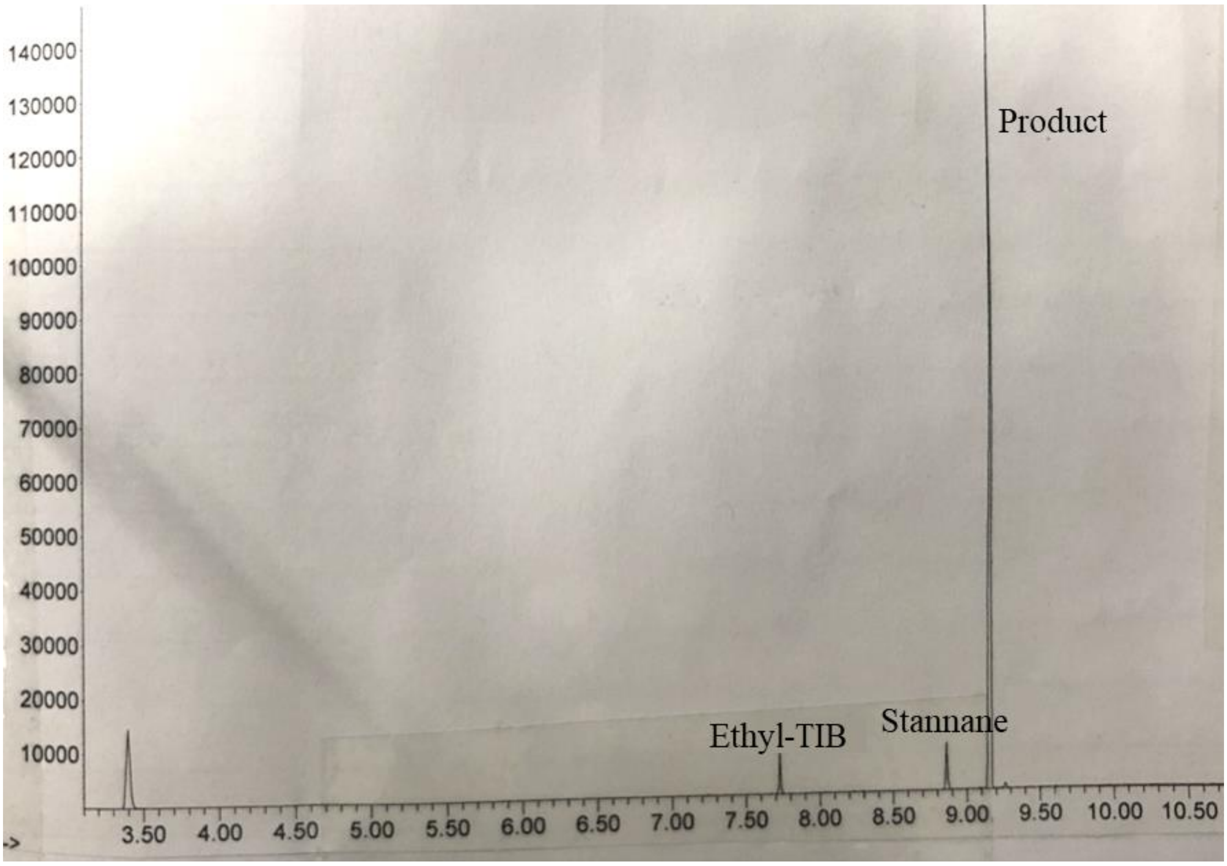

Figure 20. A labelled GCMS trace after the first homologation of boronic ester **6**

2-((2R,3S)-3-(2-isopentyl-4-(methoxymethoxy)phenyl)butan-2-yl)-4,4,5,5-tetramethyl-1,3,2-dioxaborolane, 24

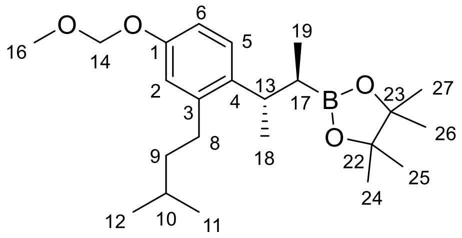

Synthesised according to GP1. Stannane **(*S*)-7** (356 mg, 0.81 mmol, 1.35 eq), *n*-BuLi (1.58 M in hexanes, 0.49 mL, 0.78 mmol, 1.30 eq) and boronic ester **23** (217 mg, 0.6 mmol, 1 eq). Lithiation time of 1 h, borylation time of 30 min, 1,2-metallate rearrangement time of 2 h. The crude boronic ester was purified by flash column chromatography (1:10 Et_2O:hexane) to give boronic ester **24** (195 mg, 83% over the two homologations, *d.r.* >99:1 by GCMS) as a colourless oil.

R_f: 0.27 (1:10 Et_2O:hexane)

$[\alpha]^{20}_D$: +16 (*c* 1.0, $CHCl_3$)

FTIR (ν_{max}/cm^{-1}, neat): 2955, 2934, 2870, 1608, 1378, 1315, 1144, 1014

^{1}H NMR (500 MHz, $CDCl_3$) δ: 7.12 (d, J = 8.5 Hz, 1H, H_5), 6.86 (dd, J = 8.5, 2.8 Hz, 1H, H_6), 6.79 (d, J = 2.8 Hz, 1H, H_2), 5.15 (s, 2H, H_{14}), 3.49 (s, 3H, H_{16}), 2.95 (dsept, J = 10.6, 6.9 Hz, 1H, H_{13}), 2.67 (ddd, J = 13.7, 9.3, 7.4 Hz, 1H, H_8), 2.55 (ddd, J = 13.7, 9.3, 7.4 Hz, 1H, H_8), 1.65 (sept, J = 6.7 Hz, 1H, H_{10}), 1.48-1.42 (m, 2H, H_9), 1.28 (s, 12H, H_{24-27}), 1.30-1.26 (m, 1H, H_{17}), 1.19 (d, J = 6.9 Hz, 3H, H_{18}), 0.97 (d, J = 6.7 Hz, 6H, $H_{11,12}$), 0.76 (d, J = 7.4 Hz, 3H, H_{19})

^{13}C NMR (126 MHz, $CDCl_3$) δ: 154.9 (C_1), 142.3 (C_3), 138.9 (C_4), 126.8 (C_5), 116.8 (C_2), 113.5 (C_6), 94.7 (C_{14}), 83.0 (C_{22}, C_{23}), 56.0 (C_{16}), 40.9 (C_9), 36.1 (C_{13}), 31.0 (C_8), 28.2 (C_{10}), 25.0 (C_{24-27}), 24.8 (C_{24-27}), 23.4 (C_{18}), 22.7 ($C_{11/12}$), 22.7 ($C_{11/12}$), 14.6 (C_{19}).

HRMS: (ESI) calcd. for $C_{23}H_{39}{}^{11}BO_4Na$ ($M+Na^+$): 413.2838; Found 413.2820

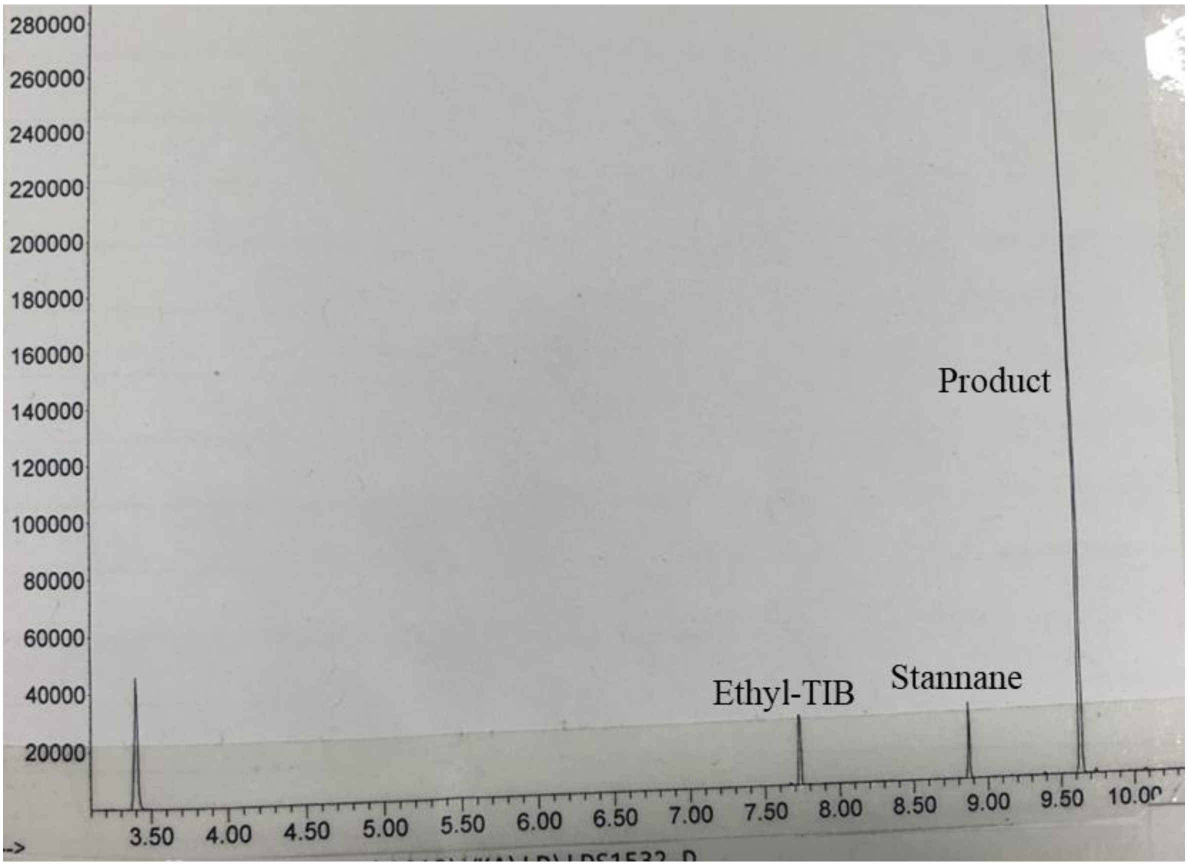

Figure 21. A labelled GCMS trace of the second homologation

2-((2S,3S,4S)-4-(2-isopentyl-4-(methoxymethoxy)phenyl)-3-methyl-1-(naphthalen-2-yl)pentan-2-yl)-4,4,5,5-tetramethyl-1,3,2-dioxaborolane, 25

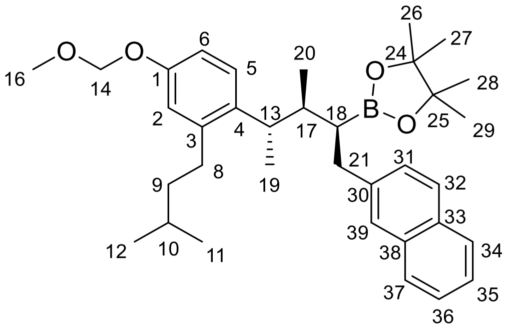

Synthesised according to GP2. Benzoate **8** (288 mg, 0.74 mmol, 1.35 eq), *s*-BuLi (1.3 M in hexanes, 0.57 mL, 0.72 mmol, 1.30 eq), (+)-sparteine (0.17 mL, 0.72 mmol, 1.30 eq) and boronic ester **24** (215 mg, 0.55 mmol, 1 eq). Lithiation time of 1 h, borylation time of 2 h 30 min, 1,2-metallate rearrangement time of 2 h. The crude boronic ester was purified by flash column chromatography (1:10 Et_2O:hexane) to give boronic ester **25** (203 mg, 72%, *d.r.* >95:5 by NMR) as a colourless oil.

R_f: 0.31 (1:10 Et_2O:hexane)

$[\alpha]^{20}_D$: +24 (*c* 1.0, $CHCl_3$)

FTIR (ν_{max}/cm^{-1}, neat): 2957, 2931, 2870, 1606, 1496, 1371, 1321, 1145, 1009

^{1}H NMR (500 MHz, $CDCl_3$) δ: 7.87-7.77 (m, 4H, Naph), 7.54-7.40 (m, 3H, Naph), 7.23 (d, J = 8.5 Hz, 1H, H_5), 6.93 (dd, J = 8.5, 2.9 Hz, 1H, H_6), 6.87 (d, J = 2.9 Hz, 1H, H_2), 5.20 (s, 2H, H_{14}), 3.53 (s, 3H, H_{16}), 3.13 (dq, J = 10.6, 6.7 Hz, 1H, H_{13}), 3.00 (dd, J = 13.9, 11.5 Hz, 1H, H_{21}), 2.94 (dd, J = 13.9, 3.9 Hz, 1H, H_{21}), 2.76 (ddd, J = 13.6, 9.9, 6.5 Hz, 1H, H_8), 2.61 (ddd, J = 13.6, 9.9, 6.5 Hz, 1H, H_8), 2.08 (dqd, J = 9.0, 6.8, 2.8 Hz, 1H, H_{17}), 2.00 (ddd, J = 11.5, 3.9, 2.8 Hz, 1H, H_{18}), 1.69 (sept, J = 6.6 Hz, 1H, H_{10}), 1.56-1.47 (m, 2H, H_9), 1.38 (d, J = 6.7 Hz, 3H, H_{19}), 1.17 (s, 12H, H_{26-29}), 1.04 (d, J = 6.6 Hz, 3H, $H_{11/12}$), 1.02 (d, J = 6.6 Hz, 3H, $H_{11/12}$), 0.86 (d, J = 6.8 Hz, 3H, H_{20})

^{13}C NMR (126 MHz, $CDCl_3$) δ: 154.9 (C_1), 142.0 (C_3), 141.0 (C_{30}), 139.2 (C_4), 133.7 (C_{38}), 132.0 (C_{33}), 128.2 (C_{31}), 127.7, 127.6, 127.5, 127.3 (C_5), 127.1 (C_{39}), 125.8, 124.9, 117.0 (C_2), 114.1 (C_6), 94.7 (C_{14}), 83.2 ($C_{24,}$ C_{25}), 56.0 (C_{16}), 40.9 (C_9), 40.6 (C_{17}), 36.4 (C_{13}), 31.5 (C_8), 30.6 (C_{21}), 28.5 (C_{18}), 28.3 (C_{10}), 25.0 (C_{26-29}), 24.6 (C_{26-29}), 22.8 ($C_{11/12}$), 22.7 ($C_{11/12}$), 21.5 (C_{19}), 16.5 (C_{20})

HRMS: (ESI) calcd. for $C_{35}H_{49}{}^{11}BO_4Na$ ($M+Na^+$): 567.3622; Found 567.3628

2-((2S,3R,4S,5S)-5-(2-isopentyl-4-(methoxymethoxy)phenyl)-4-methyl-3-(naphthalen-2-ylmethyl)hexan-2-yl)-4,4,5,5-tetramethyl-1,3,2-dioxaborolane, 26

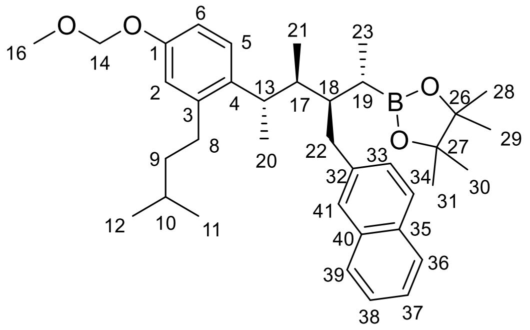

Synthesised according to GP1. Stannane ***(R)*-7** (221 mg, 0.5 mmol, 1.35 eq), *n*-BuLi (1.58 M in hexanes, 0.31 mL, 0.48 mmol, 1.30 eq) and boronic ester **25** (203 mg, 0.37 mmol, 1 eq). Lithiation time of 1 h, borylation time of 3 h, 1,2- metallate rearrangement time of 2 h. The crude boronic ester was purified by flash column chromatography (1:10 Et_2O:hexane) to give boronic ester **26** (188 mg, 89%, *d.r.* >95:5 by NMR) as a colourless oil.

R_f: 0.17 (1:10 Et_2O:hexane)

$[\alpha]^{20}_D$: +51 (*c* 1.0, $CHCl_3$)

FTIR (ν_{max}/cm^{-1}, neat): 2956, 2870, 1606, 1497, 1463, 1370, 1313, 1144, 1013

^{1}H NMR (500 MHz, $CDCl_3$) δ: 7.83-7.73 (m, 4H, $C_{34, 36, 39, 41}$), 7.51 (dd, *J* = 8.4, 1.4 Hz, 1H, C_{33}), 7.45 (td, *J* = 7.9, 1.4 Hz, 1H, C_{38}), 7.41 (td, *J* = 7.6, 1.4 Hz, 1H, C_{37}), 7.06 (d, *J* = 8.6 Hz, 1H, H_5), 6.84 (dd, *J* = 8.6, 2.3 Hz, 1H, H_6), 6.74 (d, *J* = 2.3 Hz, 1H, H_2), 5.14 (s, 2H, H_{14}), 3.48 (s, 3H, H_{16}), 2.88 (dd, *J* = 14.3, 6.5 Hz, 1H, H_{22}), 2.76-2.66 (m, 2H, H_{22}, H_{13}), 2.56 (br. dddd *J* = 9.6, 6.8, 6.5, 1.9 Hz, 1H, H_{18}), 2.46 (ddd, *J* = 14.1, 9.6, 6.4 Hz, 1H, H_8), 2.36 (ddd, *J* = 14.1, 9.6, 6.4 Hz, 1H, H_8), 1.96 (br. m, 1H, H_{17}), 1.39 (sept, *J* = 6.8 Hz, 1H, H_{10}), 1.25-1.18 (m, 3H, H_{19}, H_9), 1.18 (s, 6H, H_{28-31}), 1.17 (s, 6H, H_{28-31}), 1.06 (br. d, *J* = 6.9 Hz, 6H, H_{20}, H_{23}), 0.84 (d, *J* = 6.8 Hz, 6H, H_{11}, H_{12}), 0.67 (d, *J* = 6.8 Hz, 3H, H_{21})

^{13}C NMR (126 MHz, $CDCl_3$) δ: 154.6 (C_1), 141.9 (C_3), 140.3 (C_{32}), 139.9 (C_4), 133.8 (C_{40}), 132.2 (C_{35}), 128.2, 127.8 (C_{33}), 127.7, 127.6, 127.4, 127.1 (C_5), 125.9 (C_{38}), 125.1 (C_{37}), 116.7 (C_2), 113.8 (C_6), 94.8 (C_{14}), 83.0 (C_{26}, C_{27}), 56.1 (C_{16}), 41.7 (C_{17}), 41.4 (C_{18}), 40.5 (C_9), 36.5 (C_{13}), 36.1 (C_{22}), 31.4 (C_9), 27.8 (C_{10}), 25.0 (C_{28-31}), 24.9 (C_{28-31}), 22.8 ($C_{11/12}$), 22.7 ($C_{11/12}$), 21.6 (C_{20}), 21.6 (C_{19}), 13.6 (C_{23}), 13.0 (C_{21})

HRMS: (ESI) calcd. for $C_{37}H_{53}{}^{11}BO_4Na$ (M+Na^+): 595.3936; Found 595.3892

2-((2R,3R,4R,5S,6S)-6-(2-isopentyl-4-(methoxymethoxy)phenyl)-3,5-dimethyl-4-(naphthalen-2-ylmethyl)heptan-2-yl)-4,4,5,5-tetramethyl-1,3,2-dioxaborolane, 27

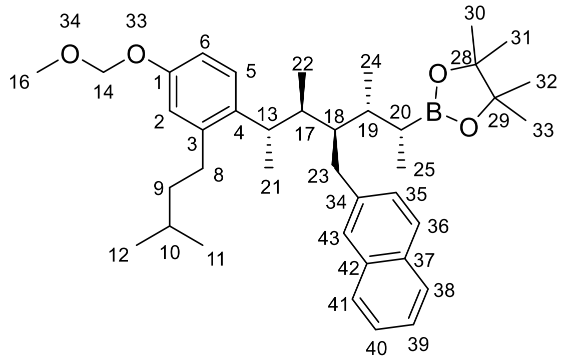

Synthesised according to GP1. Stannane **(*S*)-7** (196 mg, 0.45 mmol, 1.35 eq), *n*-BuLi (1.58 M in hexanes, 0.27 mL, 0.43 mmol, 1.30 eq) and boronic ester **26** (188 mg, 0.33 mmol, 1 eq). Lithiation time of 1 h, borylation time of 3 h, 1,2- metallate rearrangement of 2 h. The crude boronic ester was purified by flash column chromatography (1:10 Et_2O:hexane) to give boronic ester **27** (170 mg, 86%, *d.r.* >95:5 by NMR) as a colourless oil.

R*f*: 0.27 (1:10 Et_2O:hexane)

$[\alpha]^{20}_D$: +35 (*c* 1.0, $CHCl_3$)

FTIR (ν_{max}/cm^{-1}, neat): 2957, 2872, 1606, 1497, 1461, 1369, 1311, 1147, 1013

^{1}H NMR (500 MHz, $CDCl_3$) *δ*: 7.83-7.76 (m, 3H, Naph), 7.69 (s, 1H, H_{43}), 7.48-7.39 (m, 3H, Naph), 7.12 (d, *J* = 8.0 Hz, 1H, H_5), 6.85 (dd, *J* = 8.0, 2.3 Hz, 1H, H_6), 6.75 (d, *J* = 2.3 Hz, 1H, H_2), 5.15 (s, 2H, H_{14}), 3.49 (s, 3H, H_{16}), 2.93 (dd, *J* = 14.3, 5.1 Hz, 1H, H_{23}), 2.81-2.72 (m, 2H, H_{23}, H_{13}), 2.54-2.45 (br. m, 1H, H_8), 2.43-2.34 (br. m, 1H, H_8), 2.31 (br. dddd, 7.8, 6.6, 5.1, 2.5 Hz, 1H, H_{18}), 1.98 (br. dqd, *J* = 9.5, 6.7, 2.5 Hz, 1H, H_{17}), 1.87 (sept, *J* = 6.6 Hz, 1H, H_{19}), 1.40 (sept, *J* = 6.5 Hz, 1H, H_{10}), 1.34 (m, 1H, H_{20}), 1.24 (m, 2H, H_9), 1.18 (s, 12H, H_{30-33}), 1.15 (d, *J* = 6.8 Hz, 3H, H_{21}), 0.97 (d, *J* = 7.1 Hz, 3H, H_{24}), 0.87 (d, *J* = 7.5 Hz, 3H, H_{25}), 0.85 (d, *J* = 6.6 Hz, 6H, H_{11}, H_{12}), 0.68 (d, *J* = 6.7 Hz, 3H, H_{22})

^{13}C NMR (126 MHz, $CDCl_3$) *δ*: 154.6 (C_1), 141.8 (C_3), 140.7 (C_{34}), 139.6 (C_4), 133.7 (C_{26}), 131.9 (C_{27}), 127.9, 127.7, 127.6, 127.4, 127.1 (C_5), 127.1 (C_{43}), 125.7 (C_{40}), 124.9 (C_{39}), 116.6 (C_2), 113.9 (C_6), 94.6 (C_{14}), 82.7 (C_{28}, C_{29}), 55.9 (C_{16}), 42.6 (C_{18}), 40.4 (C_9), 39.7 (C_{17}), 36.7 (C_{13}), 36.5 (C_{19}), 35.0 (C_{23}), 31.3 (C_8), 27.8 (C_{10}), 24.8 (C_{30-33}), 24.8 (C_{30-33}), 22.6 (C_{11}, C_{12}), 21.8 (C_{21}), 20.4 (C_{20}), 15.3 (C_{24}), 13.9 (C_{22}), 10.9 (C_{25})

HRMS: (ESI) calcd. for $C_{39}H_{57}{}^{11}BO_4Na$ (M+Na^+): 623.4249; Found 623.4239

2-((2R,3S,4R,5R,6S,7S)-7-(2-isopentyl-4-(methoxymethoxy)phenyl)-3,4,6-trimethyl-5-(naphthalen-2-ylmethyl)octan-2-yl)-4,4,5,5-tetramethyl-1,3,2-dioxaborolane, 28

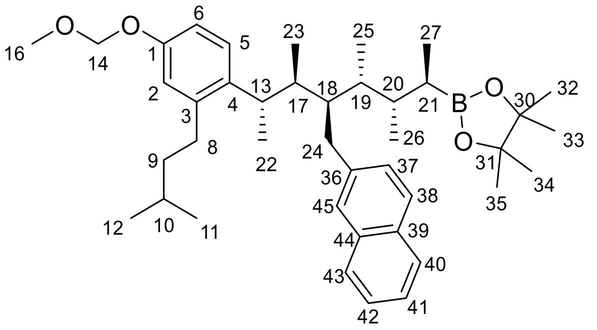

Synthesised according to GP1. Stannane **(*S*)-7** (186 mg, 0.38 mmol, 1.35 eq), *n*-BuLi (1.58 M in hexanes, 0.23 mL, 0.36 mmol, 1.30 eq) and boronic ester **27** (170 mg, 0.28 mmol, 1 eq). Lithiation time of 1 h, borylation time of 3 h, 1,2- metallate rearrangement time of 2 h. The crude boronic ester was purified by flash column chromatography (1:10 Et_2O:hexane) to give boronic ester **28** (133 mg, 76%, *d.r.* >95:5 by NMR) as a colourless oil.

R_f: 0.33 (1:10 Et_2O:hexane)

$[\alpha]^{20}_D$: +38 (*c* 1.0, $CHCl_3$)

FTIR (ν_{max}/cm^{-1}, neat): 2958, 2871, 1606, 1497, 1463, 1371, 1312, 1146, 1016

^{1}H NMR (500 MHz, $CDCl_3$) *δ*: 7.83-7.75 (m, 3H, Naph), 7.70 (s, 1H, H_{45}), 7.47-7.38 (m, 3H, Naph), 7.08 (d, *J* = 8.4 Hz, 1H, H_5), 6.85 (dd, *J* = 8.4, 2.4 Hz, 1H, H_6), 6.75 (d, *J* = 2.4 Hz, 1H, H_2), 5.14 (s, 2H, H_{14}), 3.49 (s, 3H, H_{16}), 2.90 (dd, *J* = 15.0, 5.2 Hz, 1H, H_{24}), 2.81-2.72 (m, 2H, H_{24}, H_{13}), 2.52-2.44 (br. m, 1H, H_8), 2.41-2.34 (br. m, 1H, H_8), 2.32 (br. dddd, 8.0, 6.9, 5.2, 2.5 Hz, 1H, H_{18}), 1.98 (br. dqd, *J* = 9.5, 6.9, 2.5 Hz, 1H, H_{17}), 1.79-1.69 (m, 2H, H_{19}, H_{20}), 1.39 (sept, *J* = 6.6 Hz, 1H, H_{10}), 1.31-1.19 (m, 3H, H_9, H_{21}), 1.13 (d, *J* = 6.6 Hz, 3H, H_{22}), 1.10 (s, 6H, $H_{32\text{-}35}$), 1.07 (s, 6H, $H_{32\text{-}35}$), 0.92 (d, *J* = 7.2 Hz, 3H, H_{26}), 0.85 (d, *J* = 6.9 Hz, 3H, H_{25}), 0.83 (d, *J* = 6.4 Hz, 6H, H_{11}, H_{12}), 0.74 (d, *J* = 6.6 Hz, 3H, H_{27}), 0.67 (d, *J* = 6.9 Hz, 3H, H_{23})

^{13}C NMR (126 MHz, $CDCl_3$) *δ*: 154.8 (C_1), 141.9 (C_3), 141.0 (C_{36}), 139.6 (C_4), 133.8 (C_{44}), 132.0 (C_{39}), 127.9, 127.8, 127.7, 127.6, 127.3, 127.2, 125.8 (C_{42}), 125.0 (C_{51}), 116.8 (C_2), 114.0 (C_6), 94.8 (C_{14}), 82.7 (C_{30}, C_{31}), 56.1 (C_{16}), 41.8 (C_{18}), 40.6 (C_9), 40.4 (C_{17}), 37.3 (C_{20}), 36.7 (C_{19}), 36.6 (C_{13}), 35.4 (C_{24}), 31.4 (C_8), 27.9 (C_{10}), 24.8 ($C_{32\text{-}35}$), 24.6 ($C_{32\text{-}35}$, C_{21}), 22.7 ($C_{11/12}$), 22.7 ($C_{11/12}$), 21.7 (C_{22}), 14.8 (C_{27}), 14.4 (C_{26}), 13.6 (C_{23}), 12.0 (C_{25})

HRMS: (ESI) calcd. for $C_{41}H_{61}{}^{11}BO_4Na$ ($M+Na^+$): 651.4562; Found 651.4556

2-((2S,3S,4S,5R,6R,7S,8S)-8-(2-isopentyl-4-(methoxymethoxy)phenyl)-3,4,5,7-tetramethyl-6-(naphthalen-2-ylmethyl)-1-phenylnonan-2-yl)-4,4,5,5-tetramethyl-1,3,2-dioxaborolane, 11

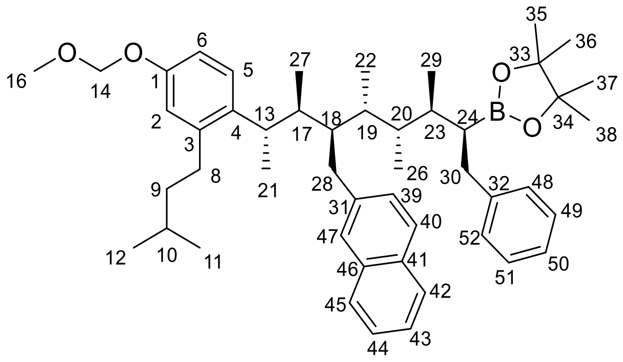

Synthesised according to GP2. Benzoate **10** (73 mg, 0.21 mmol, 1.35 eq), *s*-BuLi (1.3 M in hexanes, 0.15 mL, 0.2 mmol, 1.30 eq), (+)-sparteine (0.05 mL, 0.2 mmol, 1.30 eq) and boronic ester **28** (97 mg, 0.15 mmol, 1 eq). Lithiation time of 1 h, borylation time of 3 h, 1,2- metallate rearrangement time of 2 h. The crude boronic ester was purified by flash column chromatography (1:10 Et_2O:hexane) to give boronic ester **11** (92 mg, 84%, *d.r.* >95:5 by NMR) as a colourless oil.

R_f: 0.26 (1:10 Et_2O:hexane)

$[\alpha]^{20}_D$: +24 (*c* 1.0, $CHCl_3$)

FTIR (ν_{max}/cm^{-1}, neat): 2962, 2874, 1604, 1496, 1465, 1375, 1148, 1018

^{1}H NMR (500 MHz, $CDCl_3$) δ: 7.81-7.71 (m, 4H, Ar), 7.47-7.37 (m, 3H, Ar), 7.13-7.02 (m, 4H, Ar), 6.94 (d, *J* = 8.4 Hz, 2H, H_5, Ar), 6.86 (dd, *J* = 8.4, 2.2 Hz, 1H, H_6), 6.76 (d, *J* = 2.2 Hz, 1H, H_2), 5.14 (s, 2H, H_{14}), 3.48 (s, 3H, H_{16}), 3.01 (dd, *J* = 15.1, 3.6 Hz, 1H, H_{28}), 2.85 (dq, *J* = 10.8, 6.6 Hz, 1H, H_{13}), 2.67 (dd, *J* = 15.1, 6.8 Hz, 1H, H_{28}), 2.57-2.46 (m, H_8, 2H, H_{30}), 2.46-2.37 (br. m, 1H, H_8), 2.34 (br. dddd, 8.8, 6.8, 3.6, 2.0 Hz, 1H, H_{18}), 2.25 (dd, *J* = 13.3, 3.1 Hz, 1H, H_{30}), 2.03 (br. dqd, *J* = 10.8, 6.7, 2.2 Hz, 1H, H_{17}), 1.85-1.74 (m, 2H, H_{19}, H_{20}), 1.61 (br. dqd, *J* = 9.5, 6.7, 2.5 Hz, 1H, H_{23}), 1.42 (sept, *J* = 7.0 Hz, 1H, H_{10}), 1.38 (ddd, *J* = 12.0, 3.1, 2.5 Hz, 1H, H_{24}), 1.33-1.24 (m, 2H, H_9), 1.18 (d, *J* = 6.6 Hz, 3H, H_{21}), 1.10 (s, 6H, H_{35-38}), 1.09 (s, 6H, H_{35-38}), 0.96 (d, *J* = 6.7 Hz, 3H, H_{29}), 0.83 (m, 9H, H_{11}, H_{12}, H_{22}), 0.68 (d, *J* = 6.7 Hz, 3H, H_{27}), 0.66 (d, *J* = 6.8 Hz, 3H, H_{26}).

^{13}C NMR (126 MHz, $CDCl_3$) δ: 154.8 (C_1), 143.6 (C_{32}), 141.9 (C_3), 141.3 (C_{31}), 139.5 (C_4), 133.8 (C_{46}), 132.0 (C_{41}), 129.1 ($C_{48,52}$), 128.1, 127.9 ($C_{49,51}$), 127.7, 127.7, 127.6, 127.2, 127.0, 126.1, 125.2, 125.1, 116.9 (C_2), 114.1 (C_6), 94.8 (C_{14}), 83.0 (C_{33}, C_{34}), 56.1 (C_{16}), 42.5 (C_{18}), 40.9 (C_{17}), 40.7 (C_9), 36.9, 36.8, 36.2 (C_{13}), 36.0 (C_{28}), 31.5 (C_8), 30.3 (C_{30}), 28.6 (C_{24}), 28.0 (C_{10}), 24.9 (C_{35-38}), 24.7 (C_{35-38}), 22.7 (C_{11}, C_{12}), 21.8 (C_{21}), 16.1 (C_{29}), 12.9 (C_{27}), 12.8 (C_{26}), 12.1 (C_{22})

HRMS: (MALDI) calcd. for $C_{49}H_{69}{}^{11}BO_4Na$ (M+Na^+): 755.5189; Found 755.5180

4-((2S,3S,4R,5R,6R,7R,8S)-8-hydroxy-3,5,6,7-tetramethyl-4-(naphthalen-2-ylmethyl)-9-phenylnonan-2-yl)-3-isopentylphenol, 4

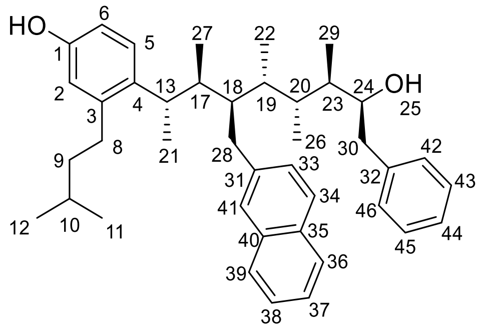

Boronic ester **11** (92 mg, 0.13 mmol) was taken up in MeOH and cooled to 0 °C. Concentrated HCl was added dropwise (5 drops) and the reaction mixture was stirred at 0 °C for 1 h then warmed to rt and stirred overnight. The reaction mixture was diluted with H_2O (5 mL) and Et_2O (5 mL). The phases were separated and the aqueous phase washes with Et_2O (3 x 5 mL). The combined organic phases were dried ($MgSO_4$) and concentrated under reduced pressure. The crude reaction mixture was taken up in THF (2 mL) and cooled to 0 °C. A premixed solution of NaOH (2M):H_2O_2 (30% aq) (2:1 3 mL) was added dropwise at 0 °C, warmed to room temperature and stirred for 4 h. The reaction mixture was diluted with H_2O (5 mL) and Et_2O (5 mL). The phases were separated and the aqueous phase washes with Et_2O (3 x 5 mL). The combined organic phases were washed with H_2O (5 mL), dried ($MgSO_4$) and concentrated under reduced pressure. The crude material was purified by flash column chromatography (7:2:1 hexane:Et_2O:EtOAc) to give **4** as a white solid (62 mg, 86% over two steps, *d.r.* >95:5 by NMR).

m.p.: 161-163 °C (diethyl ether)

R_f: 0.29 (7:2:1 hexane:Et_2O:EtOAc)

$[\alpha]^{20}_D$: +31 (*c* 1.0, $CHCl_3$)

FTIR (ν_{max}/cm^{-1}, neat): 3349, 2963, 2922, 1718, 1601, 1502, 1464, 1371, 1261, 1034, 807

^{1}H NMR (500 MHz, $CDCl_3$) δ: 7.83-7.72 (m, 3H, Ar), 7.69 (s, 1H, H_{41}), 7.46-7.38 (m, 3H, Ar), 7.25-7.15 (m, 3H, Ar), 7.09 (d, J = 8.2 Hz, 1H, H_5), 6.87 (m, 2H, Ar), 6.68 (dd, J = 8.2, 2.2 Hz, 1H, H_6), 6.60 (d, J = 2.2 Hz, 1H, H_2), 4.86 (br.s, 1H, H_{25}), 3.57 (ddd, J = 9.9, 4.6, 2.0 Hz, 1H, H_{24}), 3.02 (dd, J = 14.9, 3.1 Hz, 1H, H_{28}), 2.91 (br.dq, J = 9.5, 6.7 Hz, 1H, H_{13}), 2.67 (dd, J = 14.9, 8.3 Hz, 1H, H_{28}), 2.62-2.54 (m, 1H, H_8), 2.48-2.40 (br.m, 2H, H_8, H_{30}), 2.35 (br.dddd, J = 8.3, 8.1, 3.1, 1.7 Hz, 1H, H_{18}), 2.24 (dd, J = 13.6, 9.9, 1H, H_{30}), 2.03 (br.dqd, J = 9.5, 6.6, 1.7 Hz, 1H, H_{17}), 1.88 (br.dq, J = 8.1, 2.4 Hz, 1H, H_{19}), 1.70 (br. dqd, J = 8.7, 6.7, 2.4 Hz, 1H, H_{20}), 1.64 (br. dqd, J = 8.7, 6.7, 4.6 Hz, 1H, H_{23}), 1.50 (sept, J = 6.7 Hz, 1H, H_{10}), 1.39-1.31 (m, 2H, H_9), 1.27 (d, J

= 6.7 Hz, 3H, H_{21}), 0.93 (d, *J* = 6.7 Hz, 3H, H_{29}), 0.88 (d, *J* = 6.9 Hz, 3H, H_{22}), 0.87 (d, *J* = 6.6 Hz, 6H, H_{11}, H_{12}), 0.70 (d, *J* = 6.7 Hz, 3H, H_{27}), 0.70 (d, *J* = 6.7 Hz, 3H, H_{26})

^{13}C NMR (126 MHz, $CDCl_3$) δ: 153.1 (C_1), 142.1 (C_3), 141.0 (C_{31}), 139.4 (C_{32}), 138.0 (C_4), 133.7 (C_{40}), 132.0 (C_{35}), 129.3 ($C_{42,46}$), 128.6 ($C_{43,45}$), 128.1, 127.8, 127.6, 127.5 (C_5), 127.2, 126.4, 126.2, 125.3, 115.8 (C_2), 113.4 (C_6), 74.0 (C_{24}), 42.6 (C_{18}, C_{23}), 40.7 (C_9), 40.3 (C_{17}), 38.3 (C_{30}), 36.5 (C_{13}), 36.3 (C_{20}), 36.0 (C_{19}), 35.8 (C_{28}), 31.3 (C_8), 28.0 (C_{10}), 22.7 ($C_{11/12}$), 22.7 ($C_{11/12}$), 21.7 (C_{21}), 13.4 (C_{27}), 13.1 (C_{26}), 12.1 (C_{22}), 12.0 (C_{29})

HRMS: (MALDI) calcd. for $C_{41}H_{54}O_2Na$ ($M+Na^+$): 601.4016; Found 601.4026

2-((2S,3S,4S,5R,6R,7S,8S)-2-benzyl-8-(2-isopentyl-4-(methoxymethoxy)phenyl)-3,4,5,7-tetramethyl-6-(naphthalen-2-ylmethyl)nonyl)-4,4,5,5-tetramethyl-1,3,2-dioxaborolane, 29

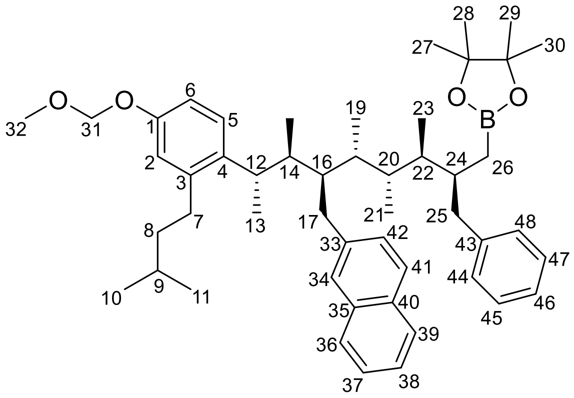

Synthesised according to GP3. Boronic ester **11** (95 mg, 0.13 mmol, 1.0 eq), bromochloromethane (0.026 mL, 0.33 mmol, 2.5 eq) and *n*-BuLi (1.6 M in hexanes, 0.21 mL, 0.33 mmol, 2.5 eq). The crude boronic ester **29** was used directly in the next step.

4-((2S,3S,4R,5R,6R,7R,8S)-8-benzyl-9-hydroxy-3,5,6,7-tetramethyl-4-(naphthalen-2-ylmethyl)nonan-2-yl)-3-isopentylphenol, 13

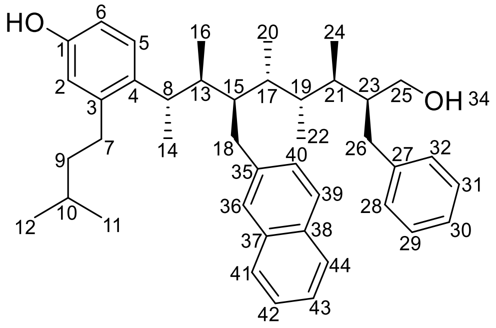

The crude boronic ester **29** was solvated in MeOH (5 mL) and cooled to 0 °C. Concentrated HCl was added (5 drops) and the reaction mixture was stirred at 0 °C for 1 h and then warmed to rt overnight. The reaction mixture was diluted with H_2O (5 mL) and Et_2O (5 mL). The phases were separated and the aqueous phase washes with Et_2O (3 x 5 mL). The combined organic phases were dried ($MgSO_4$) and concentrated under reduced pressure. The crude reaction mixture was taken up in THF (2 mL) and cooled to 0 °C. A premixed solution of NaOH (2M):H_2O_2 (30% aq) (2:1 3 mL) was added dropwise at 0 °C, warmed to room temperature and stirred for 4 h. The reaction mixture was diluted with H_2O (5 mL) and Et_2O (5 mL). The phases were separated and the aqueous phase washes with Et_2O (3 x 5 mL). The combined organic phases were washed with H_2O (5 mL), dried ($MgSO_4$) and concentrated under reduced pressure. The crude material was purified by flash column chromatography (7:2:1 hexane:Et_2O:EtOAc) to give **13** as a white solid (59 mg, 77% over three steps, *d.r.* >95:5 by NMR).

m.p.: 169-174 °C (diethyl ether)

R*f*: 0.25 (7:2:1 hexane:Et_2O:EtOAc)

$[\alpha]^{20}_D$: +20 (*c* 1.0, $CHCl_3$)

FTIR (ν_{max}/cm^{-1}, neat): 3360, 2959, 2934, 1605, 1502, 1470, 1385, 1240, 1027, 818

^{1}H NMR (500 MHz, $CDCl_3$) *δ*: 7.80-7.74 (m, 2H, Ar), 7.73-7.67 (m, 2H, Ar), 7.44-7.35 (m, 3H, Ar), 7.15-7.09 (m, 3H, $H_{29,30,31}$), 7.07 (d, *J* = 8.5 Hz, 1H, H_5), 6.85-6.80 (m, 2H, $H_{28,32}$), 6.66 (dd, *J* = 8.5, 2.9 Hz, 1H, H_6), 6.58 (d, *J* = 2.9 Hz, 1H, H_2), 4.62 (br.s, 1H, H_{34}), 3.46 (dd, *J* = 11.1, 4.3 Hz, 1H, $H_{25'}$), 3.37 (dd, *J* = 11.1, 8.9 Hz, 1H, $H_{25''}$), 3.02 (dd, *J* = 15.1, 3.9 Hz, 1H, $H_{18'}$), 2.86 (br. dq, *J* = 9.9, 6.6 Hz, 1H, H_8), 2.63 (dd, *J* = 15.1, 7.2 Hz, 1H, $H_{18''}$), 2.56-2.48 (m, 1H, $H_{7'}$), 2.44-2.37 (br.m, 1H, $H_{7''}$), 2.37 (dd, *J* = 14.1, 2.6 Hz, 1H, $H_{26'}$), 2.36-2.30 (m, 1H, H_{15}), 2.05-1.97 (m, 1H, H_{13}), 2.00 (dd, *J* = 14.1, 11.2 Hz, 1H, $H_{26''}$), 1.84 (br. dqd, *J* = 8.7, 6.9, 2.0 Hz, 1H, H_{17}), 1.82-1.75 (m, 1H, H_{23}), 1.73-1.65 (m, 2H, $H_{19,21}$), 1.44 (sept, *J* = 6.9 Hz, 1H, H_{10}), 1.32-1.26 (m, 2H, H_9), 1.22 (d, *J* = 6.6 Hz, 3H, H_{14}), 0.86 (d, *J* = 6.9 Hz, 3H, H_{20}), 0.83 (d, *J* = 6.5, 9H, $H_{11,12,24}$), 0.68-0.64 (m, 6H, $H_{16,22}$)

^{13}C NMR (126 MHz, $CDCl_3$) *δ*: 153.0 (C_1), 142.1 (C_3), 141.6 (C_{27}), 141.1 (C_{35}), 138.1, 133.7, 132.0, 129.0 ($C_{28,32}$), 128.4 ($C_{29,31}$), 128.1, 127.7, 127.7, 127.5, 127.0, 126.1, 125.8 (C_{30}), 125.2, 115.8 (C_2), 113.3 (C_6), 64.5 (C_{25}), 44.1 (C_{15}), 42.3 (C_{15}), 40.7 (C_{13}), 40.7 (C_9), 36.9 (C_{17}), 36.2 (C_8), 36.1 (C_{18}), 36.0 ($C_{19,21}$), 32.5 (C_{26}), 31.3 (C_7), 28.0 (C_{10}), 22.7 ($C_{11/12}$), 22.7 ($C_{11/12}$), 21.7 (C_{14}), 13.0 (C_{16}), 12.9 (C_{22}), 12.3 (C_{24}), 12.0 (C_{20})

HRMS: (ESI) calcd. for $C_{42}H_{56}O_2Na$ ($M+Na^+$): 615.4173; Found 615.4156

4-((12S,13R,14R,15R,16R,17S,18S)-12-benzyl-13,14,15,17-tetramethyl-16-(naphthalen-2-ylmethyl)-9-oxo-2,5,10-trioxa-8-azanonadecan-18-yl)-3-isopentylphenyl (2-(2-methoxyethoxy)ethyl)carbamate, 15

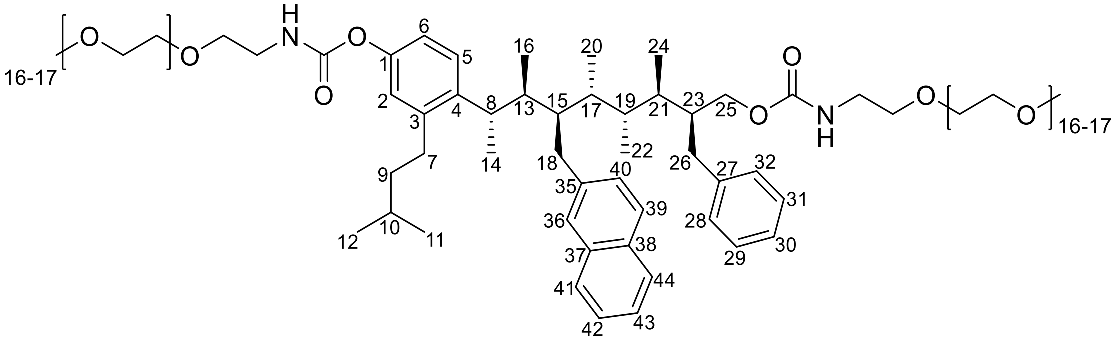

Synthesised according to GP4. Bis-alcohol **13** (19.0 mg, 0.033 mmol, 1.0 eq), Pyridine (0.045 mL, 0.66 mmol, 20.0 eq), phosgene (15 wt.% in toluene) (0.49 mL, 0.66 mmol, 20.0 eq). Formation of acyl chloride: 12h at 40 °C. Pyridine (0.023 mL, 0.33 mmol, 10.0 eq), methoxypolyethylene glycol amine (750 average Mw) in DCM (247 mg, 0.33 mmol, 10 eq). PEGylation: 24 h at 40 °C. The crude reaction mixture was then purified by reversed phase HPLC (Agilent 5 prep-C18 column, 50 × 10.0 mm, 5 μm; gradient elution using CH_3CN in H_2O: 10–40% for 10 min, 40–60% for 5 min, 60–80% for 5 min and 80–95% CH_3CN for 5 min; flow rate 5 ml/min; UV detector at 254 nm and ELS detector, retention time 15.2 min) to yield the bis-PEGylated product **15** (3.7 mg, 11%) as a colourless oil.

R_f: 0.09 (1:10 MeOH:DCM)

$[\alpha]^{20}_D$: +0.42 (*c* 1.0, $CHCl_3$)

FTIR (ν_{max}/cm^{-1}, neat): 3318, 2964, 1731, 1679, 1611, 1465, 1306, 1135, 814

^{1}H NMR (500 MHz, MeOD) δ: 7.85-7.68 (m, 4H, Ar), 7.46 (dd, *J* = 8.4, 0.9 Hz, 1H, Ar), 7.41-7.35 (m, 2H, Ar), 7.13-7.01 (m, 4H, Ar), 6.78 (dd, *J* = 7.7, 1.4 Hz, 2H, Ar), 6.64 (dd, *J* = 8.4, 2.3 Hz, 1H, H_6), 6.54 (d, *J* = 2.3 Hz, 1H, H_2), 3.94 (dd, *J* = 11.2, 8.3 Hz, 1H, $H_{25'}$), 3.88 (dd, *J* = 11.2, 5.2 Hz, 1H, $H_{25'}$), 3.79 (m, 2H, CH_2-PEG), 3.73-3.60 (m, 130H, PEG), 3.59-3.49 (m, 8H, PEG), 3.37 (s, 6H, CH_3-PEG), 3.08 (dd, *J* = 15.1, 3.0 Hz, 1H, $H_{18'}$), 2.92 (br. dq, *J* = 10.1, 6.5 Hz, 1H, H_8), 2.65 (dd, *J* = 15.1, 7.6 Hz, 1H, $H_{18''}$), 2.61-2.53 (m, 1H, $H_{7'}$), 2.43-2.33 (m, 3H, $H_{7''}$, H_{15}, $H_{26'}$), 2.12-2.02 (m, 2H, $H_{26''}$, H_{13}), 1.99-1.94 (br.m, 1H, H_{23}), 1.94-1.81 (br.m, 1H, H_{17}), 1.69 (br.dqd, *J* = 9.7, 6.6, 2.4 Hz, 1H, H_{19}), 1.57 (br.dqd, *J* = 9.7, 6.8, 2.8 Hz, 1H, H_{21}), 1.43 (sept, *J* = 6.5 Hz, 1H, H_{10}), 1.36-1.22 (m, 5H, H_9, H_{14}), 0.94-0.85 (br.d, 6H, H_{20}, H_{24}), 0.84 (br.d, *J* = 6.5 Hz, 6H, H_{11}, H_{12}), 0.69 (d, *J* = 6.9 Hz, 3H, H_{16}), 0.65 (d, *J* = 6.6 Hz, 3H, H_{22})

^{13}C NMR (126 MHz, MeOD) δ: 171.5, 164.1, 155.7, 142.6, 142.3, 142.3, 137.6, 135.1, 133.4, 129.9, 129.2, 129.1, 128.7, 128.5, 128.4, 128.1, 127.1, 126.8, 126.2, 116.8 (C_2), 114.4 (C_6), 73.7 (PEG), 73.0 (PEG), 71.6 (PEG), 71.5 (PEG), 71.5 (PEG), 71.4 (PEG), 71.3 (PEG), 67.5 (C_{25}), 62.2, 59.9 (CH_3-PEG), 59.1, 54.2, 43.5

(C$_{15}$), 42.1 (C$_{9}$), 41.8 (C$_{13}$), 38.0 (C$_{17}$), 37.9 (C$_{19}$), 37.3 (C$_{18}$), 37.2 (C$_{8}$), 33.3 (C$_{26}$), 32.5 (C$_{7}$), 29.0 (C$_{10}$), 23.0 (C$_{11/12}$), 22.9 (C$_{11/12}$), 21.9 (C$_{14}$), 13.2 (C$_{22}$), 13.2 (C$_{16}$), 12.9 (C$_{20}$ or C$_{24}$), 12.1 (C$_{20}$ or C$_{24}$)

MALDI MS:

Table 11. The Mw distribution table obtained from MALDI for PEG derivative **15**

		$[M + Na]^+$		
**n**	**Calculated Mw**	**Calculated**	**Observed**	**%Intensity**
25	1895.2	1918.2	1918.2	14
26	1939.2	1962.2	1962.3	21
27	1983.2	2006.2	2006.3	36
28	2027.3	2050.3	2050.3	47
29	2071.3	2094.3	2094.3	63
30	2115.3	2138.3	2138.4	77
31	2159.3	2182.3	2182.4	86
32	2203.4	2226.4	2226.4	92
**33**	**2247.4**	**2270.4**	**2270.4**	**100**
34	2291.4	2314.4	2314.5	90
35	2335.4	2358.4	2358.5	88
36	2379.5	2402.5	2402.5	81
37	2423.5	2446.5	2446.5	61
38	2467.5	2490.5	2490.6	53
39	2511.5	2534.5	2534.6	40
40	2555.6	2578.6	2578.6	34
41	2599.6	2622.6	2622.6	31
42	2643.6	2666.6	2667.7	26
43	2687.7	2710.7	2710.7	25
44	2731.7	2754.7	2754.7	22
45	2775.7	2798.5	2798.7	19
46	2819.7	2842.7	2842.7	18
47	2863.8	2886.8	2886.8	16
48	2907.8	2930.8	2930.8	14
49	2951.8	2974.8	2974.9	13
50	2995.8	3018.8	3018.9	10

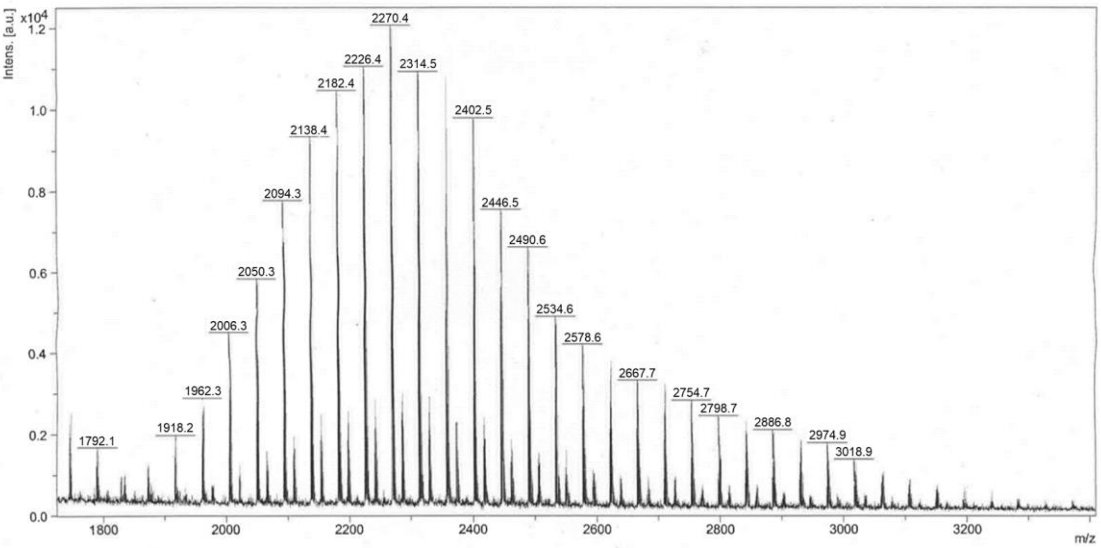

Figure 22. The MALDI MS spectrum of **15**

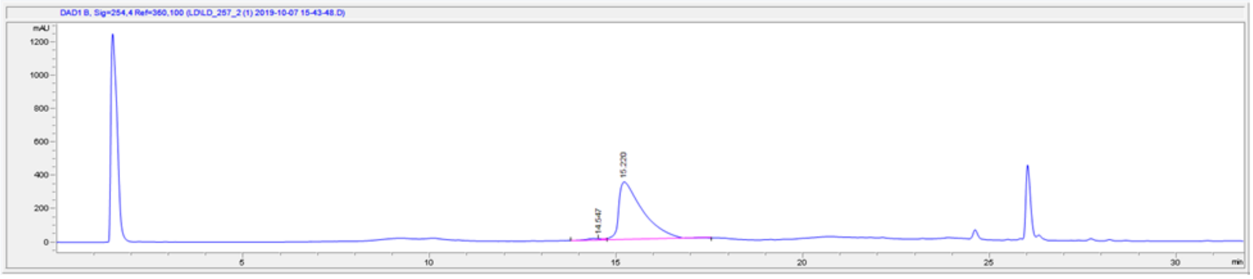

Figure 23 The RP-HPLC trace of **15**. The peak at 15.2 min was collected.

2-((3S,4S,5S)-1-(4-chlorophenyl)-5-(2-isopentyl-4-(methoxymethoxy)phenyl)-4-methylhexan-3-yl)-4,4,5,5-tetramethyl-1,3,2-dioxaborolane, 30

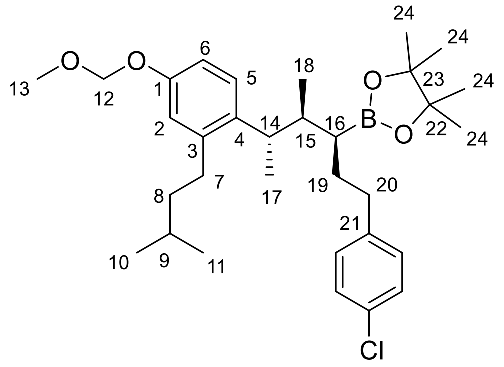

Synthesised according to GP2. Benzoate **9** (270 mg, 0.67 mmol, 1.35 eq), (+)-sparteine (158 mg, 0.67 mmol, 1.35 eq), *s*-BuLi (1.6 M in hexanes, 0.40 mL, 0.65 mmol, 1.30 eq) and boronic ester **24** (195 mg, 0.50 mmol, 1.0 eq). Lithiation time of 2 h, borylation time of 4 h, 1,2- metallate rearrangement time of 4 h. The crude boronic ester was purified by flash column chromatography (1:10 Et_2O:hexane) to give boronic ester **30** (190 mg, 71%) as a colourless oil.

R_f: 0.25 (1:10 Et_2O:hexane)

$[\alpha]^{20}_D$: +21.0 (*c* 1.0, $CHCl_3$)

IR (ν_{max}/cm^{-1}, neat): 2959, 2871, 1609, 1492, 1371, 1312, 1144, 1014

^{1}H NMR (500 MHz, $CDCl_3$) *δ*: δ 7.31-7.24 (m, 2H), 7.21-7.15 (m, 2H), 7.13 (d, *J* = 8.5 Hz, 1H, H_5), 6.86 (dd, *J* = 8.5, 2.7 Hz, 1H, H_6), 6.79 (d, *J* = 2.6 Hz, 1H, H_2), 5.16 (s, 2H, H_{12}), 3.51 (s, 3H, H_{13}), 2.89-2.83 (m, 1H, H_{14}), 2.83-2.76 (m, 1H, H_{20}), 2.70-2.60 (m, 1H, H_7), 2.56-2.50 (m, 1H, H_7), 2.50-2.45 (m, 1H, H_{20}), 1.99-1.89 (m, 1H, H_{15}), 1.87-1.76 (m, 1H, H_{19}), 1.69-1.57 (m, 2H, $H_{9,19}$), 1.50-1.36 (m, 3H, $H_{8,16}$), 1.31 (s, 6H), 1.30 (s, 6H), 1.06 (d, *J* = 6.9 Hz, 3H, H_{17}), 0.96 (d, *J* = .6.5 Hz, 3H, $H_{10/11}$), 0.95 (d, *J* = 6.5 Hz, 3H, $H_{10/11}$), 0.64 (d, *J* = 7.0 Hz, 3H, H_{18})

^{13}C NMR (101 MHz, $CDCl_3$) *δ*: ^{13}C NMR (126 MHz, $CDCl_3$) δ 154.7 (C_1), 141.9, 141.7, 139.1, 131.3, 129.9, 128.3, 127.2 (C_5), 116.8 (C_2), 113.9 (C_6), 94.6 (C_{12}), 83.1 ($C_{22,23}$), 56.0 (C_{13}), 40.8 (C_8), 40.3 (C_{15}), 36.1 (C_{14}), 35.8 (C_{20}), 31.3 (C_7), 28.2 (C_9), 26.2 (C_{19}), 25.2, 24.6, 22.7, 22.5, 21.1 (C_{17}), 16.4 (C_{18})

HRMS: (MALDI) calcd. for $C_{32}H_{48}{}^{11}B^{35}ClO_4Na$ ($M+Na^+$): 565.3232; Found 565.3239

2-((2S,3R,4S,5S)-3-(4-chlorophenethyl)-5-(2-isopentyl-4-(methoxymethoxy)phenyl)-4-methylhexan-2-yl)-4,4,5,5-tetramethyl-1,3,2-dioxaborolane, 31

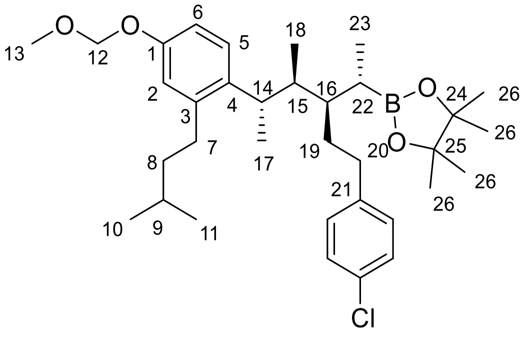

Synthesised according to GP1. Stannane ***(R)*-7** (207 mg, 0.47 mmol, 1.35 eq), *n*-BuLi (1.6 M in hexanes, 0.28 mL, 0.45 mmol, 1.30 eq) and boronic ester **30** (189 mg, 0.35 mmol, 1 eq). Lithiation time of 1 h, borylation time of 2 h, 1,2-metallate rearrangement time of 3 h. The crude boronic ester was purified by flash column chromatography (1:10 Et_2O:hexane) to give boronic ester **31** (170 mg, 85%) as a colourless oil.

R_f: 0.25 (1:10 Et_2O:hexane)

$[\alpha]^{20}_D$: +17.7 (*c* 1.0, $CHCl_3$)

IR (ν_{max}/cm^{-1}, neat): 2956, 2871, 1609, 1495, 1371, 1312, 1145, 1090, 1014.

^{1}H NMR (500 MHz, $CDCl_3$) δ: 7.30-7.23 (m, 2H), 7.20-7.13 (m, 2H), 7.02 (d, J = 8.5 Hz, 1H, H_5), 6.84 (dd, J = 8.5, 2.7 Hz, 1H, H_6), 6.78 (d, J = 2.7 Hz, 1H, H_2), 5.15 (s, 2H, H_{12}), 3.50 (s, 3H, H_{13}), 2.87-2.81 (m, 1H, H_{21}), 2.81-2.75 (m, 1H, H_{14}), 2.71-2.61 (m, 1H, H_7), 2.61-2.53 (m, 1H, H_{20}), 2.53-2.45 (m, 1H, H_7), 1.99-1.90 (m, 1H, H_{16}), 1.78-1.72 (m, 1H, H_{15}), 1.72-1.57 (m, 3H, H_9, H_{19}), 1.49-1.38 (m, 2H, H_8), 1.28 (s, 6H, H_{26}), 1.27 (s, 6H, H_{26}), 1.27-1.22 (m, 1H, H_{22}), 1.14 (d, J = 6.9 Hz, 3H, H_{17}), 1.10 (d, J = 7.3 Hz, 3H, H_{23}), 0.94 (d, J = 6.5 Hz, 3H, $H_{10/11}$), 0.94 (d, J = 6.5 Hz, 3H, $H_{10/11}$), 0.53 (d, J = 6.9 Hz, 3H, H_{18})

^{13}C NMR (101 MHz, $CDCl_3$) δ: 154. 7 (C_1), 142.0, 141. 8, 139.3, 131.3, 129.6, 128.3, 127.0 (C_5), 116.8 (C_2), 113.9 (C_6), 94.6 (C_{12}), 82. 8, 56.0 (C_{13}), 43.3 (C_{15}), 41.0 (C_{16}), 40.8 (C_8), 36.3 (C_{14}), 35. 7 (C_{20}), 31.5 (C_7), 31.1 (C_{19}), 28.3 (C_9), 24.9, 24.8, 22. 7, 22.5, 21.5 (C_{22}), 21.2 (C_{17}), 14.6 (C_{23}), 12.8 (C_{18})

HRMS: (MALDI) calcd. for $C_{34}H_{52}BClO_4Na$ ($M+Na^+$): 593.3545; Found 593.3554

2-((2R,3R,4R,5S,6S)-4-(4-chlorophenethyl)-6-(2-isopentyl-4-(methoxymethoxy)phenyl)-3,5-dimethylheptan-2-yl)-4,4,5,5-tetramethyl-1,3,2-dioxaborolane, 32

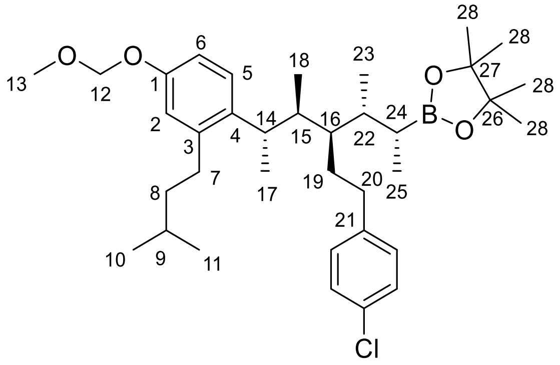

Synthesised according to GP1. Stannane **(*S*)-7** (148 mg, 0.33 mmol, 1.35 eq), *n*-BuLi (1.6 M in hexanes, 0.20 mL, 0.32 mmol, 1.30 eq) and boronic ester **31** (142 mg, 0.25 mmol, 1.0 eq). Lithiation time of 1 h, borylation time of 2 h, 1,2-metallate rearrangement time of 3 h. The crude boronic ester was purified by flash column chromatography (1:10 Et_2O:hexane) to give boronic ester **32** (118 mg, 79%) as a colourless oil.

R_f: 0.24 (1:10 Et_2O:hexane)

$[\alpha]^{20}_D$: +11.1 (*c* 1.0, $CHCl_3$)

IR (ν_{max}/cm^{-1}, neat): 2956, 2927, 2871, 1612, 1495, 1372, 1315, 1144, 1011

^{1}H NMR (500 MHz, $CDCl_3$) δ: 7.30-7.25 (m, 2H), 7.17 (m, 2H), 7.11 (d, *J* = 8.6 Hz, 1H, H_5), 6.87 (dd, *J* = 8.6, 2.7 Hz, 1H, H_6), 6.80 (d, *J* = 2.Hz, 1H, H_2), 5.17 (s, 2H, H_{12}), 3.51 (s, 3H, H_{13}), 2.88-2.79 (m, 1H, H_{14}), 2.81-2.75 (m, 1H, H_{20}), 2.73-2.62 (m, 1H, H_7), 2.65-2.55 (m, 1H, H_{20}), 2.56-2.46 (m, 1H, H_7), 1.94-1.88 (m, 1H, H_{22}), 1.88-1.82 (m, 1H, H_{16}), 1.81-1.74 (m, 1H, H_{15}), 1.73-1.65 (m, 1H, H_{19}), 1.65-1.60 (m, 1H, H_9), 1.58-1.49 (m, 1H, H_{19}), 1.48-1.40 (m, 2H, H_8), 1.37 (qd, *J* = 7.5, 3.8 Hz, 1H, H_{24}), 1.28 (s, 6H), 1.27 (s, 6H), 1.16 (d, *J* = 6.7 Hz, 3H, H_{17}), 1.01 (d, *J* = 7.5 Hz, 3H, H_{25}), 0.95 (d, *J* = 6.6 Hz, 3H, $H_{10/11}$), 0.94 (d, *J* = 6.6 Hz, 3H, $H_{10/11}$), 0.90 (d, *J* = 6.9 Hz, 3H, H_{23}), 0.53 (d, *J* = 6.8 Hz, 3H, H_{18})

^{13}C NMR (126 MHz, $CDCl_3$) δ: 154.7, 141.8, 141.7, 139.3, 131.3, 129.6, 128.4, 127.2 (C_5), 116.7 (C_2), 114.0 (C_6), 94.6 (C_{12}), 82.9, 56.0 (C_{13}), 40.8 (C_8), 40.6 (C_{15}), 39.6 (C_{16}), 36.7 (C_{14}), 36.4 (C_{22}), 35.7 (C_{20}), 31.5 (C_7), 30.8 (C_{19}), 28.3 (C_9), 24.9, 24.9, 22.7, 22.5, 21.7 (C_{17}), 20.0 (C_{24}), 15.3 (C_{23}), 13.1 (C_{18}), 10.4 (C_{25})

HRMS: (MALDI) calcd. for $C_{36}H_{56}BClO_4Na$ ($M+Na^+$): 621.3858; Found 621.3851

2-((2*R*,3R,4R,5S,6S)-4-(4-chlorophenethyl)-6-(2-isopentyl-4-(methoxymethoxy)phenyl)-3,5-dimethylheptan-2-yl)-4,4,5,5-tetramethyl-1,3,2-dioxaborolane, 33

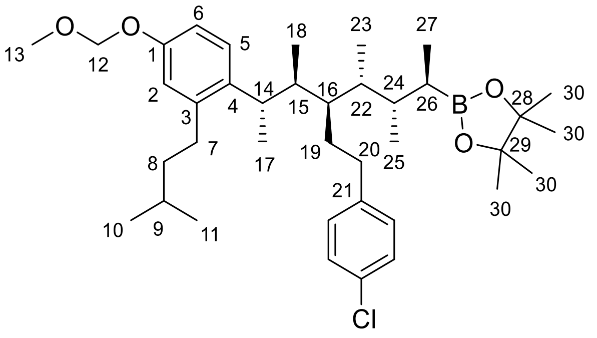

Synthesised according to GP1. Stannane **(*S*)-7** (118 mg, 0.27 mmol, 1.35 eq), *n*-BuLi (1.6 M in hexanes, 0.16 mL, 0.26 mmol, 1.30 eq) and boronic ester **32** (119 mg, 0.20 mmol, 1.0 eq). Lithiation time of 1 h, borylation time of 2 h, 1,2-metallate rearrangement time of 3 h. The crude boronic ester was purified by flash column chromatography (1:10 Et_2O:hexane) to give boronic ester **33** (94 mg, 75%) as a colourless oil.

R_f: 0.23 (1:10 Et_2O:hexane)

$[\alpha]^{20}_D$: +62.5 (*c* 1.0, $CHCl_3$)

IR (ν_{max}/cm^{-1}, neat): 2959, 2931, 2867, 1606, 1492, 1371, 1312, 1144, 1014

^{1}H NMR (500 MHz, $CDCl_3$) δ: 7.30-7.25 (m, 2H), 7.23-7.18 (m, 2H), 7.08 (d, *J* = 8.6 Hz, 1H, H_5), 6.87 (dd, *J* = 8.6, 2.7 Hz, 1H, H_6), 6.81 (d, *J* = 2.7 Hz, 1H, H_2), 5.17 (s, 2H, H_{12}), 3.51 (s, 3H, H_{13}), 2.91-2.81 (m, 1H, H_{14}), 2.79-2.71 (m, 1H, H_{20}), 2.71-2.64 (m, 1H, H_7), 2.64-2.58 (m, 1H, H_{20}), 2.57- 2.48 (m, 1H, H_7), 1.94-1.83 (m, 1H, H_{15}), 1.80-1.75 (m, 1H, H_{24}), 1.78-1.71 (m, 1H, H_{22}), 1.72-1.68 (m, 1H, H_{19}), 1.66-1.59 (m, 1H, H_9), 1.51-1.40 (m, 3H, $H_{8,19}$), 1.29 (s, 6H), 1.28 (s, 6H), 1.29-1.25 (m, 1H, H_{16}), 1.16 (d, *J* = 6.8 Hz, 3H, H_{17}), 1.14-1.07 (m, 1H, H_{26}), 0.97 (d, *J* = 6.6 Hz, 3H, $H_{10/11}$), 0.96 (d, *J* = 6.5 Hz, 3H, $H_{10/11}$), 0.95 (d, *J* = 6.6 Hz, 3H, H_{27}), 0.89 (d, *J* = 6.8 Hz, 3H, H_{25}), 0.77 (d, *J* = 6.5 Hz, 3H, H_{23}), 0.53 (d, *J* = 6.9 Hz, 3H, H_{18})

^{13}C NMR (126 MHz, $CDCl_3$) δ: 154.7, 142.0, 141. 8, 139.1, 131.3, 129.7, 127.9, 127. 2 (C_5), 116.8 (C_2), 114.0 (C_6), 94.6, 82.8, 56.00, 40.9 (C_8), 40.0 (C_{15}), 39.4 (C_{24}), 36.5 (C_{14}), 36.3 (C22), 35.9 (C_{20}), 31.5 (C_7), 31.4 (C_{19}), 28.3 (C_9), 24.9, 24.8, 22.7, 22.6, 21.3 (C_{17}), 14.7 (C_{25}), 14.2 (C_{27}), 12.7 (C_{18}), 11.6 (C_{23})

HRMS: (MALDI) calcd. for $C_{38}H_{60}BClO_4Na$ ($M+Na^+$): 649.4171; Found 649.4183

2-((2S,3S,4S,5R,6R,7S,8S)-6-(4-chlorophenethyl)-8-(2-isopentyl-4-(methoxymethoxy)phenyl)-3,4,5,7-tetramethyl-1-phenylnonan-2-yl)-4,4,5,5-tetramethyl-1,3,2-dioxaborolane, 12

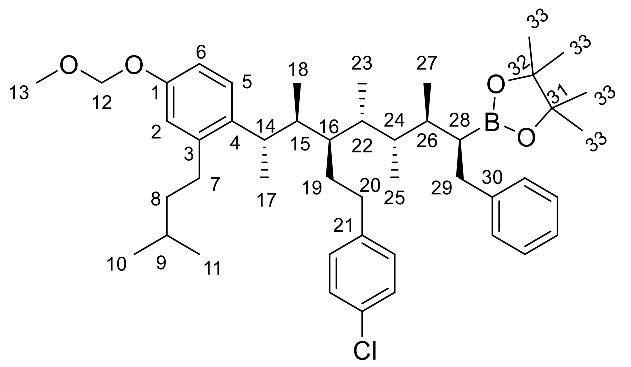

Synthesised according to GP2. Benzoate **10** (62 mg, 0.17 mmol, 1.35 eq), (+)-sparteine (77 mg, 0.17 mmol, 1.35 eq), *s*-BuLi (1.6 M in hexanes, 0.1 mL, 0.17 mmol, 1.30 eq) and boronic ester **33** (81 mg, 0.50 mmol, 1.0 eq). Lithiation time of 2 h, borylation time of 4 h, 1,2-metallate rearrangement time of 4 h. The crude boronic ester was purified by flash column chromatography (1:10 Et_2O:hexane) to give boronic ester **12** (72 mg, 76%) as a colourless oil.

R*f*: 0.25 (1:10 Et_2O:hexane)

$[\alpha]^{20}_D$: +0.51 (*c* 1.0, $CHCl_3$)

IR (ν_{max}/cm^{-1}, neat): 2959, 2934, 2871, 1609, 1492, 1460, 1378, 1311, 1145, 1091, 1014

^{1}H NMR (500 MHz, $CDCl_3$) δ: 7.31-7.26 (m, 3H), 7.26-7.22 (m, 3H), 7.18-7.13 (m, 3H), 7.11 (d, *J* = 8.5 Hz, 1H, H_5), 6.89 (dd, *J* = 8.5, 3.0 Hz, 1H, H_6), 6.82 (d, *J* = 2.7 Hz, 1H, H_2), 5.18 (s, 2H, H_{12}), 3.52 (s, 3H, H_{13}), 2.95-2.84 (m, 1H, H_{14}), 2.78-2.74 (m, 1H), 2.74-2.71 (m, 1H), 2.71-2.69 (m, 1H), 2.69-2.66 (m, 1H), 2.66-2.62 (m, 1H), 2.59-2.50 (m, 1H, H_7), 1.96-1.88 (m, 1H, H_{15}), 1.87-1.80 (m, 1H, H_{26}), 1.78-1.71 (m, 3H), 1.70-1.66 (m, 1H), 1.66-1.60 (m, 1H, H_9), 1.51-1.38 (m, 3H, $H_{8,19}$), 1.19 (d, *J* = 6.7 Hz, 3H, H_{17}), 1.15 (s, 6H), 1.14 (s, 6H), 1.03 (d, *J* = 6.8 Hz, 3H, H_{25}), 0.98 (d, *J* = 6.6 Hz, 3H, H_{27}), 0.97 (d, *J* = 6.5 Hz, 3H, $H_{10/11}$), 0.96 (d, *J* = 6.6 Hz, 3H, $H_{10/11}$), 0.80 (d, *J* = 6.0 Hz, 3H, H_{23}), 0.52 (d, *J* = 6.7 Hz, 3H, H_{18})

^{13}C NMR (126 MHz, $CDCl_3$) δ: 154.7, 143.3, 141.7, 141.6, 139.1, 131.5, 129.5, 129.0, 128.5, 128.0, 127.1, 125.4, 116.8 (C_2), 114.0(C_6), 94.6 (C_{12}), 83.0, 56.0 (C_{13}), 40.8 (C_8), 40.5 ($C_{15, 24}$), 36.7, 36.5, 36.2, 36.2, 36.1 (C_{14}), 31. 8 (C_{19}), 31.6 (C_7), 30.4, 28.6, 28.4 (C_9), 24.8, 24.5, 22.7, 22.6, 21.5 (C_{17}), 16.2 (C_{25}), 12.4, 12.1 (C_{23}), 11. 9 (C_{18})

HRMS: (MALDI) calcd. for $C_{46}H_{68}BClO_4$ ($M+Na^+$): 753.4791; Found 753.4787

2-((2S,3S,4S,5R,6R,7S,8S)-2-benzyl-6-(4-chlorophenethyl)-8-(2-isopentyl-4-(methoxymethoxy)phenyl)-3,4,5,7-tetramethylnonyl)-4,4,5,5-tetramethyl-1,3,2-dioxaborolane, 34

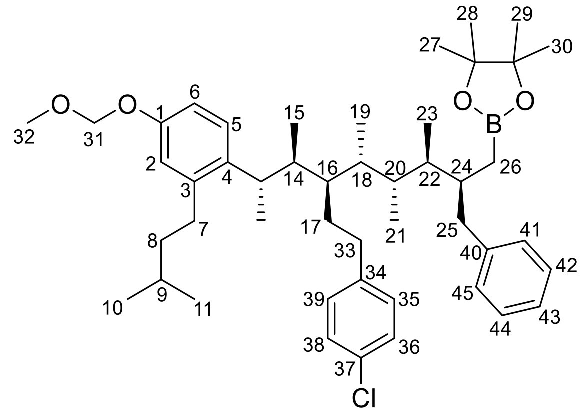

Synthesised according to GP3. Boronic ester **12** (73 mg, 0.10 mmol, 1.0 eq), bromochloromethane (39 mg, 0.30 mmol, 3.0 eq), and *n*-BuLi (1.58 M in hexanes, 0.15 mL, 0.25 mmol, 2.5 eq). The crude boronic ester **34** was used directly in the next step.

4-((2S,3S,4R,5R,6R,7R,8S)-8-benzyl-4-(4-chlorophenethyl)-9-hydroxy-3,5,6,7-tetramethylnonan-2-yl)-3-isopentylphenol, 14

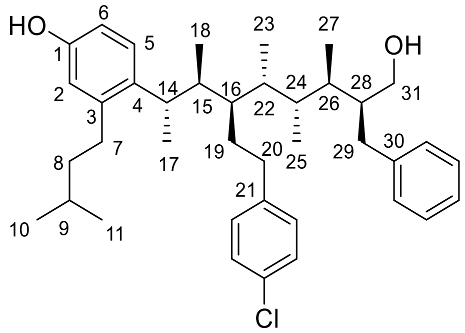

The crude boronic ester **34** was solvated in MeOH (5 mL) and cooled to 0 °C. Concentrated HCl was added (5 drops) and the reaction mixture was stirred at 0 °C for 1 h and then warmed to rt overnight. The reaction mixture was diluted with H_2O (5 mL) and Et_2O (5 mL). The phases were separated and the aqueous phase washes with Et_2O (3 x 5 mL). The combined organic phases were dried ($MgSO_4$) and concentrated under reduced pressure. The crude reaction mixture was taken up in THF (2 mL) and cooled to 0 °C. A premixed solution of NaOH (2M):H_2O_2 (30% aq) (2:1 3 mL) was added dropwise at 0 °C, warmed to room temperature and stirred for 4 h. The reaction mixture was diluted with H_2O (5 mL) and Et_2O (5 mL). The phases were separated and the aqueous phase washes with Et_2O (3 x 5 mL). The combined organic phases were washed with H_2O (5 mL), dried ($MgSO_4$) and concentrated under reduced pressure. The crude material was purified by flash column chromatography (7:2:1 hexane:Et_2O:EtOAc) to give **14** as a colourless oil (42 mg, 72% over three steps, *d.r.* >95:5 by NMR).

R_f: 0.22 (3:7 EtOAc:hexane)

$[\alpha]^{20}_D$: +0.23 (*c* 1.0, $CHCl_3$)

FTIR (ν_{max}/cm^{-1}, neat): 3440, 3158, 2968, 2935, 1605, 1515, 1497, 1382, 1255, 1061

^{1}H NMR (500 MHz, $CDCl_3$) δ: 7.35 – 7.30 (m, 2H), 7.26 – 7.19 (m, 5H), 7.16 – 7.10 (m, 2H), 7.06 (d, *J* = 8.5 Hz, 1H, H_5), 6.69 (dd, *J* = 8.5, 2.8 Hz, 1H, H_6), 6.63 (d, *J* = 2.8 Hz, 1H, H_2), 4.62 (s, 1H, H_{33}), 3.63 (dd, *J* = 10.9, 4.9 Hz, 1H, H_{31}), 3.57 – 3.49 (m, 1H, H_{31}), 2.94 – 2.84 (m, 1H, H_{14}), 2.76 (dd, *J* = 13.5, 3.0 Hz, 1H, H_{29}), 2.73 – 2.61 (m, 3H, $H_{7,20}$), 2.57 – 2.47 (m, 1H, H_7), 2.26 (dd, *J* = 13.5, 11.2 Hz, 1H, H_{29}), 2.21 – 2.14 (m, 1H, H_{28}), 1.96 – 1.88 (m, 1H, H_{15}), 1.88 – 1.82 (m, 1H, H_{26}), 1.82 – 1.72 (m, 4H, $H_{16, 19, 22, 24}$), 1.67 – 1.60 (m, 1H, H_9), 1.50 – 1.39 (m, 3H, $H_{8, 19}$), 1.19 (d, *J* = 6.8 Hz, 3H, H_{17}), 1.10 – 1.03 (brs, 1H, H_{32}), 1.00 (d, *J* = 6.6 Hz, 3H, H_{25}), 0.97 (d, *J* = 6.5 Hz, 3H, $H_{10/11}$), 0.95 (d, *J* = 6.5 Hz, 3H, $H_{10/11}$), 0.92 (d, *J* = 6.9 Hz, 3H, H_{27}), 0.82 (d, *J* = 6.2 Hz, 3H, H_{23}), 0.53 (d, *J* = 6.9 Hz, 3H, H_{18})

^{13}C NMR (126 MHz, $CDCl_3$) δ: 152.9 (C_1), 141.9 (C_3), 141.5 (C_{21}), 141.4 (C_{30}), 137.9 (C_4), 131.5, 129.5, 128.9, 128.6, 128.5, 127.3 (C_5), 126.1, 115.7 (C_2), 113.3 (C_6), 64.5 (C_{31}), 44.0 (C_{28}), 40.8 (C_8), 40.5 (C_{15}), 40.2 (C_{16}), 36.6 (C_{20}), 36.3 (C_{22}), 36.0 (C_{14}), 35.8 (C_{24}), 35.6 (C_{26}), 32.4 (C_{29}), 31.6 (C_{19}), 31.4 (C_7), 28.3 (C_9), 22.7 (C_{11}), 22.6 (C_{10}), 21.5 (C_{17}), 12.6 (C_{25}), 12.3 (C_{27}), 12.2 (C_{18}), 11.8 (C_{23})

HRMS: (MALDI) calcd. for $C_{39}H_{55}ClO_2Na$ ($M+Na^+$): 613.3788; Found 613.3796

4-((12S,13R,14R,15R,16R,17S,18S)-12-benzyl-16-(4-chlorophenethyl)-13,14,15,17-tetramethyl-9-oxo-2,5,10-trioxa-8-azanonadecan-18-yl)-3-isopentylphenyl (2-(2-polymethoxyethoxy)ethyl)carbamate, 16

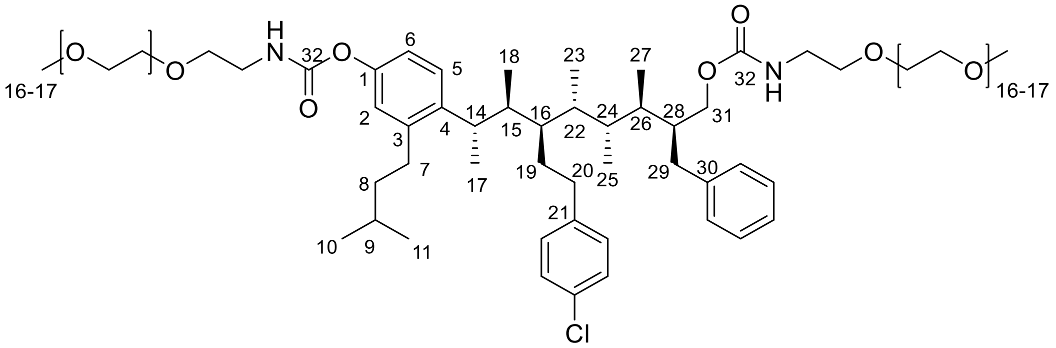

Synthesised according to GP4. Bis-alcohol **14** (12.0 mg, 0.02 mmol, 1.0 eq), Pyridine (32.0 µL, 0.40 mmol, 20.0 eq), phosgene (15 wt.% in toluene) (40 mg, 0.40 mmol, 20.0 eq). Formation of acyl chloride: 12h at 37 °C. Pyridine (32.0 µL, 0.40 mmol, 20.0 eq), methoxypolyethylene glycol amine (750 average Mw) in DCM (150 mg, 0.20 mmol, 10.0 eq). PEGylation: 12 h at 37 °C. The crude reaction mixture was then purified by reversed phase HPLC (Agilent 5 prep-C18 column, 50 × 10.0 mm, 5 µm; gradient elution using CH3CN in H2O: 10–40% for 10 min, 40–60% for 5 min, 60–80% for 5 min and 80–95% CH3CN for 5 min; flow rate 4 ml/min; UV detector at 254 nm and ELS detector, retention time 23.4 min) to yield the bis-PEGylated product **16** (3.1 mg, 9%) as a colourless oil.

R*f*: 0.12 (1:9 MeOH:DCM)

FTIR (ν_{max}/cm^{-1}, neat): 3353, 2927, 2868, 1743, 1719, 1495, 1459, 1350, 1247, 1103, 1039

^{1}H NMR (500 MHz, MeOD) δ 7.28 (t, *J* = 7.5 Hz, 2H, Ar), 7.26-7.15 (m, 8H, Ar), 6.94 (dd, *J* = 8.5, 2.6 Hz, 1H, H_6), 6.88 (d, *J* = 2.5 Hz, 1H, H_2), 4.04-3.91 (m, 2H, H_{31}), 3.81-3.75 (m, 1H), 3.75-3.57 (m, 126H, PEG), 3.57-3.53 (m, 4H, CH_2-PEG), 3.53-3.47 (m, 2H, CH_2-PEG), 3.38 (t, *J* = 5.5 Hz, 2H, CH_2-PEG), 3.37 (s, 6H, CH_3-PEG), 3.26 (t, *J* = 5.5 Hz, 2H), 3.01-2.91 (m, 1H, H_{14}), 2.81-2.67 (m, 4H, $H_{20,7,29}$), 2.63-2.54 (m, 1H, H_7), 2.42-2.32 (m, 2H, $H_{29,28}$), 2.04-1.95 (m, 1H, H_{15}), 1.87-1.73 (m, 5H), 1.66-1.56 (m, 1H, H_9), 1.52-1.42 (m, 3H), 1.19 (d, *J* = 6.8 Hz, 3H, H_{17}), 1.01-0.91 (m, 12H), 0.84 (d, *J* = 6.1 Hz, 3H, H_{23}), 0.55 (d, *J* = 6.9 Hz, 3H, H_{18})

^{13}C NMR (101 MHz, MeOD) *δ*: 157.7, 156.0, 148.7, 142.3, 141.5, 141.4, 140.9, 131.3, 129.5, 128.6, 128.2, 128.2, 126.7, 125.7, 121.8 (C_2), 119.3 (C_6), 71.6, 70.2, 70.2, 70.0, 69.9, 69.9, 69.6, 69.4, 65.7 (C_{31}), 57.7, 40.8, 40.7, 40.6, 40.3, 40.0, 36.2, 36.1, 36.0, 35.9, 35.5, 31.8 (C_{29}), 31.2 (C_7), 31.0 (C_{19}), 28.1 (C_9), 21.8, 21.3, 20.5 (C_{17}), 11.7, 11.4, 11.2, 10.9 (C_{23})

MALDI MS:

Table 12. The Mw distribution table obtained from MALDI for PEG derivative **16**

n	Calculated Mw	[M + Na]$^+$ Calculated	[M + Na]$^+$ Observed	%Intensity
27	1981.2	2004.2	2004.4	30
28	2025.2	2048.2	2048.5	46
29	2069.2	2092.2	2092.5	52
30	2113.3	2136.3	2136.5	73
31	2157.3	2180.3	2180.6	86
32	2201.3	2224.3	2224.6	96
**33**	**2245.3**	**2268.3**	**2268.6**	**100**
34	2289.4	2312.4	2312.7	86
35	2333.4	2356.4	2356.7	89
36	2377.4	2400.4	2400.7	78
37	2421.4	2444.4	2444.7	50
38	2465.5	2488.5	2488.8	58
39	2509.5	2532.5	2532.8	46
40	2553.5	2576.5	2576.8	34
41	2597.5	2620.5	2620.8	30
43	2685.6	2708.6	2708.9	19

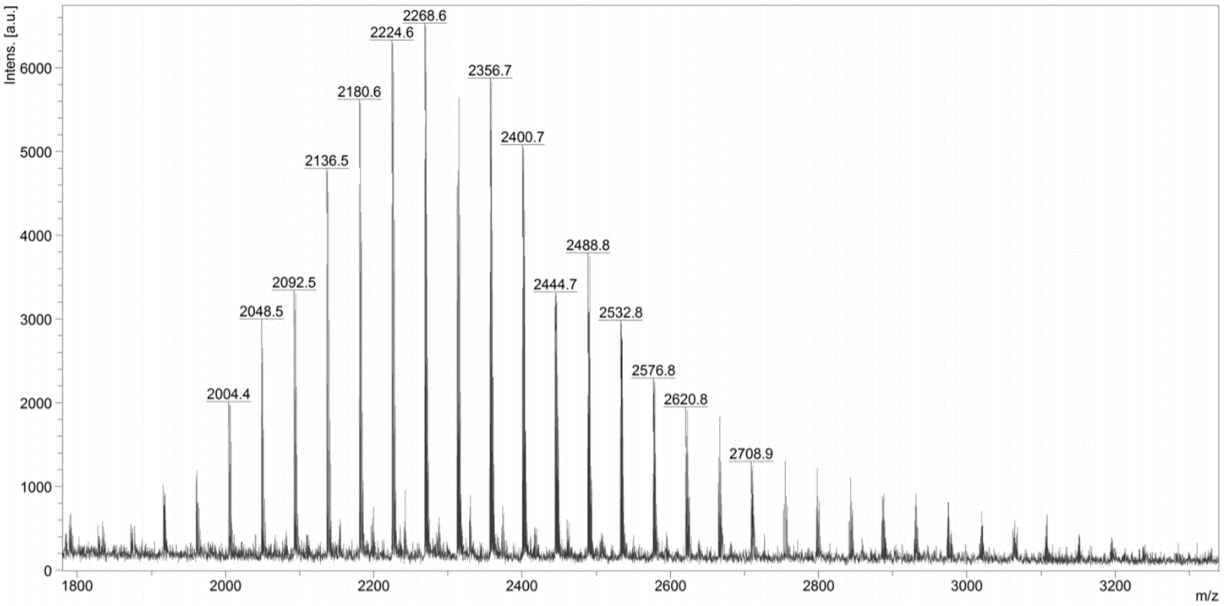

Figure 24. The MALDI MS spectrum of **16**

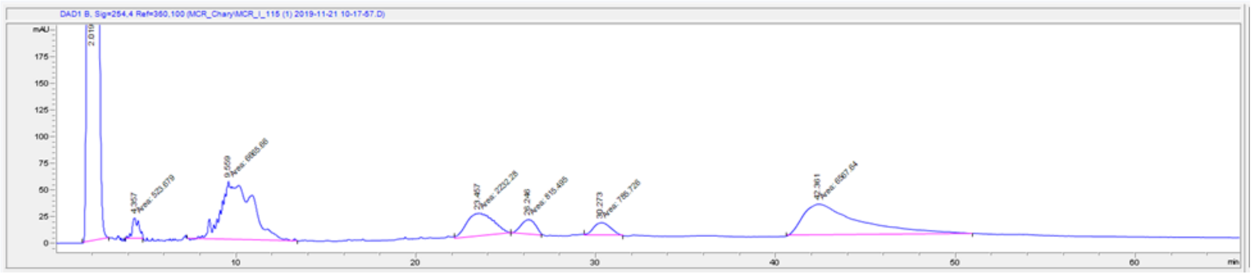

Figure 25 The RP-HPLC trace of **16**. The peak at 23.4 min was collected.

2-(4-(methoxymethoxy)phenyl)-4,4,5,5-tetramethyl-1,3,2-dioxaborolane, 35

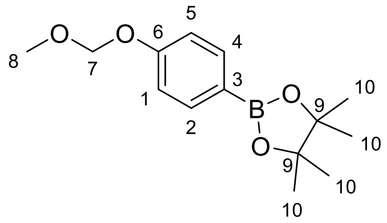

Under a nitrogen atmosphere, 4-hydroxyphenylboronic acid pinacol ester (2.2 g, 10.00 mmol, 1.0 eq) was dissolved in 40 mL of anhydrous DCM, and the solution was cooled to 0 °C. Diisopropylethylamine (2.6 mL, 15.00 mmol, 1.5 eq) was added, followed by methoxymethyl chloride (0.9 mL, 12.00 mmol, 1.2 eq). The solution was stirred at room temperature for 12 h and quenched by addition of water (100 mL). The solution was extracted with DCM (3 x 50 mL). The ether extracts were combined, washed with brine (100 mL), dried ($MgSO_4$), and concentrated to afford a brown oil. The crude product was purified by flash column chromatography (1:9 EtOAc:hexane) to afford the boronic ester **35** in 2.5g (96%) as a colourless oil.

R_f: 0.60 (1:9 Et_2O:hexane)

IR (ν_{max}/cm^{-1}, neat): 2956, 2872, 1606, 1497, 1463, 1372, 1315, 1144, 1011.

^{1}H NMR (500 MHz, $CDCl_3$) δ: 7.84-7.73 (m, 2H), 7.10-7.01 (m, 2H), 5.23 (s, 2H, H_7), 3.49 (s, 3H, H_8), 1.36 (s, 12H, H_{10})

^{13}C NMR (126 MHz, $CDCl_3$) δ: 159.8 (C_6), 136.5, 115.4, 94.1 (C_7), 83.6 (C_9), 56.0 (C_8), 24.9 (C_{10})

HRMS: (ESI) calcd. for $C_{14}H_{21}BO_4Na$ ($M+Na^+$): 287.1431; Found 287.1427

2-((2R,3S)-3-(4-(methoxymethoxy)phenyl)butan-2-yl)-4,4,5,5-tetramethyl-1,3,2-dioxaborolane, 36

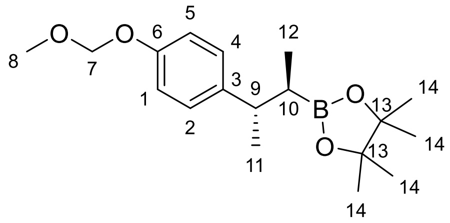

Boronic ester **35** (265 mg, 1.00 mmol, 1.0 eq) was subjected to two iterative homologations according to GP1. After each homologation reaction, the reaction mixture was filtered through a plug of silica and the solvent was removed under reduced pressure to give the crude boronic ester.

1st homologation. Synthesised according to GP1: Stannane **(*S*)-7** (594 mg, 1.35 mmol, 1.35 eq), *n*-BuLi (1.6 M in hexanes, 0.81 mL, 1.30 mmol, 1.30 eq) and boronic ester **35** (265 mg, 1.00 mmol, 1.0 eq). Lithiation time of 1 h, borylation time of 2 h, 1,2-metallate rearrangement time of 3 h.

2nd homologation. Synthesised according to GP1: Stannane **(*S*)-7** (594 mg, 1.35 mmol, 1.35 eq), *n*-BuLi (1.6 M in hexanes, 0.81 mL, 1.30 mmol, 1.30 eq). The crude boronic ester (~1 mmol) was added as a solution in anhydrous Et_2O (2mL). Lithiation time of 1 h, borylation time of 2 h, 1,2-metallate rearrangement time of 3 h. After two homologations the crude boronic ester was purified by flash column chromatography (1:10 Et_2O:hexane) to give boronic ester **36** (260 mg, 81%) as a colourless oil.

R_f: 0.32 (1:9 Et_2O:hexane)

$[\alpha]^{20}_D$: +12.0 (*c* 0.1, $CHCl_3$)

IR (ν_{max}/cm^{-1}, neat): 2963, 2927, 1612, 1511, 1463, 1380, 1313, 1234, 1152, 1010.

^{1}H NMR (500 MHz, $CDCl_3$) δ: 7.13-7.08 (m, 2H, $H_{2,4}$), 6.99-6.95 (m, 2H, $H_{1,5}$), 5.17 (s, 2H, H_7), 3.50 (s, 3H, H_8), 2.68 (dq, J = 10.5, 6.9 Hz, 1H, H_9), 1.30 (s, 6H), 1.29 (s, 6H), 1.23 (d, J = 6.9 Hz, 3H, H_{11}), 1.26-1.21 (m, 1H, H_{10}), 0.76 (d, J = 7.4 Hz, 3H, H_{12})

^{13}C NMR (126 MHz, $CDCl_3$) δ: 155.3 (C_6), 140.8 (C_3), 128.2 ($C_{2,4}$), 116.0 ($C_{1,5}$), 94.6 (C_7), 83.0, 55.9 (C_8), 42.0 (C_9), 25.2 (C_{10}), 24.8 (C_{14}), 24.7 (C_{14}), 23.0 (C_{11}), 14.3 (C_{12})

HRMS: (ESI) calcd. for $C_{18}H_{29}BO_4Na$ ($M+Na^+$): 343.2057; Found 343.2054

2-((2R,3R,4R,5S,6S)-6-(4-(methoxymethoxy)phenyl)-3,4,5-trimethylheptan-2-yl)-4,4,5,5-tetramethyl-1,3,2-dioxaborolane, 37

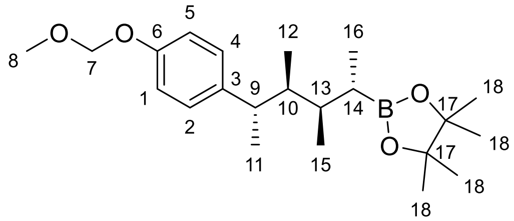

Boronic ester **36** (256 mg, 0.80 mmol, 1.0 eq) was subjected to two iterative homologations according to GP1. After each homologation reaction, the reaction mixture was filtered through a plug of silica and the solvent was removed under reduced pressure to give the crude boronic ester for the subsequent reaction.

1st homologation. Synthesised according to GP1: Stannane **(*R*)-7** (475 mg, 1.08 mmol, 1.35 eq), *n*-BuLi (1.6 M in hexanes, 0.66 mL, 1.04 mmol, 1.30 eq) and boronic ester **36** (256 mg, 0.80 mmol, 1.0 eq). Lithiation time of 1 h, borylation time of 2 h, 1,2-metallate rearrangement time of 3 h.

2nd homologation. Synthesised according to GP1: Stannane **(*R*)-7** (475 mg, 1.08 mmol, 1.35 eq), *n*-BuLi (1.6 M in hexanes, 0.66 mL, 1.04 mmol, 1.30 eq). The crude boronic ester (~1mmol) was added as a solution in anhydrous Et_2O (2mL). Lithiation time of 1 h, borylation time of 2 h, 1,2-metallate rearrangement time of 3 h. After two homologations the crude boronic ester was purified by flash column chromatography (1:10 Et_2O:hexane) to give boronic ester **37** (216 mg, 72%) as a colourless oil.

R_f: 0.35 (1:9 Et_2O:hexane).

$[\alpha]^{20}_D$: –5.0 (*c* 0.1, $CHCl_3$).

IR (ν_{max}/cm^{-1}, neat): 2966, 2931, 2878, 1612, 1510, 1497, 1378, 1311, 1232, 1152, 1009.

^{1}H NMR (500 MHz, $CDCl_3$) δ: 7.12-7.06 (m, 2H, $H_{2,4}$), 7.00-6.93 (m, 2H, $H_{1,5}$), 5.17 (s, 2H, H_7), 3.51 (s, 3H, H_8), 2.46 (dq, J = 10.2, 6.9 Hz, 1H, H_9), 1.88 (dqd, J = 10.1, 6.9, 3.3 Hz, 1H, H_{13}), 1.76 (dqd, J = 10.0, 6.8, 3.3 Hz, 1H, H_{10}), 1.28 (s, 12H), 1.20 (d, J = 6.9 Hz, 3H, H_{11}), 1.05 – 0.99 (m, 1H, H_{14}), 0.96 (d, J = 7.0 Hz, 3H, H_{16}), 0.82 (d, J = 6.9 Hz, 3H, H_{15}), 0.47 (d, J = 6.8 Hz, 3H, H_{12}).

^{13}C NMR (126 MHz, $CDCl_3$) δ: 155.2 (C_6), 141.8 (C_3), 128.4 ($C_{2,4}$), 115.9 ($C_{1,5}$), 94.7 (C_7), 82.8, 56.0 (C_8), 42.8 (C_9), 39.3 (C_{10}), 35.2 (C_{13}), 24.8, 24. 7, 21.2 (C_{14}), 20.6 (C_{11}), 13.6, 13.5, 11.1 (C_{12}).

HRMS: (ESI) calcd. for $C_{22}H_{37}BO_4Na$ (M+Na$^+$): 399.2683; Found 399.2683.

2-((2S,3S,4S,5R,6R,7S,8S)-8-(4-(methoxymethoxy)phenyl)-3,4,5,6,7-pentamethylnonan-2-yl)-4,4,5,5-tetramethyl-1,3,2-dioxaborolane, 38

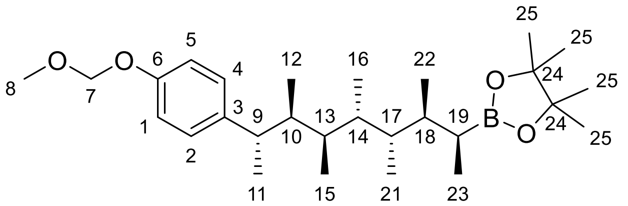

Boronic ester **37** (188 mg, 0.5 mmol, 1.0 eq) was subjected to three iterative homologations according to GP1. After each homologation reaction, the reaction mixture was filtered through a plug of silica and the solvent was removed under reduced pressure to give the crude boronic ester for the subsequent reaction.

1st homologation. Synthesised according to GP1: Stannane **(*S*)-7** (297 mg, 0.67 mmol, 1.35 eq), *n*-BuLi (1.6 M in hexanes, 0.40 mL, 0.65 mmol, 1.30 eq) and boronic ester **37** (188 mg, 0.50 mmol, 1.0 eq). Lithiation time of 1 h, borylation time of 2 h, 1,2-metallate rearrangement of 3 h.

2nd homologation. Synthesised according to GP1: Stannane **(*S*)-7** (297 mg, 0.67 mmol, 1.35 eq), *n*-BuLi (1.6 M in hexanes, 0.40 mL, 0.65 mmol, 1.30 eq). The crude boronic ester (~0.5 mmol) was added as a solution in anhydrous Et_2O (2mL). Lithiation time of 1 h, borylation time of 2 h, 1,2-metallate rearrangement of 3 h.

3rd homologation. Synthesised according to GP1: Stannane **(*R*)-7** (264 mg, 0.60 mmol, 1.35 eq), *n*-BuLi (1.6 M in hexanes, 0.36 mL, 0.58 mmol, 1.30 eq). The crude boronic ester (~0.45 mmol) was added as a solution in anhydrous Et_2O (2mL). Lithiation time of 1 h, borylation time of 2 h, 1,2-metallate rearrangement of 3 h. After three homologations the crude boronic ester was purified by flash column chromatography (1:10 Et_2O:hexane) to give boronic ester **38** (149 mg, 65%) as a colourless oil.

R_f: 0.30 (1:9 Et_2O:hexane).

$[\alpha]^{20}_D$: +35.0 (*c* 1.0, $CHCl_3$).

IR (ν_{max}/cm^{-1}, neat): 2961, 2931, 2871, 1725, 1609, 1467, 1378, 1251, 1137, 1075.

^{1}H NMR (500 MHz, $CDCl_3$) δ: 7.09 (m, 2H, $H_{2,4}$), 6.97 (m, 2H, $H_{1,5}$), 5.18 (s, 2H, H_7), 3.51 (s, 3H, H_8), 2.46 (dq, J = 10.3, 6.9 Hz, 1H, H_9), 1.82 – 1.64 (m, 3H), 1.56 – 1.45 (m, 2H), 1.40 – 1.33 (m, 1H, H_{19}), 1.29 – 1.24 (m, 12H, H_{25}), 1.19 (d, J = 6.9 Hz, 3H, H_{11}), 0.86 (d, J = 7.4 Hz, 3H, H_{23}), 0.83 (d, J = 6.8 Hz, 3H, H_{22}), 0.79 – 0.73 (m, 9H), 0.48 (d, J = 6.8 Hz, 3H, H_{12}).

^{13}C NMR (101 MHz, $CDCl_3$) δ: 155.2, 141.9, 128.4, 116.0, 94.7 (C_7), 82.8, 56.0 (C_8), 43.4 (C_9), 39.8 (C_{10}), 35.8, 35.5, 35.1, 35.0, 29.7, 25.0, 24.7, 20.5 (C_{11}), 19.1 (C_{19}), 15.8 (C_{22}), 11.9 (C_{12}), 11.3, 11.2, 10.9, 8.0 (C_{23}).

HRMS: (ESI) calcd. for $C_{28}H_{49}BO_4Na$ ($M+Na^+$): 483.3622; Found 483.3621.

2-((2R,3S,4S,5R,6R,7S,8S)-8-(4-(methoxymethoxy)phenyl)-2,3,4,5,6,7-hexamethylnonyl)-4,4,5,5-tetramethyl-1,3,2-dioxaborolane, 39

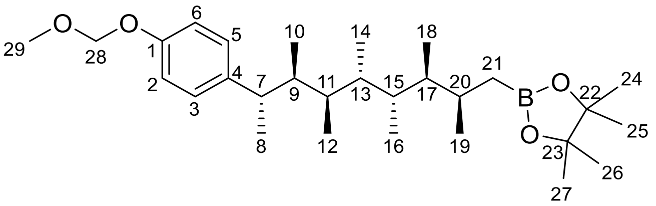

Synthesised according to GP3. Boronic ester **38** (138 mg, 0.30 mmol, 1.0 eq), bromochloromethane (117 mg, 0.90 mmol, 3.0 eq) and *n*-BuLi (1.6 M in hexanes, 0.45 mL, 0.75 mmol, 2.5 eq). The crude boronic ester **39** was used directly in the next step.

4-((2S,3S,4R,5S,6R,7R,8S)-9-hydroxy-3,4,5,6,7,8-hexamethylnonan-2-yl)phenol, 40

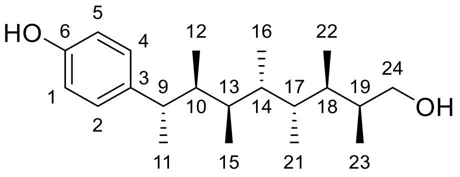

The crude boronic ester **39** was solvated in MeOH (5 mL) and cooled to 0 °C. Concentrated HCl was added (5 drops) and the reaction mixture was stirred at 0 °C for 1 h and then warmed to rt overnight. The reaction mixture was diluted with H_2O (5 mL) and Et_2O (5 mL). The phases were separated and the aqueous phase washes with Et_2O (3 x 5 mL). The combined organic phases were dried ($MgSO_4$) and concentrated under reduced pressure. The crude reaction mixture was taken up in THF (2 mL) and cooled to 0 °C. A premixed solution of NaOH (2M):H_2O_2 (30% aq) (2:1 3 mL) was added dropwise at 0 °C, warmed to room temperature and stirred for 4 h. The reaction mixture was diluted with H_2O (5 mL) and Et_2O (5 mL). The phases were separated and the aqueous phase washes with Et_2O (3 x 5 mL). The combined organic phases were washed with H_2O (5 mL), dried ($MgSO_4$) and concentrated under reduced pressure. The crude material was purified by flash column chromatography (7:2:1 hexane:Et_2O:EtOAc) to give **40** as a colourless oil (85 mg, 89% over three steps, *d.r.* >95:5 by NMR).

R_f: 0.22 (3:7 EtOAc:hexane)

$[\alpha]^{20}_D$: +35.0 (*c* 1.0, $CHCl_3$)

FTIR (ν_{max}/cm^{-1}, neat): 3438, 3119, 2957, 2920, 1594, 1514, 1381, 1254, 1225, 1059, 830

^{1}H NMR (500 MHz, MeOD) δ: 6.98 (d, *J* = 8.5 Hz, 2H, $H_{2,4}$), 6.71 (d, *J* = 8.5 Hz, 2H, $H_{1,5}$), 3.50 -3.39 (m, 2H, H_{24}), 3.37 (s, 1H), 2.45 (dq, *J* = 10.3, 6.9 Hz, 1H, H_9), 1.99-1.95 (m, 1H, H_{19}), 1.85-1.79 (m, 1H, H_{10}), 1.79-1.73 (m, 1H), 1.68-1.61 (m, 2H), 1.61-1.54 (m, 1H, H_{13}), 1.18 (d, *J* = 6.9 Hz, 3H, H_{11}), 0.82 (d, *J* = 6.8 Hz, 3H), 0.80

(d, *J* = 6.6 Hz, 3H), 0.79 (d, *J* = 6.6 Hz, 3H, H_{15}), 0.77 (d, *J* = 7.0 Hz, 3H, H_{23}), 0.73 (d, *J* = 6.3 Hz, 3H), 0.50 (d, *J* = 6.8 Hz, 3H, H_{12})

^{13}C NMR (126 MHz, MeOD) δ: 154.9 (C_6), 139.0 (C_3), 127.9 ($C_{2,4}$), 114.6 ($C_{1,5}$), 66.3 (C_{24}), 43.2 (C_9), 39.8 (C_{10}), 35.8 (C_{19}), 35.2, 35.2, 35.1, 35.1, 19.6 (C_{11}), 10.8 (C_{12}), 10.2, 10.1, 10.1, 9.9, 8.8 (C_{23})

HRMS: (MALDI) calcd. for $C_{21}H_{36}O_2Na$ ($M+Na^+$): 343.2608; Found 343.2615.

(2S,3R,4R,5S,6R,7S,8S)-8-(4-(((2-(2-methoxyethoxy)ethyl)carbamoyl)oxy)phenyl)-2,3,4,5,6,7-hexamethylnonyl poly-(2-(2-methoxyethoxy)ethyl)carbamate, 17

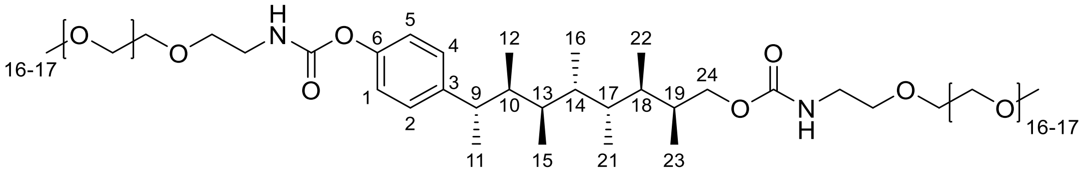

Synthesised according to GP4. Bis-alcohol **40** (12.8 mg, 0.04 mmol, 1.0 eq), Pyridine (64.0 μL, 0.80 mmol, 20.0 eq), phosgene (15 wt.% in toluene) (80 mg, 0.80 mmol, 20.0 eq). Formation of acyl chloride: 12h at 37 °C. Pyridine (64.0 μL, 0.80 mmol, 20.0 eq), methoxypolyethylene glycol amine (750 average Mw) in DCM (300 mg, 0.40 mmol, 10.0 eq). PEGylation: 12 h at 37 °C. The crude reaction mixture was then purified by reversed phase HPLC (Agilent 5 prep-C18 column, 50 × 10.0 mm, 5 μm; gradient elution using CH3CN in H2O: 10–60% for 15 min, 60–80% for 5 min and 80–95% CH3CN for 5 min; flow rate 5 ml/min; UV detector at 254 nm and ELS detector, retention time 15.0 min) to yield the bis-PEGylated product **17** (8.3 mg, 13%) as a colourless oil.

R*f*: 0.20 (1:9 MeOH:DCM)

FTIR (ν_{max}/cm^{-1}, neat): 3360, 2924, 2870, 1743, 1605, 1460, 1350, 1250, 1105

^{1}H NMR (500 MHz, MeOD) *δ*: 7.20 (d, *J* = 8.7 Hz, 2H, $H_{2,4}$), 7.04 (d, *J* = 8.7 Hz, 2H, $H_{1,5}$), 3.95 (d, *J* = 7.3 Hz, 2H, H_{24}), 3.84-3.76 (m, 2H, CH_2-PEG), 3.74-3.58 (m, 128H, PEG), 3.58-3.53 (m, 6H, CH_2-PEG), 3.53-3.46 (m, 2H, CH_2-PEG), 3.39 (t, *J* = 5.4 Hz, 2H, CH_2-PEG), 3.38 (s, 6H, CH_3-PEG), 3.30 (t, *J* = 5.6 Hz, 2H, H_{26}), 2.67-2.48 (m, 1H, H_9), 2.27-2.06 (m, 1H, H_{18}), 1.95-1.75 (m, 2H, $H_{10,13}$), 1.71-1.49 (m, 3H), 1.22 (d, *J* = 6.9 Hz, 3H, H_{11}), 0.90-0.72 (m, 15H), 0.52 (d, *J* = 6.8 Hz, 3H, H_{12})

^{13}C NMR (101 MHz, MeOD) *δ*: 157.8 (C_{25}), 156.0 (C_{28}), 149.3 (C_6), 145.1 (C_3), 127.9 ($C_{2,4}$), 121.2 ($C_{1,5}$), 71.6, 70.3, 70.2, 70.2, 70.2, 70.1, 70.0, 69.9, 69.9, 69.6, 69.4, 68. (C_{24}), 57.7, 43.5 (C_9), 40.6, 40., 39.7 (C_{10}), 35.5, 35.2, 35.1, 33.0 (C_{19}), 19.6 (C_{11}), 11.0 (C_{12}), 10.3, 10.2, 9.9, 8.9 (C_{23})

MALDI MS:

Table 13. The Mw distribution table obtained from MALDI for PEG derivative **17**

n	Calculated Mw	$[M + Na]^+$ Calculated	$[M + Na]^+$ Observed	%Intensity
23	1535.0	1557.9	1558.2	20
24	1579.0	1602.0	1602.2	30
25	1623.0	1646.0	1646.3	46
26	1667.0	1690.0	1690.3	53
27	1711.1	1734.1	1734.3	63
28	1755.1	1778.1	1778.4	56
29	1799.1	1822.1	1822.4	66
30	1843.1	1866.1	1866.4	80
31	1887.1	1910.2	1910.4	86
**32**	**1931.2**	**1954.2**	**1954.5**	**100**
33	1975.2	1998.2	1998.5	93
34	2019.2	2042.2	2042.5	70
35	2063.3	2086.3	2086.6	53
36	2107.3	2130.3	2130.6	36
37	2195.4	2218.3	2218.6	30
39	2239.4	2262.4	2262.7	23

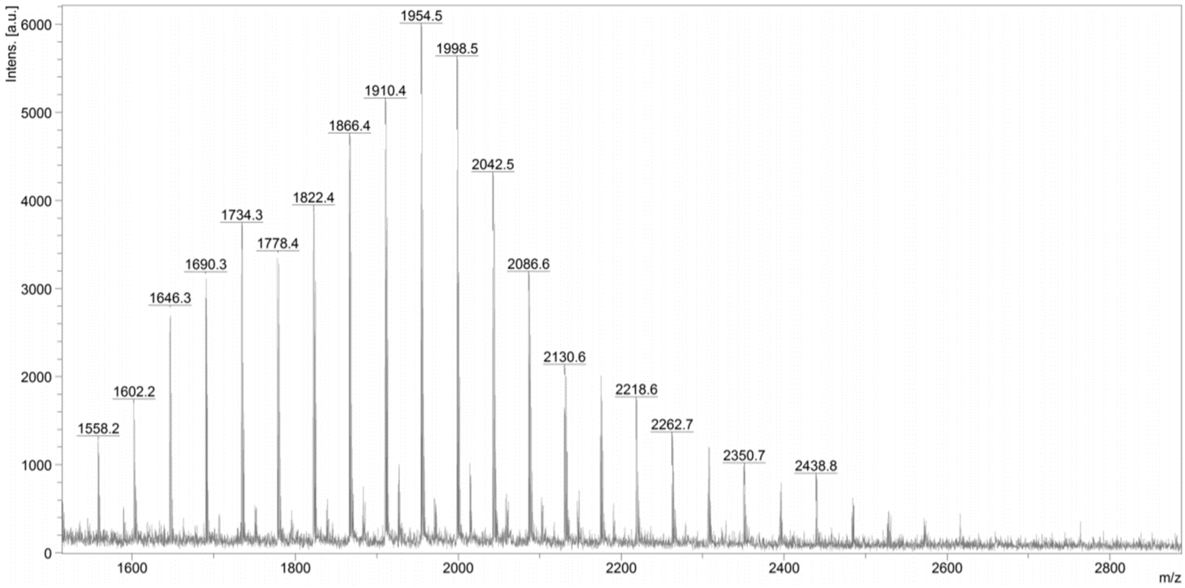

Figure 26. The MALDI MS spectrum of **17**

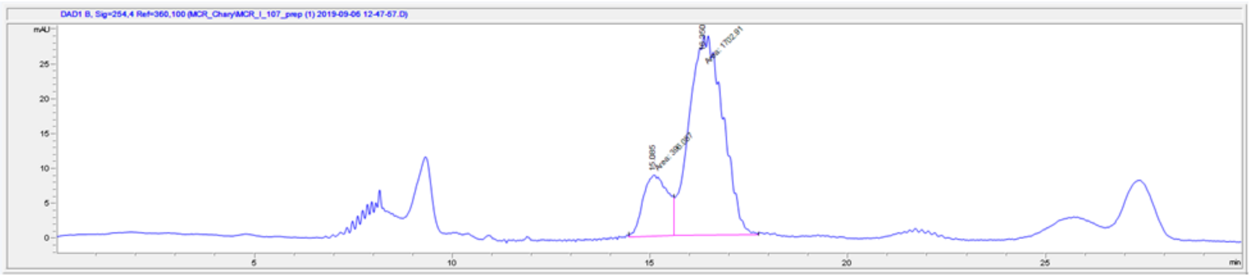

Figure 27 The RP-HPLC trace of **17**. The peak at 15.0 min was collected.

1-bromo-2-isopentyl-4-(methoxymethoxy)benzene, 41

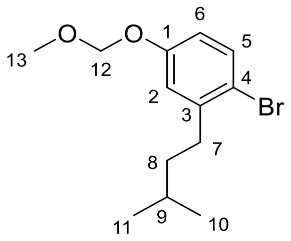

To a solution of (*E*)-1-bromo-4-(methoxymethoxy)-2-(3-methylbut-1-en-1-yl)benzene (1.42 g, 5.00 mmol, 1.0 eq) in ethanol (43 mL) was added PtO_2 (113 mg, 0.50 mmol, 0.1 eq). A balloon of hydrogen gas was added, and the reaction was evacuated and back filled with hydrogen three times. The reaction was stirred under a hydrogen atmosphere overnight at room temperature, then filtered through a pad of celite and concentrated *in vacuo*. The crude product was purified by flash column chromatography (5% Et_2O in Pentane) to give compound **41** (1.3 g, 93%) as a colourless oil.

R_f: 0.43 (1:9 Et_2O:Hexane)

IR (ν_{max}/cm^{-1}, neat): 2956, 2870, 1606, 1497, 1463, 1370, 1313, 1144, 1013

^{1}H NMR (400 MHz, $CDCl_3$) δ: 7.39 (d, J = 8.7 Hz, 1H, H_5), 6.89 (d, J = 2.9 Hz, 1H, H_2), 6.74 (dd, J = 8.7, 2.9 Hz, 1H, H_6), 5.13 (s, 2H, H_{12}), 3.46 (s, 3H, H_{13}), 2.71-2.62 (m, 2H, H_7), 1.70-1.55 (m, 1H, H_9), 1.52-1.42 (m, 2H, H_8), 0.95 (d, J = 6.6 Hz, 6H, $H_{10,11}$)

^{13}C NMR (101 MHz, $CDCl_3$) δ: 156.6 (C_1), 143.5 (C_3), 133.2 (C_5), 118.0 (C_2), 116.2 (C_4), 115.2 (C_6), 94.5 (C_{12}), 56.0 (C_{13}), 39.1 (C_7), 34.3 (C_8), 28.0 (C_9), 22.5 ($C_{10,11}$)

HRMS: (ESI) calcd. for $C_{13}H_{19}BrO_2Na$ (M+Na^+): 309.0461; Found 309.0467

2-(2-isopentyl-4-(methoxymethoxy)phenethyl)-4,4,5,5-tetramethyl-1,3,2-dioxaborolane, 42

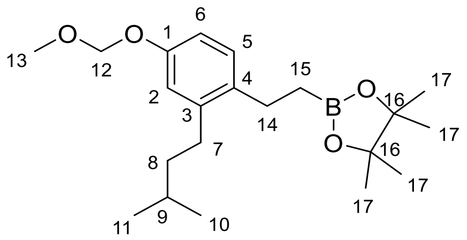

A 100 mL round bottom flask charged with Mg turnings (144 mg, 6.00 mmol, 1.5 eq) and anhydrous LiCl (252 mg, 6.00 mmol, 1.5 eq) was heated with hot air gun under vacuum for 5 minutes. The round bottom flask was then cooled to rt and placed in an ice-water bath (0°C). Anhydrous THF (10 mL) was added and a solution of 1-bromo-2-isopentyl-4-(methoxymethoxy)benzene **41** (1.1 g, 4.00 mmol, 1.0 eq) in THF (5 mL) was added dropwise. The reaction mixture was stirred for 3 h at 65°C. The mixture was then cooled to 0°C and a solution of $FeCl_3$ (32 mg, 0.20 mmol, 0.05 eq) in anhydrous THF (0.5mL) was added, followed by vinyl acetate (1.0 g, 12.00 mmol, 3.0 eq). The mixture was stirred for 3 h at 0°C, then quenched with saturated aqueous NH_4Cl solution (10 mL) and extracted with EtOAc (3 x 10 mL). The combined organic layers were dried over $MgSO_4$ and the solvent was removed under reduced pressure. The crude reaction mixture contains an inseparable mixture of product and stating material in 2:1 ratio, determined by NMR. Therefore, the crude product was directly subjected to the hydroboration. A round-bottom flask was charged with $[Ir(cod)Cl]_2$ (80 mg, 0.12 mmol, 3.0 mol%) and dppb (51 mg, 0.12 mmol, 3.0 mol%). Anhydrous THF (15 mL) was added at rt, followed by pinacolborane (660 mg, 5.20 mmol, 1.3 eq) and the crude reaction mixture in THF (3 mL). The reaction mixture was stirred at rt overnight. The reaction was quenched with methanol (1 mL) and water (3 mL) and the product was extracted with Et_2O (3 x 5 mL), and dried over $MgSO_4$. The crude product was purified by flash column chromatography (1:9 Et_2O:Hexane) to give the boronic ester **42** (622 mg, 44% over two steps) as a colourless oil.

R*f*: 0.35 (1:9 Et_2O:Hexane)

IR (ν_{max}/cm^{-1}, neat): 2956, 2931, 2874, 1612, 1499, 1463, 1372, 1315, 1144, 1011

^{1}H NMR (400 MHz, $CDCl_3$) δ: 7.09 (d, J = 8.1 Hz, 1H, H_5), 6.84 – 6.75 (m, 2H, $H_{2,6}$), 5.12 (s, 2H, H_{12}), 3.46 (s, 3H, H_{13}), 2.73 – 2.61 (m, 2H, H_{14}), 2.61 – 2.51 (m, 2H, H_7), 1.70 – 1.56 (m, 1H, H_9), 1.50 – 1.39 (m, 2H, H_8), 1.22 (s, 12H, H_{17}), 1.12 – 1.02 (m, 2H, H_{15}), 0.94 (d, J = 6.6 Hz, 6H, $H_{10,11}$)

^{13}C NMR (101 MHz, $CDCl_3$) δ: 155.2 (C_1), 142.03, 135.65, 129.37, 116.8 (C_2), 113.3 (C_6), 94.6 (C_{12}), 83.04, 55.9 (C_{13}), 40.4, 30.6, 28.2, 25.7, 24.8, 22.5, 12.9

HRMS: (ESI) calcd. for $C_{21}H_{35}BO_4Na$ ($M+Na^+$): 385.2521; Found 385.2524

(R)-2-(4-(2-isopentyl-4-(methoxymethoxy)phenyl)-1-(naphthalen-2-yl)butan-2-yl)-4,4,5,5-tetramethyl-1,3,2-dioxaborolane, 43

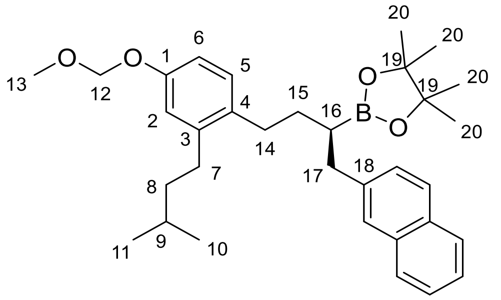

Synthesised according to GP2. benzoate **8** (542 mg, 1.35 mmol, 1.35 eq), (+) sparteine (316 mg, 1.35 mmol, 1.35 eq), *s*-BuLi (1.6 M in hexanes, 0.81 mL, 1.30 mmol, 1.30 eq) and boronic ester **42** (362 mg, 1.00 mmol, 1.0 eq). Lithiation time of 2 h, borylation time of 4 h, 1,2-metallate rearrangement time of 4 h. The crude boronic ester was purified by flash column chromatography (1:10 Et_2O:hexane) to give boronic ester **43** (423 mg, 82%) as a colourless oil.

R_f: 0.30 (1:9 Et_2O:Hexane)

$[\alpha]^{20}_D$: +0.25 (*c* 1.0, $CHCl_3$)

IR (ν_{max}/cm^{-1}, neat): 2961, 2928, 2870, 1695, 1608, 1463, 1292, 954

^{1}H NMR (400 MHz, $CDCl_3$) δ: 7.81-7.69 (m, 3H), 7.63 (brs, 1H), 7.46-7.33 (m, 3H), 7.02 (d, *J* = 7.9 Hz, 1H, H_5), 6.82-6.75 (m, 2H, $H_{2,6}$), 5.12 (s, 2H, H_{12}), 3.46 (s, 3H, H_{13}), 2.96 (dd, *J* = 13.8, 8.4 Hz, 1H, H_{17}), 2.87 (dd, *J* = 13.8, 7.6 Hz, 1H, H_{17}), 2.69-2.55 (m, 2H, H_{14}), 2.55-2.46 (m, 2H, H_7), 1.81-1.62 (m, 2H, H_{15}), 1.62-1.51 (m, 2H, $H_{9,16}$), 1.46-1.35 (m, 2H, H_8), 1.17 (s, 6H), 1.14 (s, 6H), 0.90 (d, *J* = 6.4 Hz, 6H, $H_{10,11}$)

^{13}C NMR (101 MHz, $CDCl_3$) δ: 155.3 (C_1), 142.2 (C_3), 139.7, 134.0 (C_4), 133.5, 132.0, 130.1 (C_5), 127.7, 127.6, 127.6, 127.4, 126.8, 125.7, 124.9, 116.9 (C_2), 113.4 (C_6), 94.6 (C_{12}), 83.1, 55. 9 (C_{13}), 40.4 (C_8), 37.4 (C_{17}), 32.9 (C_{15}), 31.7 (C_{14}), 30.6 (C_7), 28.2 (C_9), 25.8 (C_{16}), 24.8, 22.6, 22.7

HRMS: (MALDI) calcd. for $C_{33}H_{45}BO_4Na$ ($M+Na^+$): 539.3309; Found 539.3318

(*R*)-2-(6-(2-isopentyl-4-(methoxymethoxy)phenyl)-4-(naphthalen-2-ylmethyl)hexyl)-4,4,5,5-tetramethyl-1,3,2-dioxaborolane, 44

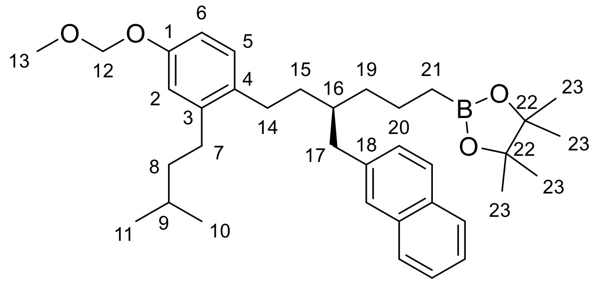

Boronic ester **43** (361 mg, 0.70 mmol, 1.0 eq) was subjected to three iterative Matteson homologations according to GP3. After each homologation reaction, the reaction mixture was filtered through a plug of silica and the solvent was removed under reduced pressure to give the crude boronic ester for the subsequent reaction.

1st homologation. Synthesised according to GP3: Boronic ester **43** (361 mg, 0.70 mmol, 1.0 eq.), Bromochloromethane (271 mg, 2.10 mmol, 3.0 eq), and *n*-BuLi (1.6 M in hexanes, 1.09 mL, 1.75 mmol, 2.5 eq). Lithiation and borylation time of 1 h at -78 °C, 1,2-metallate rearrangement time of 1 h at rt.

2nd homologation. Synthesised according to GP3: The crude boronic ester (~0.70 mmol), Bromochloromethane (271 mg, 2.10 mmol, 3.0 eq), and *n*-BuLi (1.6 M in hexanes, 1.09 mL, 1.75 mmol, 2.5 eq). Lithiation and borylation time of 1 h at -78 °C, 1,2-metallate rearrangement time of 1 h at rt.

3rd homologation. Synthesised according to GP3: The crude boronic ester (~0.70 mmol), Bromochloromethane (271 mg, 2.10 mmol, 3.0 eq), and *n*-BuLi (1.6 M in hexanes, 1.09 mL, 1.75 mmol, 2.5 eq). Lithiation and borylation time of 1 h at -78 °C, 1,2-metallate rearrangement time of 1 h at rt. After three homologations the crude boronic ester was purified by flash column chromatography (1:10 Et_2O:hexane) to give boronic ester **44** (242 mg, 62% over three homologations) as a colourless oil.

R_f: 0.35 (1:9 Et_2O:Hexane).

$[\alpha]^{20}_D$: +70.0 (*c* 1.0, $CHCl_3$).

IR (ν_{max}/cm^{-1}, neat): 2952, 2928, 2871, 1729, 1609, 1499, 1152, 1074, 1017.

^{1}H NMR (400 MHz, $CDCl_3$) δ: 7.78 (dd, *J* = 7.6, 1.7 Hz, 1H, H_{Naph}), 7.73 (dd, *J* = 7.8, 3.6 Hz, 2H, H_{Naph}), 7.56 (brs, 1H, H_{Naph}), 7.46-7.36 (m, 2H, H_{Naph}), 7.30 (dd, *J* = 8.4, 1.7 Hz, 1H, H_{Naph}), 6.97 (d, *J* = 8.1 Hz, 1H, H_5), 6.80-6.71 (m, 2H, $H_{2,6}$), 5.12 (s, 2H, H_{12}), 3.46 (s, 3H, H_{13}), 2.80 (dd, *J* = 14.0, 7.2 Hz, 1H, H_{17}), 2.73 (dd, *J* = 14.0, 7.6 Hz, 1H, H_{17}), 2.63-2.49 (m, 2H, H_{14}), 2.49-2.43 (m, 2H, H_7), 1.85-1.81 (m, 1H, H_{16}), 1.54-1.45 (m, 5H, $H_{20,15,9}$), 1.42-1.31 (m, 4H, $H_{19,8}$), 1.17 (s, 12H), 0.88 (d, *J* = 6.8 Hz, 3H), 0.86 (d, *J* = 6.8 Hz, 3H), 0.76 (t, *J* = 7.5 Hz, 2H, H_{21}).

^{13}**C NMR** (101 MHz, $CDCl_3$) δ: 155.6 (C_1), 142.5 (C_3), 139.4 (C_{18}), 134.4, 133. 9, 132.3, 130.4 (C_5), 128.2, 128.0, 127.9, 127.8, 127.6, 126.1, 125.3, 117.3 (C_2), 113.7 (C_6), 94.9 (C_{12}), 83.2, 56.2 (C_{13}), 41.1 (C_8), 40.9 (C_{17}), 40.1 (C_{16}), 36.4 (C_{19}), 35.5 (C_{15}), 31.0 (C_7), 29.6 (C_{14}), 28.6 (C_9), 25.1, 22.9, 21.5 (C_{21}).

HRMS: (MALDI) calcd. for $C_{36}H_{51}BO_4Na$ ($M+Na^+$): 581.3779; Found 581.3770.

2-((2R,6R)-8-(2-isopentyl-4-(methoxymethoxy)phenyl)-6-(naphthalen-2-ylmethyl)-1-phenyloctan-2-yl)-4,4,5,5-tetramethyl-1,3,2-dioxaborolane, 45

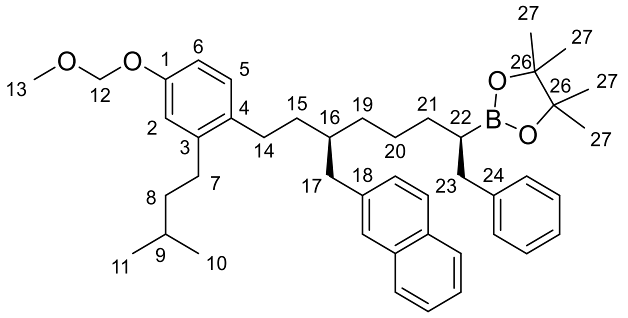

Synthesised according to GP2. benzoate **10** (190 mg, 0.54 mmol, 1.35 eq), (+) sparteine (126 mg, 0.54 mmol, 1.35 eq), *s*-BuLi (1.6 M in hexanes, 0.32 mL, 0.52 mmol, 1.30 eq) and boronic ester **44** (223 mg, 0.40 mmol, 1.0 eq). Lithiation time of 2 h, borylation time of 4 h, 1,2-metallate rearrangement time of 4 h. The crude boronic ester was purified by flash column chromatography (1:10 Et_2O:hexane) to give boronic ester **45** (190 mg, 79%) as a colourless oil.

R_f: 0.32 (1:9 Et_2O:Hexane).

$[\alpha]^{20}_D$: +0.55 (*c* 1.0, $CHCl_3$).

IR (ν_{max}/cm^{-1}, neat): 2956, 2872, 1606, 1497, 1463, 1372, 1315, 1144, 1011.

^{1}H NMR (500 MHz, $CDCl_3$) δ: 7.82 (dd, J = 7.8, 1.7 Hz, 1H, H_{Naph}), 7.79 – 7.73 (m, 2H, H_{Naph}), 7.58 (brs, 1H, H_{Naph}), 7.49 – 7.41 (m, 2H, H_{Naph}), 7.31 (dd, J = 8.4, 1.7 Hz, 1H, H_{Naph}), 7.27 – 7.13 (m, 5H), 6.97 (d, J = 8.0 Hz, 1H, H_5), 6.82 – 6.79 (m, 1H, H_2), 6.77 (dd, J = 8.0, 2.5 Hz, 1H, H_6), 5.14 (s, 2H, H_{12}), 3.49 (s, 3H, H_{13}), 2.82 (dd, J = 13.5, 6.5 Hz, 1H, H_{17}), 2.77 – 2.64 (m, 3H), 2.64 – 2.55 (m, 1H), 2.54 – 2.43 (m, 3H), 1.90 – 1.79 (m, 1H, H_{16}), 1.55 – 1.49 (m, 3H), 1.45 – 1.36 (m, 7H), 1.31 – 1.26 (m, 2H), 1.17 (s, 6H, H_{27}), 1.14 (s, 6H, H_{27}), 0.89 (d, J = 6.5 Hz, 3H, $H_{10/11}$), 0.88 (dd, J = 6.5 Hz, 3H, $H_{10/11}$).

^{13}C NMR (126 MHz, $CDCl_3$) δ: 155.3 (C_1), 142.3, 142.1, 139.0, 134.0, 133.5, 132.0, 130.0 (C_5), 128.9, 128.0, 127.9, 127.7, 127.6, 127.4, 127.3, 125. 8, 125.6, 125.0, 116.9 (C_2), 113.4 (C_6), 94.6 (C_{12}), 83.0, 55.9 (C_{13}), 40.6, 40.5 (C_{17}), 39.7 (C_{16}), 37.3, 35.2, 33.5, 31.4, 30.7, 29.3, 28.2, 26.1, 24.8, 24.7, 22.5 ($C_{10,11}$).

HRMS: (MALDI) calcd. for $C_{44}H_{59}BO_4Na$ ($M+Na^+$): 685.4406; Found 685.4412

2-((2R,6R)-2-benzyl-8-(2-isopentyl-4-(methoxymethoxy)phenyl)-6-(naphthalen-2-ylmethyl)octyl)-4,4,5,5-tetramethyl-1,3,2-dioxaborolane, 46

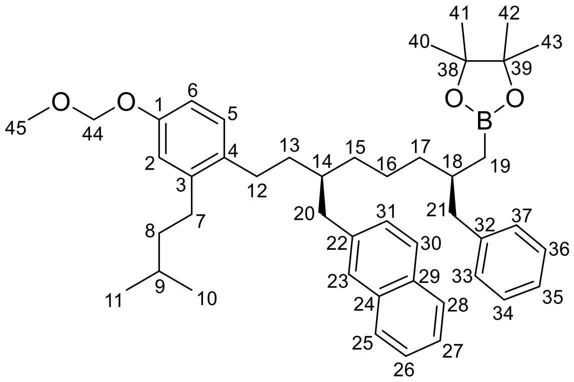

Synthesised according to GP3. Boronic ester **45** (132 mg, 0.20 mmol, 1.0 eq), bromochloromethane (77 mg, 0.60 mmol, 3.0 eq) and *n*-BuLi (1.6 M in hexanes, 0.31 mL, 0.5 mmol, 2.5 eq). The crude boronic ester **46** was used directly in the next step.

4-((3R,7R)-7-benzyl-8-hydroxy-3-(naphthalen-2-ylmethyl)octyl)-3-isopentylphenol, 47

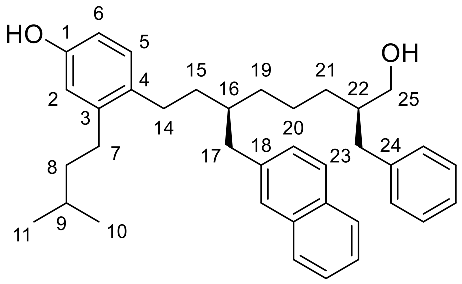

The crude boronic ester **46** was solvated in MeOH (5 mL) and cooled to 0 °C. Concentrated HCl was added (5 drops) and the reaction mixture was stirred at 0 °C for 1 h and then warmed to rt overnight. The reaction mixture was diluted with H_2O (5 mL) and Et_2O (5 mL). The phases were separated and the aqueous phase washes with Et_2O (3 x 5 mL). The combined organic phases were dried ($MgSO_4$) and concentrated under reduced pressure. The crude reaction mixture was taken up in THF (2 mL) and cooled to 0 °C. A premixed solution of NaOH (2M):H_2O_2 (30% aq) (2:1 3 mL) was added dropwise at 0 °C, warmed to room temperature and stirred for 4 h. The reaction mixture was diluted with H_2O (5 mL) and Et_2O (5 mL). The phases were separated and the aqueous phase washes with Et_2O (3 x 5 mL). The combined organic phases were washed with H_2O (5 mL), dried ($MgSO_4$) and concentrated under reduced pressure. The crude material was purified by flash column chromatography (7:2:1 hexane:Et_2O:EtOAc) to give **47** as a colourless oil (64 mg, 61% over three steps).

R_f: 0.4 (4:6 EtOAc:Hexane)

$[\alpha]^{20}_D$: –0.41 (*c* 1.0, MeOH)

IR (ν_{max}/cm^{-1}, neat): 3438, 3119, 2967, 2920, 1594, 1514, 1460, 1382, 1254, 1226, 1060, 830

^{1}H NMR (500 MHz, MeOD) δ: 7.81 (dd, J = 7.7, 1.2 Hz, 1H, Ar), 7.79-7.75 (m, 2H, Ar), 7.58 (s, 1H, Ar), 7.48-7.39 (m, 2H, Ar), 7.29 (dd, J = 8.3, 1.8 Hz, 1H, Ar), 7.25-7.19 (m, 2H, Ar), 7.17-7.11 (m, 3H, Ar), 6.84 (d, J = 8.1 Hz, 1H, H_5), 6.53 (d, J = 2.5 Hz, 1H, H_2), 6.50 (dd, J = 8.1, 2.5 Hz, 1H, H_6), 3.45-3.42 (m, 2H, H_{25}), 2.79 (dd, J = 13.6, 6.8 Hz, 1H, H_{17}), 2.72 (dd, J = 13.6, 3.0 Hz, 1H, H_{17}), 2.68 (dd, J = 9.3, 2.8 Hz, 1H, H_{23}), 2.61-2.54 (m, 1H, H_{14}), 2.53-2.48 (m, 1H, H_{23}), 2.49-2.43 (m, 1H, H_{14}), 2.39 (td, J = 7.4, 3.1 Hz, 2H, H_7), 1.87-1.79 (m, 1H, H_{16}), 1.79-1.70 (m, 1H, H_{22}), 1.56-1.18 (br m, 12H), 0.86 (d, J = 6.7 Hz, 3H), 0.84 (d, J = 6.7 Hz, 3H)

^{13}C NMR (126 MHz, MeOD) δ: 154.8 (C_1), 141.5, 140.9, 138.9, 133.7, 132.2, 131.1, 129.9 (C_5), 128.8, 127.8, 127.4, 127.3, 127.2, 127.0, 126.9, 125.4, 125.4, 124.7, 115.3 (C_2), 112.3 (C_6), 63.7 (C_{25}), 42.4 (C_{22}), 40.5, 40.5, 39.2 (C_{16}), 37.1 (C_{23}), 35.3, 33.4, 30.4, 30.3, 29.0 (C_{14}), 27.9 (C_9), 23.5, 21.6

HRMS: (MALDI) calcd. for $C_{37}H_{46}O_2Na$ (M+Na$^+$): 545.3990; Found 545.3999

4-((3R,7R)-7-benzyl-8-hydroxy-3-(naphthalen-2-ylmethyl)octyl)-3-isopentylphenol, 18

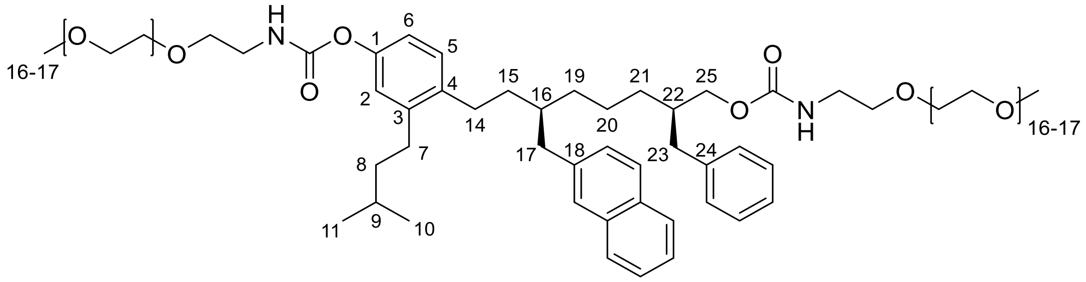

Synthesised according to GP4. Bis-alcohol **47** (15.6 mg, 0.03 mmol, 1.0 eq), Pyridine (47.0 µL, 0.6 mmol, 20.0 eq), phosgene (15 wt.% in toluene) (59 mg, 0.6 mmol, 20.0 eq). Formation of acyl chloride: 12h at 37 °C. Pyridine (47.0 µL, 0.6 mmol, 20.0 eq), methoxypolyethylene glycol amine (750 average Mw) in DCM (225 mg, 0.3 mmol, 10.0 equiv). PEGylation: 12 h at 37 °C. The crude reaction mixture was then purified by reversed phase HPLC (Agilent 5 prep-C18 column, 50 × 10.0 mm, 5 µm; gradient elution using CH3CN in H2O: 10–40% for 10 min, 40–60% for 5 min, 60–80% for 5 min and 80–95% CH3CN for 5 min; flow rate 5 ml/min; UV detector at 254 nm and ELS detector, retention time 26.0 min) to yield the bis-PEGylated product **18** (6.1 mg, 12%) as a colourless oil.

R_f: 0.25 (1:9 MeOH:DCM)

FTIR (ν_{max}/cm^{-1}, neat): 3431, 2921, 1610, 1514, 1462, 1382, 1225, 1060

^{1}H NMR (500 MHz, Methanol-*d*$_4$) δ: 8.56 (s, 2H, Ar), 7.83 (d, *J* = 7.8 Hz, 1H, Ar), 7.79 (d, *J* = 8.1 Hz, 2H, Ar), 7.61 (s, 1H, Ar), 7.48-7.41 (m, 2H, Ar), 7.31 (d, *J* = 8.4 Hz, 1H, Ar), 7.25 (t, *J* = 7.5 Hz, 2H, Ar), 7.20-7.12 (m, 3H, Ar), 7.04 (d, *J* = 8.0 Hz, 1H, H_5), 6.85-6.78 (m, 2H, $H_{1,6}$), 3.97-3.89 (m, 2H, H_{25}), 3.72-3.57 (m, 110H, PEG), 3.57-3.51 (m, 6H, CH_2-PEG), 3.37 (s, 6H, CH_3-PEG), 2.86-2.81 (m, 1H, H_{17}), 2.77-2.54 (m, 5H), 2.50-2.43 (m, 2H, H_7), 2.08-2.02 (m, 1H), 1.97-1.90 (m, 1H, H_{22}), 1.89-1.83 (m, 1H, H_{16}), 1.57-1.43 (m, 4H), 0.87 (d, *J* = 6.7 Hz, 3H), 0.86 (d, *J* = 6.7 Hz, 3H)

^{13}C NMR (126 MHz, Methanol-*d*$_4$) δ: ^{13}C NMR (126 MHz, MeOD) δ 165.4, 149.2, 141.6, 140.2, 138.6, 137.2, 133.7, 132.2, 129.7, 128.8, 128.0, 127.5, 127.3, 127.0, 125.6, 124.9, 121.7, 118.7, 71.6, 70.1, 69.9, 68.8, 66.8, 57.7, 40.5, 40.3, 39.2, 30.1, 29.3, 27.9, 23.4, 21.6

MALDI MS:

Table 14. The Mw distribution table obtained from MALDI for PEG derivative **18**

n	Calculated Mw	$[M + Na]^+$ Calculated	$[M + Na]^+$ Observed	%Intensity
27	1781.1	1804.1	1804.4	11
28	1825.1	1848.1	1848.4	26
29	1869.1	1892.1	1892.4	34
30	1913.1	1936.1	1936.4	46
31	1957.2	1980.2	1980.5	76
32	2001.2	2024.2	2024.5	80
**33**	**2045.2**	**2068.2**	**2068.5**	**100**
34	2089.2	2112.2	2112.6	97
35	2133.3	2156.3	2156.6	96
36	2177.3	2200.3	2200.6	84
37	2221.3	2244.3	2244.6	61
38	2265.4	2288.3	2288.7	42
39	2309.4	2332.4	2332.7	30
40	2353.4	2376.4	2376.7	13
41	2397.4	2420.4	2420.8	7

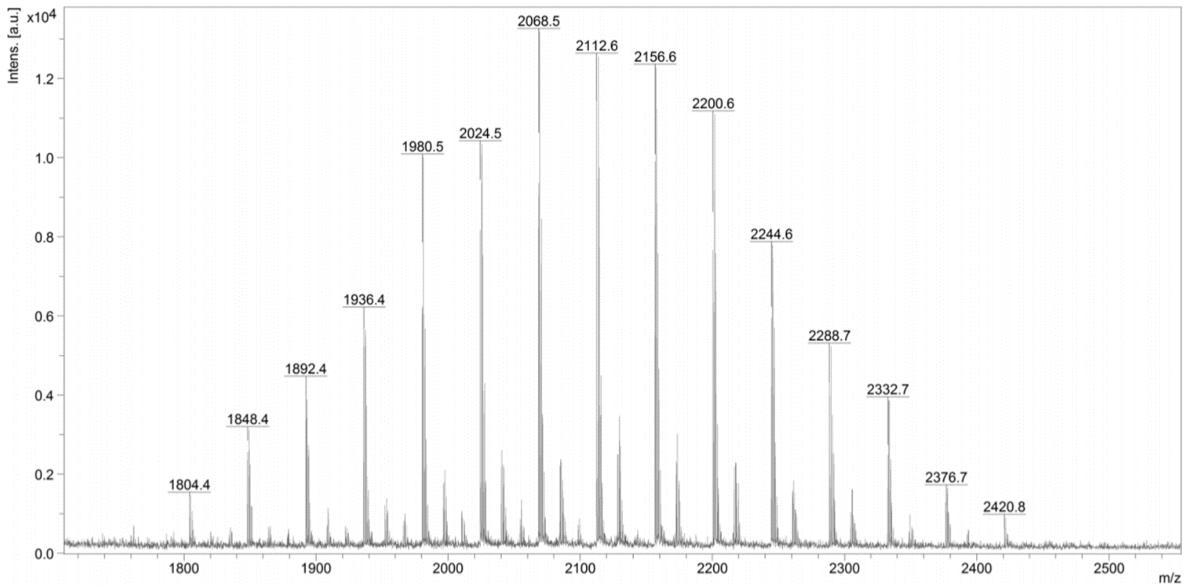

Figure 28. The MALDI MS spectrum of **18**

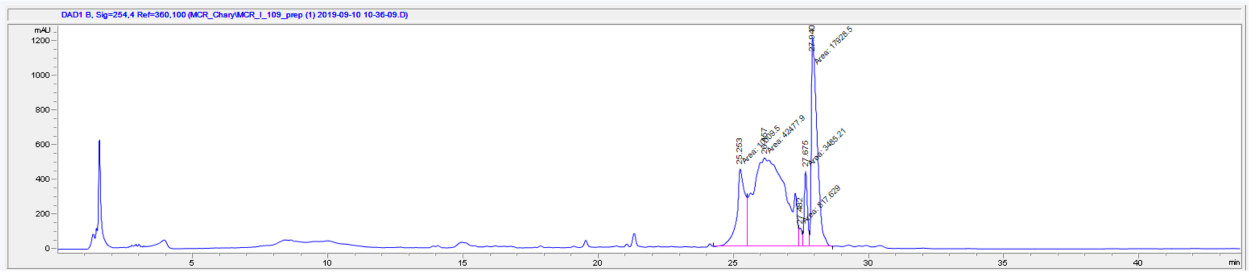

Figure 29 The RP-HPLC trace of **18**. The peak at 26 min was collected.

4. The Calculation of Gibbs Free Energies, Boltzmann Populations and Boltzmann Averaged NMR Parameters using Density Functional Theory

4.1 General Computational Information

DFT Calculations, including geometry optimisations, frequency calculations and NMR calculations, were performed using the Gaussian 09 Software Package.[11] DFT calculations were performed using BlueCrystal Phase 3, a supercomputing facility available within the advanced computing research center at the University of Bristol (http://www.bris.ac.uk/acrc).

Following the conformational search, the top 99.9% of conformers, corresponding to 241 conformers for **3**, were subjected to a DFT geometry optimisation-frequency calculation using mpw1pw91/6-31g(d) basis set (basis set 1-BS1). The integral equation formalism polarisable continuum model was used for solvation (IEFPCM, chloroform). Conformers that either did not converge or converged to an imaginary frequency were eliminated from the conformational pool.

The optimised geometries were subjected to a frequency single point calculation using mpw1pw91/6-311g(d,p) (basis set 2-BS2) to obtain a more accurate description of conformer energies. The calculations were performed using the same solvation method in chloroform. The Gibbs free energies (in kJ mol^{-1}) of each conformer were estimated according to:

$$G_i^{BS2} = E_i^{BS2} + \left(G_i^{BS1} - E_i^{BS1}\right)$$

Equation 2.The estimation of the Gibbs energy of conformer *i*.

Where G_i^{BS2} is the estimated Gibbs energy of conformer *i* using 6-311g(d,p), E_i^{BS2} is the potential energy of conformer i calculated using 6-311g(d,p), G_i^{BS1} is the calculated Gibbs energy of conformer *i* using 6-31g(d) and E_i^{BS1} is the potential energy of conformer *i* calculated using 6-31g(d).

The estimated Gibbs energies of each conformer were subsequently used in the Boltzmann analysis of conformer population according to:

$$P_i = 100\,x\,\left(\frac{g e^{\frac{\Delta G_i^{BS2}}{RT}}}{\sum_{i=1}^{n} g e^{\frac{\Delta G_i^{BS2}}{RT}}}\right)$$

Equation 3. The calculation of the Boltzmann population of conformer *i*

Where P_i is the Boltzmann population of conformer *i*, ΔG_i^{BS2} is the relative estimated Gibbs energy in kJ mol^{-1} of conformer *i*, R is the ideal gas constant (8.314 x 10^{-3} kJ mol^{-1}), T is the temperature of the system (298 K) and g is the degeneracy of conformer *i* (which is 1 for all conformers in this study).

The Boltzmann distribution was used to select the top 99% of conformers to subject to the DFT NMR calculations to calculate scalar coupling constants and magnetic shielding tensors. The NMR properties were computed using the GIAO (gauge-independent atomic orbitals) method using mpw1pw91/6-311g(d,p). The IEFPCM solvation model was used with the solvent chloroform chosen. The additional keyword ‘mixed’ was included to allow for mixing between the core orbitals and the valence orbitals.

4.2 The Calculation of Boltzmann Averaged Chemical Shifts

The Boltzmann averaged magnetic shielding tensors were calculated according to **Equation 4**.

$$\sigma^x = \frac{\sum_i (\sigma_i^x \; x \; p_i)}{\sum p}$$

Equation 4. The calculation of the Boltzmann averaged magnetic shielding tensors.

Where σ^x is the magnetic shielding tensor of nucleus x for conformer i, p_i is the Boltzmann population of conformer i and $\sum p$ is the combined population of conformers involved.

The chemical shifts were calculated according to **Equation 5**.

$$\delta_{calc}^x = \frac{\sigma^{TMS} - \sigma^x}{1 - \frac{\sigma^{TMS}}{10^6}}$$

Equation 5. The calculation of chemical shift from magnetic shielding tensor

Where δ_{calc}^x is the calculated chemical shift of nucleus x, σ^x is the magnetic shielding tensor of nucleus x, σ^{TMS} is the magnetic shielding tensor of the relevant nuclei of TMS calculated in the same solvent and at the same level of theory. For proton this corresponds to 31.92258 and for carbon this corresponds to 189.3625.

The calculated chemical shifts were linearly scaled to remove any systematic errors according to **Equation 6**.

$$\delta_{scaled} = (\delta_{calculated} - intercept)/slope$$

Equation 6. The scaling of calculated chemical shifts

Where the intercept and the slope were obtained by plotting the calculated chemical shifts ($\delta_{calculated}$) against the experimental chemical shifts.

4.3 The Calculation of Boltzmann Averaged Scalar Coupling Constants

The calculated scalar coupling constants for each conformer, both HH and HC, were Boltzmann averaged against their calculated Boltzmann populations according to **Equation 7**.

$$^{n}J_{HX,calc} = \frac{\sum_i (J_{HX,i} \; x \; p_i)}{\sum p}$$

Equation 7. The calculation of the Boltzmann averaged scalar coupling constants

Where $^{n}J_{HX,calc}$ is the Boltzmann averaged scalar coupling constant in Hz, $J_{HX,i}$ is the scalar coupling constant of conformer i, p_i is the population of conformer i and $\sum p$ is the combined population of conformers involved.

4.4 The Calculation of Boltzmann Averaged Interproton Distances

The interproton distances were obtained from the optimised geometries after the geometry optimisation and frequency calculation, performed using mpw1pw91/6-31g(d) basis set. The interproton distances of each conformer were Boltzmann averaged against their calculated Boltzmann populations according to **Equation 8**.

$$r_{Ha-Hb,calc} = \left(\sum_i (r_{Ha-Hb,i})^{-6} \; x \; p_i\right)^{-\frac{1}{6}}$$

Equation 8. The calculation of Boltzmann averaged interproton distances

Where $r_{Ha-Hb,calc}$ is the Boltzmann averaged interproton distance between H_a and H_b, $r_{Ha-Hb,i}$ is the calculated interproton distance between H_a and H_b for conformer i and p_i is the population of conformer i.

4.5 The DFT Calculated Gibbs free energies and Boltzmann Populations for Conformers of 4

The method described above was used to calculate the Gibbs energies of the conformers of **4** and their corresponding Boltzmann populations. The relative potential and Gibbs energies calculated using both basis set 1 and basis set 2, for all conformers under 10 kJ mol^{-1} are provided in **Table 15**. The relative Gibbs energies estimated for basis set 2 were used to calculate the conformer Boltzmann populations.

Table 15. The Calculated Data for **4**

Conformer	ΔE (MM) (kJ mol^{-1})	ΔE (BS1) (kJ mol^{-1})	ΔG (BS1) (kJ mol^{-1})	ΔE (BS2) (kJ mol^{-1})	ΔG_{est} (BS2) (kJ mol^{-1})	Boltzmann Population (%)
2	0.33	2.38	0.00	1.66	0.00	23.99
3	1.57	5.06	0.85	3.73	0.24	21.74
1	0.00	3.18	2.93	1.86	2.34	9.34
4	3.95	3.12	3.62	1.62	2.84	7.62
12	7.08	7.17	5.03	5.84	4.43	4.02
189	18.73	2.64	4.36	2.33	4.77	3.50
14	7.27	7.80	5.47	6.38	4.78	3.49
30	10.39	7.72	5.62	6.39	5.01	3.17
9	5.66	5.32	6.42	4.07	5.90	2.21
18	8.24	5.88	7.10	4.46	6.40	1.81
41	11.82	12.77	7.25	11.19	6.40	1.81
67	13.90	3.91	6.45	3.36	6.63	1.65
8	4.93	6.28	7.32	4.95	6.71	1.60
19	8.76	0.94	5.93	1.38	7.09	1.37
11	7.02	0.00	6.86	0.00	7.59	1.12
147	17.38	6.13	7.56	5.93	8.09	0.92
110	16.24	6.85	8.08	6.33	8.28	0.85
86	14.93	7.33	8.71	7.16	9.26	0.57
188	18.72	7.37	9.30	7.25	9.91	0.44
131	16.87	12.88	9.98	12.32	10.15	0.40

The Boltzmann populations calculated by DFT can be used to plot a 3D-bubble plot of dihedral number, dihedral angle and population (**Figure 30**). The size of each bubble represents the population of the conformer contributing to that dihedral angle. The total population of conformers adopting a linear conformation after DFT is 81%.

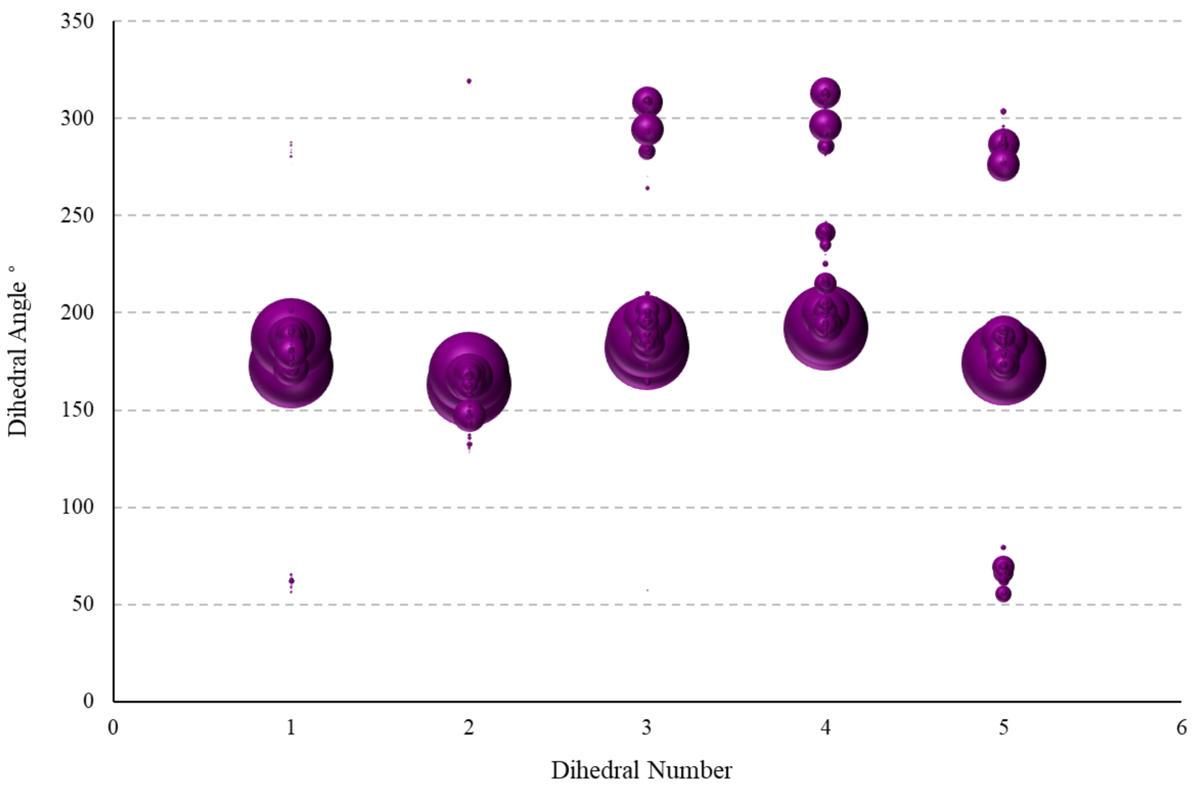

Figure 30. The bubble plot of **4** using populations calculated from DFT calculations.

5. The Acquisition of NMR Parameters in Conformational Analysis

5.1 The Measurement of $^{n}J_{HH}$ Scalar Coupling Constants

$^{n}J_{HH}$ scalar coupling constants were measured either directly from multiplets in the 1D ^{1}H spectrum or from simulations of the ^{1}H NMR spectrum using Spin Simulation in MestreNova. Prior to Spin Simulation, a PSYCHE pureshift ^{1}H spectrum was obtained to provide exact chemical shifts. The measured chemical shifts, estimated *J*-couplings and line widths of the peaks were entered into the Spin Simulation software and the spectrum was simulated (**Table 16**, **Table 17**). The simulated spectrum (**Figure 31**) was superimposed with the experimental spectrum (**Figure 32**) and the *J*-couplings were changed in steps of 0.1 Hz until the best fit between the experimental and simulated spectrum was observed.

Table 16. The parameters used in the partial simulation of the spectrum of **4**

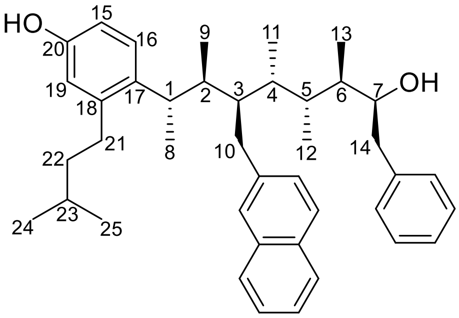

Atom	Chemical Shift	N	Spin	Line Width
1	2.9048	1	½	3.8
2	2.0221	1	½	4.5
3	2.3428	1	½	4.5
4	1.8814	1	½	3.0
5	1.6855	1	½	2.8
6	1.6309	1	½	3.0
7	3.5727	1	½	2.5
8	1.2666	3	½	2.0
9	0.6955	3	½	2.0
10’	3.0223	1	½	3.0
10’	2.6628	1	½	3.5
11	0.8801	3	½	2.0
12	0.6967	3	½	2.5
13	0.9281	3	½	2.5
14’	2.4323	1	½	3.0
14’’	2.2415	1	½	2.5

Table 17. The coupling constants used in the simulation of the spectrum of **4**

	1	2	3	4	5	6	7	8	9	10’	10’’	11	12	13	14’	14’’
1																
2	10.3															
3		2.0														
4			8.2													
5				2.9												
6					9.2											
7						4.6										
8	6.7															
9		6.6														
10’			3.1													
10’’			8.4							15.1						
11				6.9												
12					6.8											
13						6.8										
14’							2.2									
14’’							9.8								13.4	

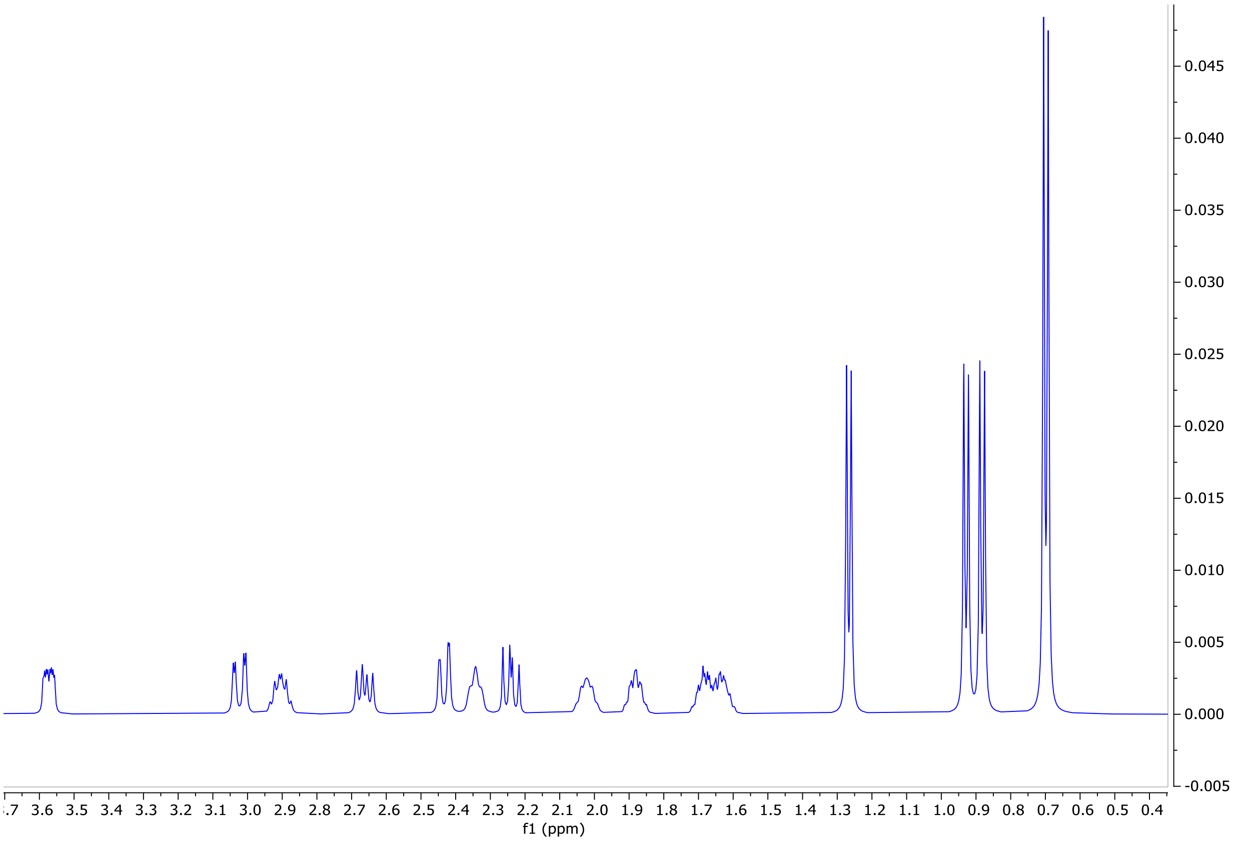

Figure 31. The partial simulation of the spectrum of **4**

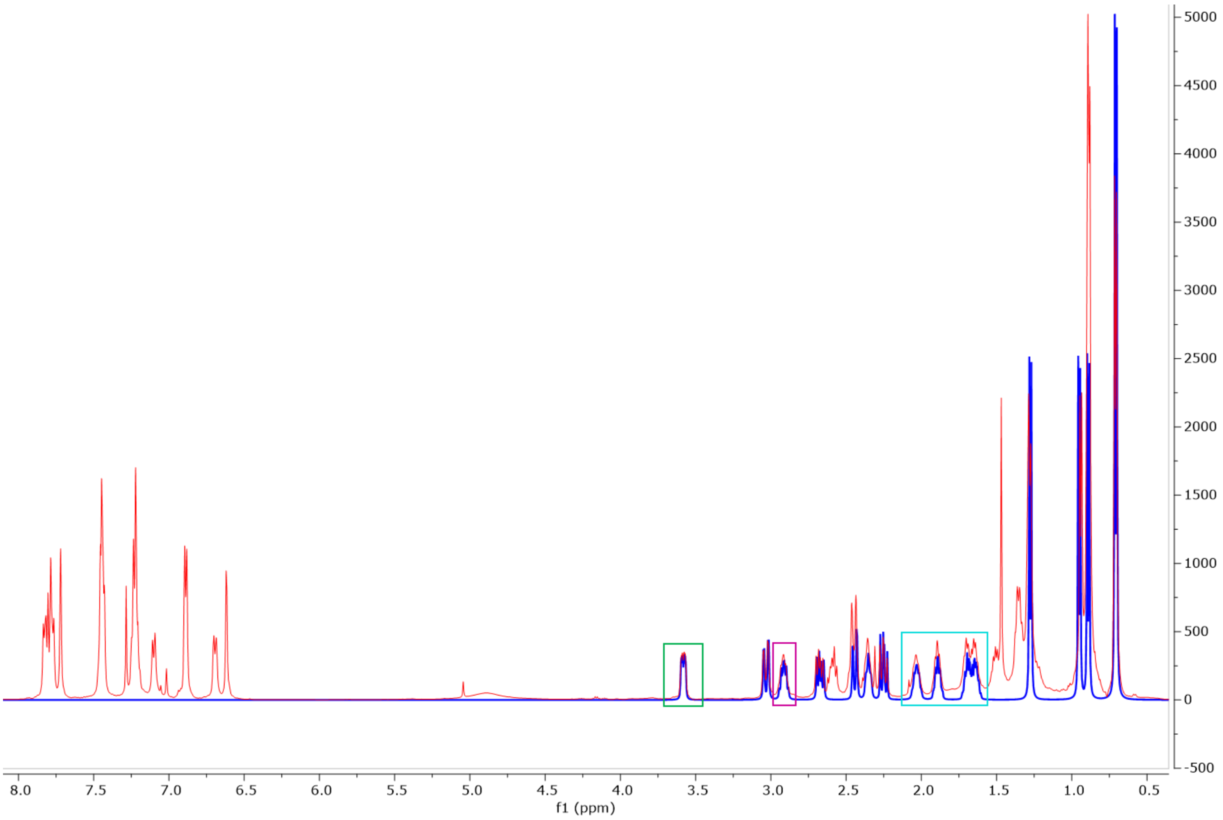

Figure 32. The overlay of the simulated spectrum (blue) and the experimental spectrum (red). Some of the backbone peaks are highlighted by coloured boxes and a zoom in of these peaks is shown in the subsequent figures.

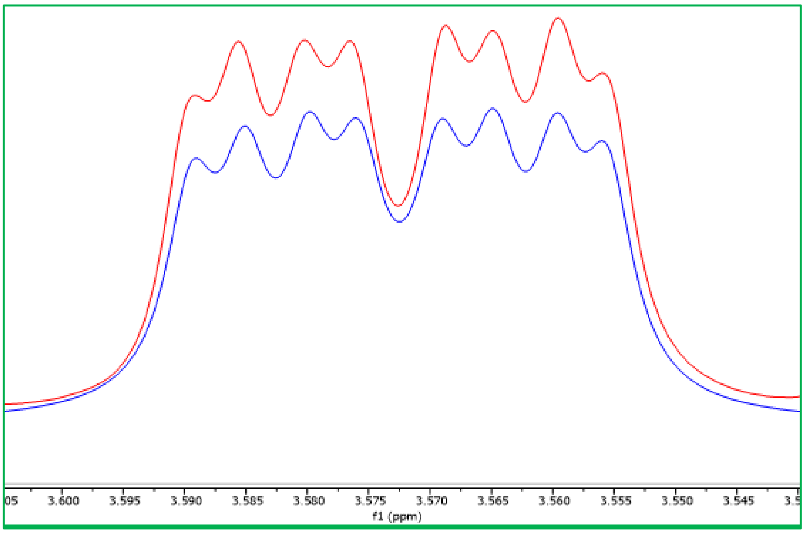

Figure 33. The simulated (blue) and experimental (red) peak of H_7

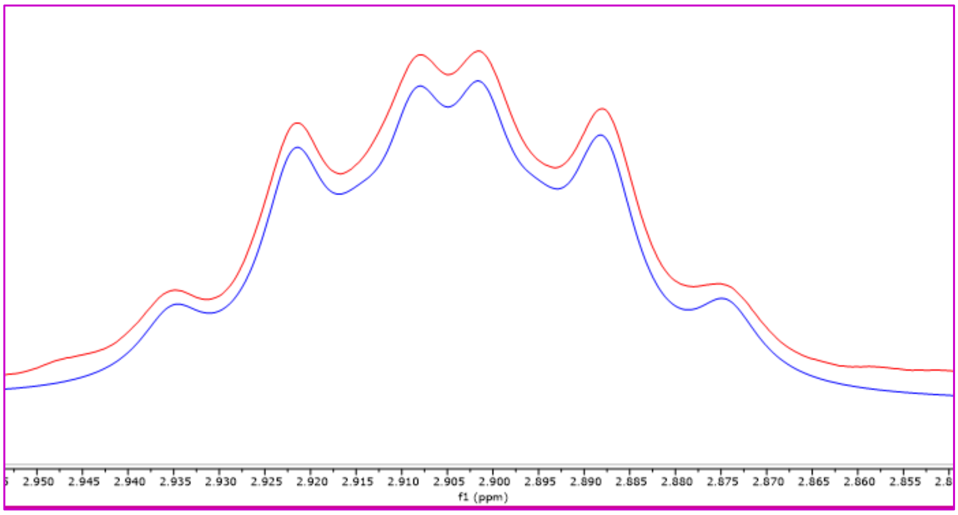

Figure 34. The simulated (blue) and experimental (red) peak of H_1.

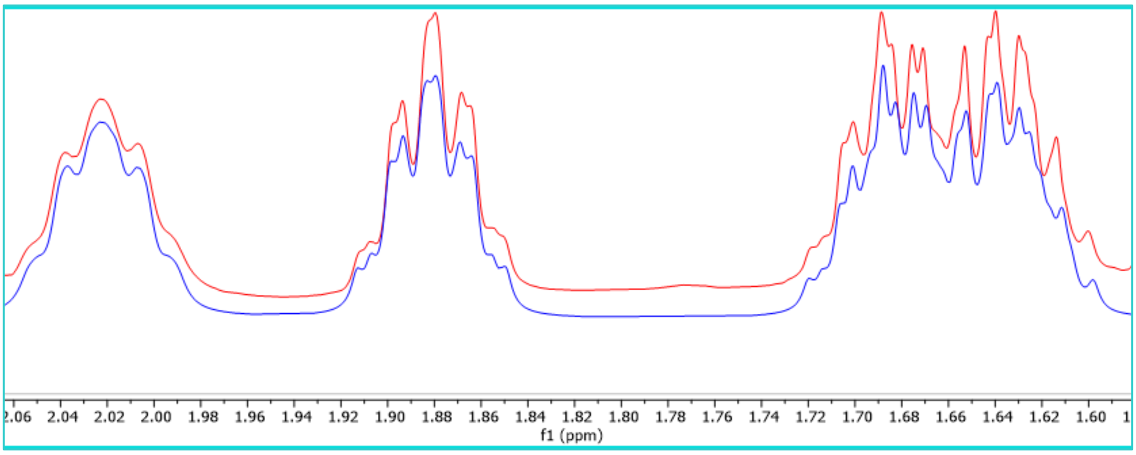

Figure 35. The simulated (blue) and experimental (red) peaks of H_2, H_4, H_5 and H_6.

5.2 The Measurement of $^{n}J_{CH}$ Scalar Coupling Constants

$^{n}J_{CH}$ coupling constants were measured using Accordion In-Phase and Anti Phase (IPAP) HSQMBC NMR Spectra. The In-Phase and Anti Phase spectra were acquired interleaved. The acquisition parameters include: 8 scans, 2048 f1 increments and 16384 f2 data points, f1 spectral width of 180 ppm (22624 Hz), f2 spectral width of 11 ppm (5507 Hz). The spectrum was acquired in $CDCl_3$ with 9 mg in 0.7 mL. The sum (**Figure 36**) and difference (**Figure 37**) were created by adding and subtracting the IP and AP spectra. The offset between the sum and the difference multiplets allows the extraction of the values of $^{n}J_{CH}$ (**Figure 38**). The data was measured using a Bruker AVANCE III HD 500 MHz NMR spectrometer with a 5 mm DCH ^{13}C-^{1}H/D Cryo Probe

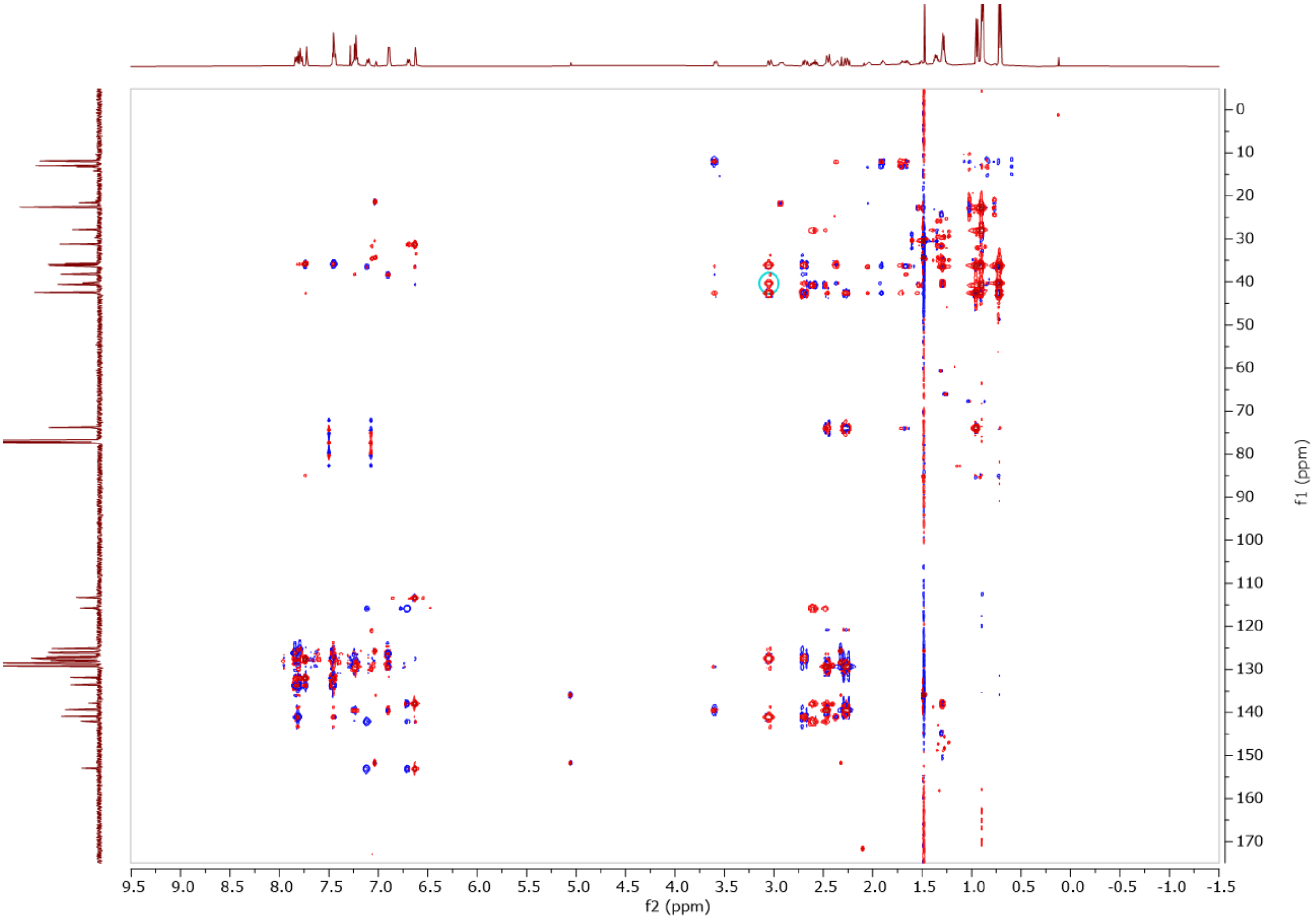

Figure 36. The sum of the IP and AP spectra of the IPAP_HSQMBC of **4.**

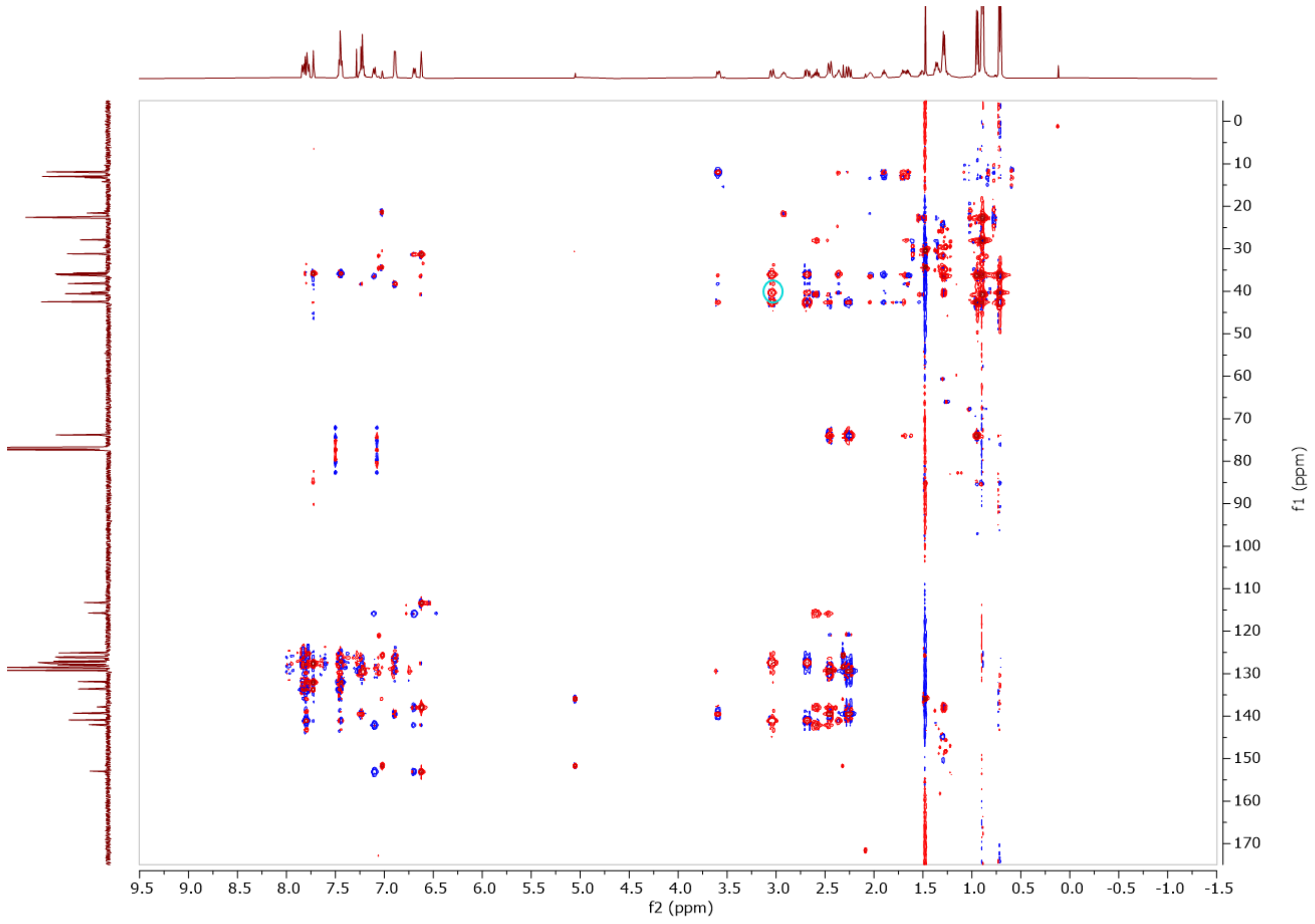

Figure 37. The difference of the IP and AP spectra of the IPAP_HSQMBC of **4**.

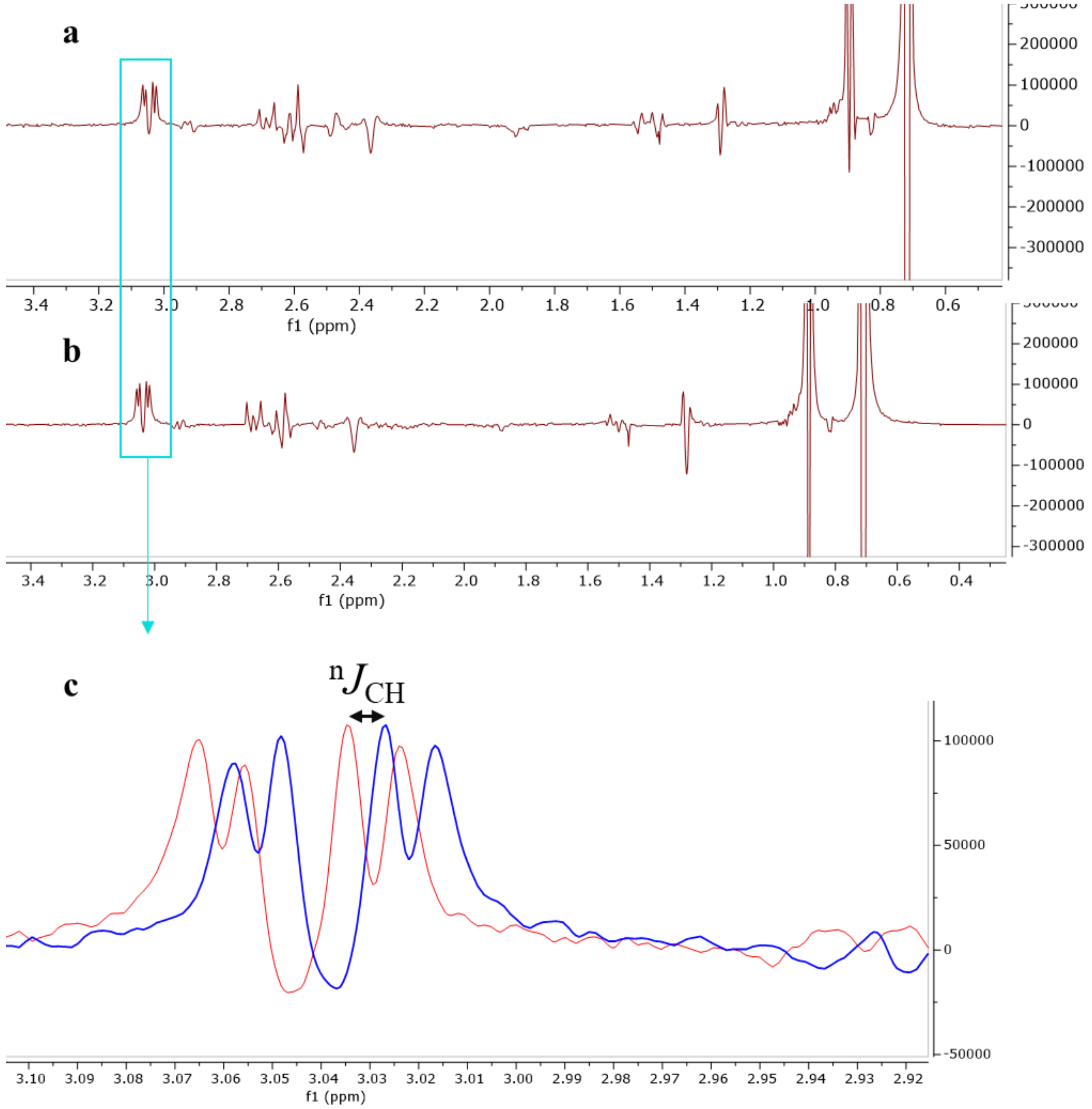

Figure 38. A horizontal 1D trace at 40.3 ppm of both the sum (**a**) and difference (**b**) spectra. **c**) shows the overlay of both the sum and difference 1D-traces for the peak at 3.04 ppm of molecule **4**. The offset between the multiplets is the ^1H-^{13}C coupling constant.

5.3 The Measurement of Quantitative Interproton Distances

The interproton distances were measured using the CSSF-NOESY experiment (256 scans, 2 s relaxation delay, 500 ms mixing time, 30 ppm (15015 Hz) spectral width, 65536 f1 data points). The data was measured using a Bruker AVANCE III HD 500 MHz NMR spectrometer with a 5 mm DCH ^{13}C-^{1}H/D Cryo Probe.

NOE intensities can be used to derive interproton distances because the distance between two atoms is proportional to the NOE intensity between the same atoms. Thus using a known distance as a reference, typically a methylene pair, it is possible to calculate the interproton distance between two atoms using Equation 9.

$$r_{NOE} = r_{ref}\left[\frac{\eta_{NOE}}{\eta_{ref}}\right]^{-1/6}$$

Equation 9. The calculation of interproton distances from NOE intensities.

Where r_{NOE} is the interproton distance between the atoms of interest, r_{ref} is the interproton distance between two reference atoms, η_{NOE} is the NOE intensity between two atoms of interest and η_{ref} is the NOE intensity between two reference atoms. In order to compare all NOE intensities across different 1D-NOESY spectra, differing rates of external relaxation for each proton needs to be corrected for. This can be achieved by applying the PANIC method described by Macura.[12] By setting the irradiated peak to 1000 in all selective 1D-NOSY experiments, differing rates of external relaxation is corrected for.

For **4**, the reference distance was that between $H_{10''}$ and $H_{10'}$ (1.74 Å). The reference distance is incrementally changed to reduce the overall MAD for the dataset. This means that there may be a small %deviation between the experimental reference distance and its calculated value, however the overall MAD will be reduced. Thus, using this reference distance and NOE intensity, all measured NOE intensities can be used to derive an interproton distance (**Figure 39**).

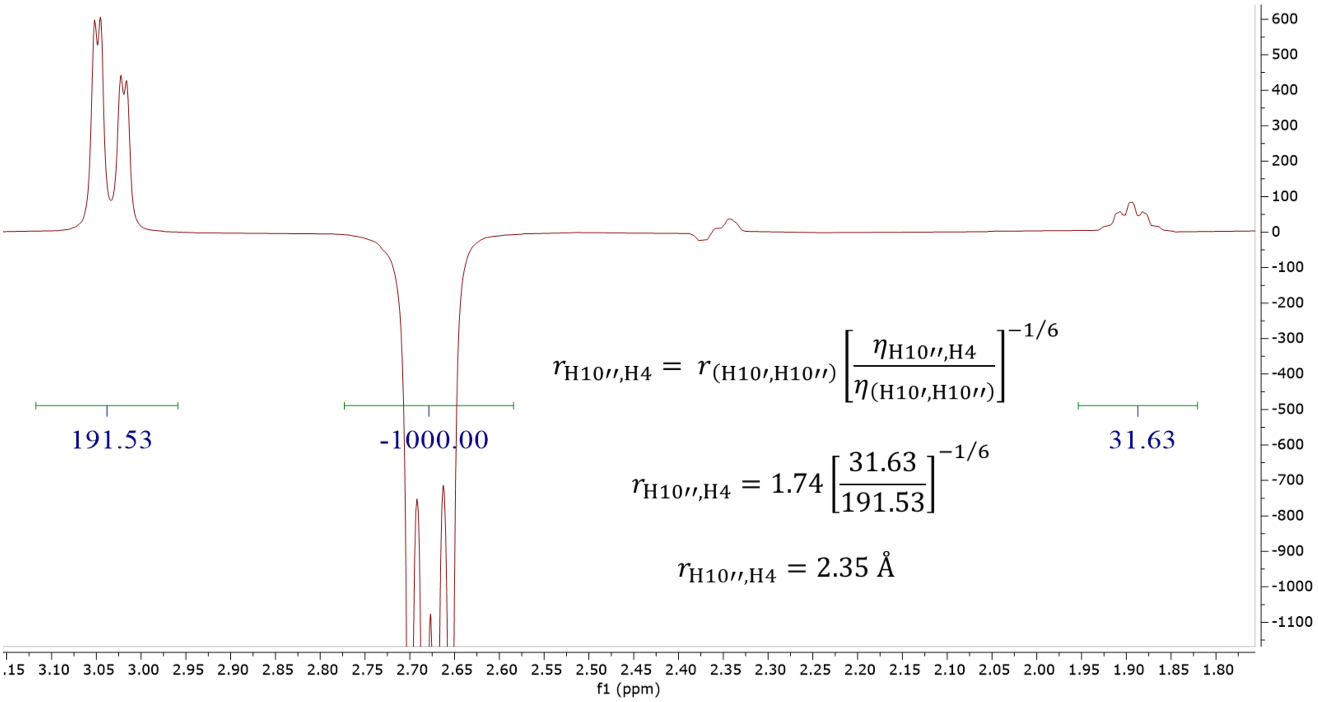

Figure 39. Deriving the $H_{10''}$-H_4 interproton distance.

6. The Conformational Analysis of 4 using NMR Spectroscopy and Computation

The Boltzmann averaged NMR parameters, calculated as described in the previous section, were then compared to the experimentally measured NMR parameters. The quality of the fit between the two sets of data was assessed using the mean absolute deviation (MAD) and Standard Deviation (SD).

The MAD was calculated using either Equation 10 when calculating the %deviation between experimental and calculated interproton distances or Equation 11 when calculating the deviation between experimental and calculated scalar coupling constants and chemical shifts.

$$\%\text{Deviation} = \left(\frac{r_{calc} - r_{exp}}{r_{calc}}\right) x\ 100$$

Equation 10. The calculation of %deviation for interproton distances

$$\text{Deviation} = \left(\delta/J_{calc} - \delta/J_{exp}\right)$$

Equation 11. The calculation of deviation for scalar couplings or chemical shifts.

6.1 A Comparison of Experimental and Calculated $^nJ_{HH}$ Scalar Coupling Constants

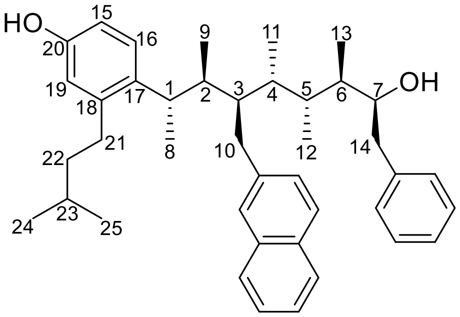

Table 18. A comparison of the experimentally derived $^3J_{HH}$ scalar coupling constants to those calculated by DFT calculations for **4**.

Proton	Proton	Experimental Coupling (Hz)	Calculated Coupling (Hz)	Deviation (Hz)
1	2	10.3	10.7	0.4
2	3	2.0	2.6	0.6
3	4	8.2	9.3	1.1
4	5	2.9	3.2	0.3
5	6	9.2	9.4	0.2
6	7	4.6	4.9	0.3
			**Mean Absolute Deviation (Hz)**	**0.48**
			**Standard Deviation (Hz)**	**0.33**

6.2 A Comparison of Experimental and Calculated ${}^{n}J_{CH}$ Scalar Coupling Constants

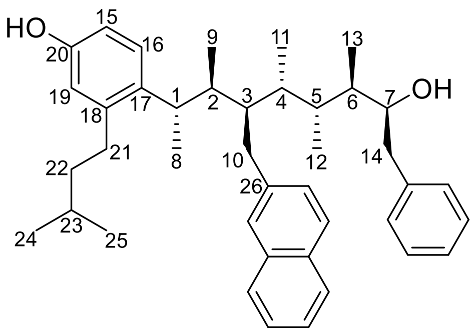

Table 19. A comparison of the experimentally derived ${}^{n}J_{CH}$ scalar coupling constants to those calculated by DFT calculations for **4**.

Proton	Carbon	Experimental Coupling (Hz)	Calculated Coupling (Hz)	Deviation (Hz)
1	9	2.5	1.7	-0.7
1	8	4.5	4.3	-0.2
2	8	3.3	3.3	0.0
2	1	5.4	5.3	-0.1
3	11	3.2	2.4	-0.8
3	9	6.1	6.2	0.1
3	4	4.8	5.0	0.2
3	2	4.9	4.7	-0.2
3	26	4.3	4.8	0.4
4	12	5.6	5.5	-0.1
4	2	2.7	2.2	-0.5
4	11	4.7	4.3	-0.4
4	5	5.5	4.7	-0.8
5	7	2.2	2.4	0.2
5	12	4.5	4.3	-0.2
6	12	2.6	2.6	0.1
6	14	5.0	5.2	0.2
6	13	3.7	3.8	0.1
6	5	5.4	4.7	-0.7
7	5	2.0	1.3	-0.7
7	13	4.4	4.5	0.1
7	6	2.5	2.2	-0.3
10’	4	5.7	6.6	0.9
10’	2	3.7	4.6	1.0
10’	3	4.4	4.7	0.3
10’	26	6.7	8.0	1.3
10’’	4	4.5	5.4	0.8
10’’	3	4.6	4.5	-0.1
10’’	26	5.8	5.4	-0.4
13	5	4.1	4.2	0.1
13	6	3.9	3.8	0.0
13	7	4.3	4.3	0.0
14’	6	2.2	2.2	0.1
14’	7	6.8	6.9	0.1

14’’	6	1.3	0.8	-0.5
14’’	7	1.9	1.8	-0.1
15	19	4.7	4.9	0.2
15	20	2.7	2.6	0.0
16	1	3.5	3.7	0.2
16	20	10.4	10.3	-0.1
19	21	4.7	5.3	0.7
19	1	1.4	0.6	-0.8
19	15	5.0	5.3	0.3
19	20	3.1	3.9	0.8
21’	23	2.6	2.3	-0.3
21’	22	4.9	5.0	0.1
21’	19	5.8	6.1	0.3
			**Mean Absolute Deviation (Hz)**	**0.35**
			**Standard Deviation (Hz)**	**0.48**

6.3 A Comparison of Experimental and Calculated Interproton Distances

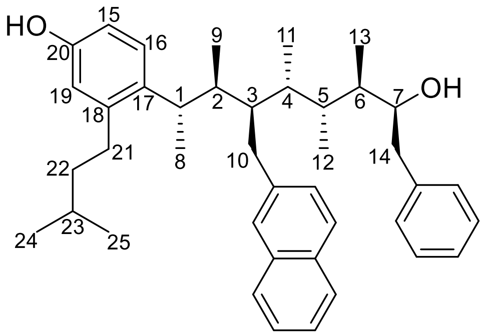

Table 20. A comparison between the experimentally derived interproton distances from 1D-NOE spectroscopy to those calculated by DFT calculations for **4**.

Irradiated ^{1}H (Ha)	Observed ^{1}H (Hb)	Relative η_{AB}	Experimental r_{AB} (Å)	Calculated r_{AB} (Å)	Deviation (%)
7	14’	17.18	2.60	2.49	-4.42
	14’’	6.77	3.04	3.04	-0.02
	4	14.04	2.69	3.11	15.66
	13	2.37	3.62	3.59	-0.63
	12	12.67	2.74	2.77	1.40
10’	1	60.52	2.11	2.08	-1.48
	10’’	182.70	1.75	1.74	-0.64
	3	19.99	2.54	2.67	5.28
1	10’	34.86	2.31	2.08	-10.13
	20’	40.61	2.25	2.19	-2.84
	20’’	21.76	2.50	2.45	-2.20
	3	11.73	2.77	2.91	5.03
	2	5.66	3.13	3.02	-3.56
**10’’**	**10’ (r_{ref})**	**191.53**	**1.74**	**1.74**	0.15
	4	31.63	2.35	2.41	2.53
3	10’	15.69	2.64	2.67	1.11
	1	13.11	2.72	2.91	7.00
	2	25.06	2.44	2.48	1.54
	4	13.14	2.72	2.83	4.08
	8	18.88	2.56	2.51	-1.97
	11	4.27	3.28	3.27	-0.19
2	16	48.81	2.19	2.27	4.10
	3	22.77	2.48	2.48	-0.07
	8	6.96	3.02	2.93	-3.18
	11	15.60	2.64	2.68	1.46
4	7	14.60	2.67	3.11	16.42
	10’’	20.57	2.52	2.41	-4.56
	3	11.13	2.80	2.83	1.24
	13	15.17	2.66	2.75	3.49
				**Mean Absolute Deviation (%)**	**3.67**
				**Standard Deviation (%)**	**5.42**

6.4 A Comparison of Experimental and Calculated Chemical Shifts

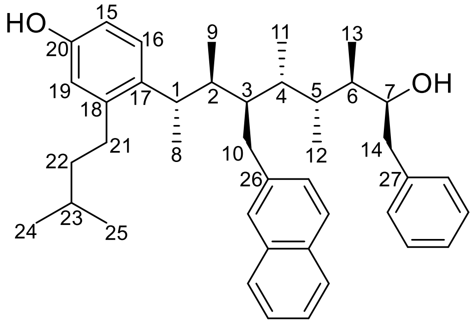

Table 21. A comparison of the experimental δ_H to those calculated by DFT calculations for **4**.

Proton	Experimental δ_H (ppm)	Magnetic Shielding Tensor	Calculated δ_H (ppm)	Scaled δ_H (ppm)	Deviation (ppm)
1	2.91	28.80	3.13	3.00	0.09
2	2.03	29.79	2.14	2.05	0.02
3	2.35	29.34	2.59	2.48	0.13
4	1.88	29.84	2.08	2.00	0.12
5	1.70	30.23	1.69	1.62	-0.08
6	1.64	30.21	1.71	1.64	0.00
7	3.57	28.67	3.25	3.12	-0.45
8	1.27	30.39	1.54	1.47	0.20
9	0.70	31.23	0.69	0.66	-0.04
10’	3.02	28.54	3.38	3.24	0.22
10’’	2.67	29.28	2.65	2.54	-0.13
11	0.88	31.03	0.90	0.86	-0.02
12	0.70	31.44	0.49	0.47	-0.23
13	0.93	30.89	1.04	1.00	0.07
14’	2.24	29.66	2.26	2.17	-0.07
14’’	2.45	29.70	2.23	2.14	-0.31
15	6.68	25.08	6.85	6.57	-0.11
16	7.09	24.43	7.50	7.19	0.10
19	6.60	24.95	6.98	6.70	-0.10
21’	2.59	29.03	2.90	2.78	0.19
21’’	2.45	29.36	2.56	2.46	0.01
22’	1.34	30.43	1.49	1.43	0.09
22’’	1.34	30.50	1.43	1.37	0.03
23	1.50	30.30	1.63	1.56	0.06
24/25	0.87	31.00	0.93	0.89	0.02
				**Mean Absolute Deviation (ppm)**	**0.11**
				**Standard Deviation (ppm)**	**0.16**

Table 22. A comparison of the experimental δ_C to those calculated by DFT calculations for **4**.

**Carbon**	**Experimental δ_C (ppm)**	**Magnetic Shielding Tensor**	**Calculated δ_C (ppm)**	**Scaled δ_C (ppm)**	**Deviation (ppm)**
1	36.5	149.09	40.3	37.0	0.5
2	40.3	144.69	44.7	41.3	1.0
3	42.6	143.36	46.0	42.5	-0.1
4	36.0	148.10	41.3	38.0	2.0
5	36.3	149.64	39.7	36.5	0.2
6	42.6	144.86	44.5	41.1	-1.5
7	74.0	113.05	76.3	71.7	-2.3
8	21.7	164.82	24.5	21.9	0.2
9	13.4	175.48	13.9	11.6	-1.8
10	35.8	148.60	40.8	37.5	1.7
11	12.1	175.66	13.7	11.4	-0.7
12	13.1	175.38	14.0	11.7	-1.4
13	12.0	175.53	13.8	11.6	-0.4
14	38.3	147.95	41.4	38.1	-0.2
15	113.4	73.01	116.4	110.2	-3.2
16	127.5	55.28	134.1	127.3	-0.2
17	138.0	45.53	143.9	136.7	-1.3
18	142.1	39.14	150.2	142.8	0.7
19	115.8	69.17	120.2	113.9	-1.9
20	153.1	28.42	161.0	153.2	0.1
21	31.3	152.29	37.1	33.9	2.6
22	40.7	144.64	44.7	41.3	0.6
23	28.0	155.76	33.6	30.6	2.6
24/25	22.7/22.7	164.47/165.67	24.9/23.7	22.2/21.1	-0.5/-1.6
26	141.0	39.55	149.8	142.5	1.5
27	139.4	42.42	147.0	139.7	0.3
				**Mean Absolute Deviation (ppm)**	**1.14**
				**Standard Deviation (ppm)**	**1.46**

7. The Binding of the Designed Inhibitors to Mdm2 using ^{1}H-^{15}N HSQC Spectroscopy

7.1 The Expression and Purification of ^{15}N-Mdm2

Sequence Used:

MHHHHHHGKPIPNPLLGLDSTENLYFQGIDPFTLVRPKPLLLKLLKSVGAQKDTYTMKEVLFYLGQY
IMTKRLYDEKQQHIVYCSNDLLGDLFGVPSFSVKEHRKIYTMIYRNLVVVNQQESSDSGTSVSEN

A fragment of the N-terminal domain of human Mdm2 (residues 27-127) was cloned into the pET-151 (Thermofisher) vector and expressed in the *E. coli* BL21(DE3) strain using standard protocols. Briefly, three colonies of the transformed cells were used to inoculate 50 mL of LB media supplemented with 100 µg/ml ampicillin and incubated at 37 °C overnight. 2 mL of the seed broth was used to inoculate flasks containing 200 mL of LB media supplemented with 200 µg/ml of ampicillin. Flasks were incubated at 37 °C until an OD_{600} of 1.0-1.4 was reached. Cell cultures were then cooled to 16 °C and induced with 200 µM of IPTG. Protein expression was maintained overnight at 16 °C. Cells were harvested by centrifugation at 6000 rpm at 4 °C. The cell pellet was resuspended in 20 mL of column buffer (50 mM Tris base, 0.5 M NaCl, pH 8.0) followed by flash freezing in liquid nitrogen. The cell suspension was stored at -20 °C until ready for purification. Prior to purification, the cells were lysed at -10 °C by sonication for a total time of 10 minutes consisting of cycles of 5 seconds sonication followed by 20 seconds rest. Cells were clarified by centrifuged at 15000 rpm and the cell lysate was purified using a HisTrap HP 5 mL column loaded with Ni^{2+} . Protein was eluted via a linear gradient from 5 to 100% elution buffer (50 mM Tris buffer at pH 8.0 with 0.3 M NaCl and 0.8 M imidazole) and collected in 1 mL fractions. The pure fractions were analysed by SDS PAGE and those containing the protein of interest were combined. Due to the presence of a free cysteine, Mdm2 can form disulfide dimers (~30 kDa) during purification that can monomerized with reducing agents once pure.

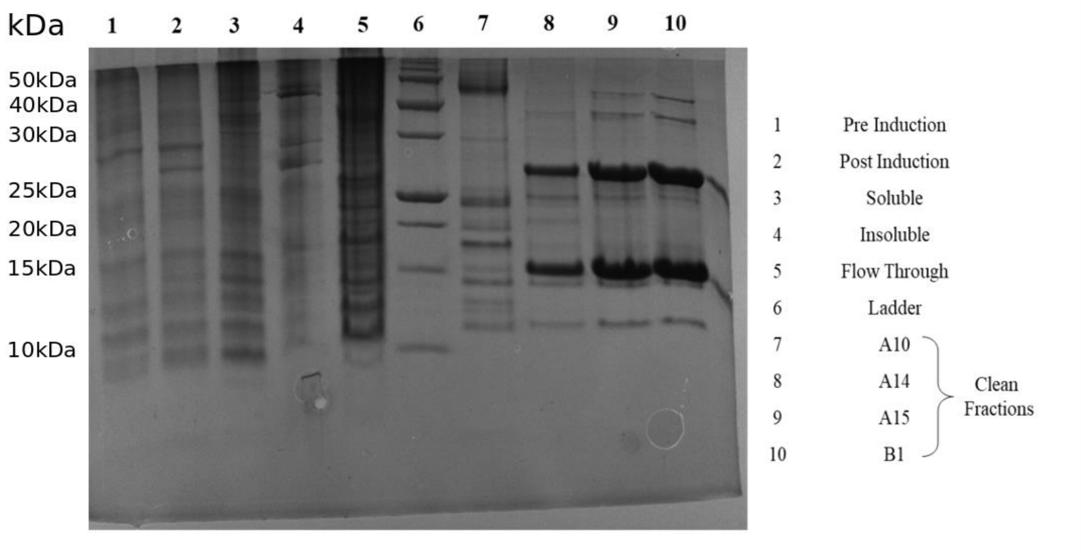

Figure 40. The SDS-PAGE results following the affinity purification of His_6-Mdm2 using a HisTrap HP 5 mL Ni column.

Initially, TEV protease was used to cleave the His_6-tag, but in all the conditions used this was unsuccessful. However, as the tag aided protein solubility and the ^1H-^{15}N HSQC of His_6-Mdm2 is well-dispersed with most of the backbone resonances readily assignable (Figure 40), the tag was left on the protein for the NMR experiments. To obtain >90% pure protein the combined fractions from the first HisTrap HP 5 mL Ni column were subjected to a second purification using a HiLoad 26/60 Superdex 75 prep grade column, equilibrated with 20 mM Tris, 250 mM NaCl, 1 mM TCEP at pH 7.5. Gel electrophoresis was used to confirm the fractions containing pure Mdm2.

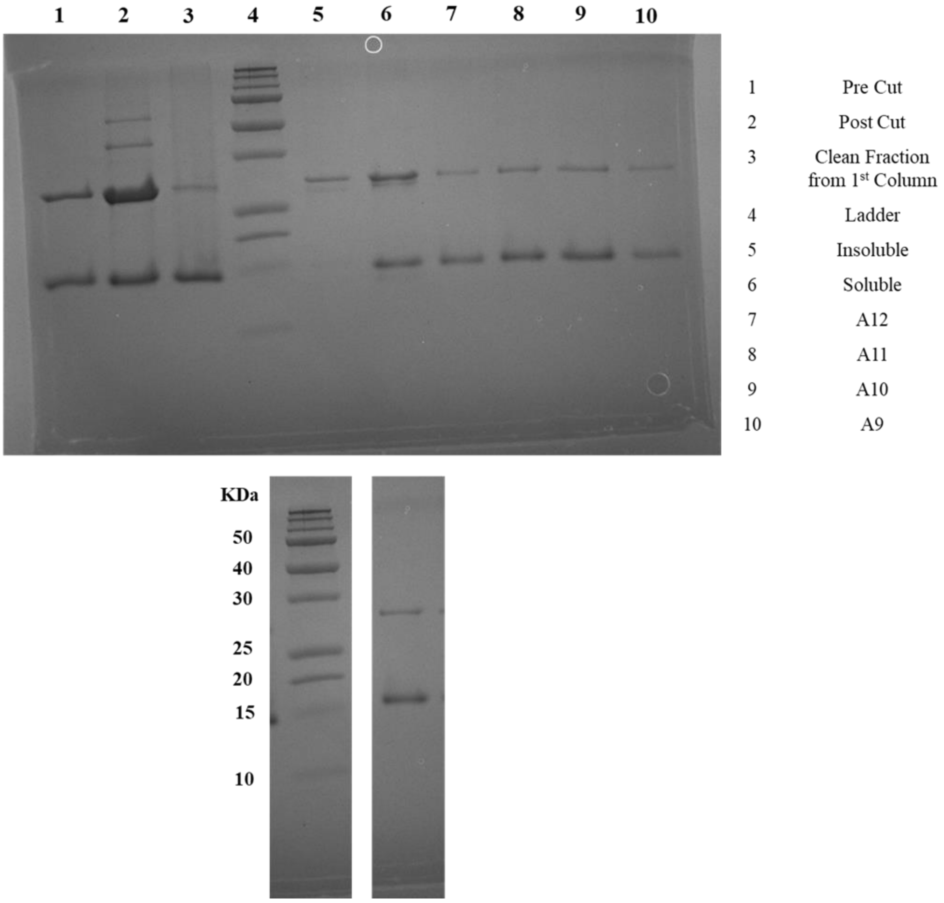

Figure 41. The SDS-PAGE results following the second purification of Mdm2 using a HiLoad 26/60 Superdex 75 prep grade column.

Samples were desalted for MALDI mass spectrometry using a C_4 ZipTip™ (Millipore). The ZipTip was activated with 3 10 µL washes of 50% acetonitrile in water and equilibrated with five 10 µL washes of 0.1% TFA in water. A 10 µL sample was then loaded onto the ZipTip with 15 washes. The protein was desalted with ten 10 µL washes with 5% MeOH, 0.1% TFA in water and discarded to waste. Finally, the sample was eluted with 10 µL of 70% acetonitrile, 0.1% TFA in water. MALDI mass spectrometry confirm that Mdm2 had been successfully expressed, and a M_w of 15233.0 Da was observed, corresponding to the expected M_w of the His_6-tagged Mdm2. Obs: 15233.00 Da Expt: 15232.51 Da.

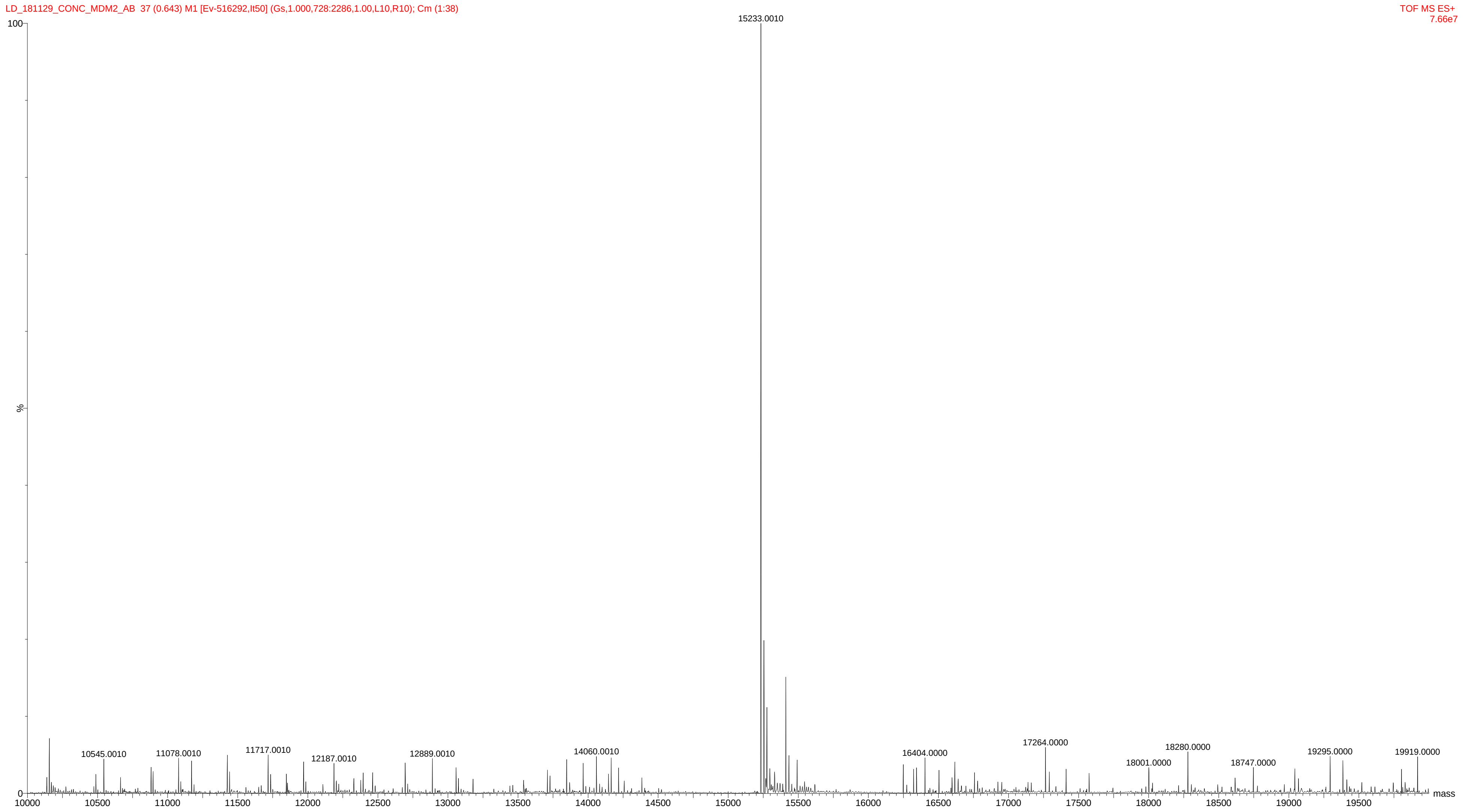

Figure 42. The results from the MALDI mass spectroscopy of Mdm2, showing the Mw found.

Following the successful expression of unlabelled Mdm2, ^{15}N-labelled Mdm2 was expressed. Briefly, three colonies of transformed *E. coli* BL21 (DE3) cells incubated overnight at 37 °C in 50 mL of LB media with100 ug/ml of ampicillin. 2 mL of the seed broth was then used to inoculate flasks containing 200 mL of LB media and 400 μL of ampicillin. and incubated at 37 °C until an OD_{600} of 1.0-1.4 was reached. The Cells were harvested by centrifugation at 6000 rpm and washed twice with sterile M9 media before being resuspended in 1L of sterile M9 media supplemented with 450 μL of ampicillin, $^{15}NH_4Cl$ (1 g/L), glucose (3 g/L), $MgSO_4$ (200 μg/mL) and a trace metal mix (150 μL). The cells were then incubated at 37 °C for 1 hour before the temperature was dropped to 16 ^{o}C. The culture was then induced with IPTG (200 μM) at 16 °C overnight. Cells were harvested by centrifugation at 6000 rpm and purified as described for the unlabelled Mdm2. MALDI mass spectroscopy confirmed that full ^{15}N labelling had occurred. Obs: 15411.0 Expt: 15414.1

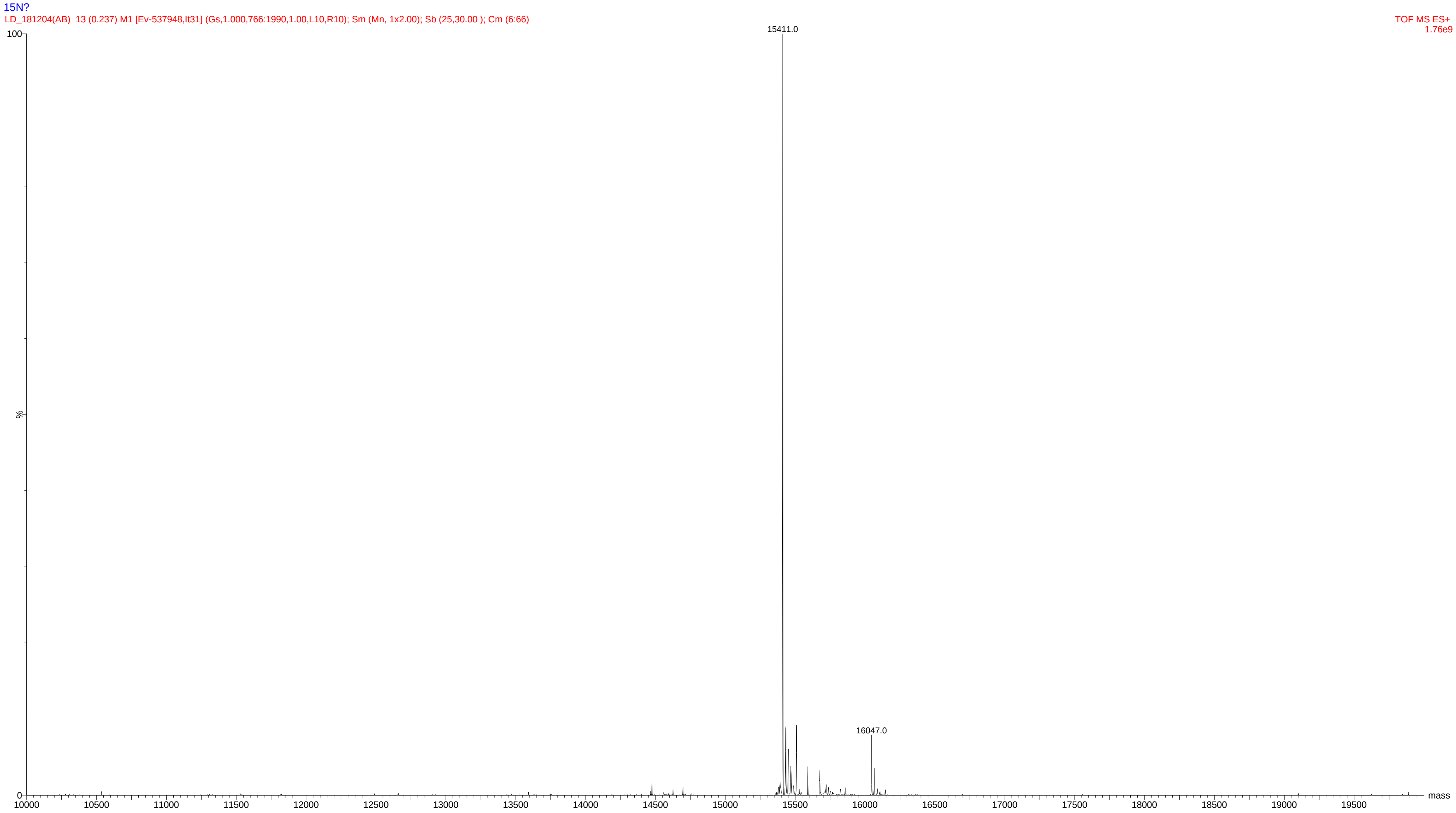

Figure 43. The results from the MALDI mass spectroscopy of ^{15}N-Mdm2, showing the M_w found.

NMR spectroscopy was performed to confirm the purity of Mdm2 and that it was folded. A 2D- ^{1}H-^{15}N TROSY of Mdm2 was acquired at a concentration of 130 µM on a 700 MHz spectrometer fitted with a 1.7 mm inverse triple resonance micro-Cryo Probe at 20 °C. The protein was solubilised in a 10 mM $NaHPO_4$ solution with 150 mM NaCl at pH 7.4. 10% D_2O was added to the sample to provide a lock signal. The total sample volume was 50 µl using 1.7 mm NMR tubes. The acquisition parameters include: 8-32 scans, 256 f1 increments and 512 f2 data points, f1 spectral width of 25 ppm (1774 Hz), f2 spectral width of 12 ppm (8403 Hz). Spectra were processed and analysed in MestreNova v14.0. Spectra were zero filled to 4096 data points and manually phase corrected using the largest peak as the pivot point. The acquired TROSY was partially assigned using previously reported assignments of Mdm2 in the literature.[13,14]

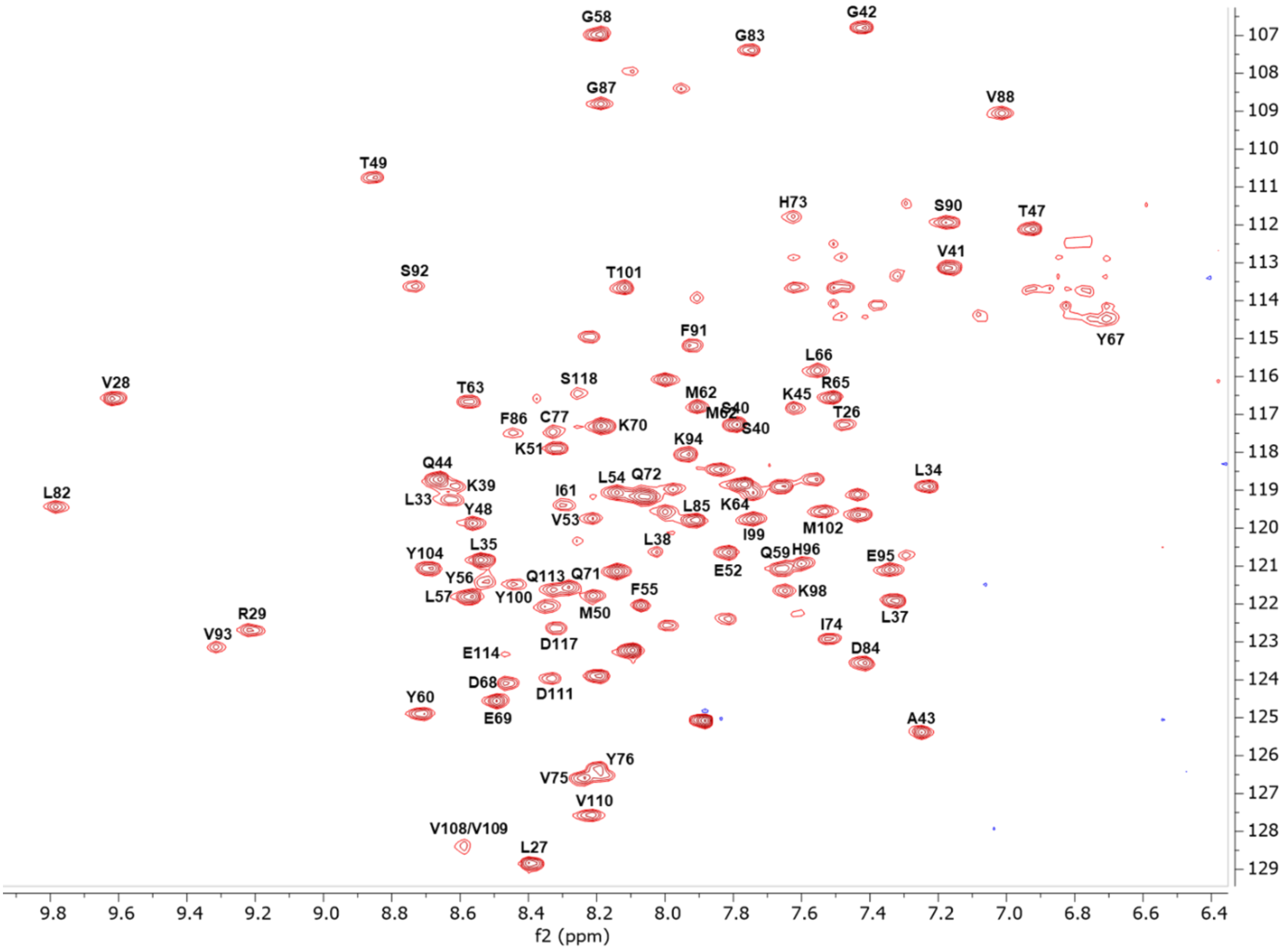

Figure 44. The ^{1}H-^{15}N-TROSY of ^{15}N-labelled Mdm2 at pH 7.4 and 20 °C with the assignments highlighted.

Table 23. A comprehensive list of the resolved peaks with their assignments

F1 (ppm)	F2 (ppm)	Assignment
8.20	118.35	14Thr
7.51	116.50	26Thr
8.45	128.15	27Leu
9.66	116.10	28Val
9.25	121.99	29Arg
8.71	118.64	33Leu
7.30	118.35	34Leu
8.56	120.22	(35Leu/56Tyr)
7.35	121.33	37Leu
8.18	120.52	38Leu
8.66	118.33	39Lys
7.83	116.57	40Ser
7.23	112.63	41Val
7.46	106.35	42Gly
7.31	124.78	43Ala
8.70	118.06	44Gln
7.67	116.26	45Lys
6.97	111.17	47Thr
8.59	120.72	48Tyr
8.89	110.11	49Thr
8.25	121.14	50Met
7.82	119.93	52Glu
8.30	118.93	54Leu
8.17	122.73	55Phe
8.63	121.30	57Leu
8.26	106.37	58Gly
7.82	121.83	59Gln
8.76	124.15	60Tyr
8.58	119.18	61Ile
7.92	116.17	62Met
8.63	116.04	63Thr
7.56	116.07	65Arg
7.61	115.17	66Leu
6.75	113.96	67Tyr
8.49	123.74	68Asp
8.54	124.00	69Glu
8.21	116.67	70Lys
8.29	120.92	71Gln
8.29	119.39	72Gln
7.96	113.50	73His
7.58	122.30	74Ile
8.27	125.89	75Val
8.24	125.73	76Tyr
8.38	117.27	77Cys
7.62	118.11	80Asp

9.81	118.83	82Leu
7.77	106.86	83Gly
7.49	123.04	84Asp
8.00	119.38	85Leu
8.36	116.99	86Phe
8.24	108.39	87Gly
7.05	108.50	88Val
7.21	111.25	90Ser
8.27	114.43	91Phe
8.71	112.86	92Ser
9.37	122.42	93Val
7.97	117.28	94Lys
7.34	120.37	95Glu
7.69	120.48	96His
7.73	121.32	98Lys
7.74	119.05	99Ile
8.39	121.11	100Tyr
8.12	112.73	101Thr
7.59	120.05	102Met
8.73	120.22	104Tyr
8.67	127.85	(108Val/109Val)
8.29	127.29	110Val
8.25	123.42	111Asn
8.37	122.07	117Asp
8.31	115.93	118Ser
7.45	119.18	None
7.49	118.78	None
7.71	118.35	None
7.77	118.46	None
7.80	118.18	None
7.88	117.90	None
7.97	124.49	None
7.99	114.62	None
8.03	115.45	None
8.04	118.96	None
8.05	122.01	None
8.09	118.63	None
8.12	118.45	None
8.14	121.59	None
8.32	116.80	None
8.39	121.42	None
8.40	123.48	None

7.2 The binding of 15 by TROSY spectroscopy

A 2D-^{1}H-^{15}N TROSY of ^{15}N-labelled Mdm2 was acquired at a concentration of 50 µM on a 700 MHz spectrometer fitted with a 1.7 mm inverse triple resonance micro-Cryo Probe at 20 °C. The protein was solubilised in a 20 mM Tris buffer with 250 mM NaCl at pH 7.5. 10% D_2O was added to the sample to provide a lock signal. 10% Acetone-d_6 was added for ligand solubility. The total sample volume was 50 µl using 1.7 mm NMR tubes. The acquisition parameters include: 512 scans, 256 f1 increments and 512 f2 data points, f1 spectral width of 25 ppm (1774 Hz), f2 spectral width of 12 ppm (8403 Hz). A 2D-^{1}H-^{15}N TROSY of Mdm2 (50 µM) with ligand **15** (70 µM) in the same buffer conditions was acquired with the same acquisition parameters. Spectra were processed and analysed in MestreNova v14.0. Spectra were zero filled to 4096 data points and manually phase corrected using the largest peak as the pivot point.

Chemical shift perturbations (CSP's) were calculated as a Euclidean distance using **Equation 12** with a value of α=0.14. The value of α was calculated from the ratio of the chemical shift range (in ppm) of ^{1}H to that of ^{15}N (3.08/22.1 = 0.139).

$$\delta_{obs} = \sqrt{\frac{1}{2}[\delta_H^2 + (\alpha\delta_N^2)]}$$

Equation 12. The calculation of Euclidean distance

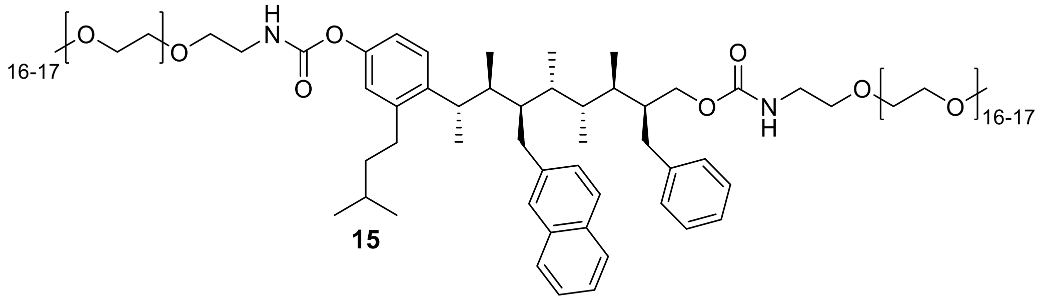

15

Scheme 1. The structure of Ligand **15**

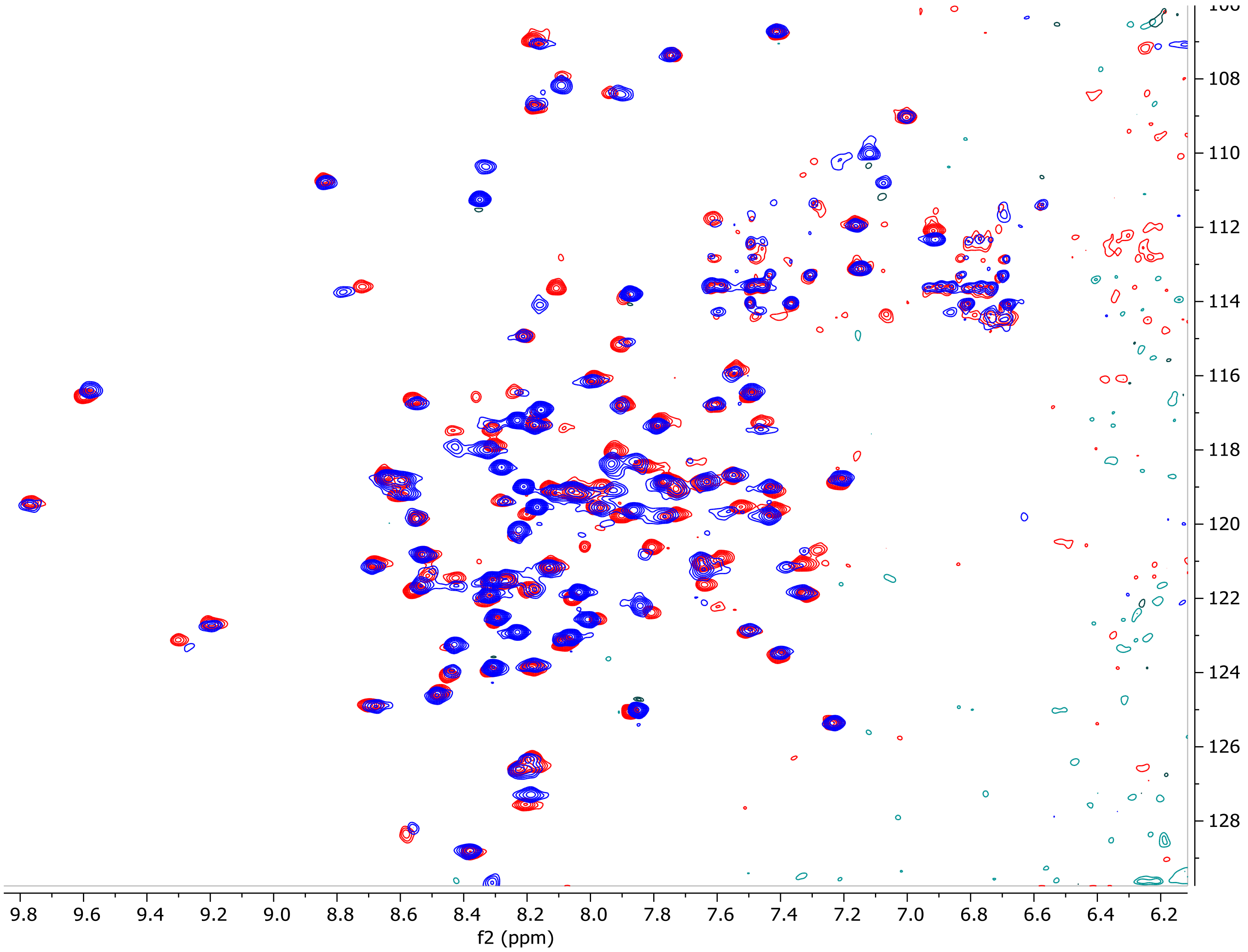

Figure 45. The ^{1}H-^{15}N-TROSY of 15N-labelled Mdm2 (red) and Mdm2+ligand **15** (blue) acquired at pH 7.5 and 20 °C.

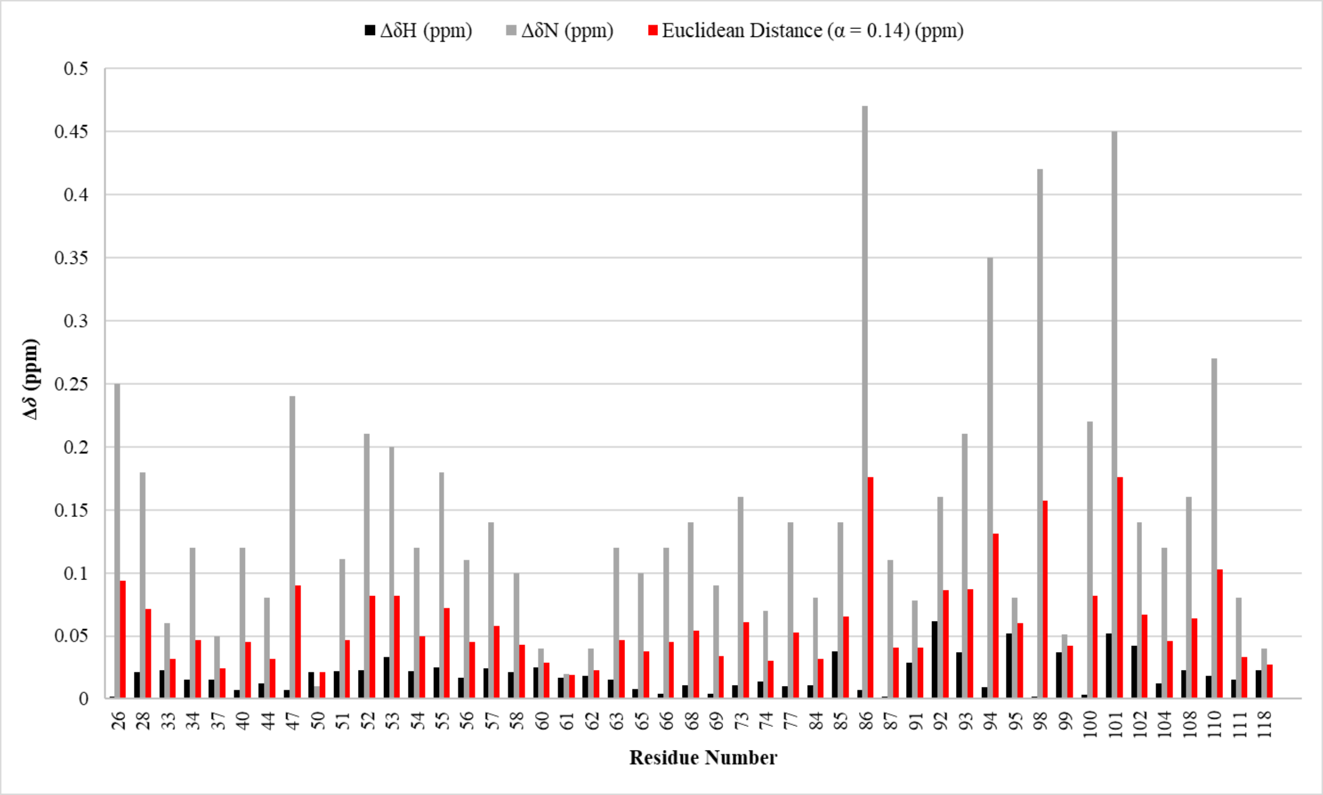

Figure 46 A graph to show the ^{1}H and ^{15}N CSP and the corresponding Euclidean Distance upon binding of ligand **15** to Mdm2

7.3 The Estimation of K_d using ^{1}H-^{15}N SOFAST HMQC for 15

A 2D ^{1}H-^{15}N SOFAST HMQC of Mdm2 at a concentration of 70 µM with five concentrations of ligand **15** ranging from 17.5 to 280 µM was acquired on a 700 MHz spectrometer fitted with a 1.7 mm inverse triple resonance micro-Cryo Probe 20 °C using the sofasthmqc sequence. The protein was solubilised in a 20 mM Tris buffer with 250 mM NaCl at pH 7.5. 10% D_2O was added to the sample to provide a lock signal. 10% Acetone-d_6 was added for ligand solubility. A 10 mM stock solution of ligand **15** in acetone-d_6 was prepared and the required volume was added to the protein sample to give final ligand concentrations of 17.5 µM, 35 µM, 70 µM, 140 µM and 280 µM. The total sample volume was 50 µL using 1.7 mm NMR tubes. The acquisition parameters include: 64 scans, 128 f1 increments and 757 f2 data points, f1 spectral width of 25 ppm (1774 Hz), f2 spectral width of 12 ppm (8418 Hz). Spectra were processed and analysed in MestreNova v14.0. Spectra were zero filled to 4096 data points and manually phase corrected using the largest peak as the pivot point.

The ^{1}H-^{15}N SOFAST HMQC spectra were analysed using Mbinding from MestreNova. The Euclidean distances are calculated using Equation 12 and the value of α used was 0.14. The experimental CSP's were used to calculate binding curves. The CSP's measured at different protein concentrations can be used in a nonlinear least-square fitting to calculate K_d using Equation 13. The final K_d was calculated as the average of the individual values of K_d obtained for 20 different peaks.

$$\Delta\delta_{obs} = \Delta\delta_{max} \frac{\left\{([P]_t + [L]_t + K_d) - [([P]_t + [L]_t + K_d)^2 - 4[P]_t[L]_t]^{\frac{1}{2}}\right\}}{2[P]_t}$$

Equation 13. Using CSP's to estimate K_{d}.

Where $\Delta\delta_{obs}$ is the change in the observed shift from the shift of the native protein, $\Delta\delta_{max}$ is the maximum chemical shift change on saturation of the protein with ligand, $[P]_t$ is the total protein concentration and $[L]_t$ is the total ligand concentration.

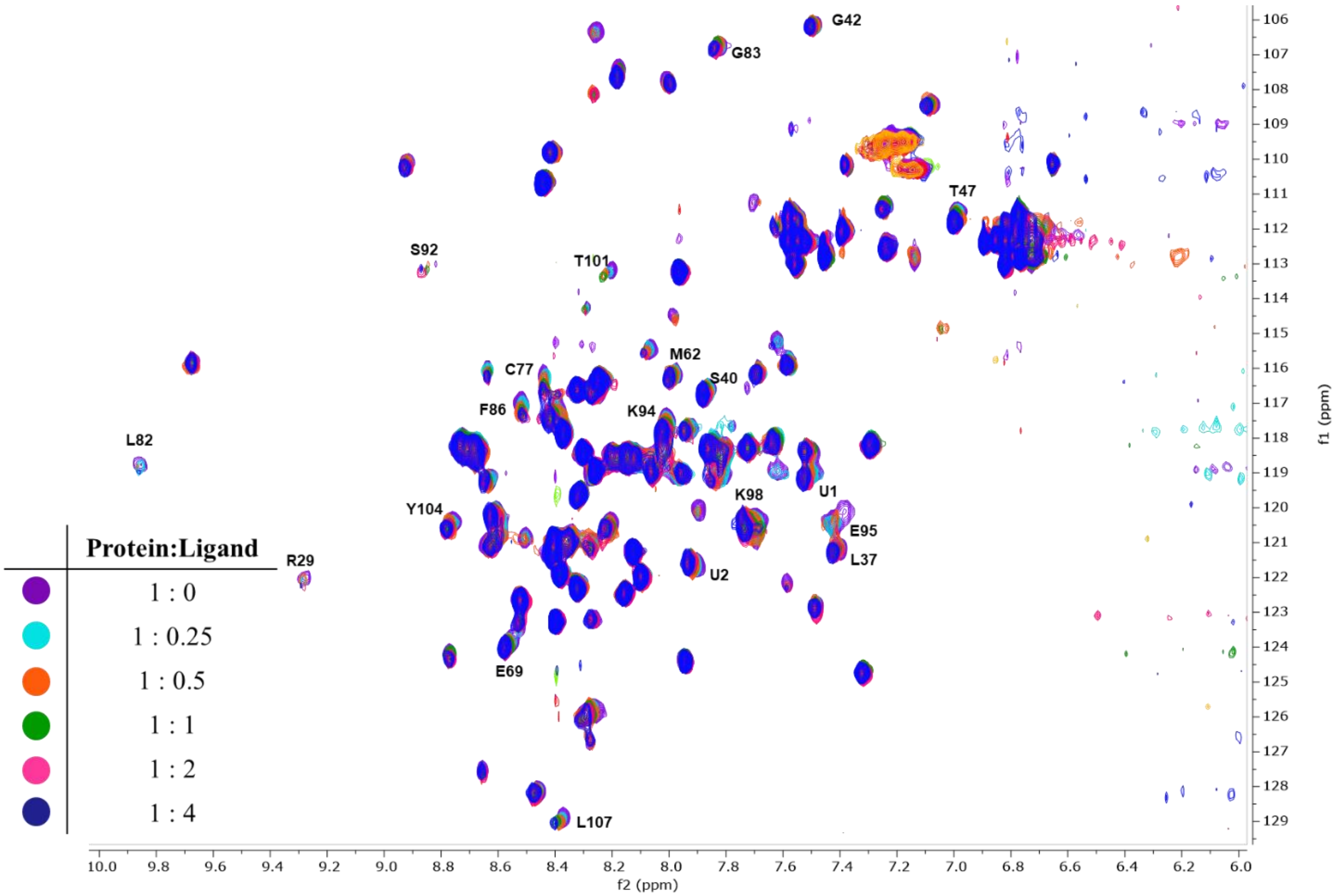

Figure 47. The titration of ligand **15** against Mdm2. Purple (70 µM Mdm2), light blue (70 µM Mdm2 + 17.5 µM ligand), orange (70 µM Mdm2 + 35 µM ligand), green (70 µM Mdm2 + 70 µM ligand), pink (70 µM Mdm2 + 140 µM ligand), dark blue (70 µM Mdm2 + 280 µM ligand). The 20 peaks that were considered in the calculation of K_d are highlighted.

Table 24. The calculated value of K_d and maximum CSP for 20 different residues interacting with **15**. The average K_d and σ K_d are also shown.

Residue	K_d (μM)	CSP Max (ppm)
C77	16.91	0.09
E95	15.98	0.03
F86	13.21	0.06
G42	20.28	0.02
G83	24.96	0.03
K94	12.27	0.06
K98	12.90	0.05
L107	15.17	0.04
L37	23.39	0.07
L82	21.47	0.04
M62	20.88	0.03
R29	17.07	0.05
S40	18.17	0.03
S92	11.62	0.06
T101	10.49	0.07
T47	12.08	0.05
Y104	15.34	0.04
E69	20.96	0.03
unassigned1 (U1)	24.23	0.05
unassigned2 (U2)	16.24	0.03
	**Average K_d (μM)**	**17.18**
	**σ K_d (μM)**	**4.45**

Example Binding Data for T101

Table 25. A table showing the chemical shifts of the T101 residue at increasing concentrations of **15**. The measured and calculated CSP’s are also shown.

Lt/Pt	F1 (ppm)	F2 (ppm)	CSP (ppm)	Calculated CSP (ppm)
0	113.18	8.20	0.000	0.000
0.25	113.24	8.21	0.015	0.015
0.5	113.30	8.22	0.031	0.027
1	113.37	8.23	0.045	0.046
2	113.40	8.25	0.058	0.060
4	113.49	8.24	0.065	0.065

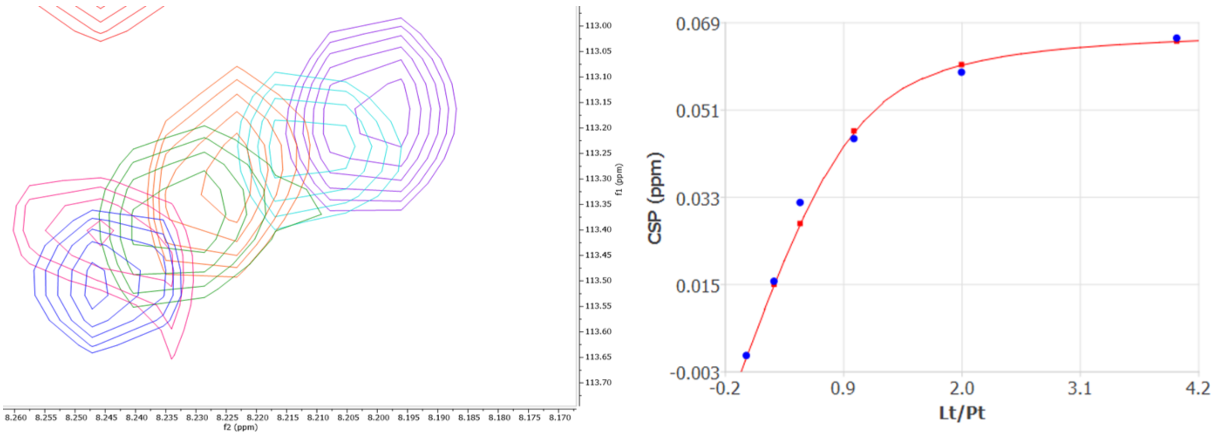

Figure 48. The peaks of the T101 residue at increasing concentrations of **15** (left) and the binding curve plotted using Mbinding. The blue are the experimental CSP’s and the red are the calculated CSP’s.

7.4 The Estimation of K_d using ^1H-^{15}N SOFAST HMQC for 16

A series of 2D ^1H-^{15}N SOFAST HMQC spectra of Mdm2 (70 µM) with five different concentrations of ligand **16**, ranging from 17.5 to 280 µM, were acquired using the same conditions and procedures as described for ligand **16** previously. The ^1H-^{15}N SOFAST HMQC spectra were analysed using Mbinding from MestreNova and an average value of K_d was obtained.

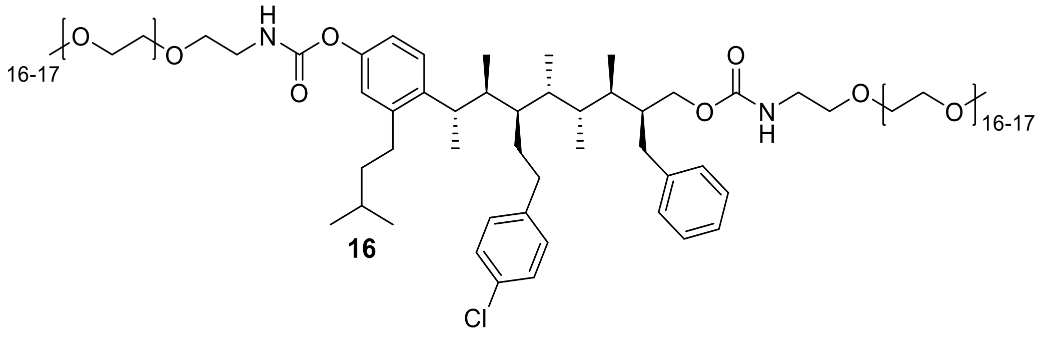

16

Table 26. The calculated value of K_d and maximum CSP for 20 different residues interacting with **16**. The average K_d and σ K_d are also shown.

Residue	K_d (µM)	CSP Max (ppm)
C77	11.84	0.09
E69	3.29	0.05
F86	5.43	0.13
F91	7.34	0.06
G83	6.86	0.05
K64	9.70	0.06
K94	6.85	0.11
L107	8.04	0.08
L37	12.41	0.05
L82	11.07	0.04
M62	14.47	0.08
S40	10.62	0.07
S92	3.72	0.13
T47	7.38	0.10
T49	9.96	0.06
U1	7.17	0.07
U2	11.70	0.07
Y104	8.55	0.07
Y48	6.71	0.04
Y60	13.11	0.05
**Average K_d (µM)**		**8.81**
**σ K_d (µM)**		**3.07**

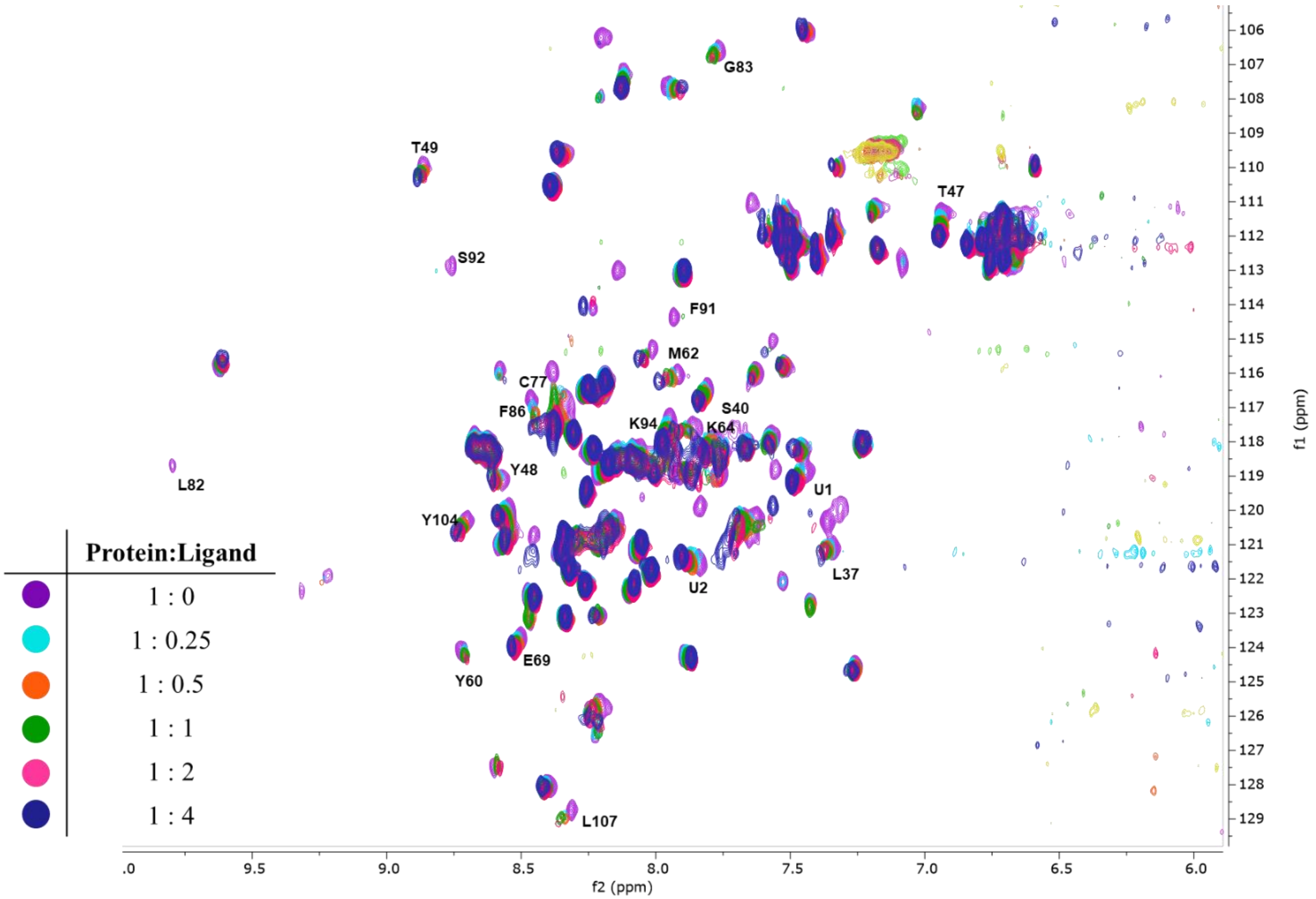

Figure 49. The titration of ligand **16** against Mdm2. Purple (70 μM Mdm2), light blue (70 μM Mdm2 + 17.5 μM ligand), orange (70 μM Mdm2 + 35 μM ligand), green (70 μM Mdm2 + 70 μM ligand), pink (70 μM Mdm2 + 140 μM ligand), dark blue (70 μM Mdm2 + 280 μM ligand). The 20 peaks that were considered in the calculation of K_d are highlighted.

Example Binding Data for M62

Table 27. A table showing the chemical shifts of the M62 residue at increasing concentrations of **16**. The measured and calculated CSP is also shown.

Lt/Pt	F1 (ppm)	F2 (ppm)	CSP (ppm)	Calculated CSP (ppm)
0	116.03	7.92	0.000	0.000
0.25	116.06	7.94	0.023	0.015
0.5	116.10	7.95	0.031	0.027
1	116.16	7.95	0.043	0.046
2	116.22	7.97	0.061	0.063
4	116.22	7.98	0.072	0.071

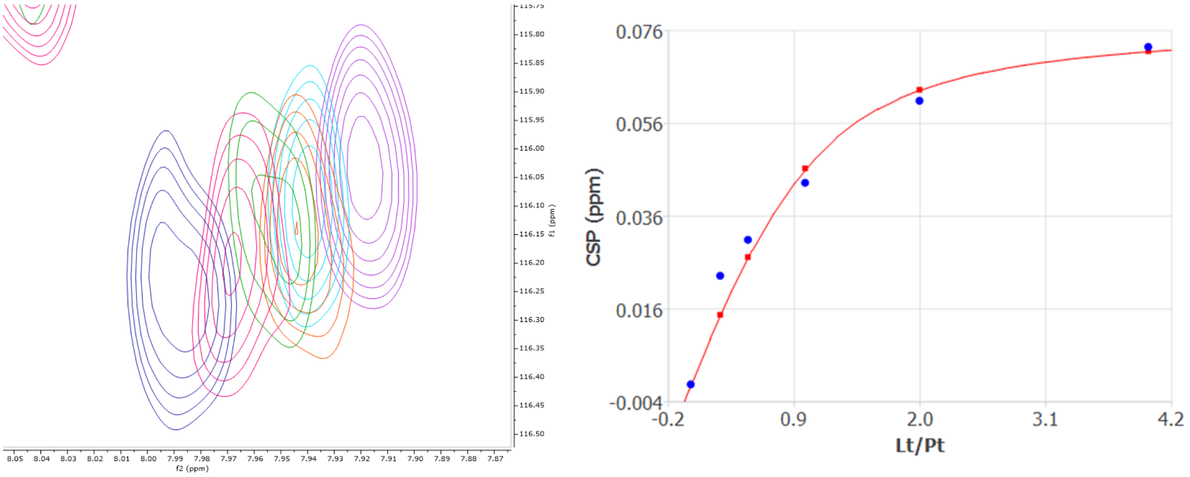

Figure 50. The peaks of the M62 residue at increasing concentrations of **16** (left) and the binding curve plotted using Mbinding. The blue are the experimental CSP’s and the red are the calculated CSP’s.

7.5 The Estimation of K_d using ^1H-^{15}N SOFAST HMQC for 17

A series of 2D ^1H-^{15}N SOFAST HMQC spectra of Mdm2 (70 µM) with five different concentrations of ligand **17**, ranging from 35 to 350 µM, were acquired using the same conditions and procedures as described for ligand **17** previously. The ^1H-^{15}N SOFAST HMQC spectra were analysed using Mbinding from MestreNova and an average value of K_d was obtained.

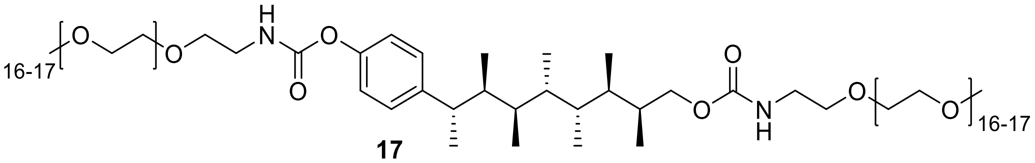

17

Table 28. The calculated value of K_d and maximum CSP for 5 different residues interacting with **17**. The average K_d and σ K_d are also shown.

**Residue**	**K_d (µM)**	**CSP Max (ppm)**
E95	206.98	0.06
M62	199.03	0.04
S40	255.83	0.04
T101	180.29	0.08
T47	217.37	0.05
	**Average K_d (µM)**	**211.90**
	**σ K_d (µM)**	**28.06**

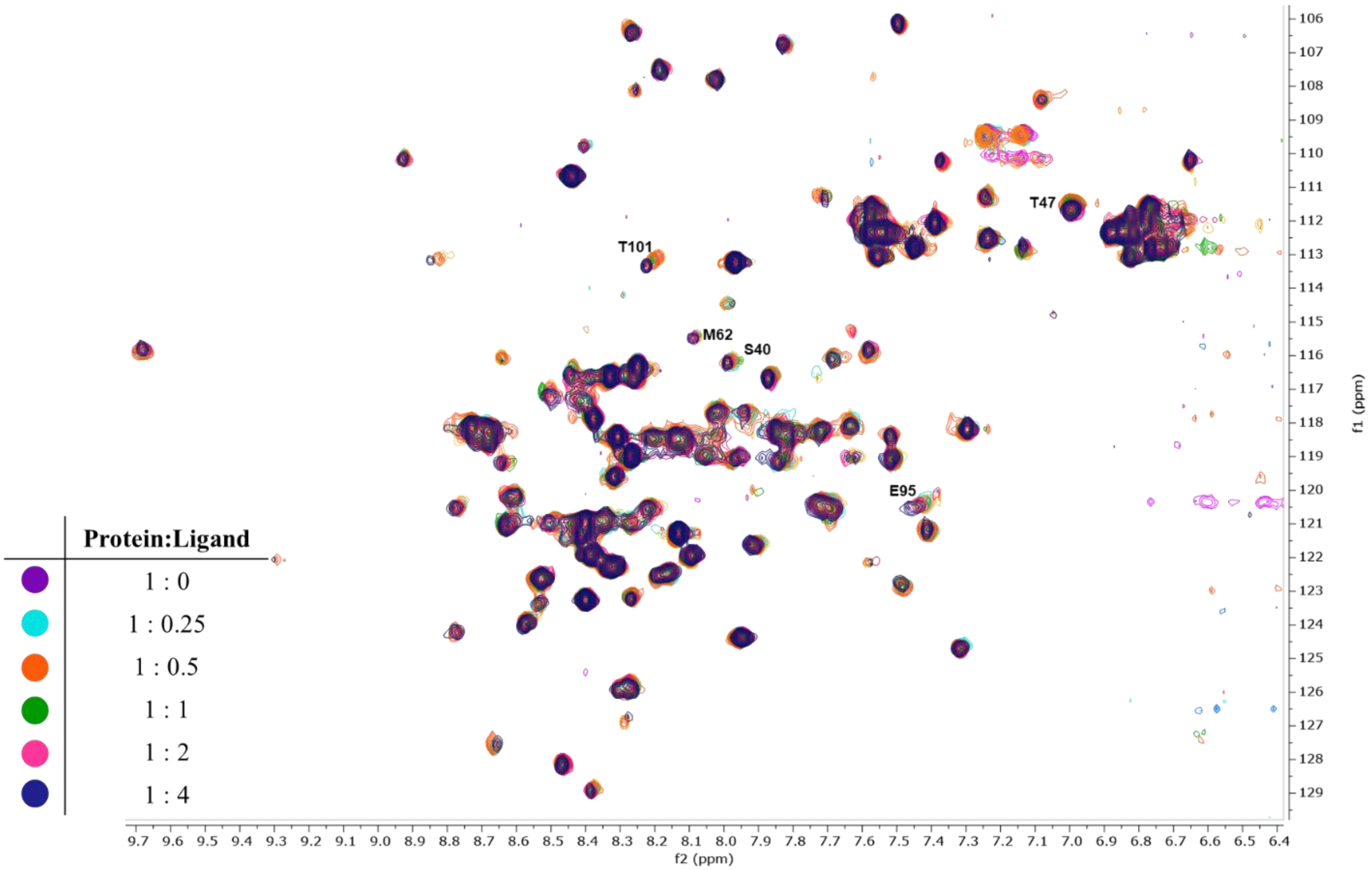

Figure 51. The titration of ligand **17** against Mdm2. Purple (70 µM Mdm2), light blue (70 µM Mdm2 + 35 µM ligand), orange (70 µM Mdm2 + 70 µM ligand), green (70 µM Mdm2 + 140 µM ligand), pink (70 µM Mdm2 + 280 µM ligand), dark blue (70 µM Mdm2 + 350 µM). The 5 peaks that were considered in the calculation of K_d are highlighted.

Example Binding Data for T101

Table 29. A table showing the chemical shifts of the T101 residue at increasing concentrations of **17.** The measured and calculated CSP is also shown.

Lt/Pt	F1 (ppm)	F2 (ppm)	CSP (ppm)	Calculated CSP (ppm)
0	113.09	8.191	0.000	0.000
0.25	113.17	8.20	0.017	0.011
0.5	113.18	8.21	0.022	0.020
1	113.20	8.21	0.025	0.032
2	113.36	8.22	0.052	0.048
4	113.34	8.23	0.052	0.053

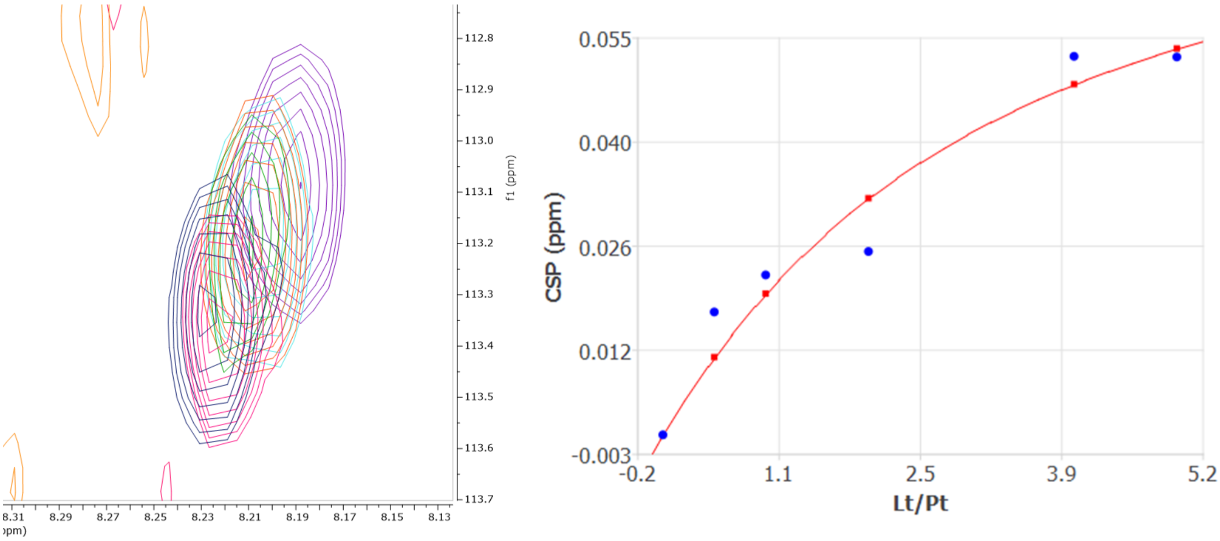

Figure 52. The peaks of the T101 residue at increasing concentrations of **17** (left) and the binding curve plotted using Mbinding. The blue are the experimental CSP’s and the red are the calculated CSP’s.

7.6 The Estimation of K_d using ^{1}H-^{15}N SOFAST HMQC for 18

A series of 2D ^{1}H-^{15}N SOFAST HMQC spectra of Mdm2 (70 µM) with six different concentrations of ligand **18**, ranging from 35 to 350 µM, were acquired using the same conditions and procedures as described for ligand **18** previously. The ^{1}H-^{15}N SOFAST HMQC spectra were analysed using Mbinding from MestreNova and an average value of K_d was obtained.

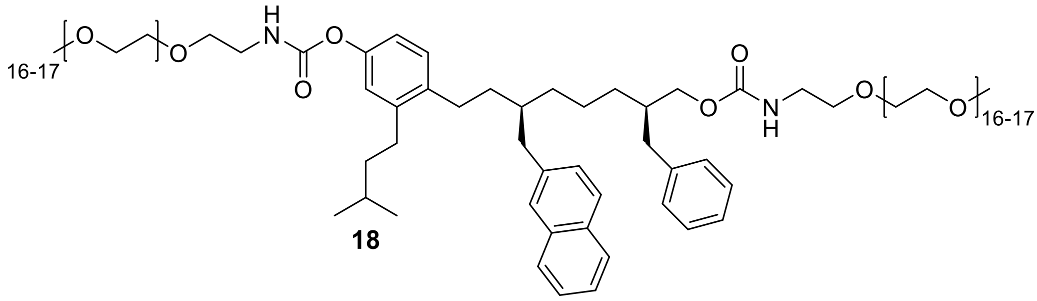

18

Table 30. The calculated value of K_d and maximum CSP for 5 different residues interacting with **18**. The average K_d and σ K_d are also shown.

Residue	K_d (µM)	CSP Max (ppm)
C77	149.37	0.17
E52	152.20	0.08
F86	169.84	0.09
Y104	157.06	0.04
T101	183.56	0.11
	**Average K_d (µM)**	**162.40**
	**σ K_d (µM)**	**14.19**

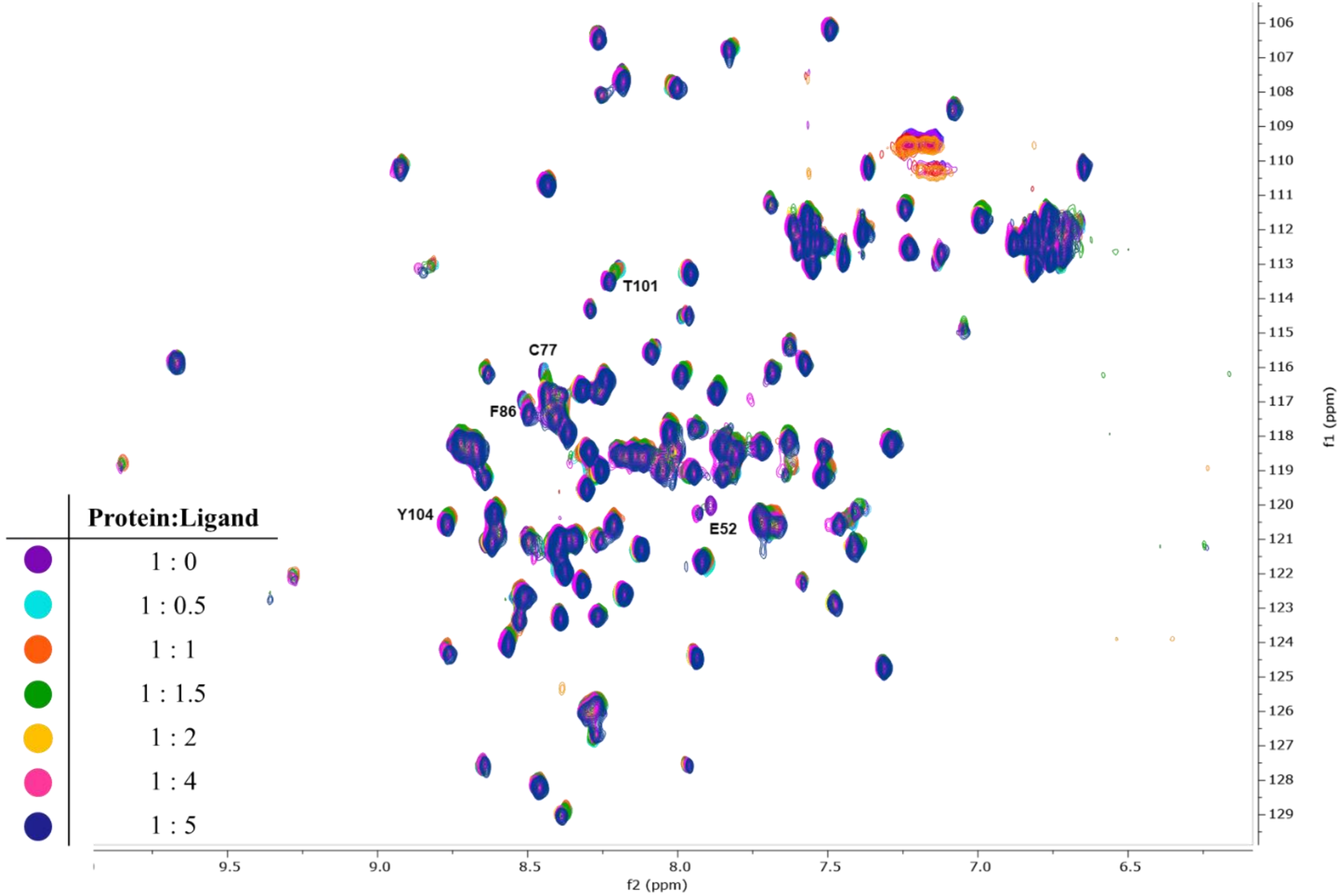

Figure 53. The titration of ligand **18** against Mdm2. Purple (70 µM Mdm2), light blue (70 µM Mdm2 + 35 µM ligand), orange (70 µM Mdm2 + 70 µM ligand), green (70 µM Mdm2 + 105 µM ligand), yellow (70 µM Mdm2 + 140 µM ligand) pink (70 µM Mdm2 + 280 µM ligand), dark blue (70 µM Mdm2 + 350 µM). The 5 peaks that were considered in the calculation of K_d are highlighted.

Example Binding Data for F86

Table 31. A table showing the chemical shifts of the F86 residue at increasing concentrations of **18**. The measured and calculated CSP is also shown.

Lt/Pt	F1 (ppm)	F2 (ppm)	CSP (ppm)	Calculated CSP (ppm)
0	116.97	8.52	0.000	0.000
0.5	117.01	8.51	0.011	0.012
1	117.05	8.50	0.019	0.022
1.5	117.15	8.50	0.032	0.030
2	117.21	8.51	0.038	0.036
4	117.29	8.50	0.049	0.053
5	117.35	8.50	0.060	0.058

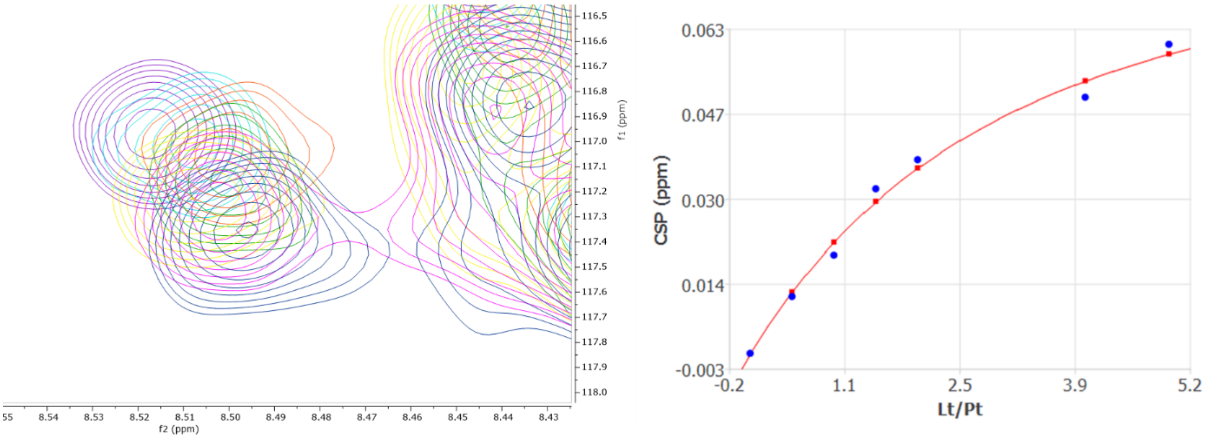

Figure 54. The peaks of the F86 residue at increasing concentrations of **18** (left) and the binding curve plotted using Mbinding. The blue are the experimental CSP's and the red are the calculated CSP's.

8.0 NMR Spectra

^{1}H-NMR (400 MHz, CDCl3) of compound **8**

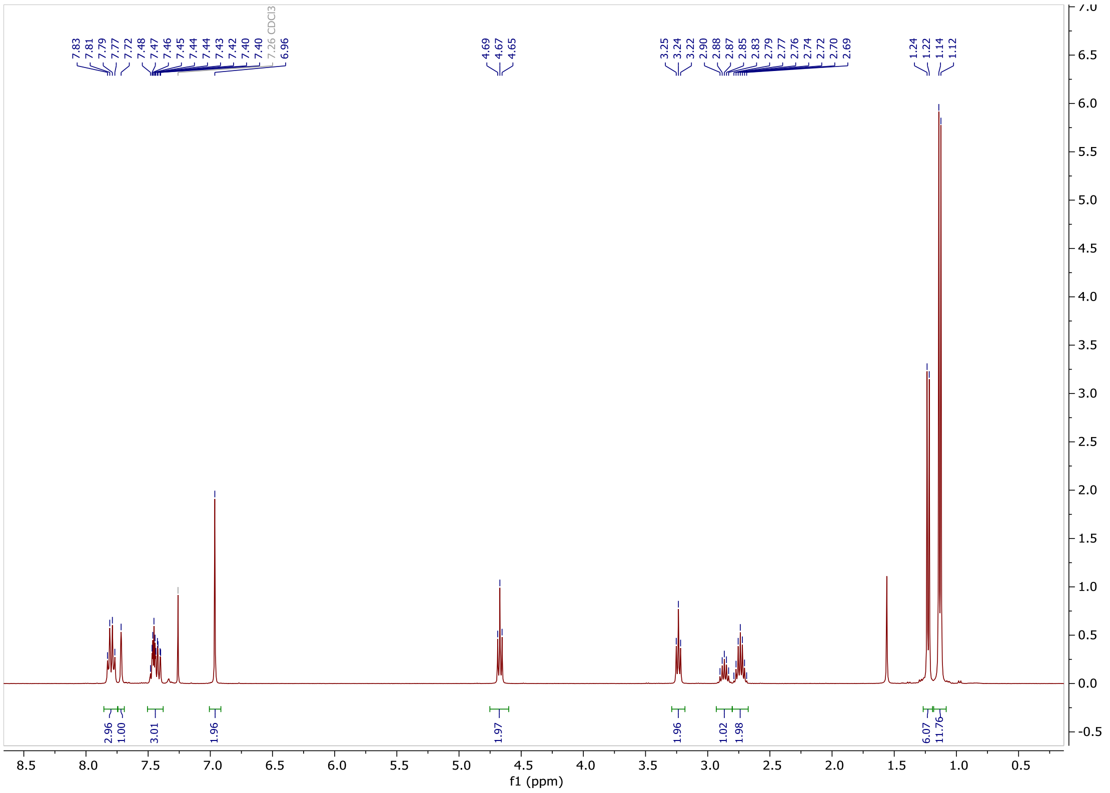

^{13}C-NMR (100 MHz, CDCl3) of compound **8**

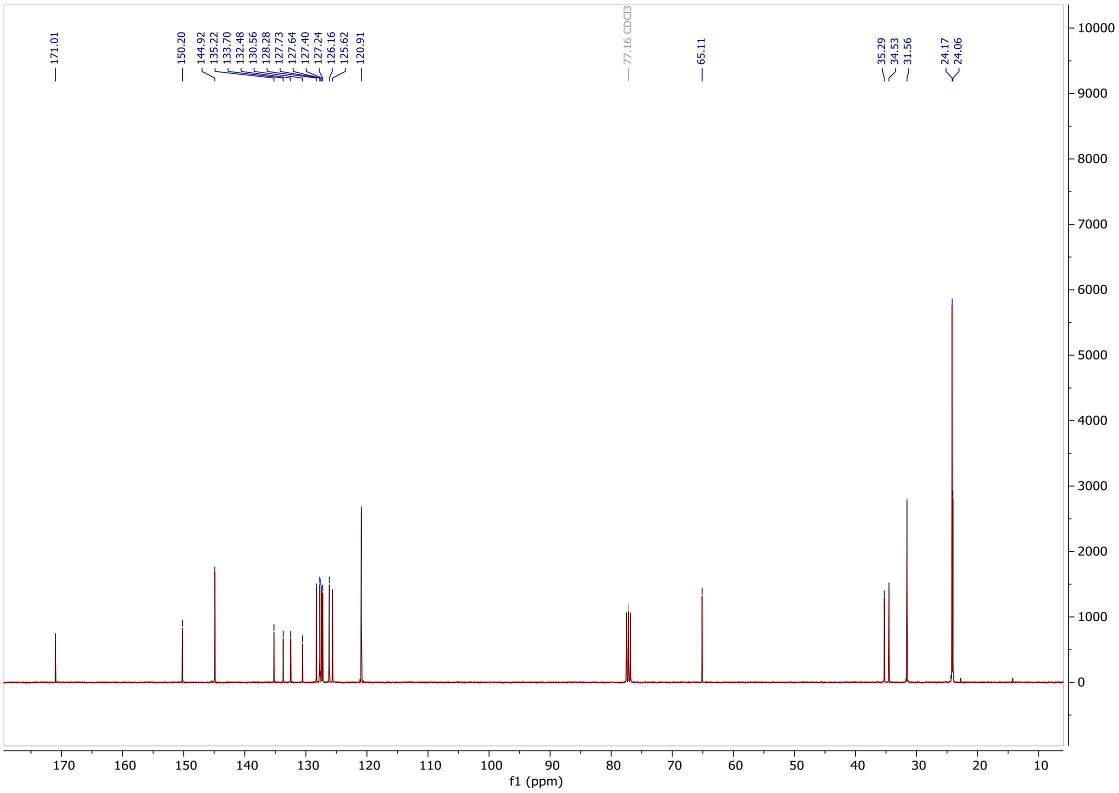

^{1}H-NMR (500 MHz, CDCl3) of compound **9**

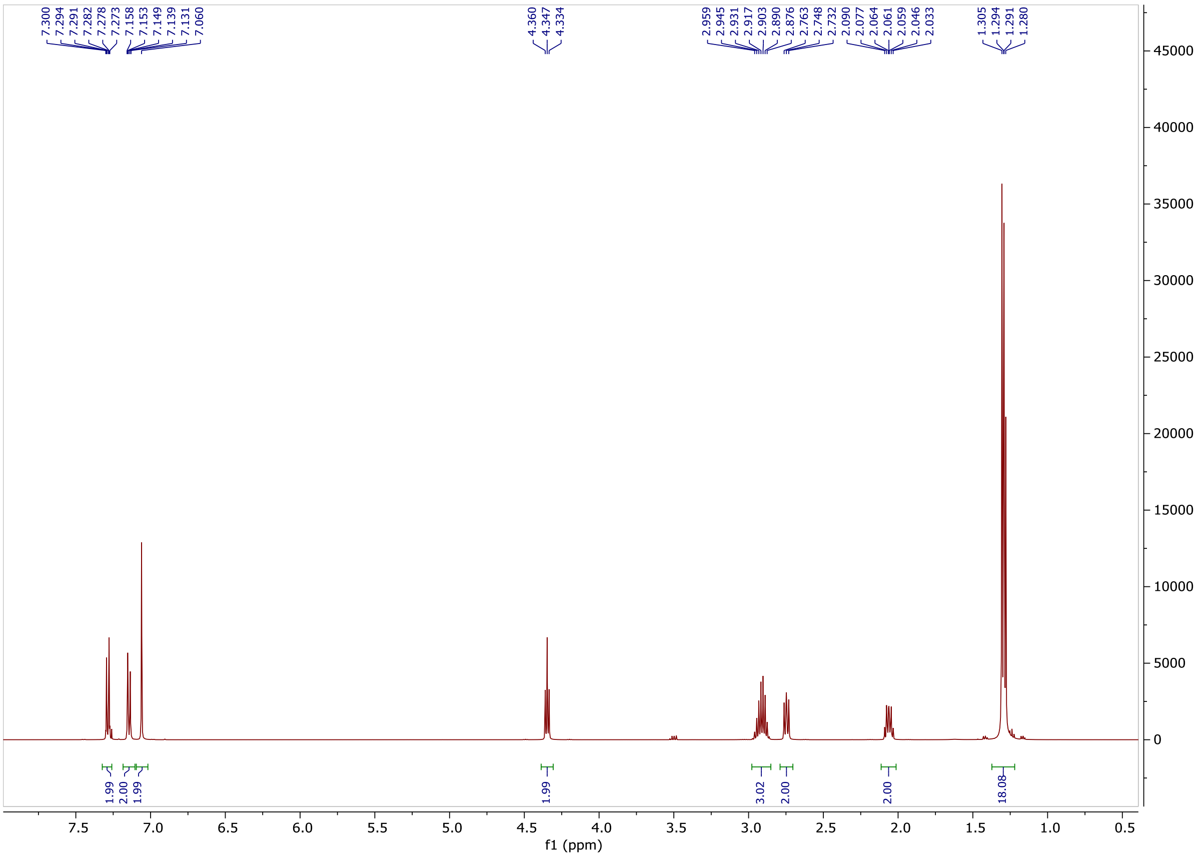

^{13}C-NMR (125 MHz, CDCl3) of compound **9**

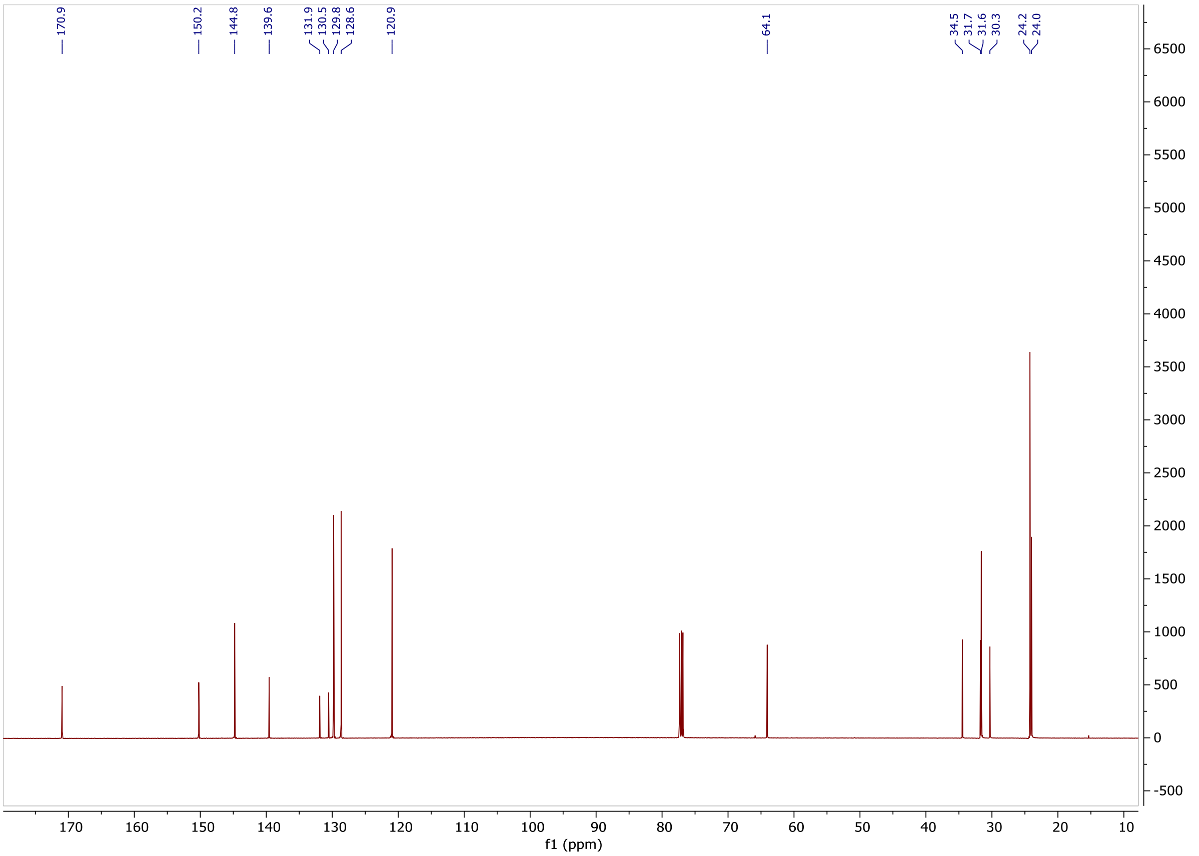

^{1}H-NMR (400 MHz, CDCl3) of compound **10**

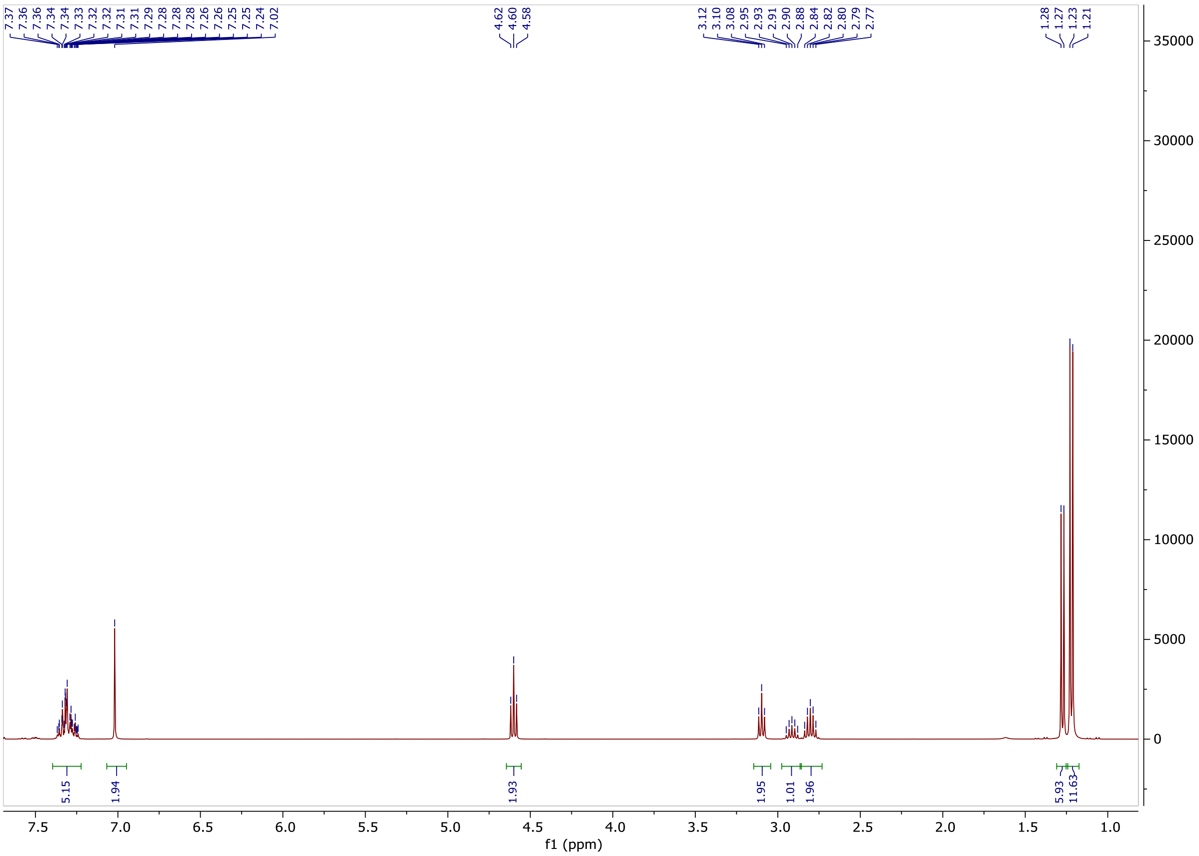

¹³C-NMR (100 MHz, CDCl3) of compound **10**

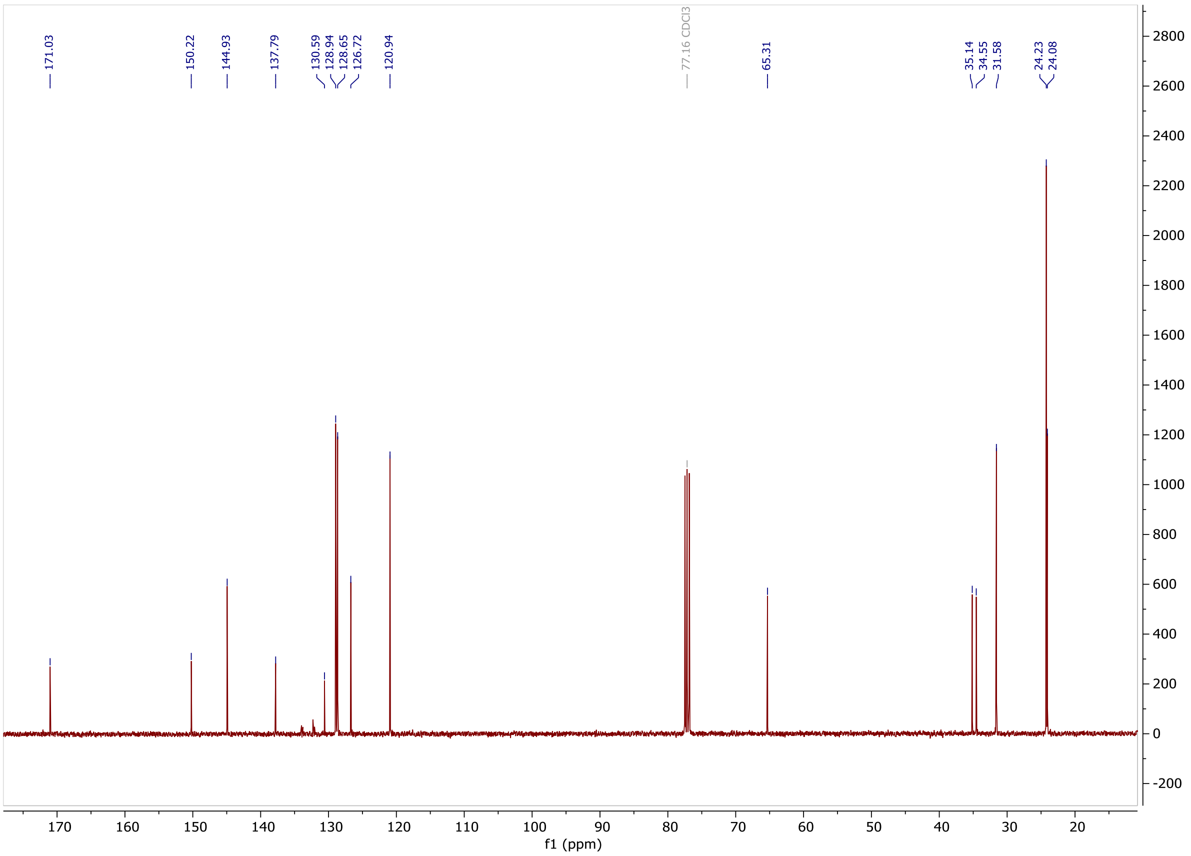

¹H-NMR (400 MHz, CDCl3) of compound **20**

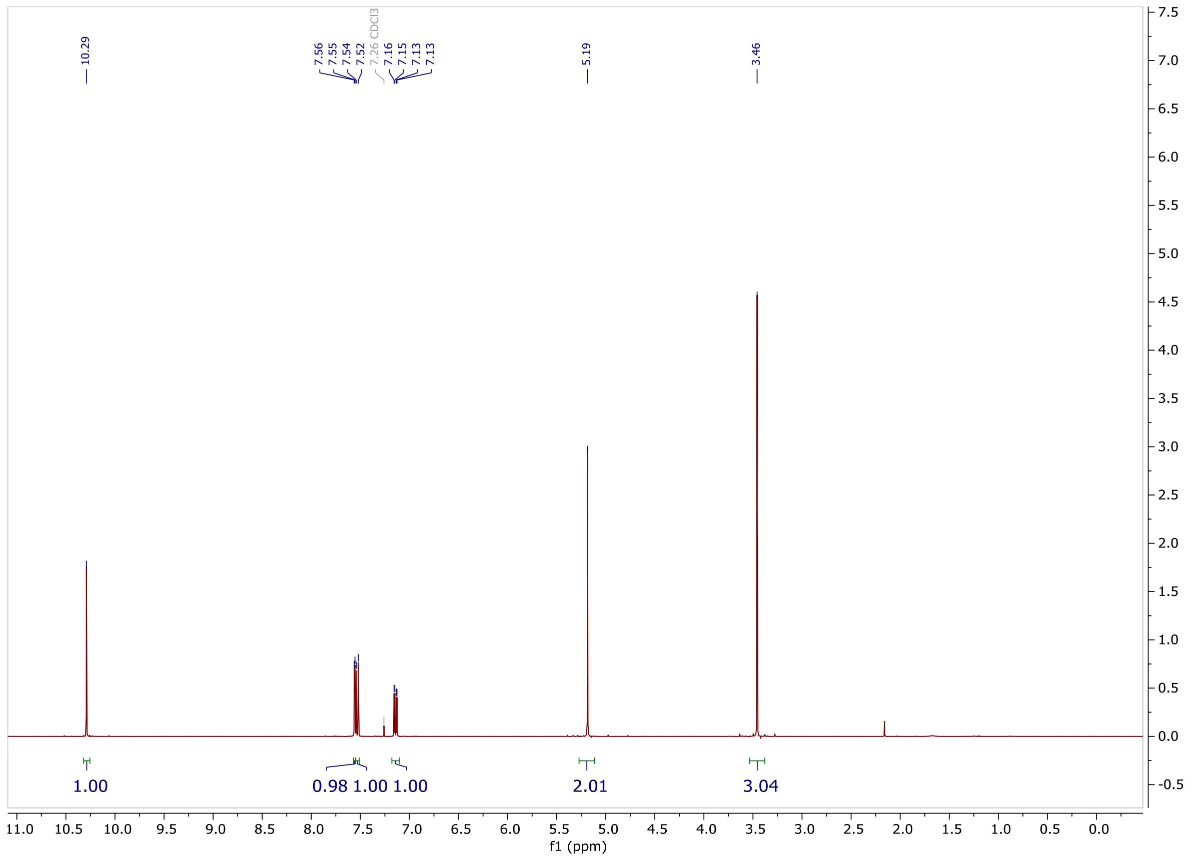

^{13}C-NMR (100 MHz, CDCl3) of compound **20**

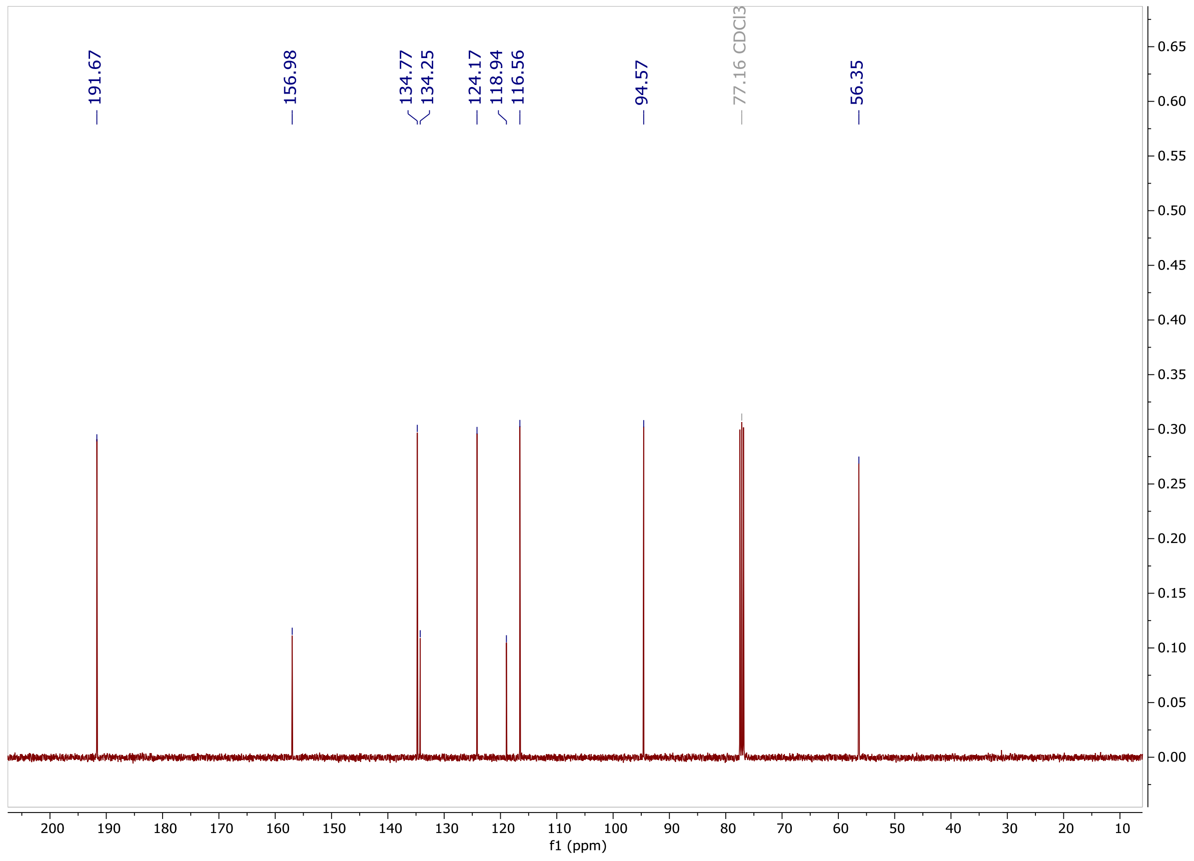

^{1}H-NMR (400 MHz, CDCl3) of compound **21**

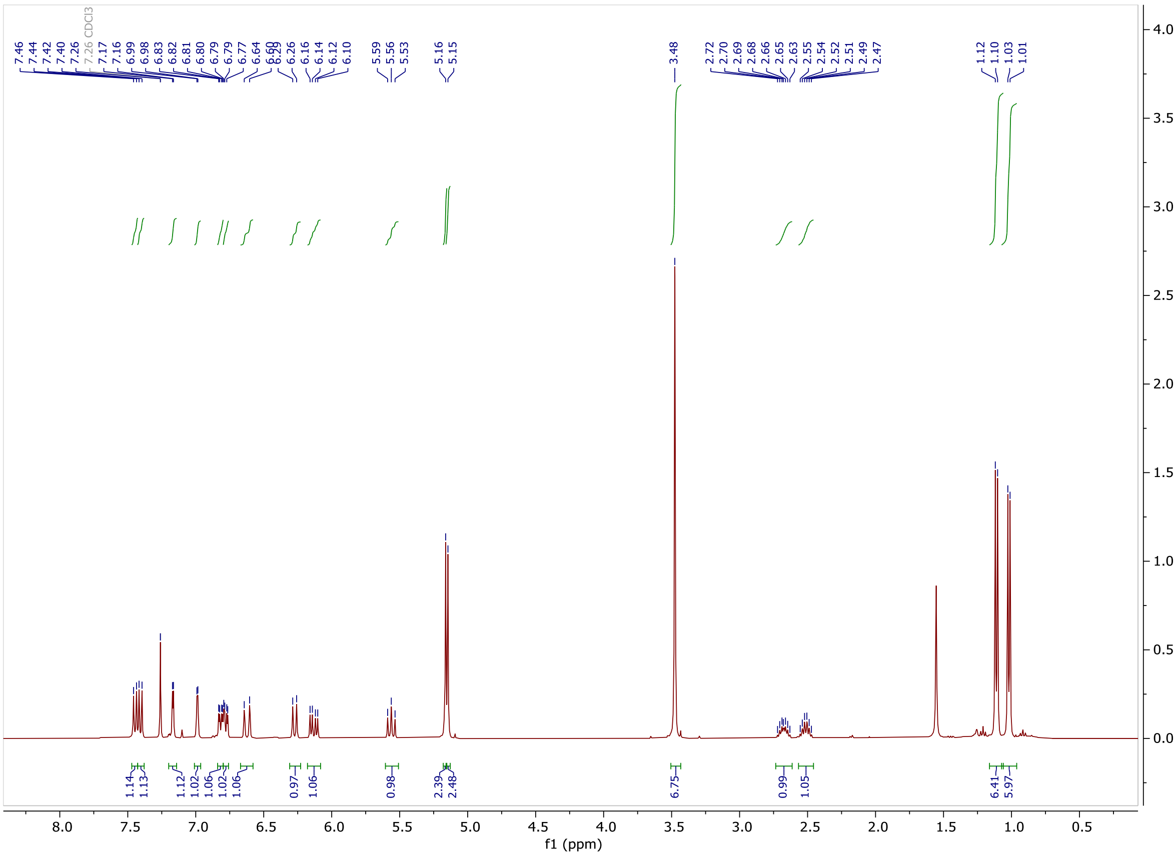

^{13}C-NMR (100 MHz, CDCl3) of compound **21**

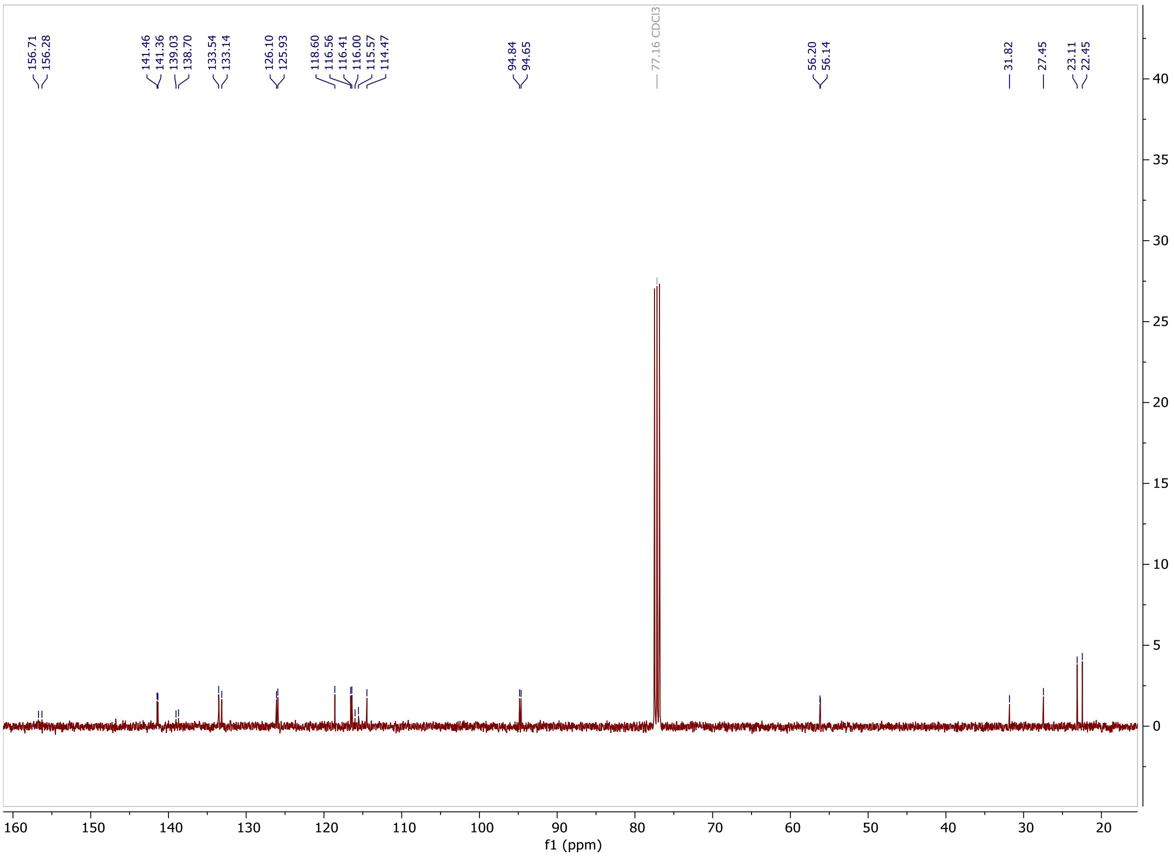

^{1}H-NMR (400 MHz, CDCl3) of compound **22**

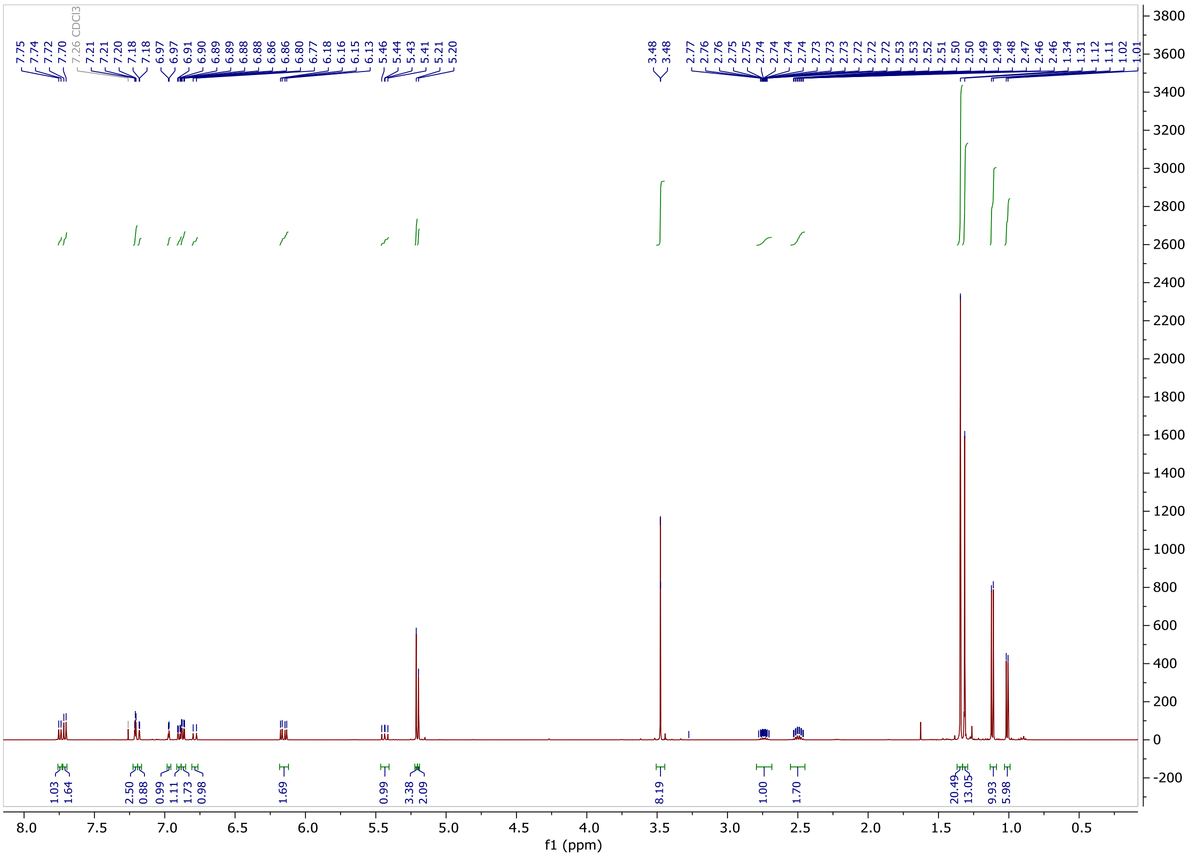

^{13}C-NMR (100 MHz, CDCl3) of compound **22**

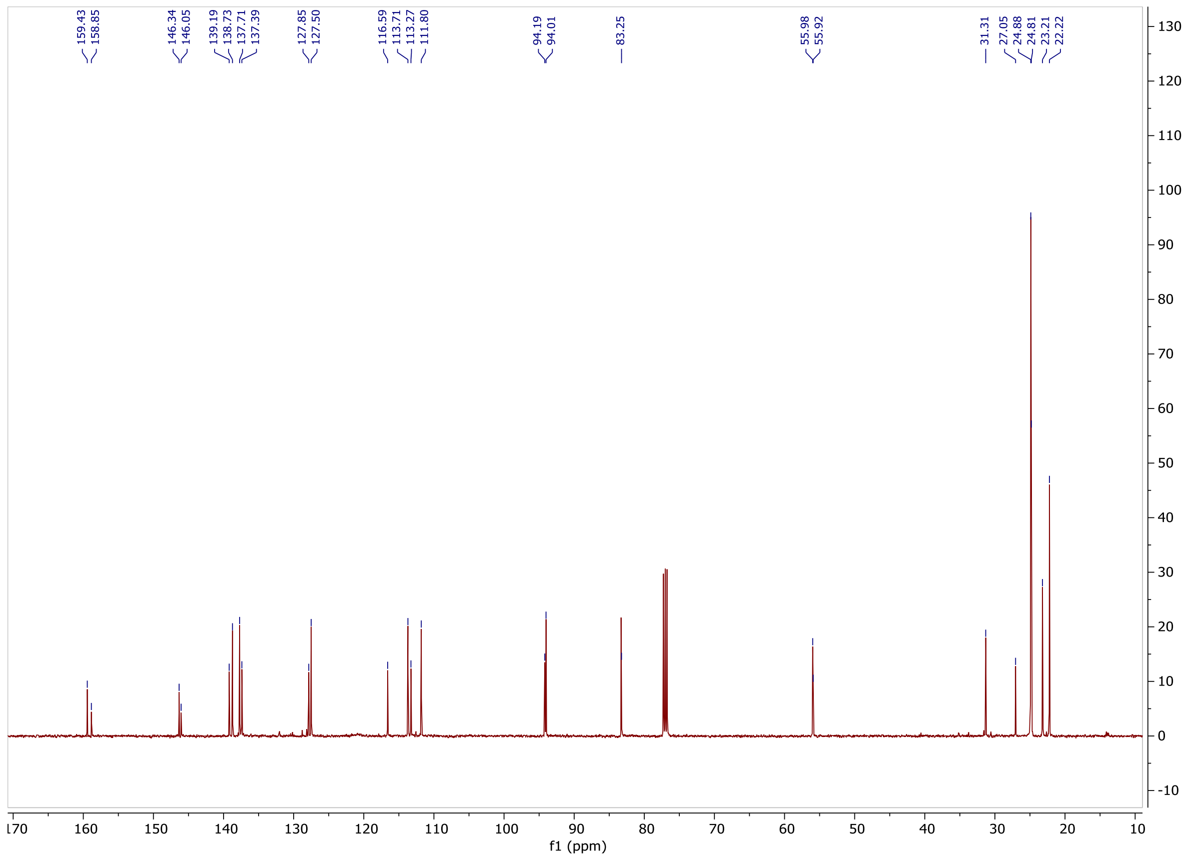

^{1}H-NMR (400 MHz, CDCl3) of compound **6**

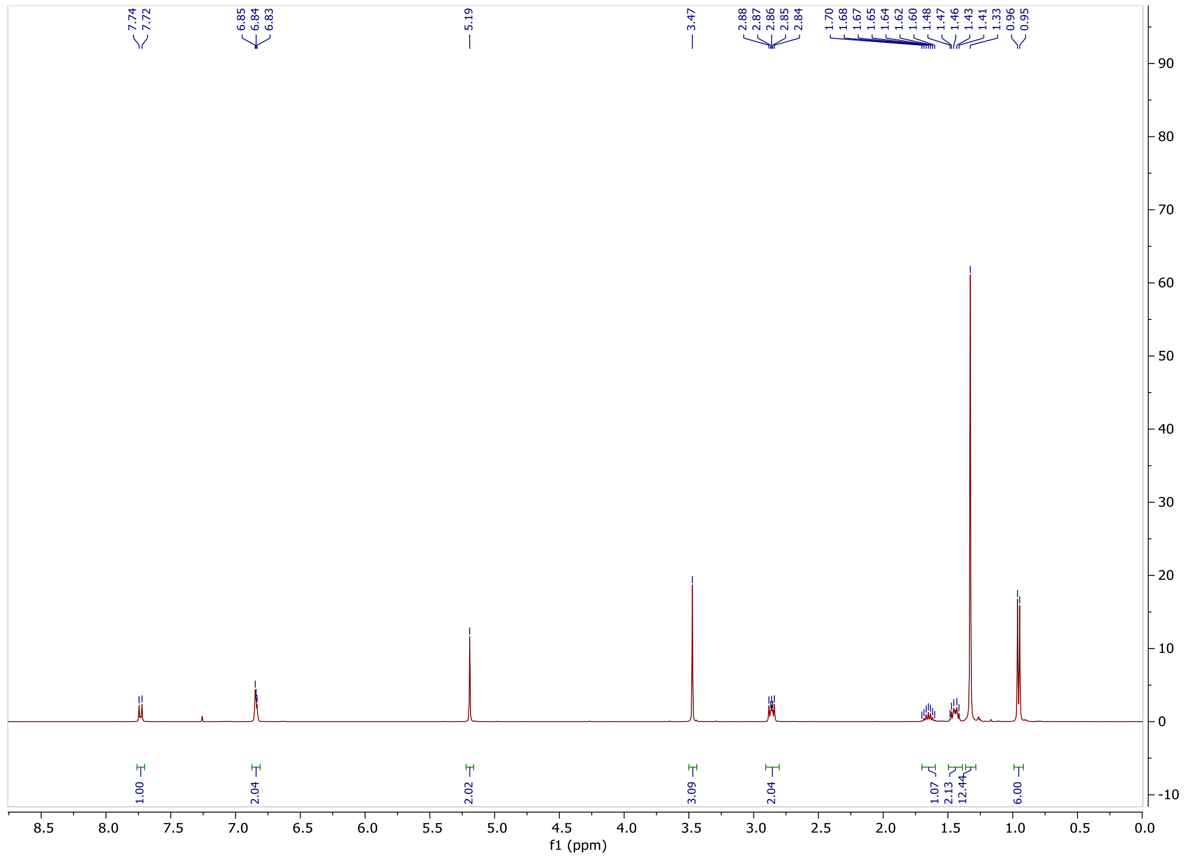

^{13}C-NMR (100 MHz, CDCl3) of compound **6**

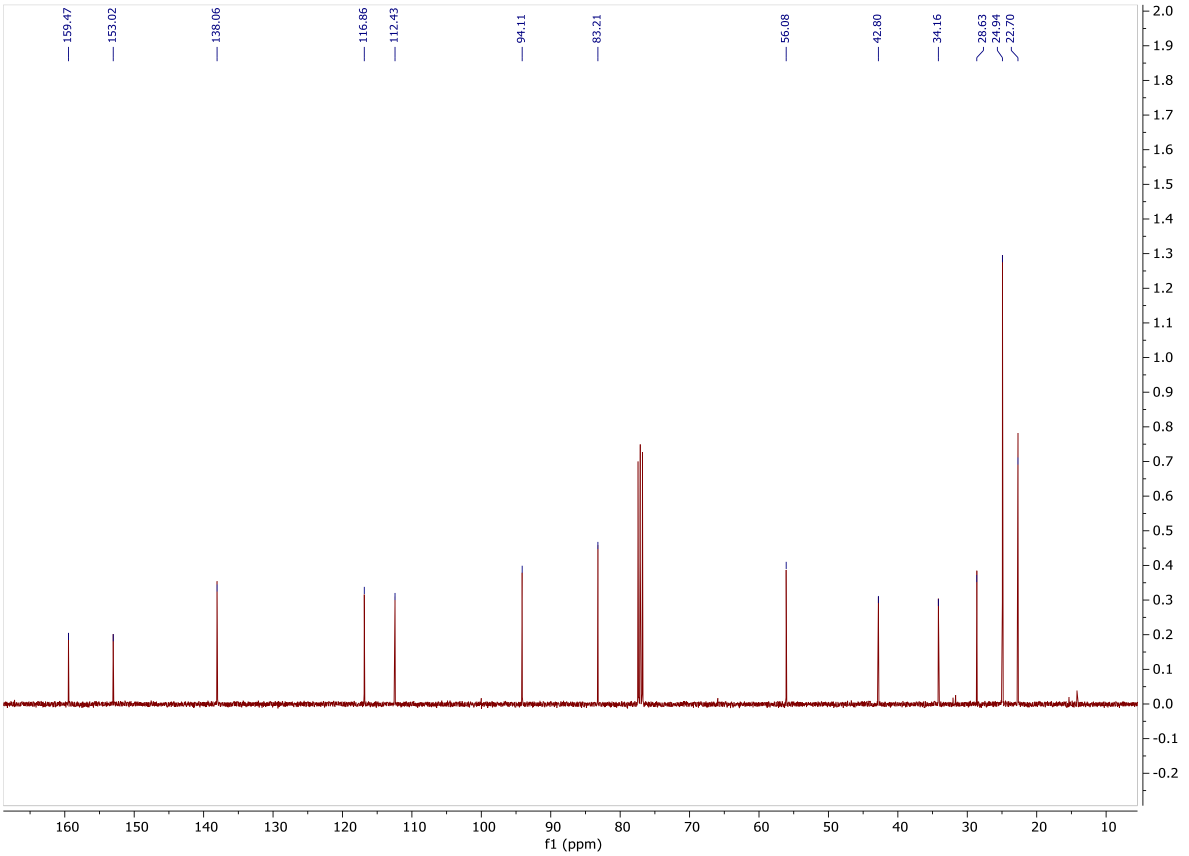

^{1}H-NMR (500 MHz, CDCl3) of compound **24**

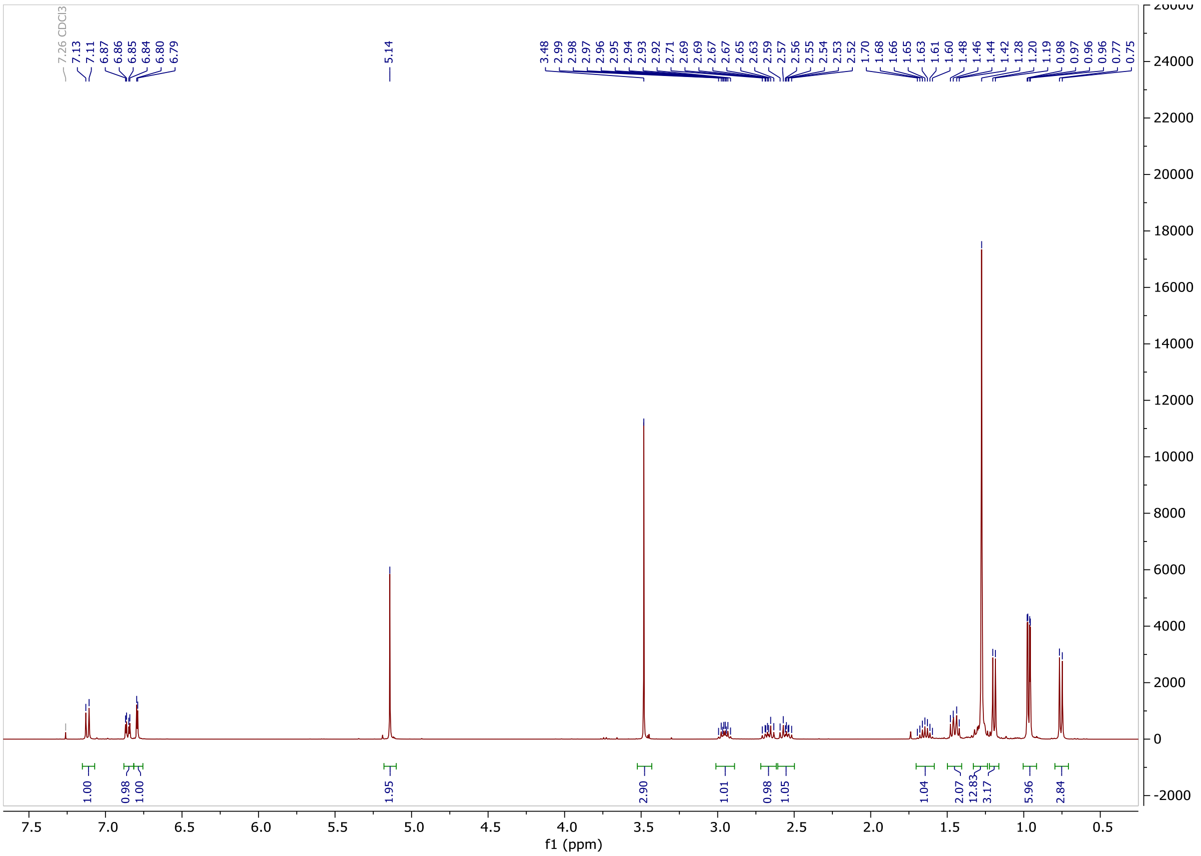

^{13}C-NMR (125 MHz, CDCl3) of compound **24**

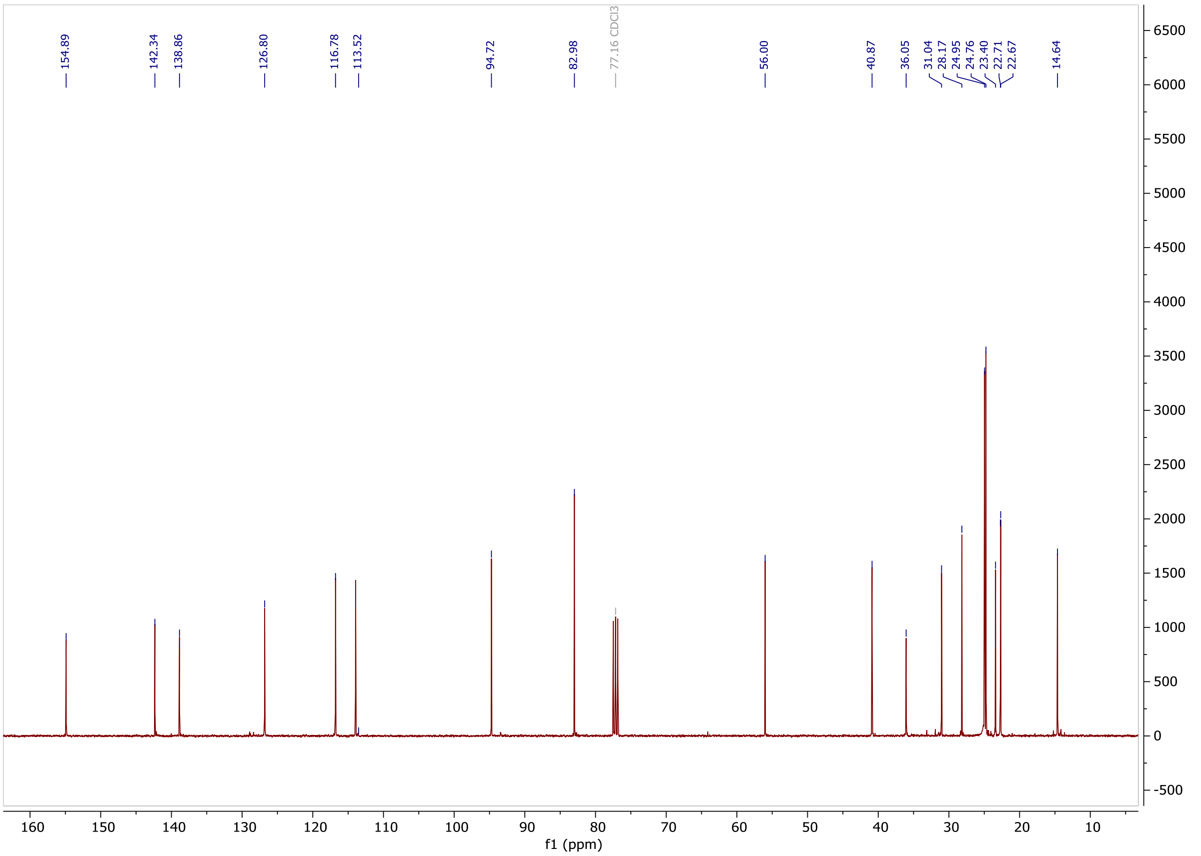

^{1}H-NMR (500 MHz, CDCl3) of compound **25**

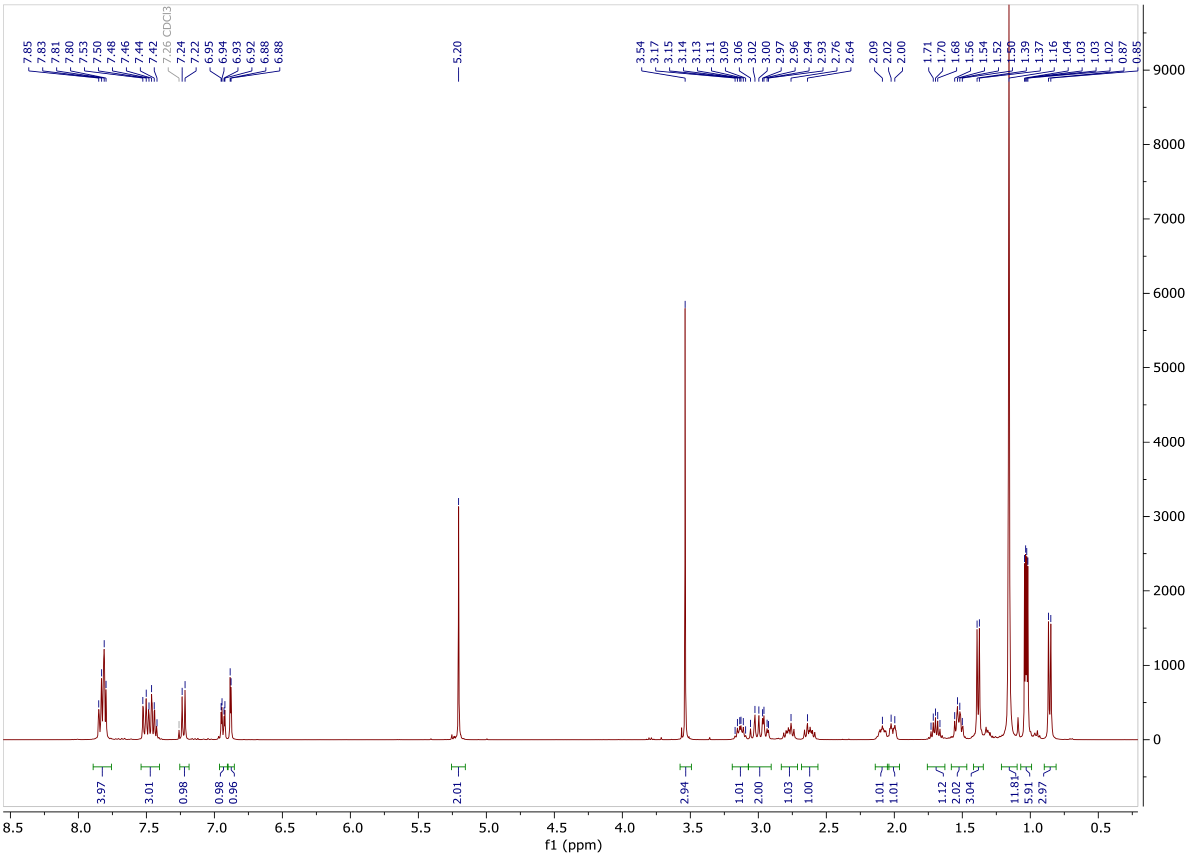

^{13}C-NMR (125 MHz, CDCl3) of compound **25**

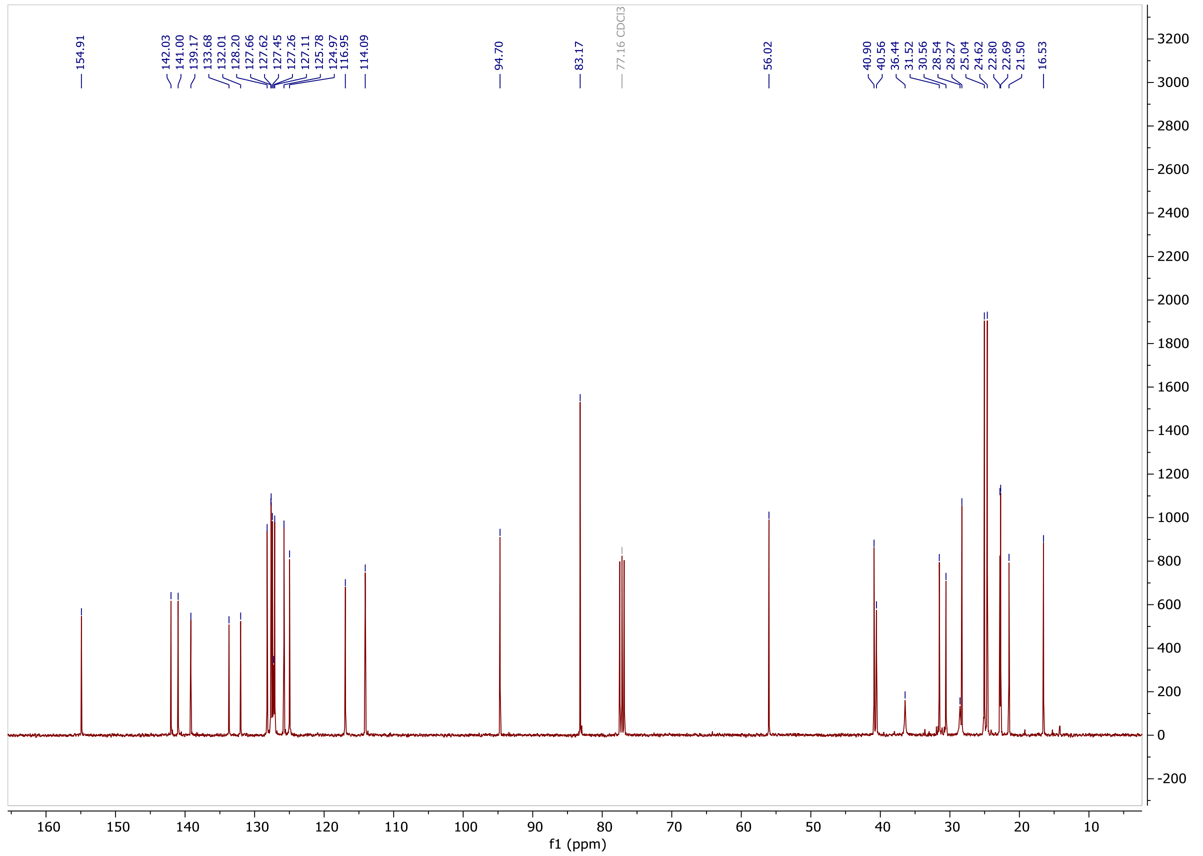

^{1}H-NMR (500 MHz, CDCl3) of compound **26**

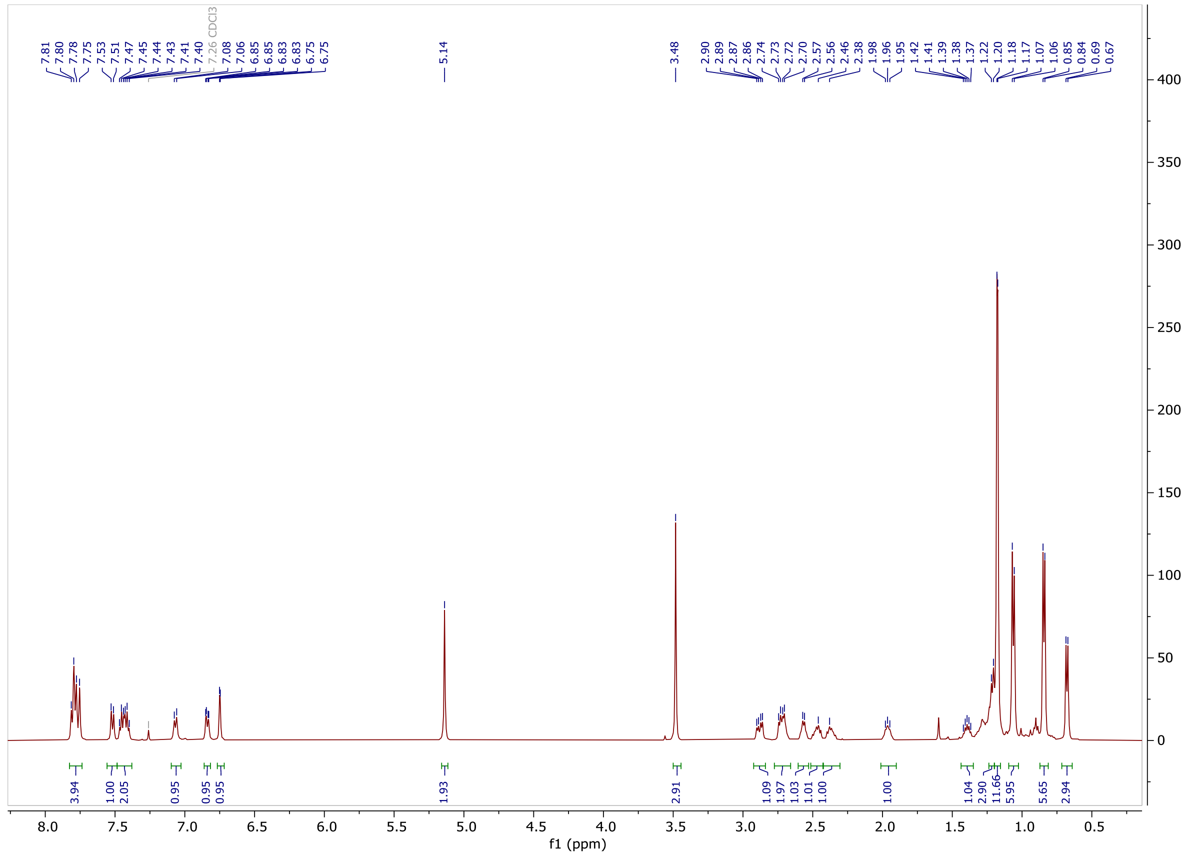

^{13}C-NMR (125 MHz, CDCl3) of compound **26**

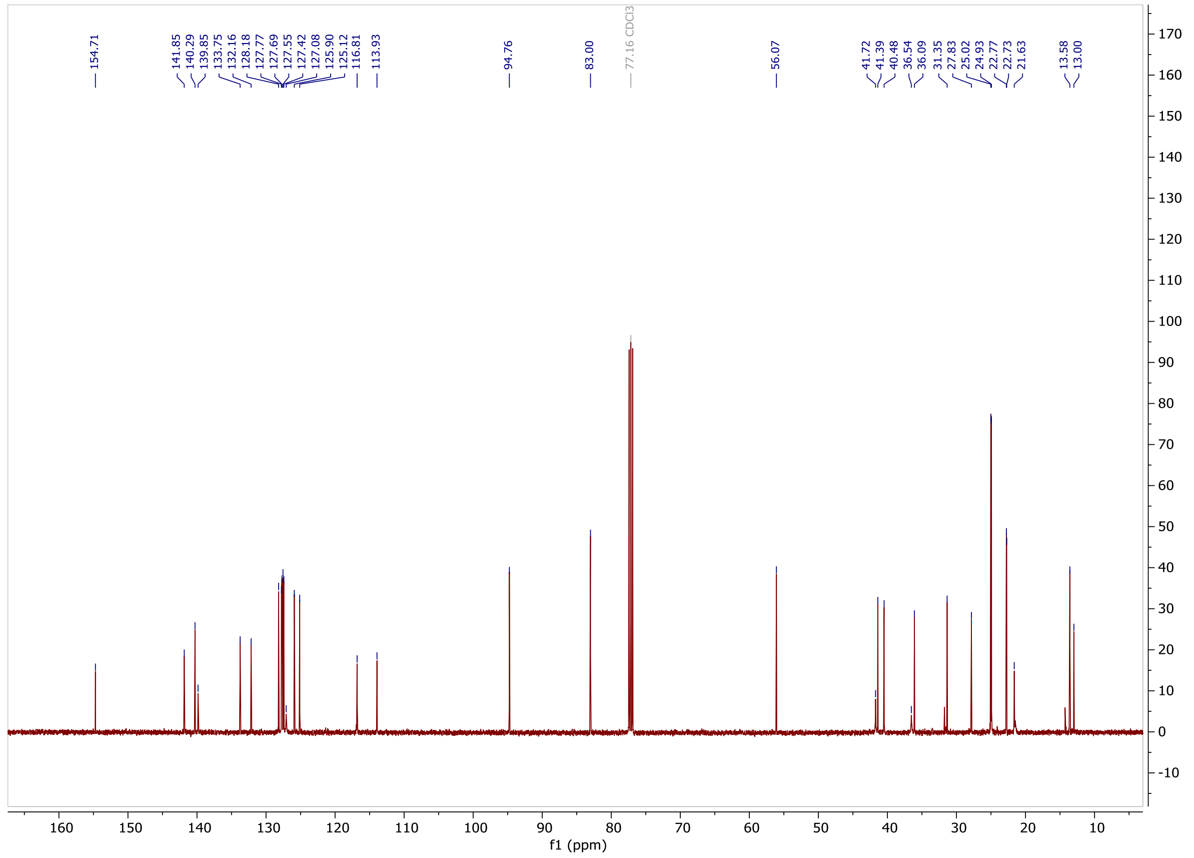

^{1}H-NMR (500 MHz, CDCl3) of compound **27**

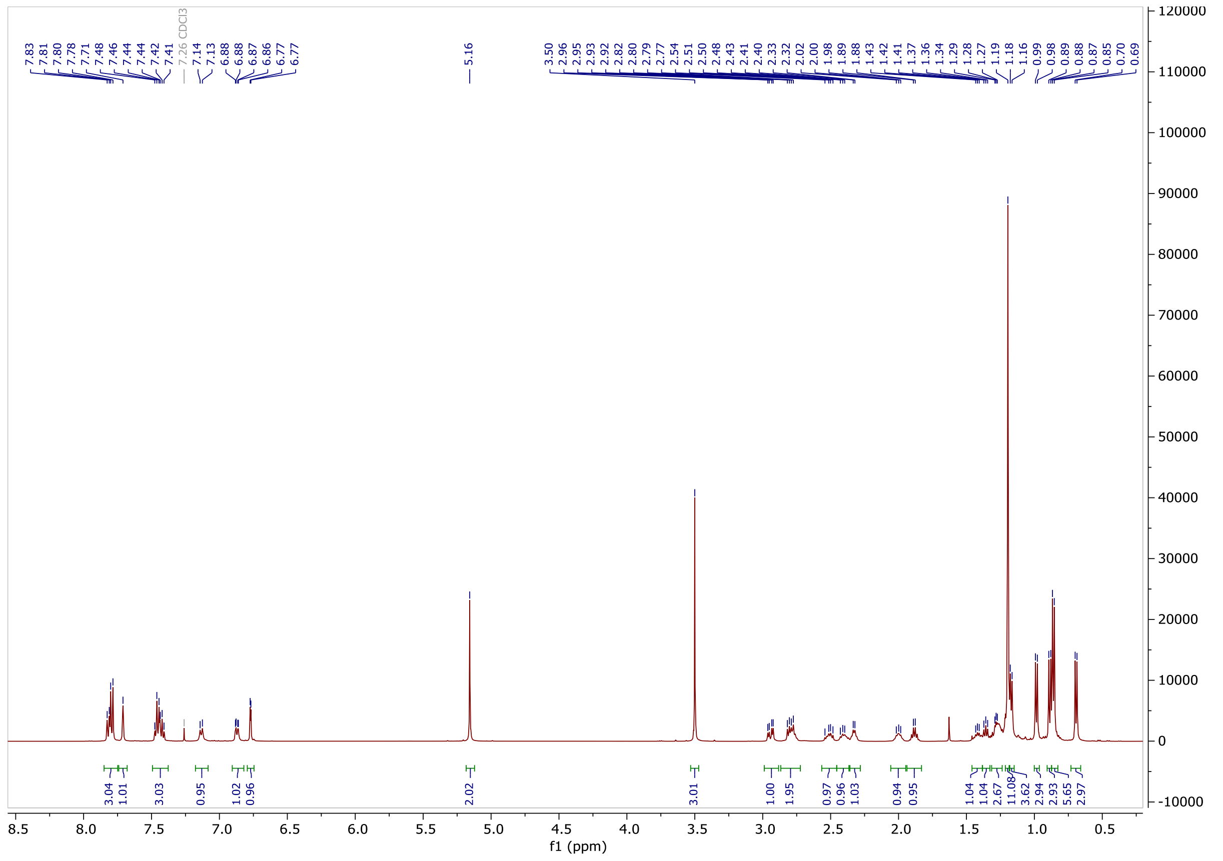

^{13}C-NMR (125 MHz, CDCl3) of compound **27**

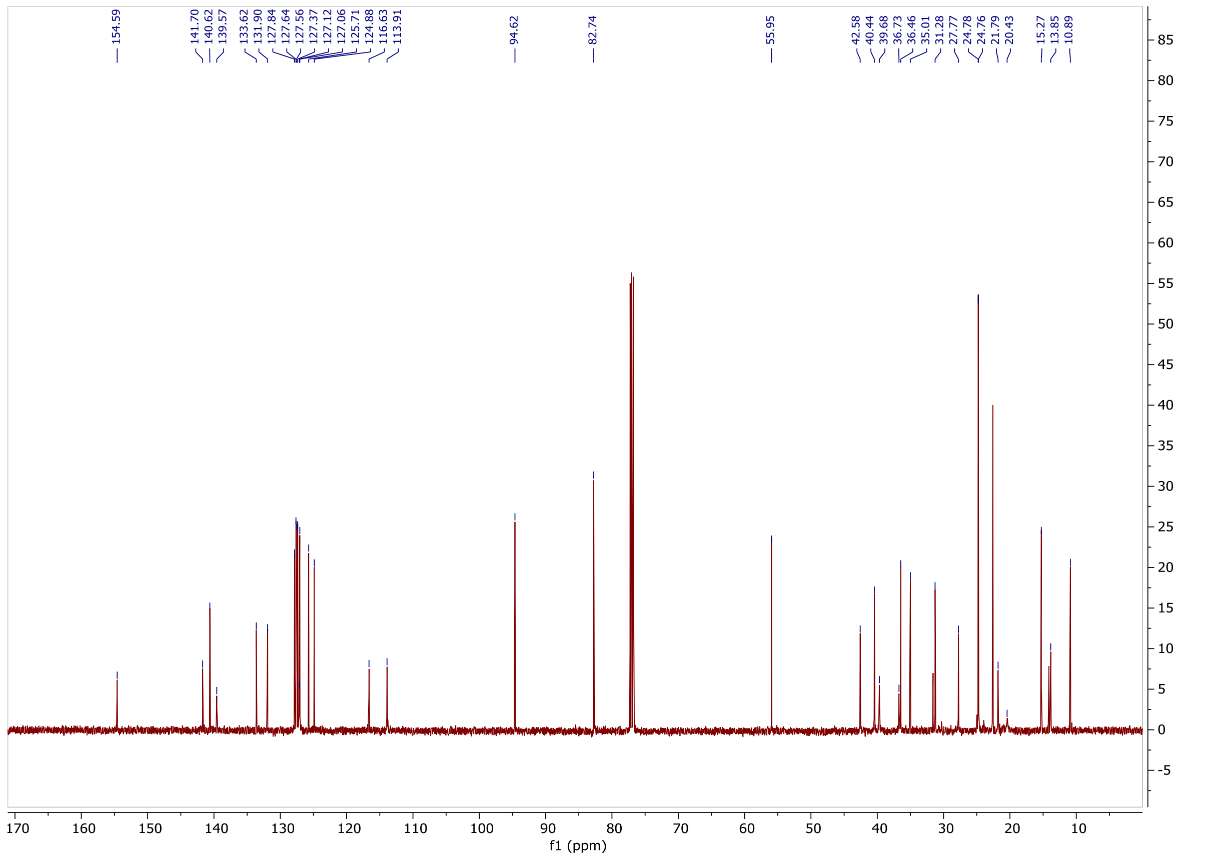

^{1}H-NMR (500 MHz, CDCl3) of compound **28**

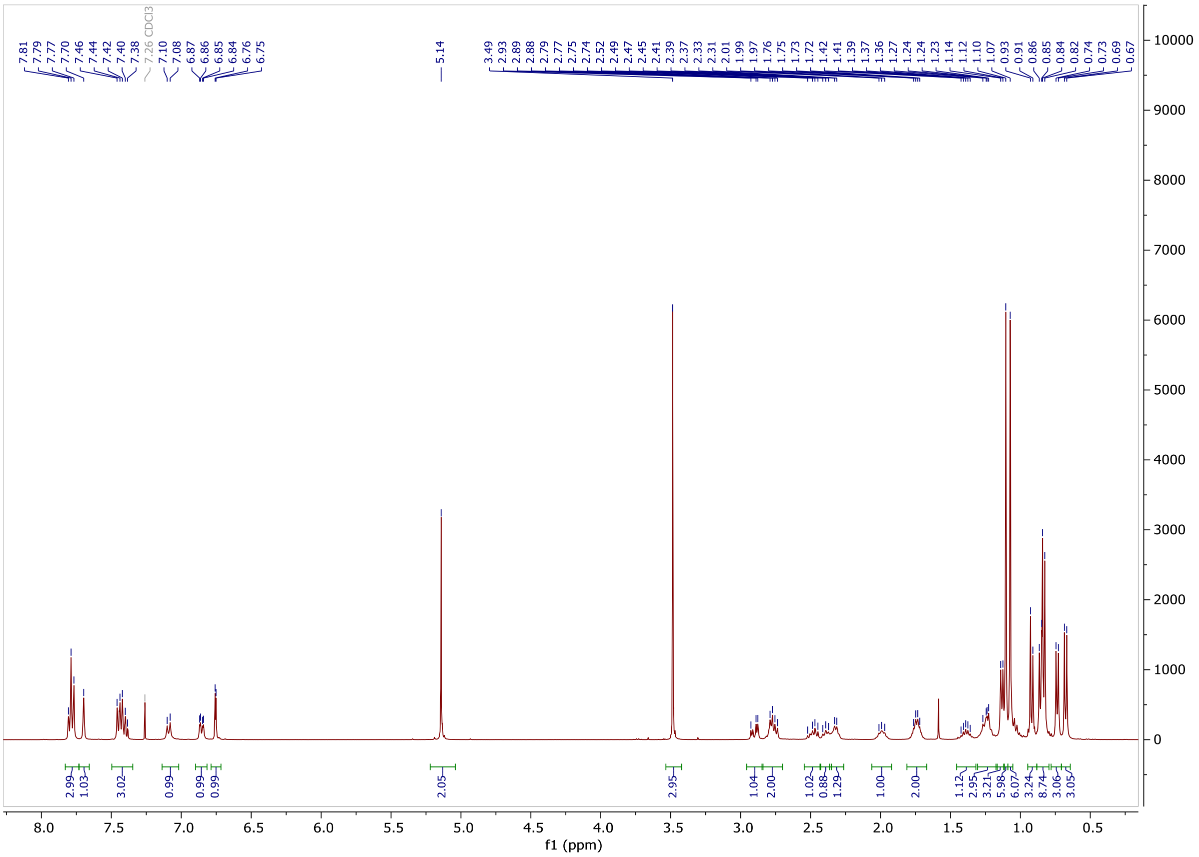

^{13}C-NMR (125 MHz, CDCl3) of compound **28**

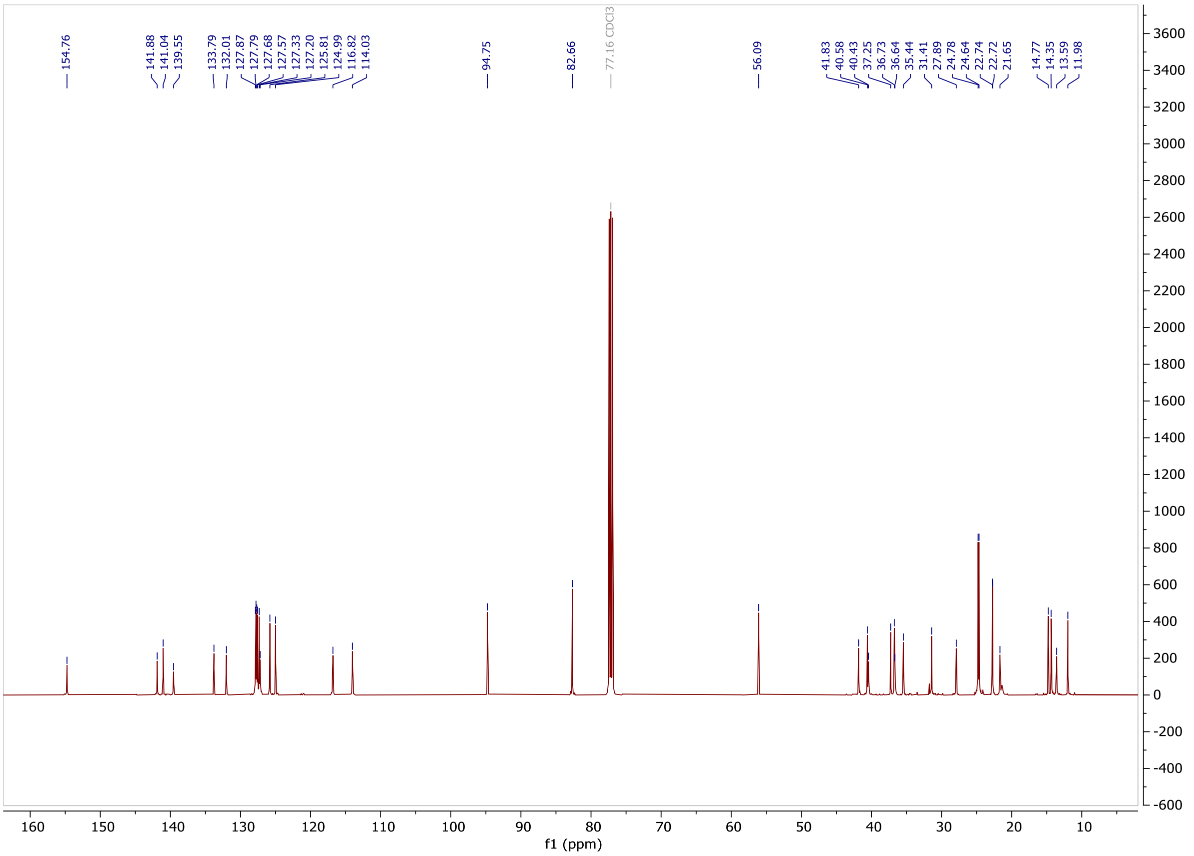

^{1}H-NMR (500 MHz, CDCl3) of compound **11**

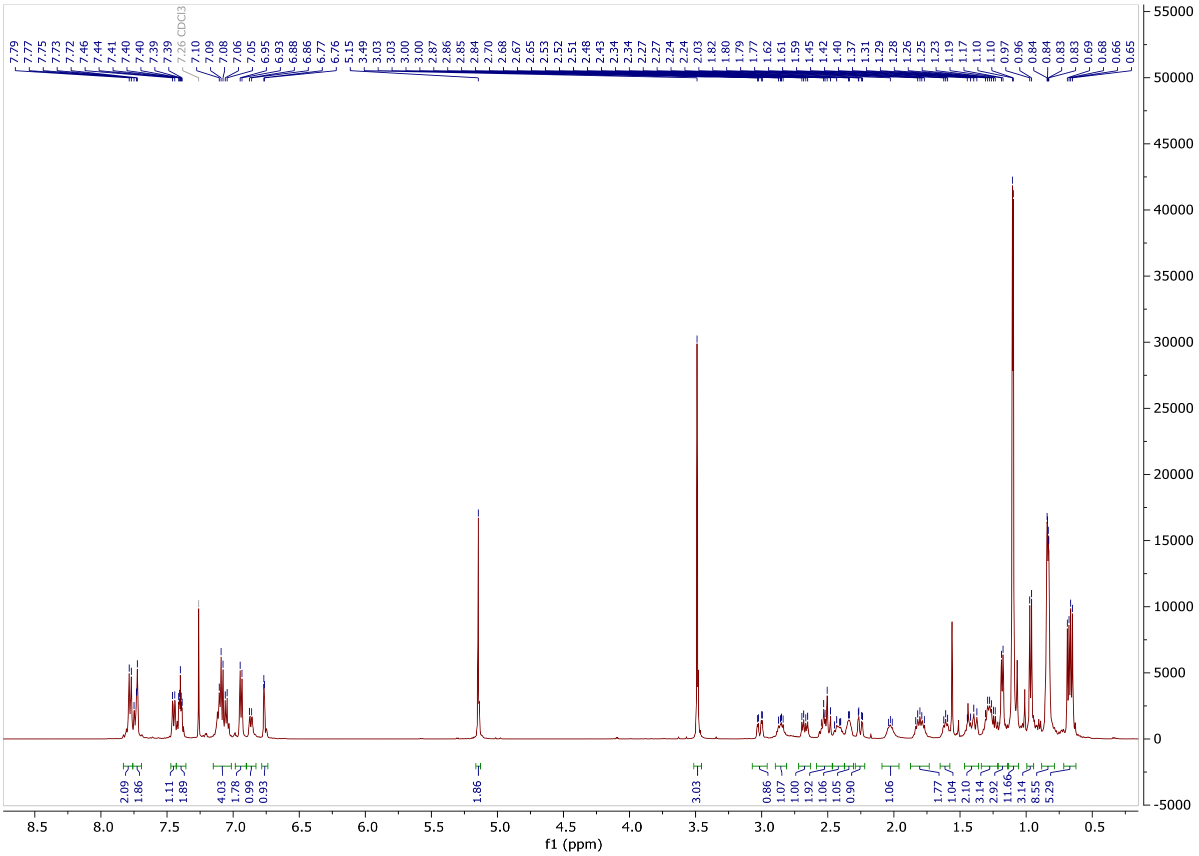

^{13}C-NMR (125 MHz, CDCl3) of compound **11**

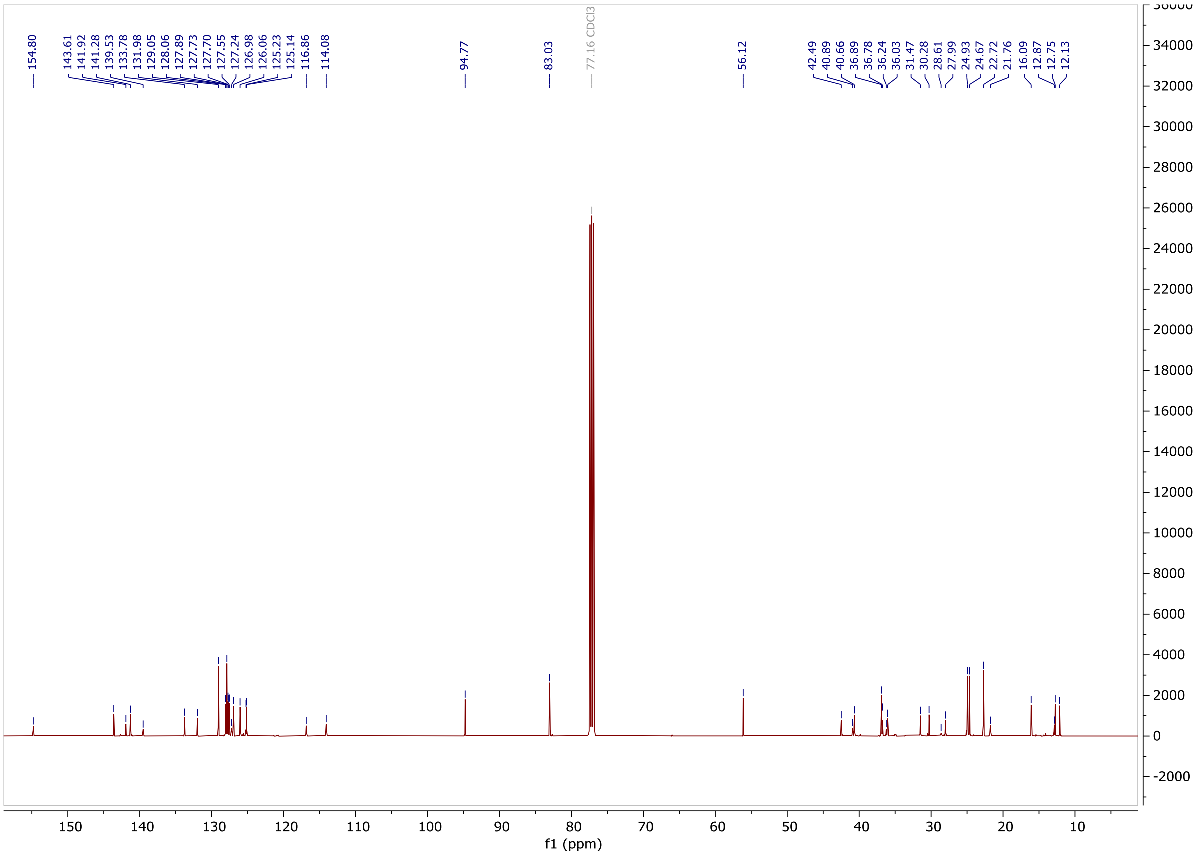

^{1}H-NMR (500 MHz, CDCl3) of compound **4**

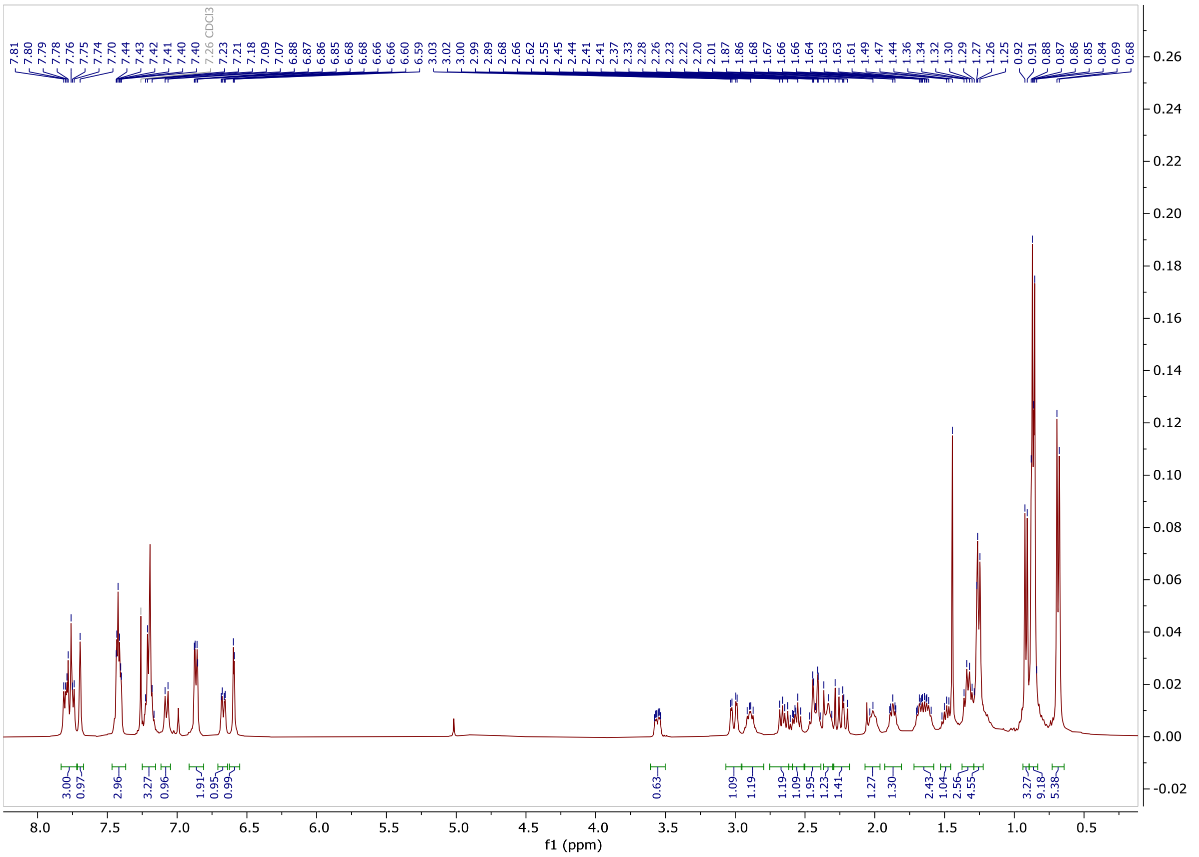

^{13}C-NMR (125 MHz, CDCl3) of compound **4**

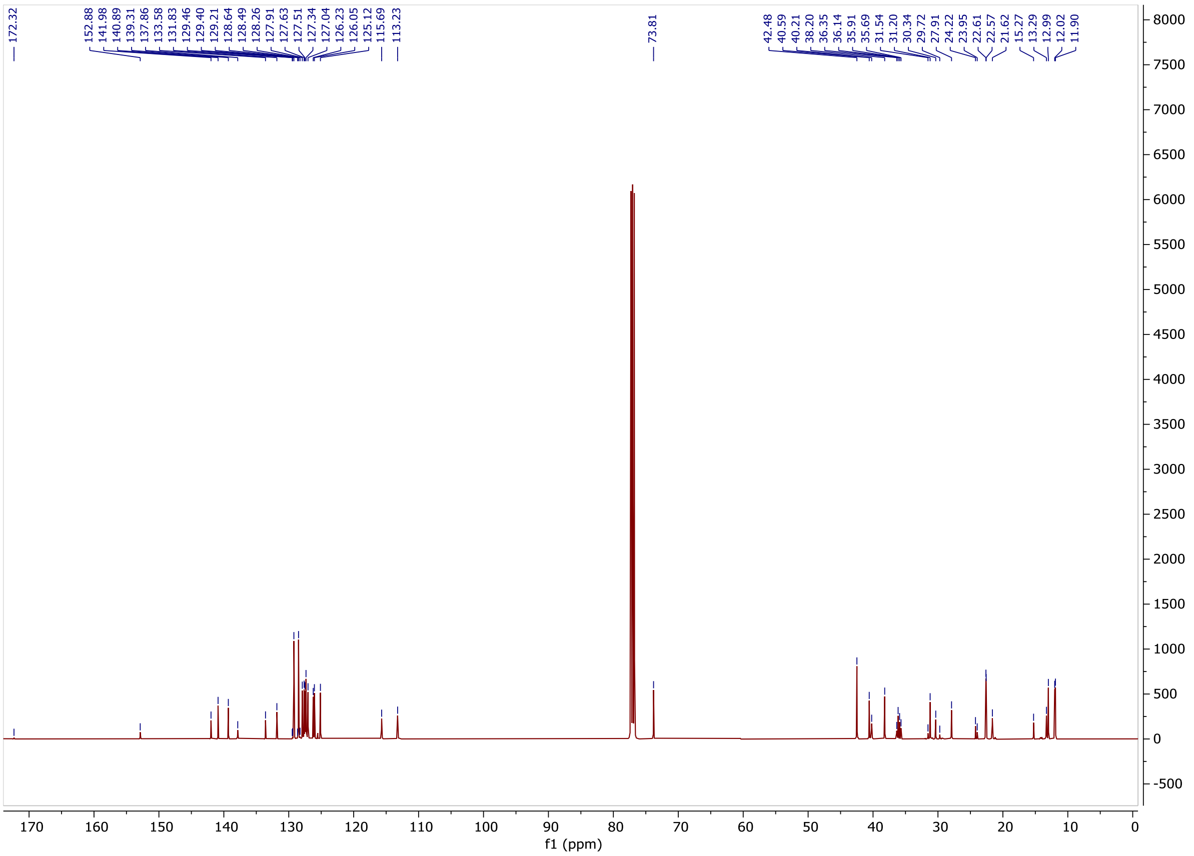

^{1}H-NMR (500 MHz, CDCl3) of compound **13**

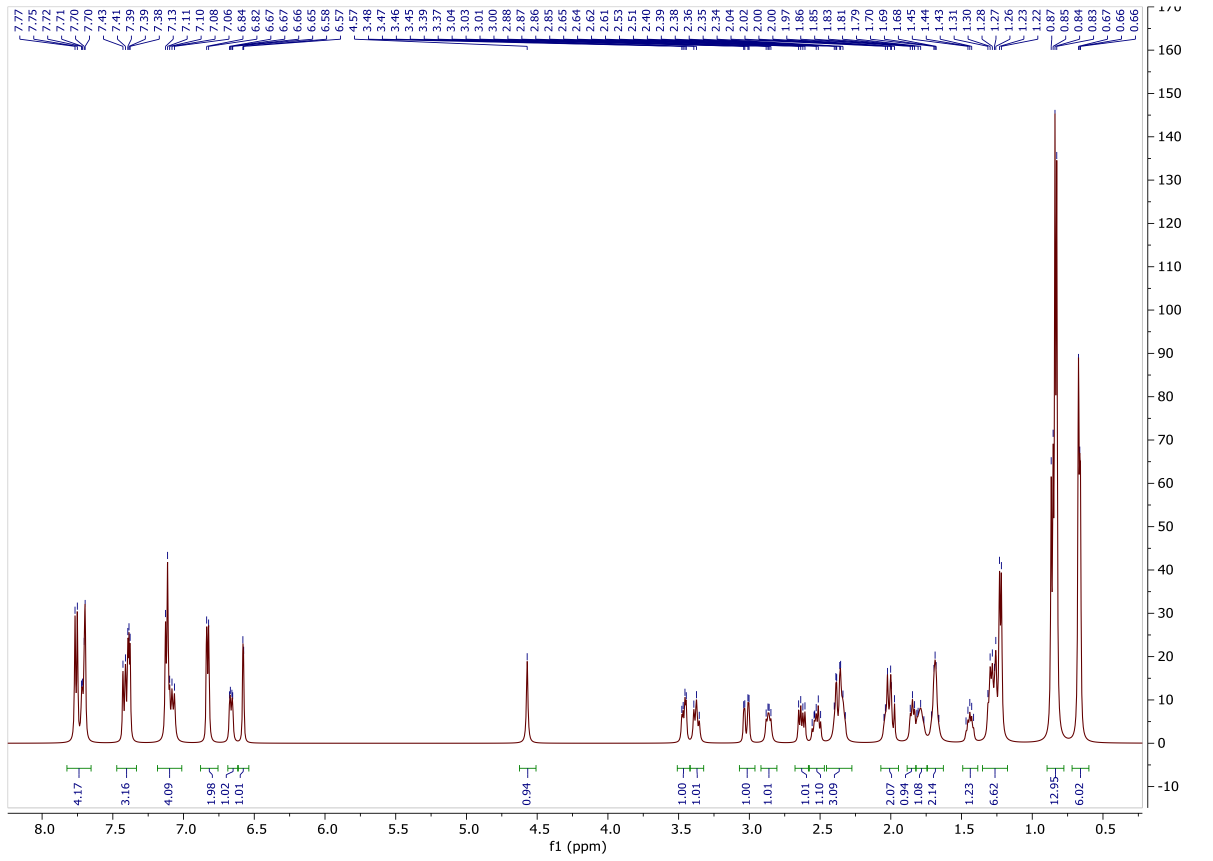

^{13}C-NMR (125 MHz, CDCl3) of compound **13**

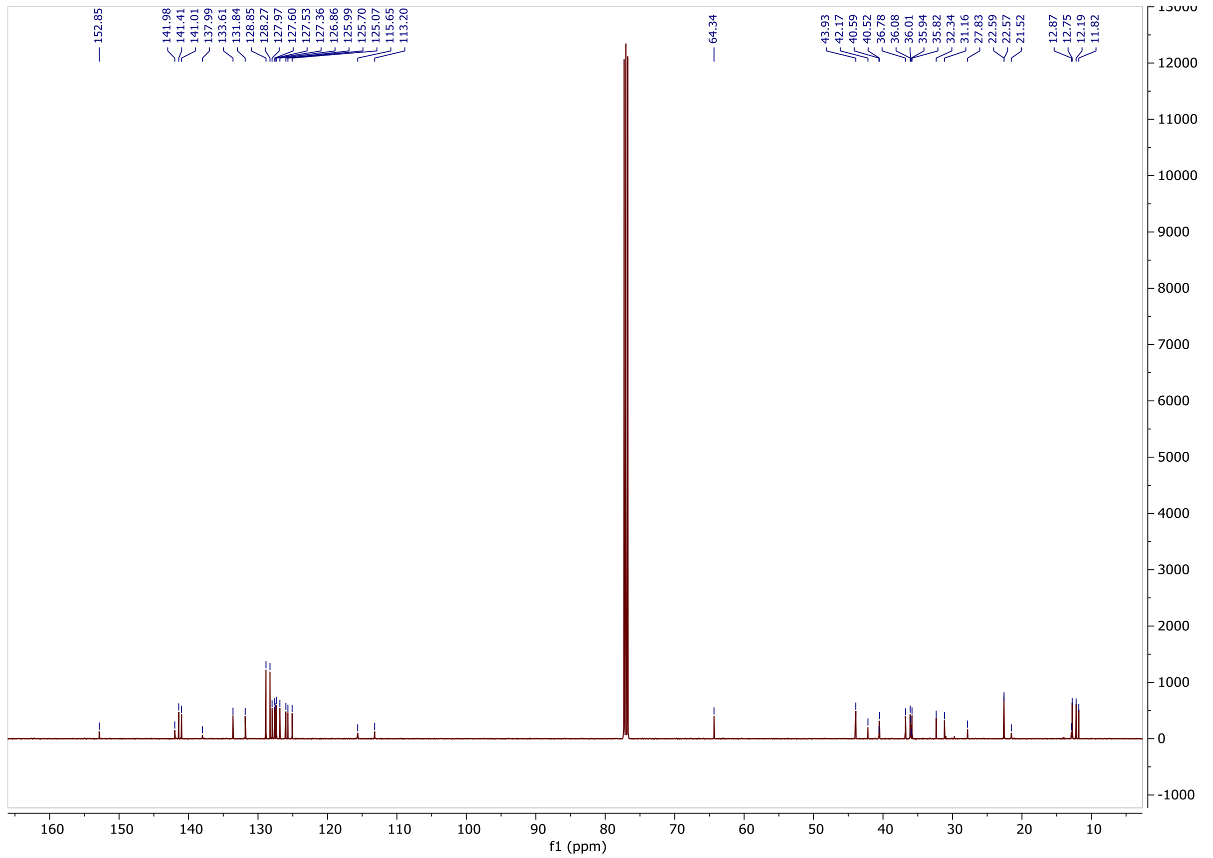

^{1}H-NMR (500 MHz, MeOD) of compound **15**

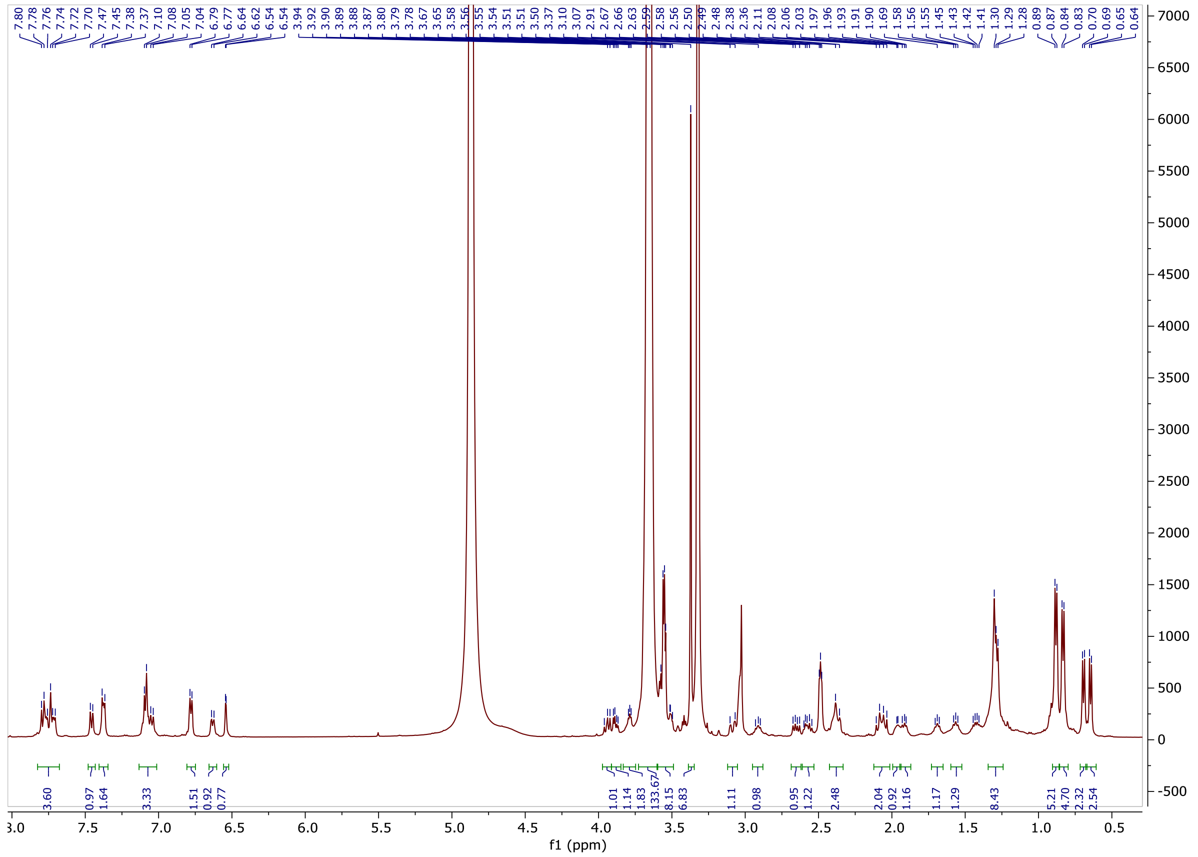

^{13}C-NMR (125 MHz, MeOD) of compound **15**

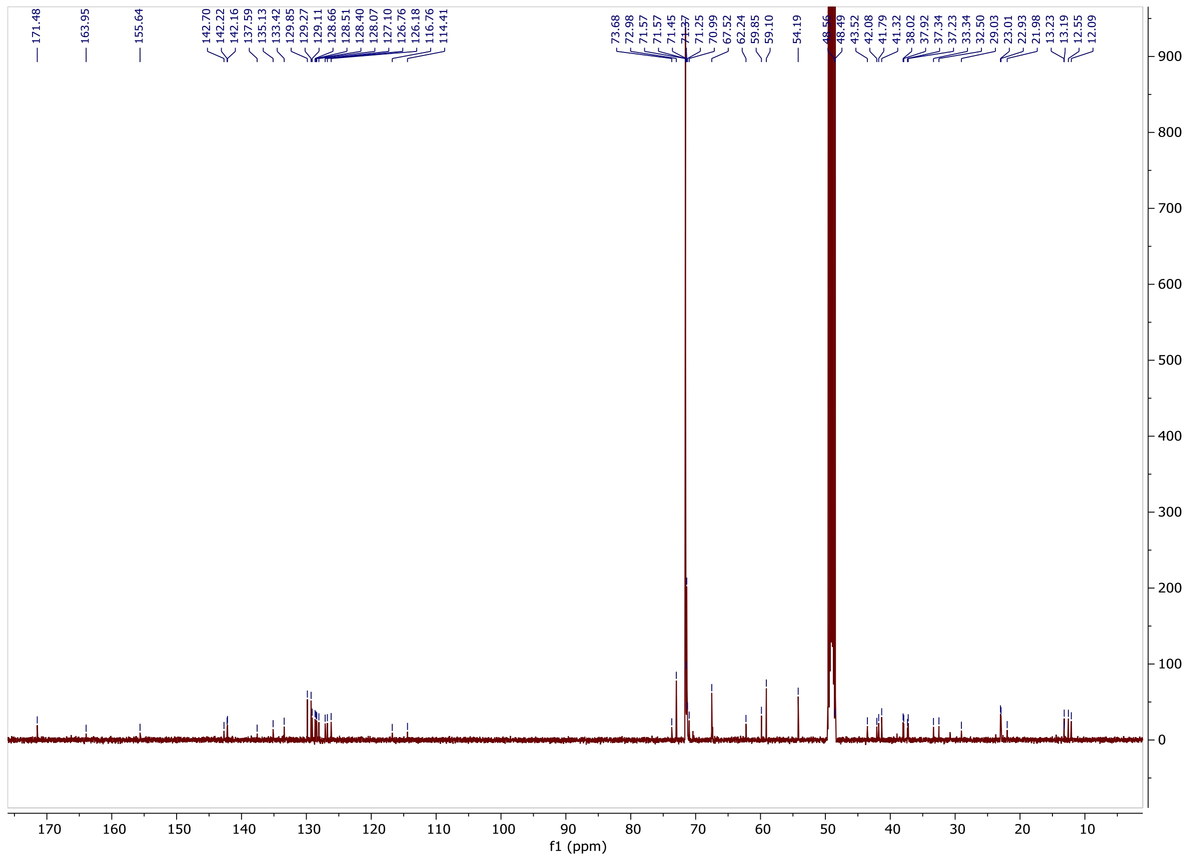

^{1}H-NMR (500 MHz, CDCl3) of compound **30**

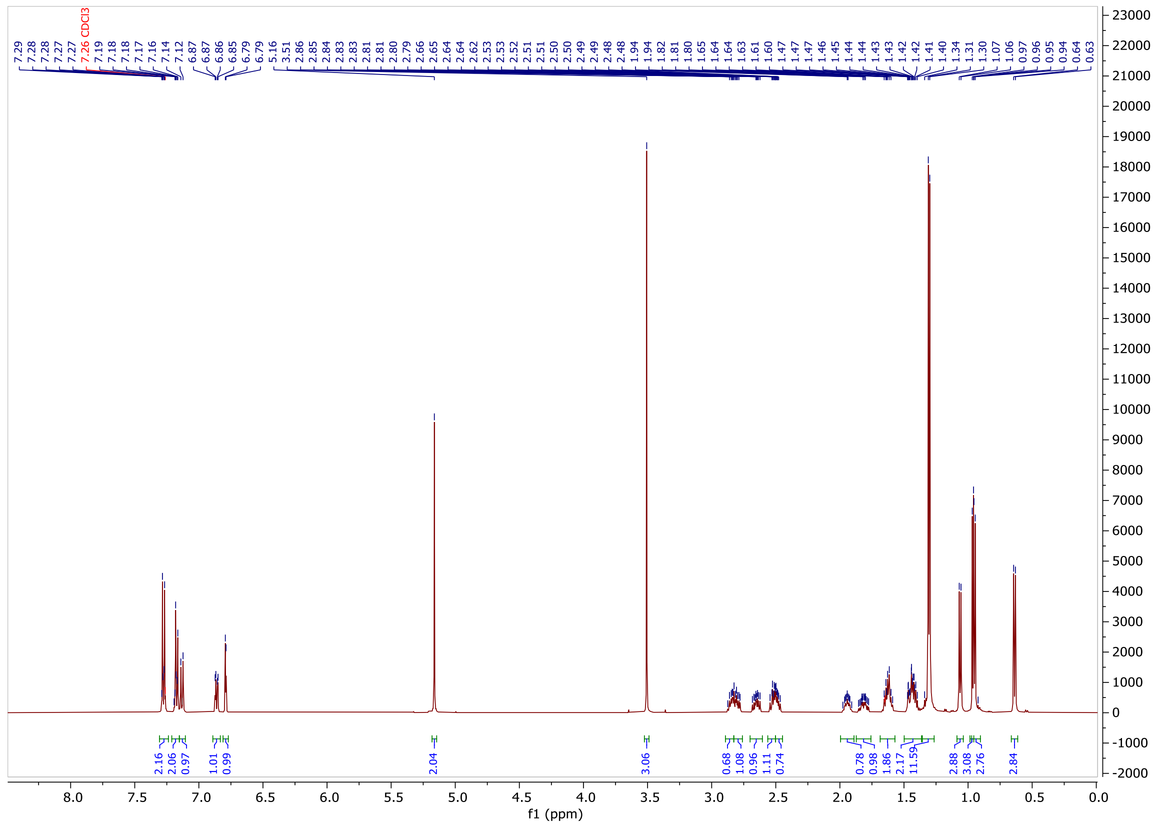

^{13}C-NMR (125 MHz, CDCl3) of compound **30**

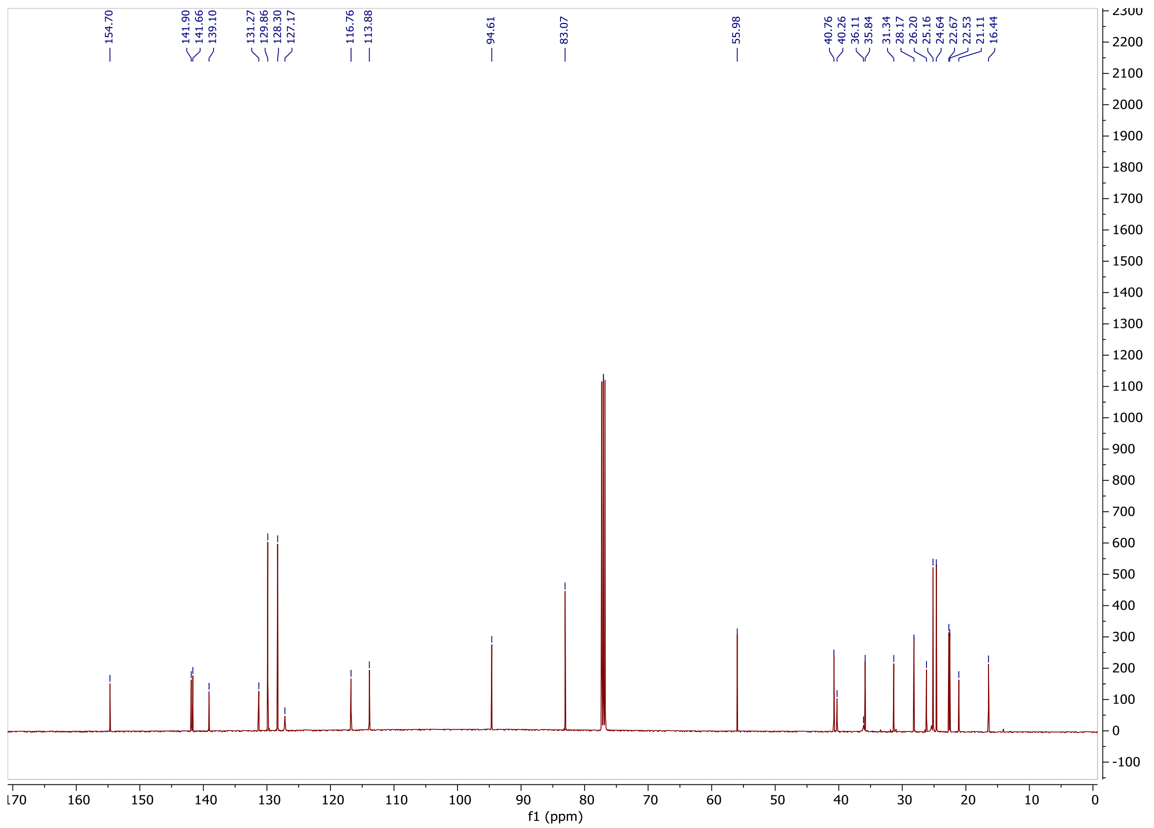

^{1}H-NMR (500 MHz, CDCl3) of compound **31**

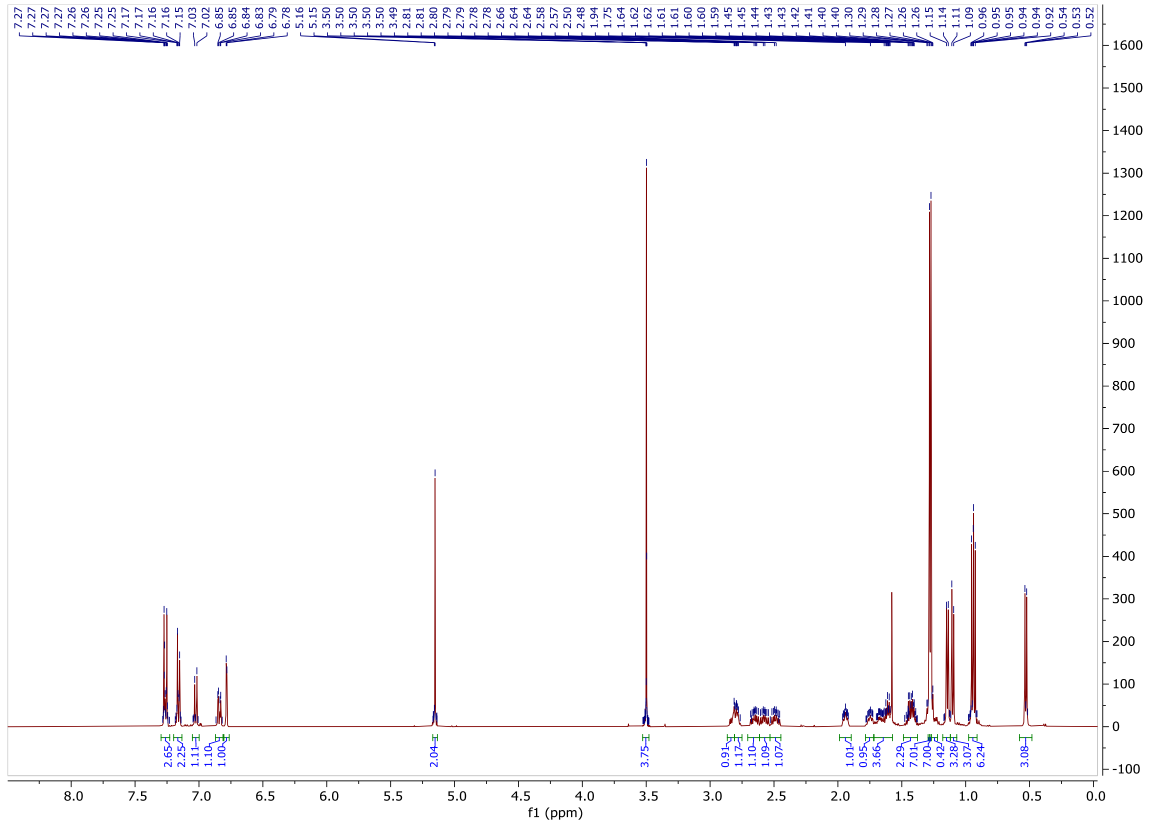

^{13}C-NMR (125 MHz, CDCl3) of compound **31**

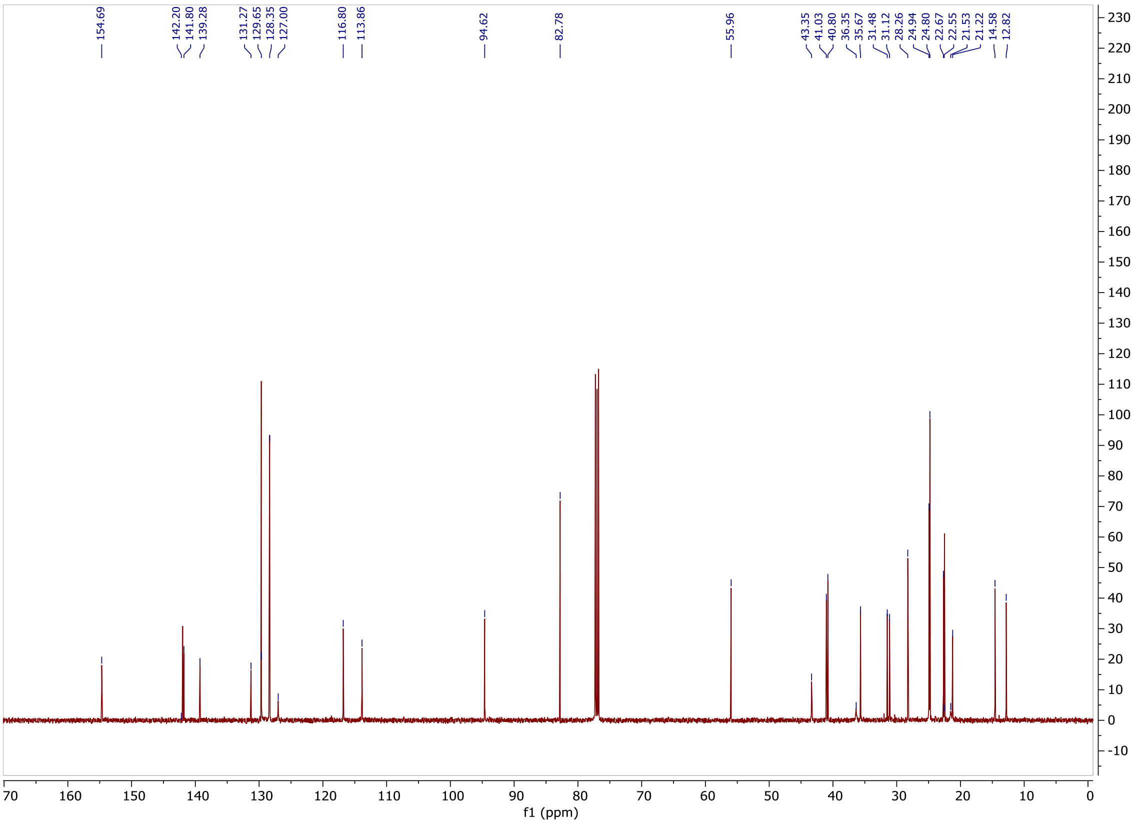

^{1}H-NMR (500 MHz, CDCl3) of compound **32**

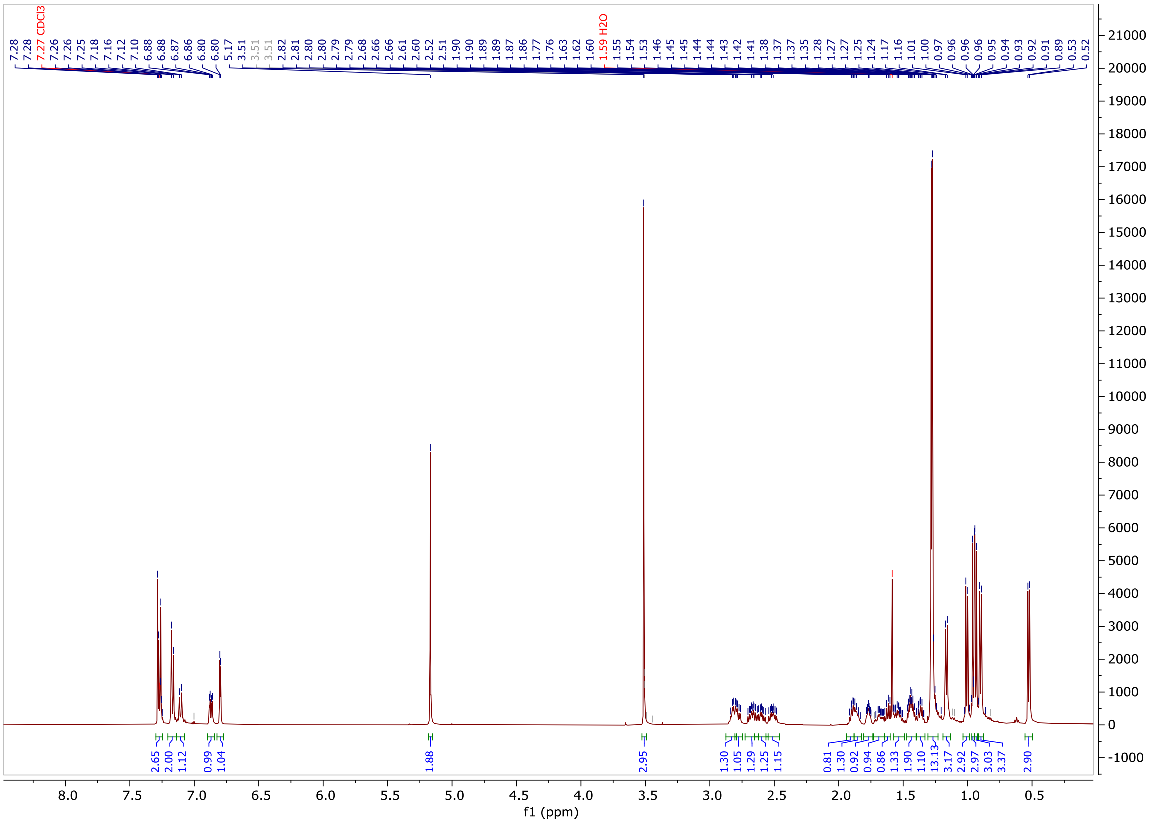

^{13}C-NMR (125 MHz, CDCl3) of compound **32**

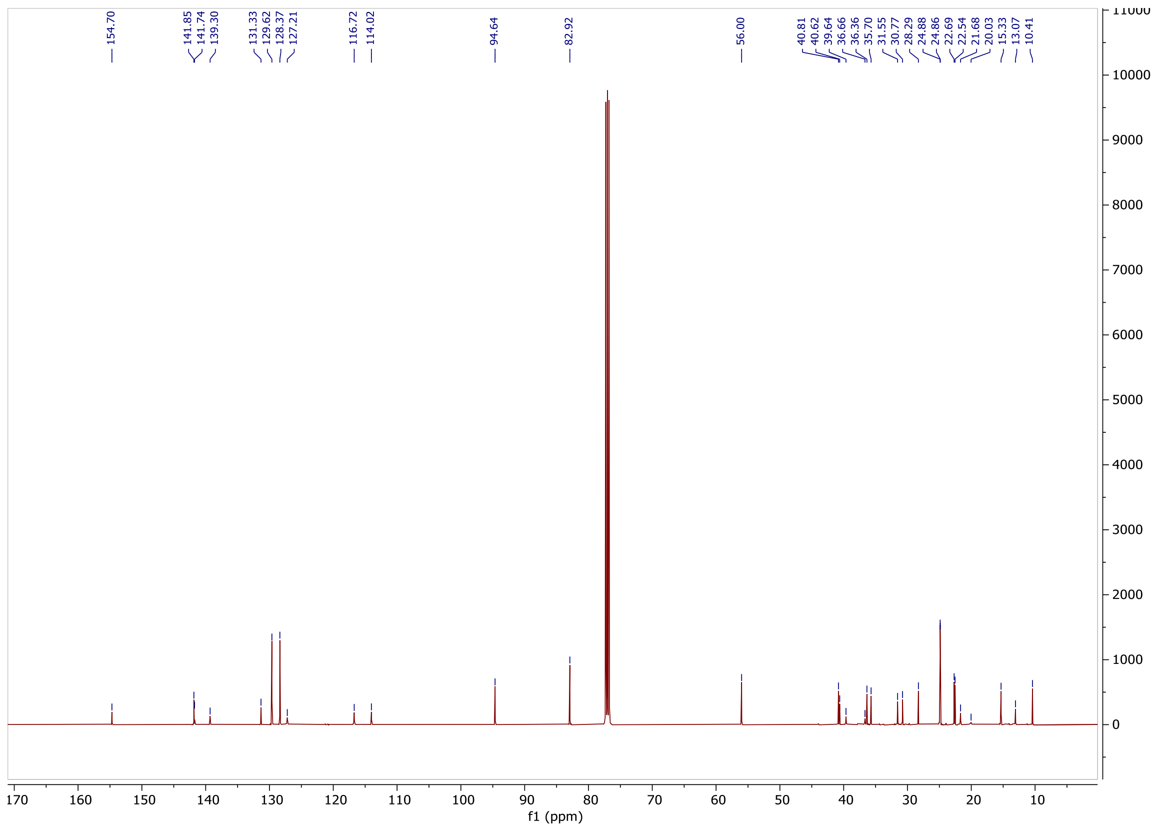

^{1}H-NMR (500 MHz, CDCl3) of compound **33**

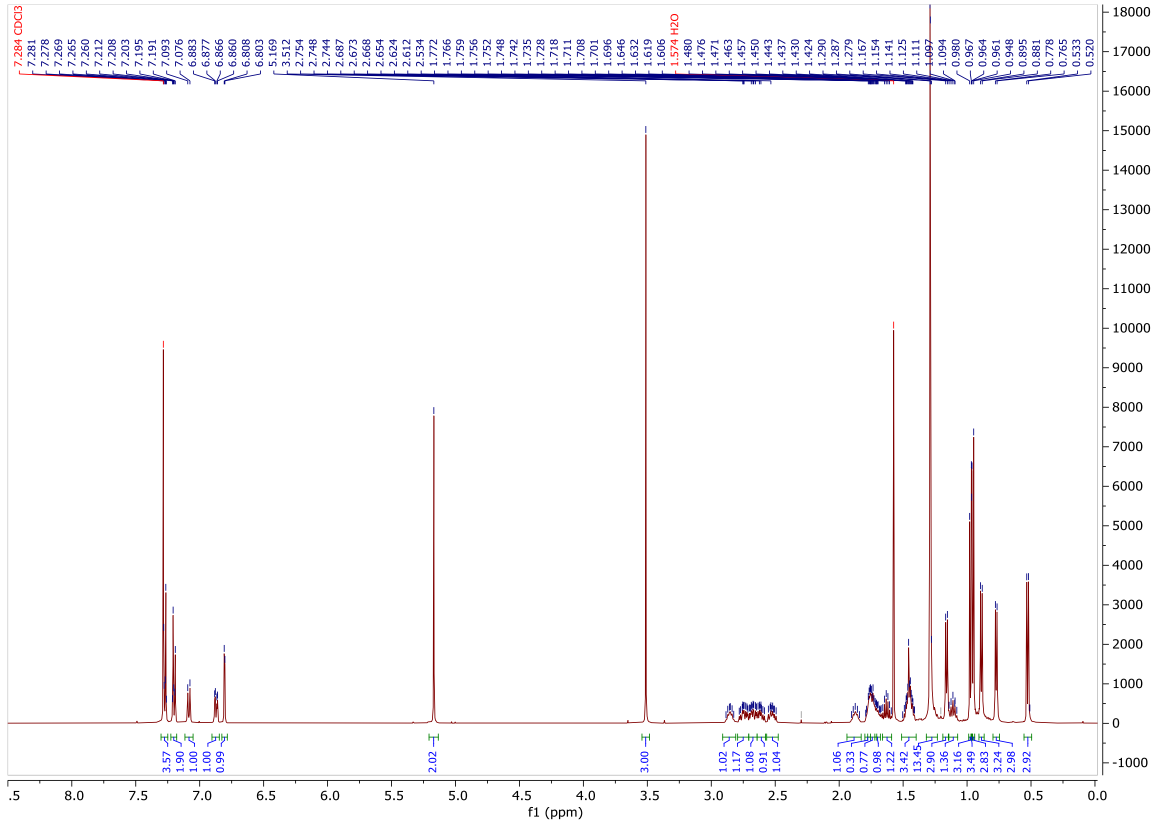

^{13}C-NMR (125 MHz, CDCl3) of compound **33**

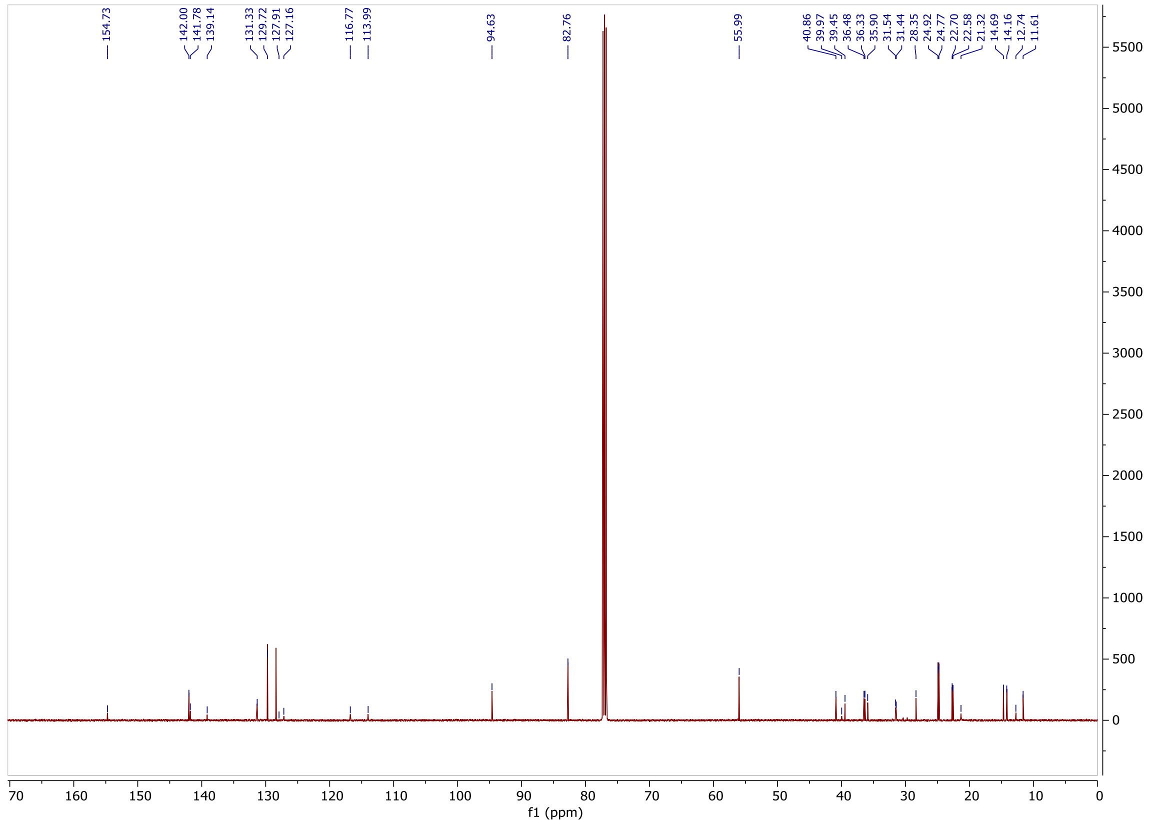

^{1}H-NMR (500 MHz, CDCl3) of compound **12**

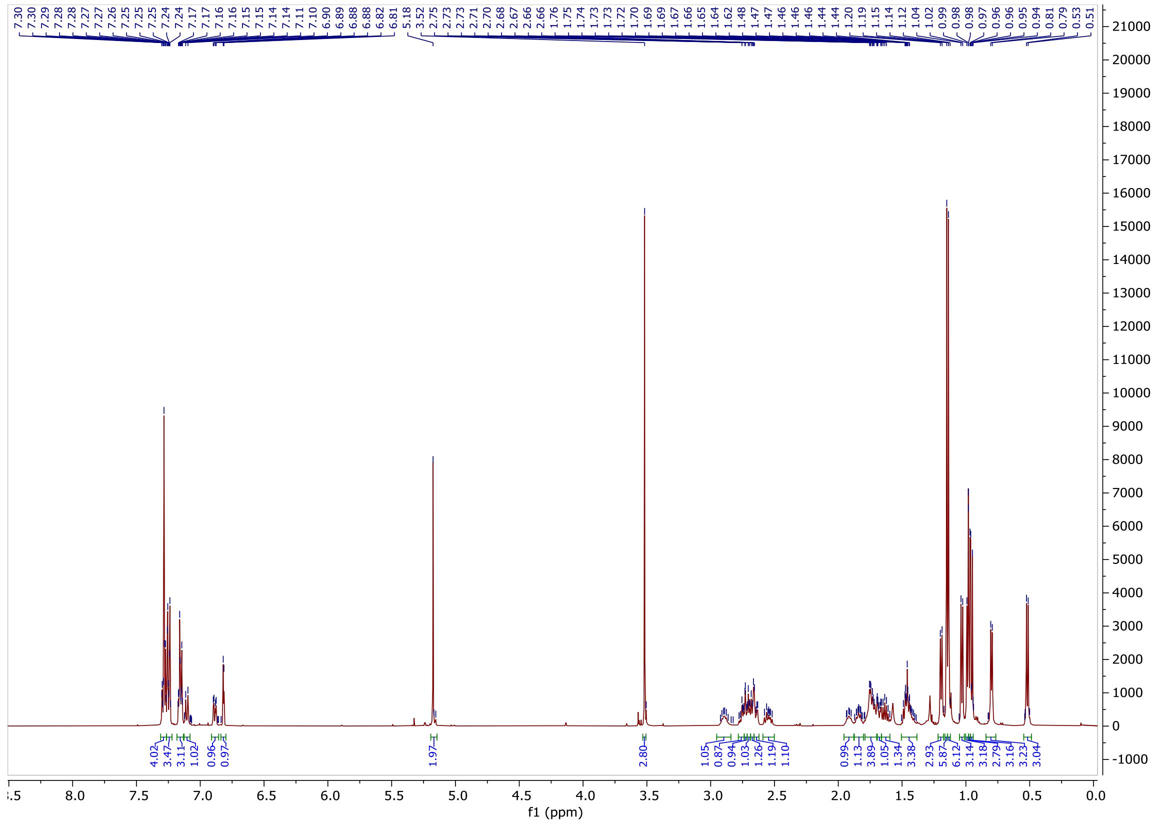

^{13}C-NMR (125 MHz, CDCl3) of compound **12**

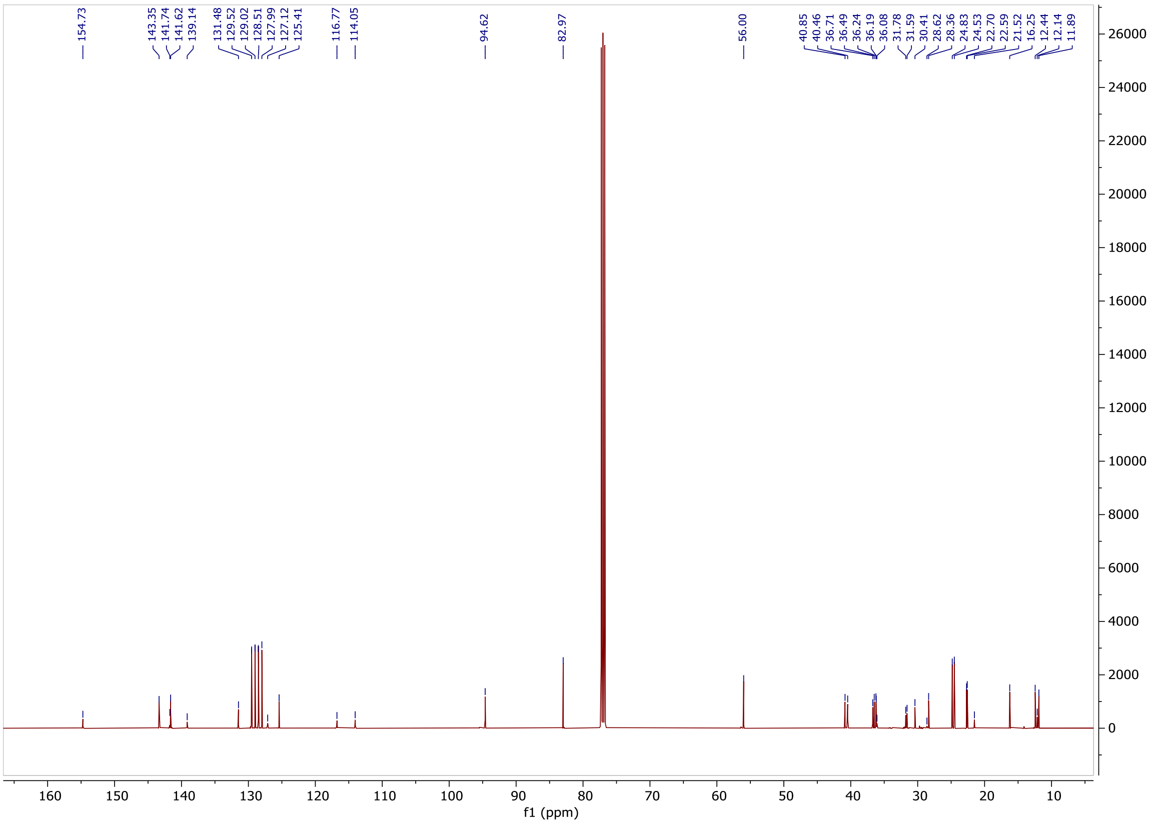

^{1}H-NMR (500 MHz, CDCl3) of compound **14**

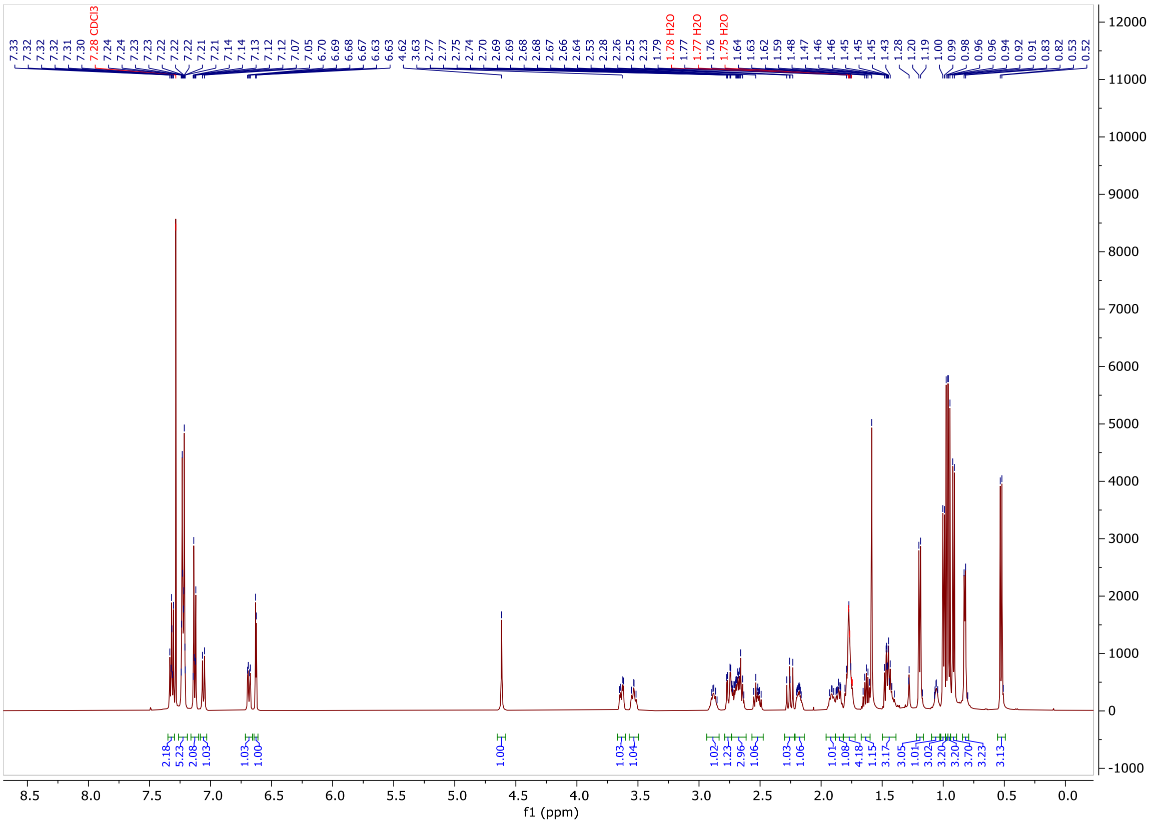

^{13}C-NMR (125 MHz, CDCl3) of compound **14**

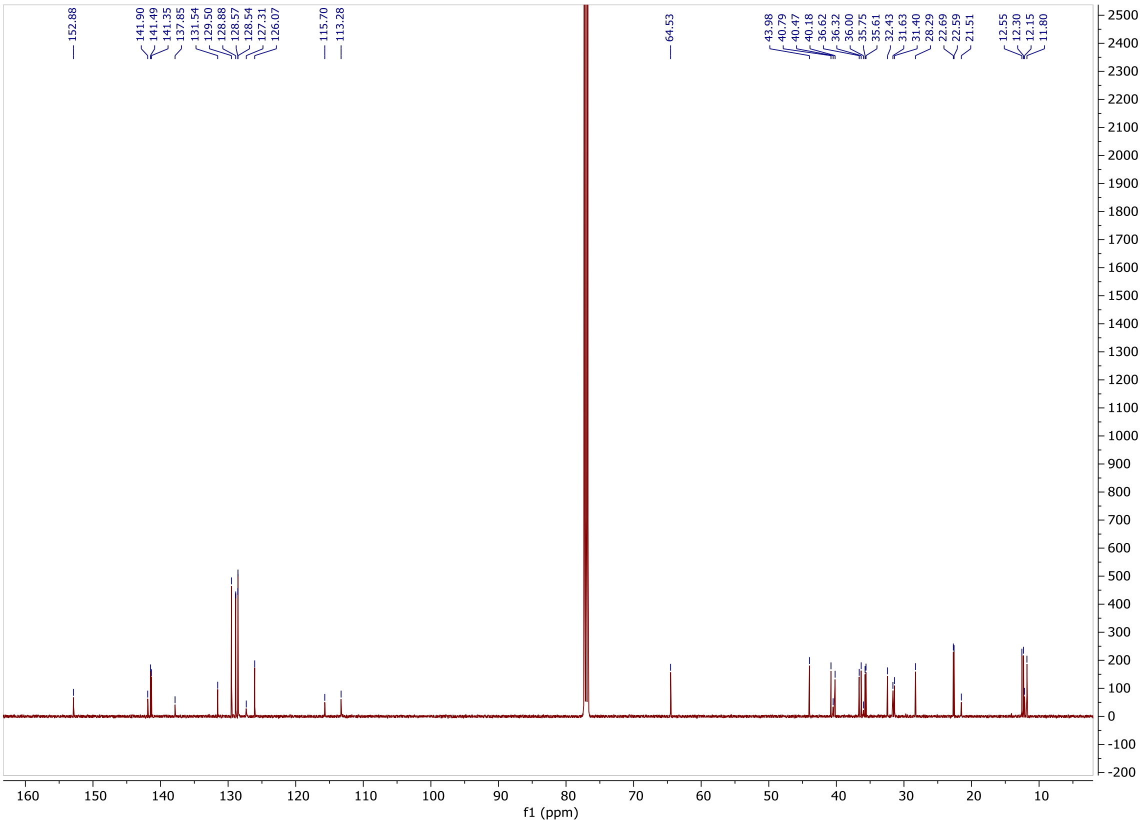

^{1}H-NMR (500 MHz, MeOD) of compound **16**

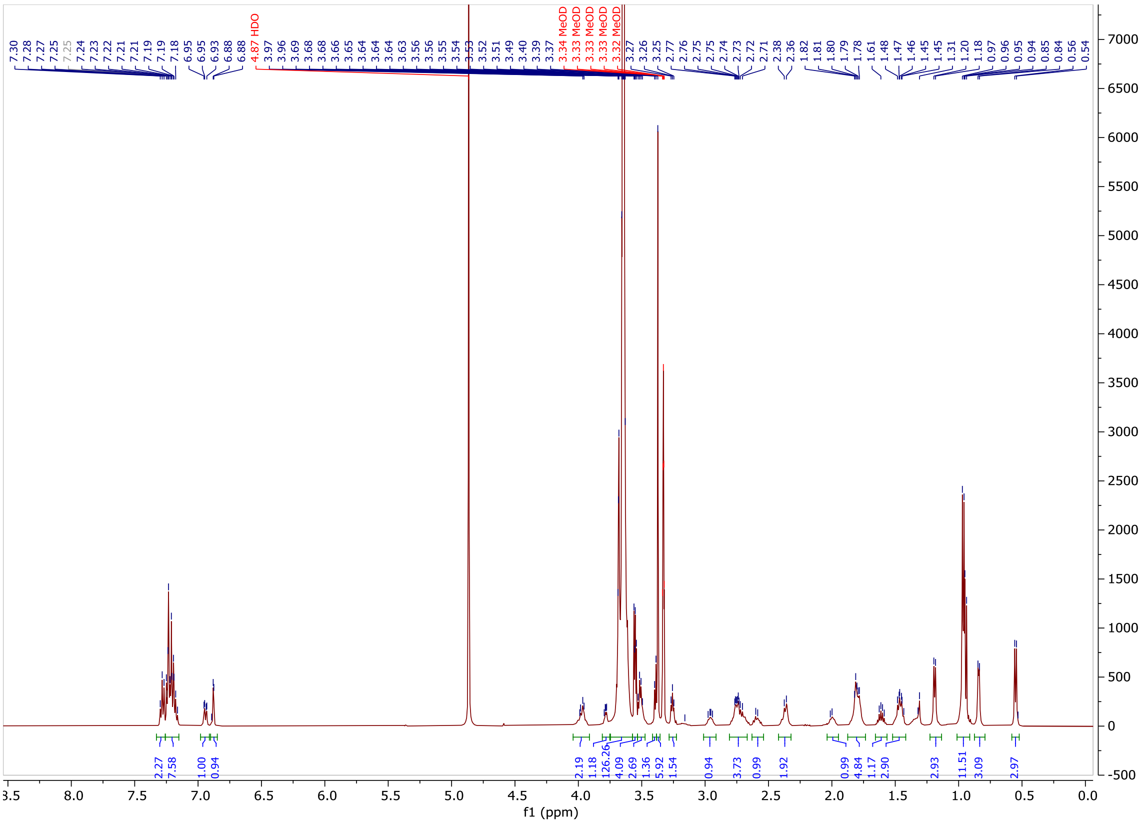

^{13}C-NMR (125 MHz, MeOD) of compound **16**

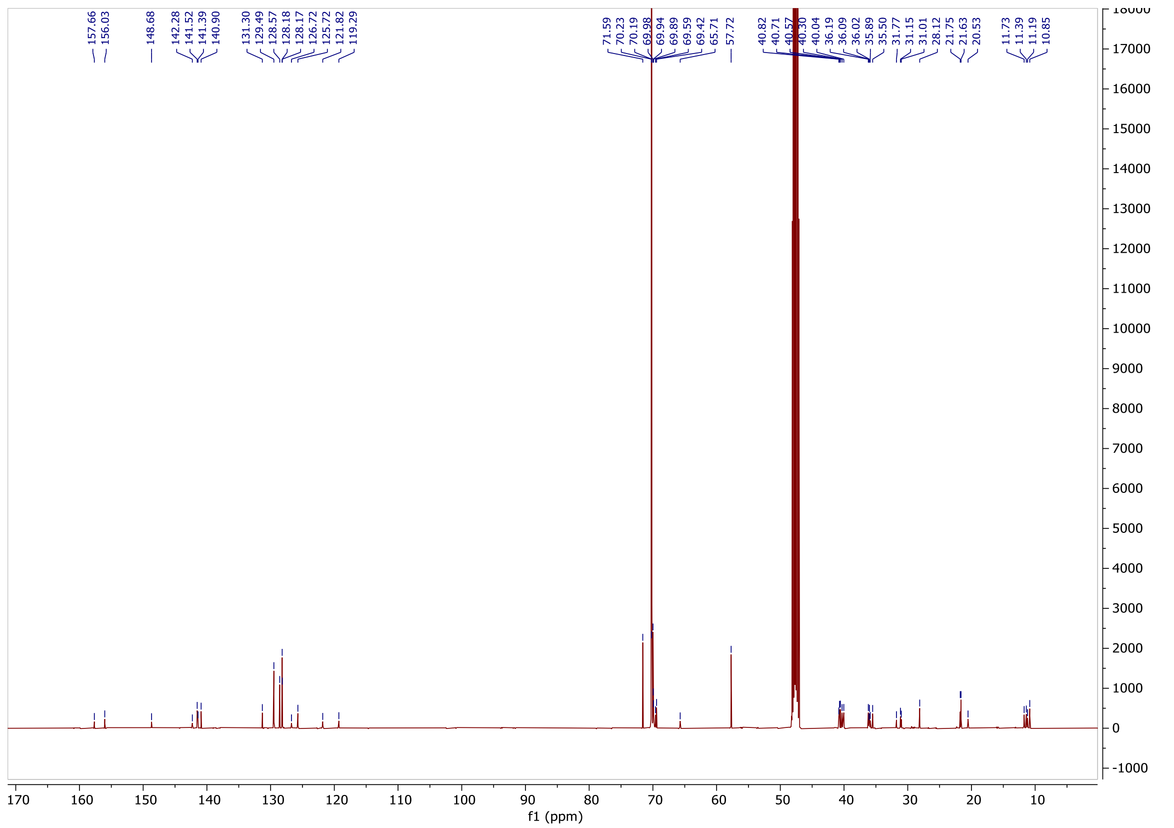

^{1}H-NMR (500 MHz, CDCl3) of compound **35**

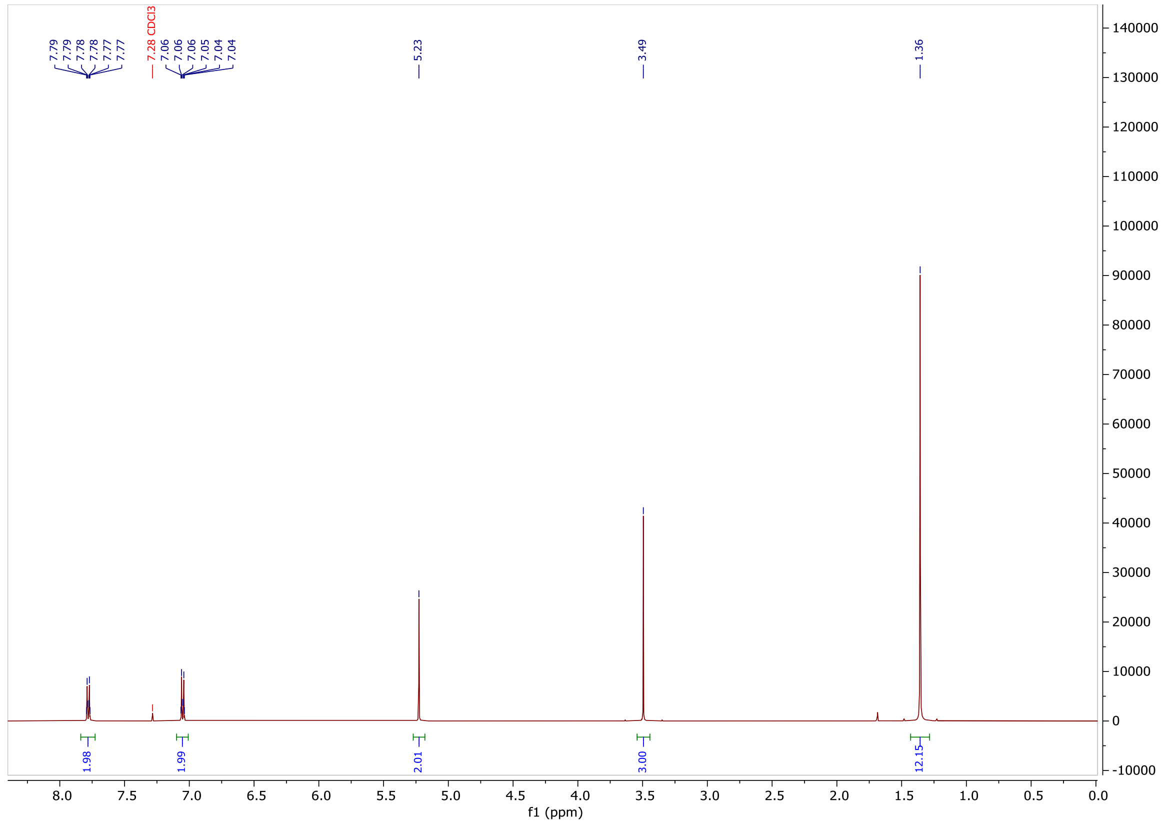

^{13}C-NMR (125 MHz, CDCl3) of compound **35**

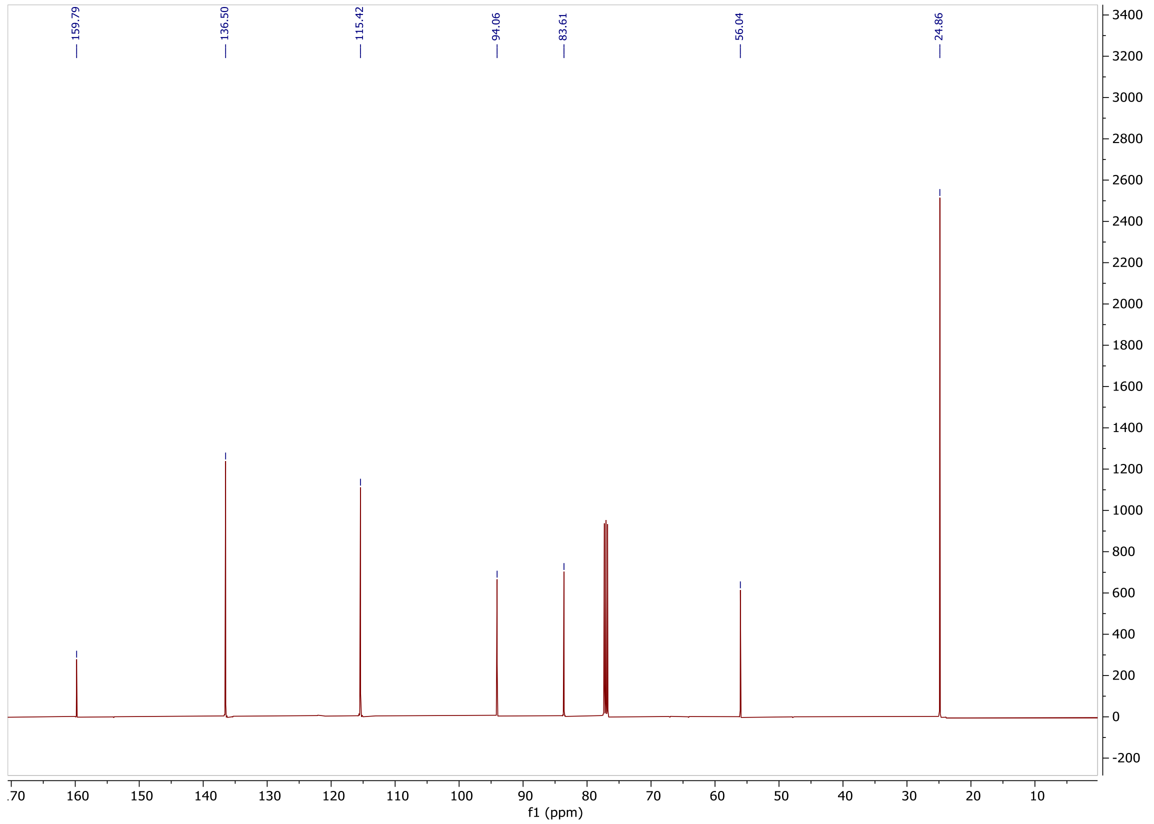

^{1}H-NMR (500 MHz, CDCl3) of compound **36**

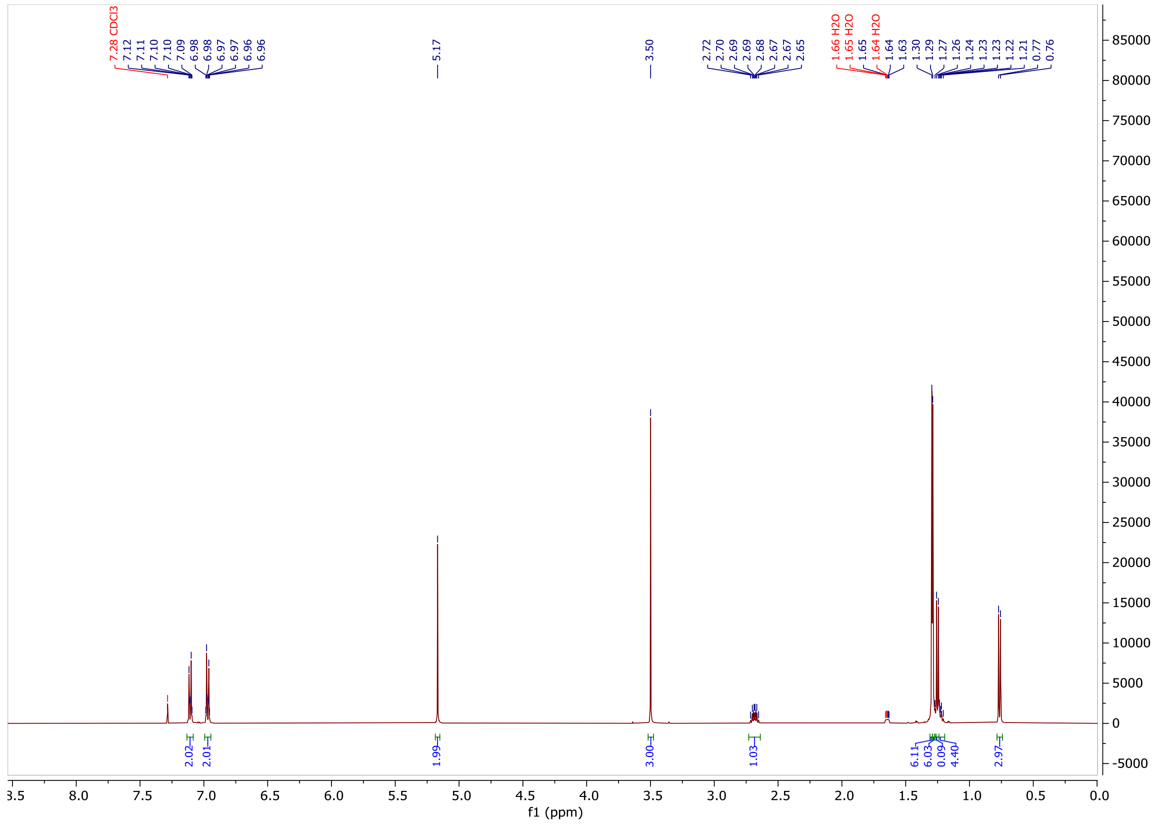

^{13}C-NMR (125 MHz, CDCl3) of compound **36**

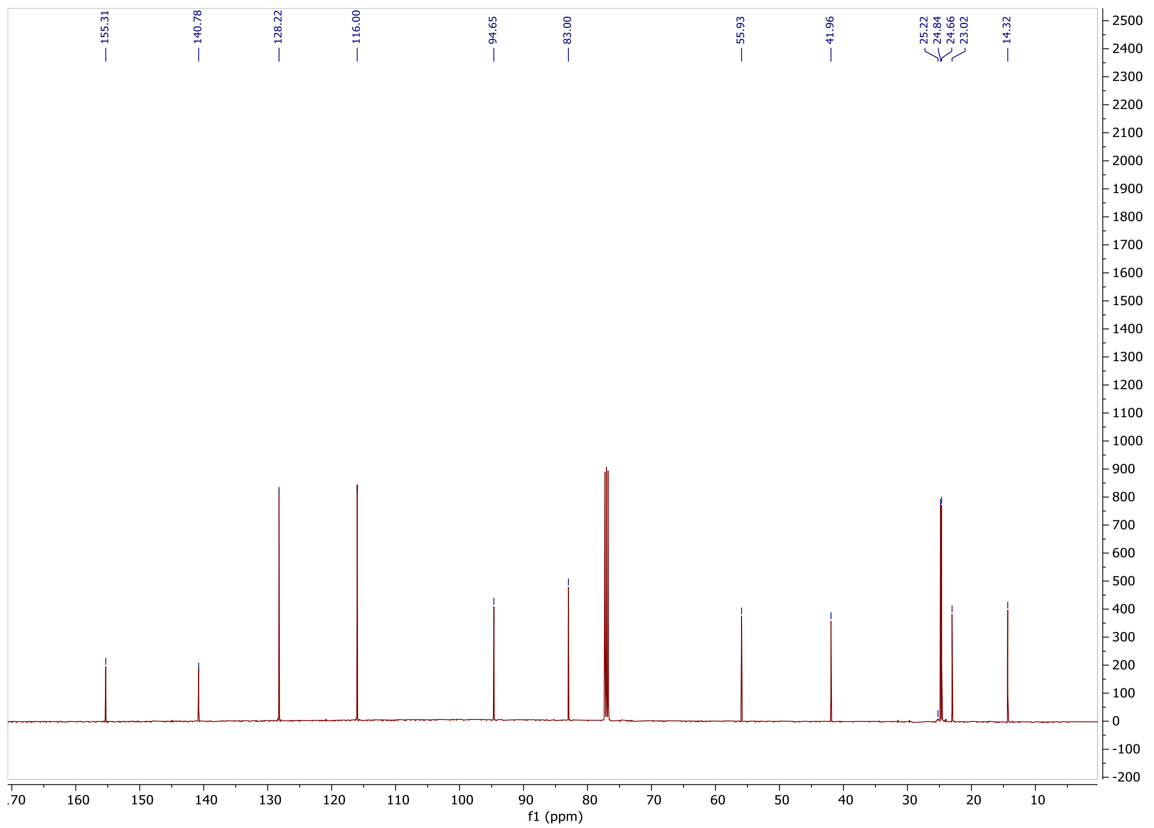

^{1}H-NMR (500 MHz, CDCl3) of compound **37**

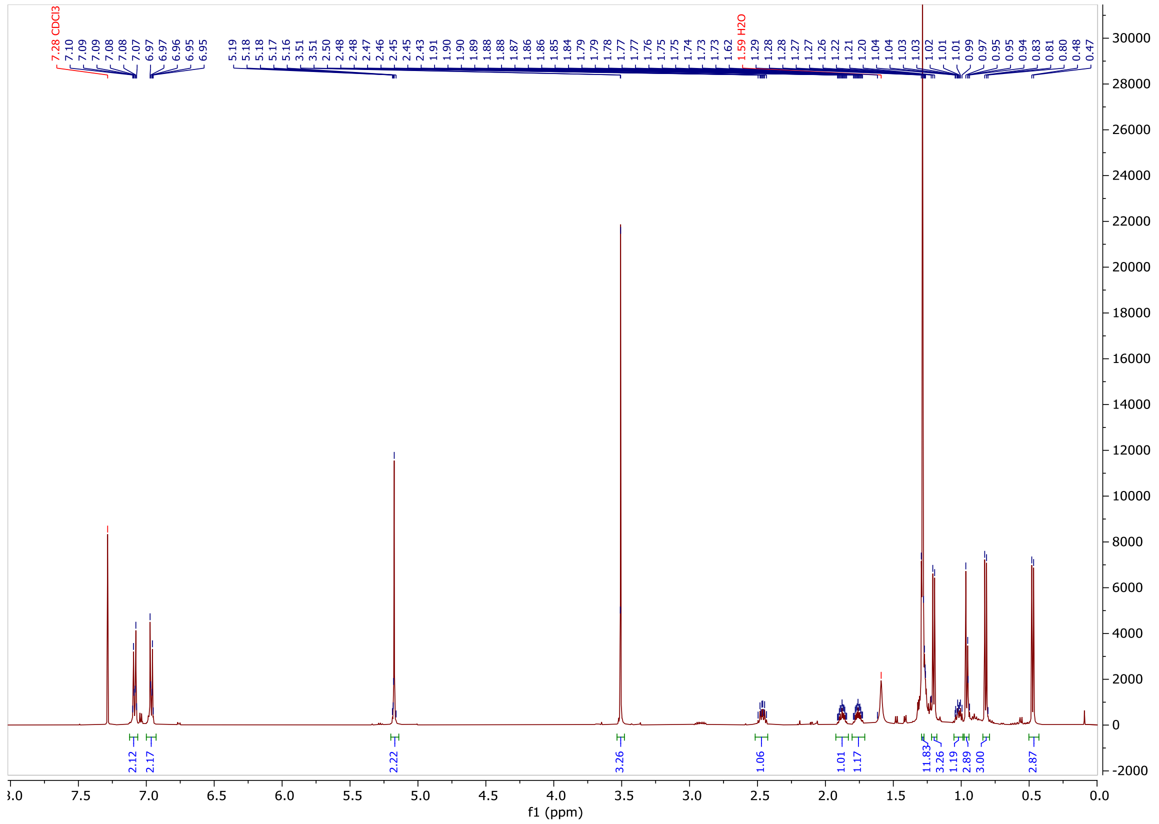

^{13}C-NMR (125 MHz, CDCl3) of compound **37**

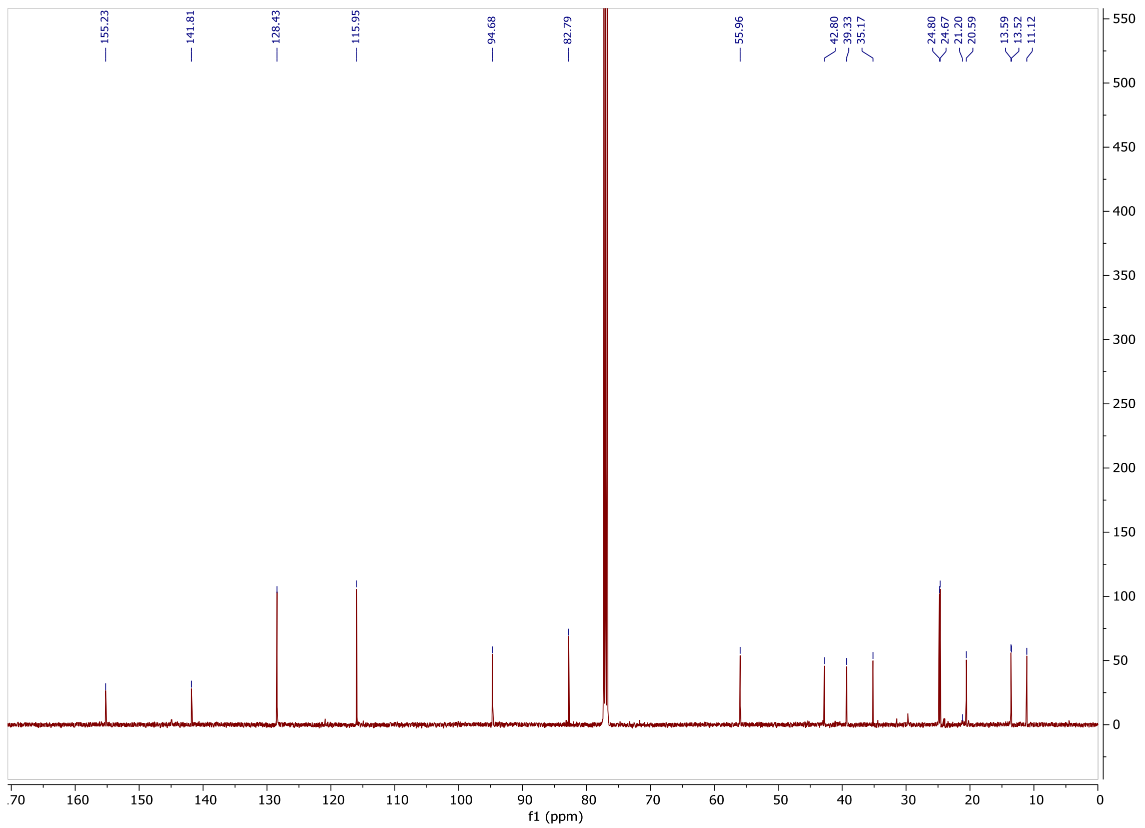

^{1}H-NMR (500 MHz, CDCl3) of compound **38**

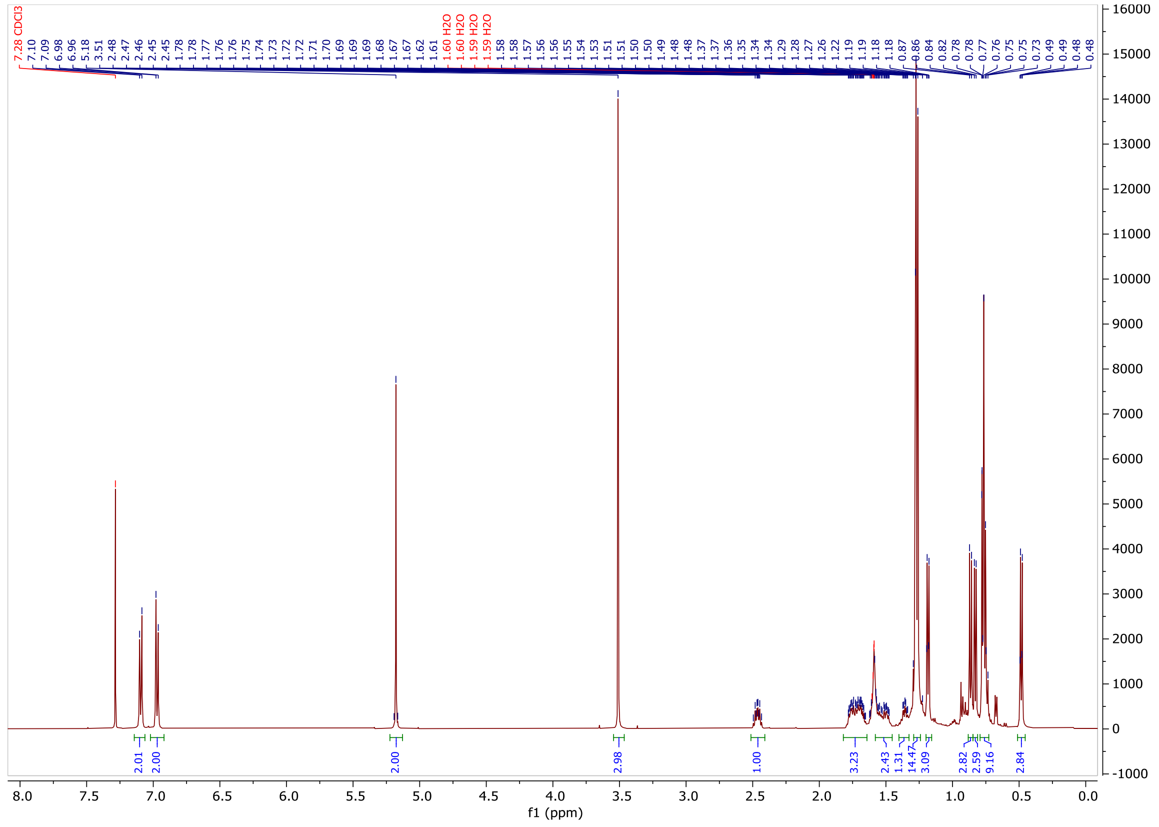

^{13}C-NMR (125 MHz, CDCl3) of compound **38**

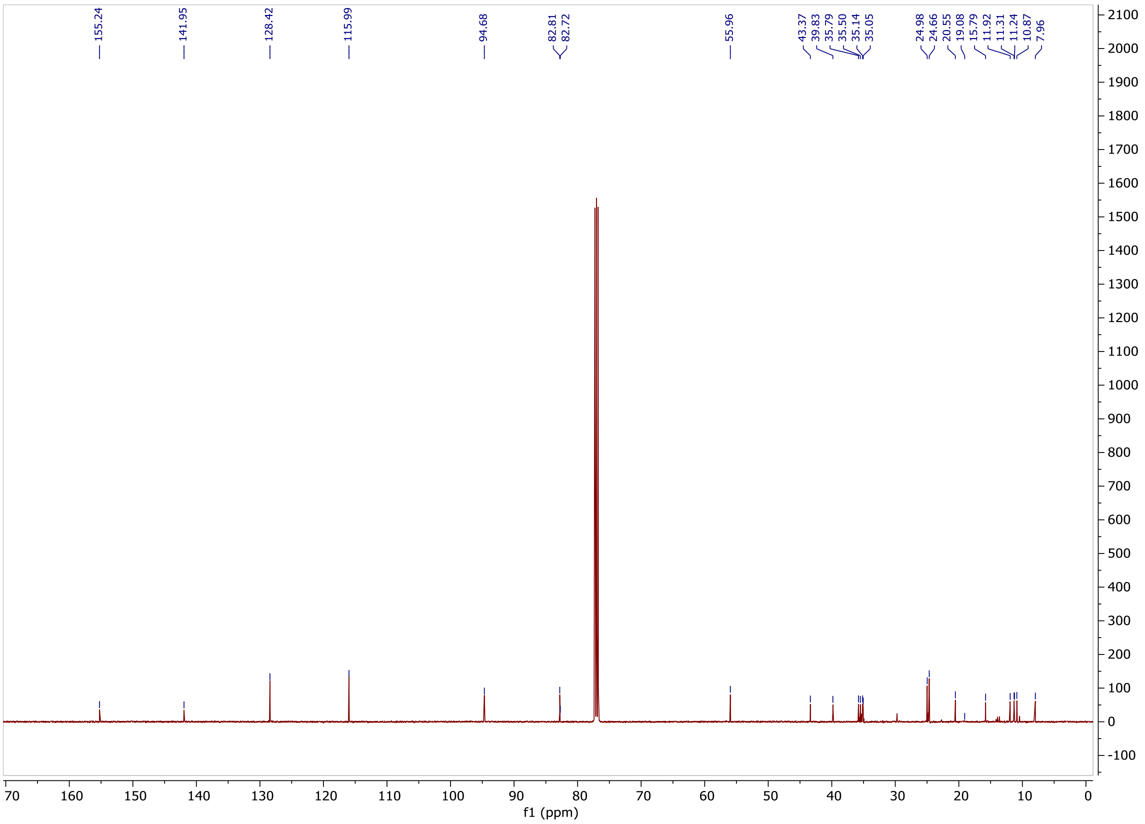

^{1}H-NMR (500 MHz, MeOD) of compound **40**

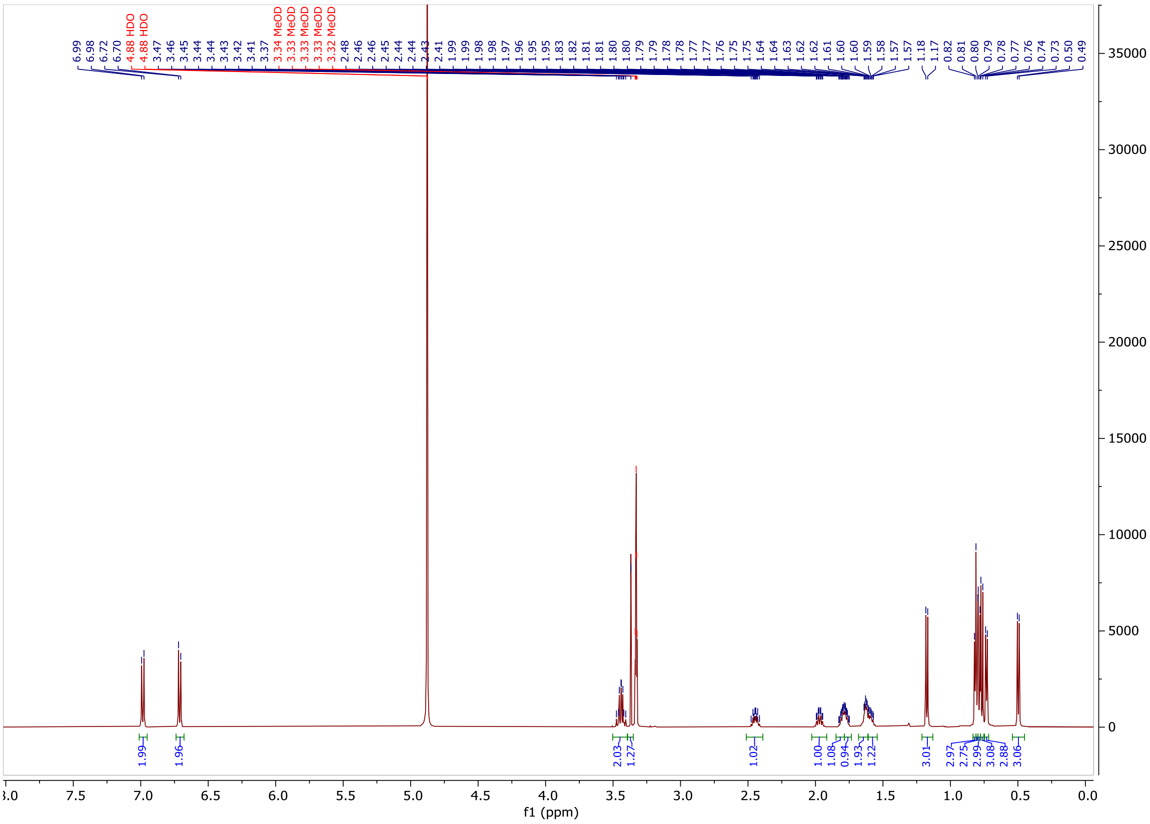

^{13}C-NMR (125 MHz, MeOD) of compound **40**

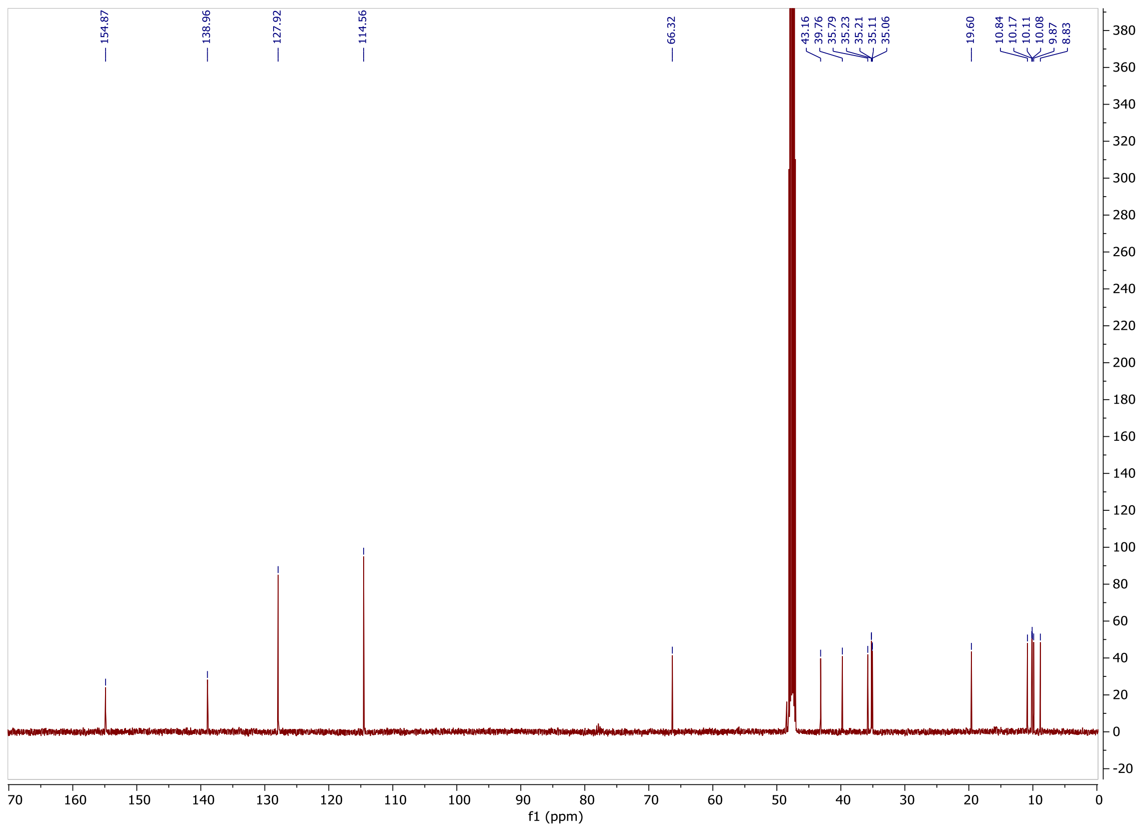

^{1}H-NMR (500 MHz, MeOD) of compound **17**

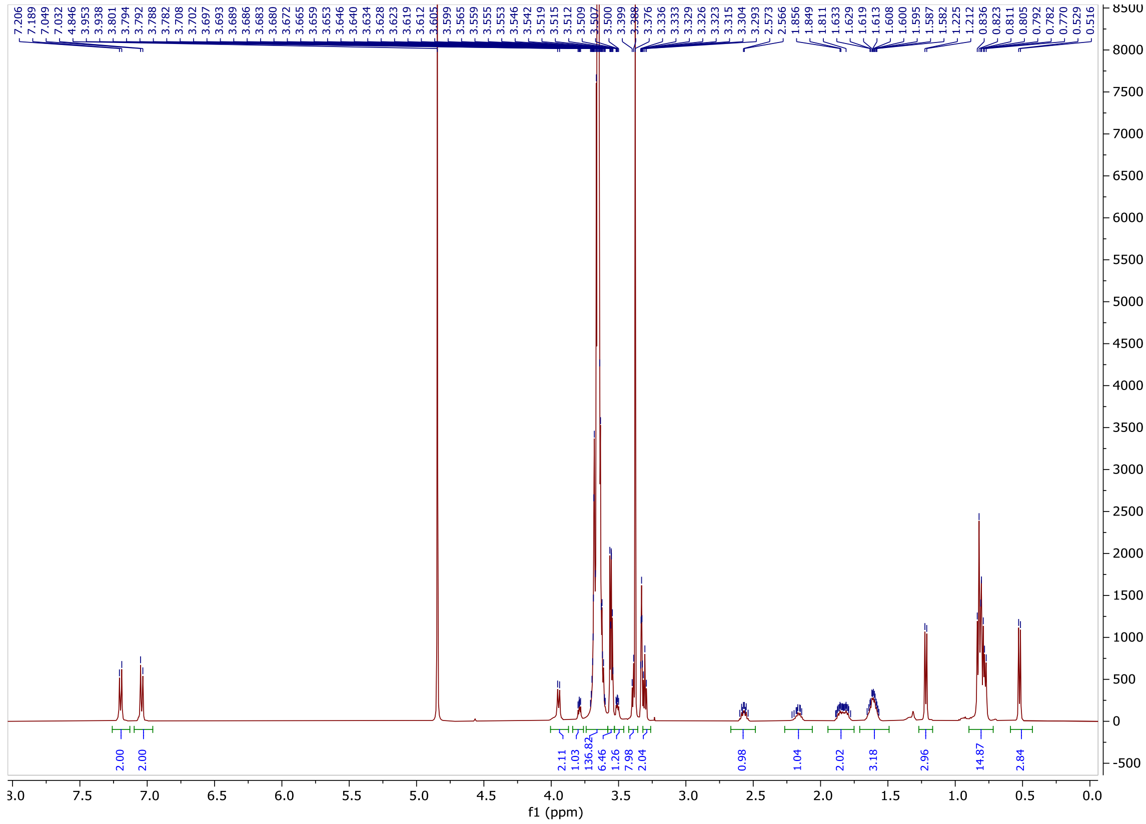

^{13}C-NMR (125 MHz, MeOD) of compound **17**

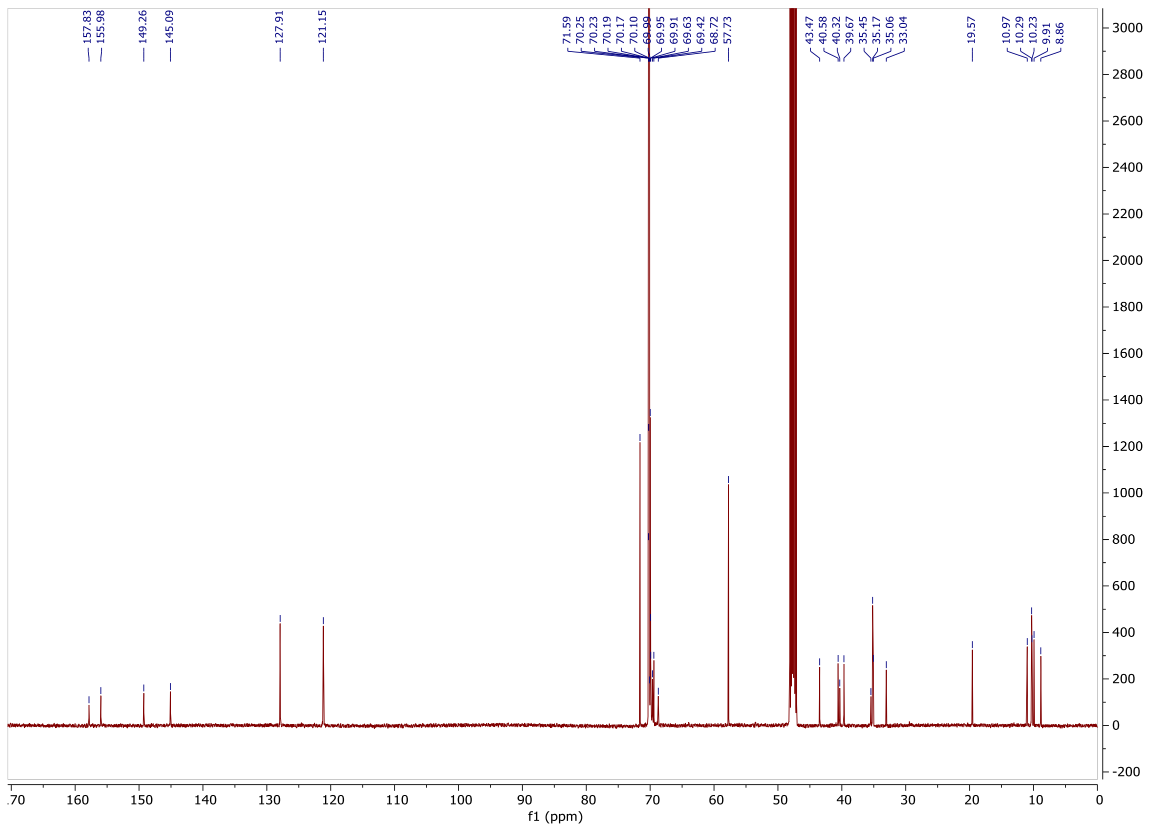

^{1}H-NMR (500 MHz, CDCl3) of compound **41**

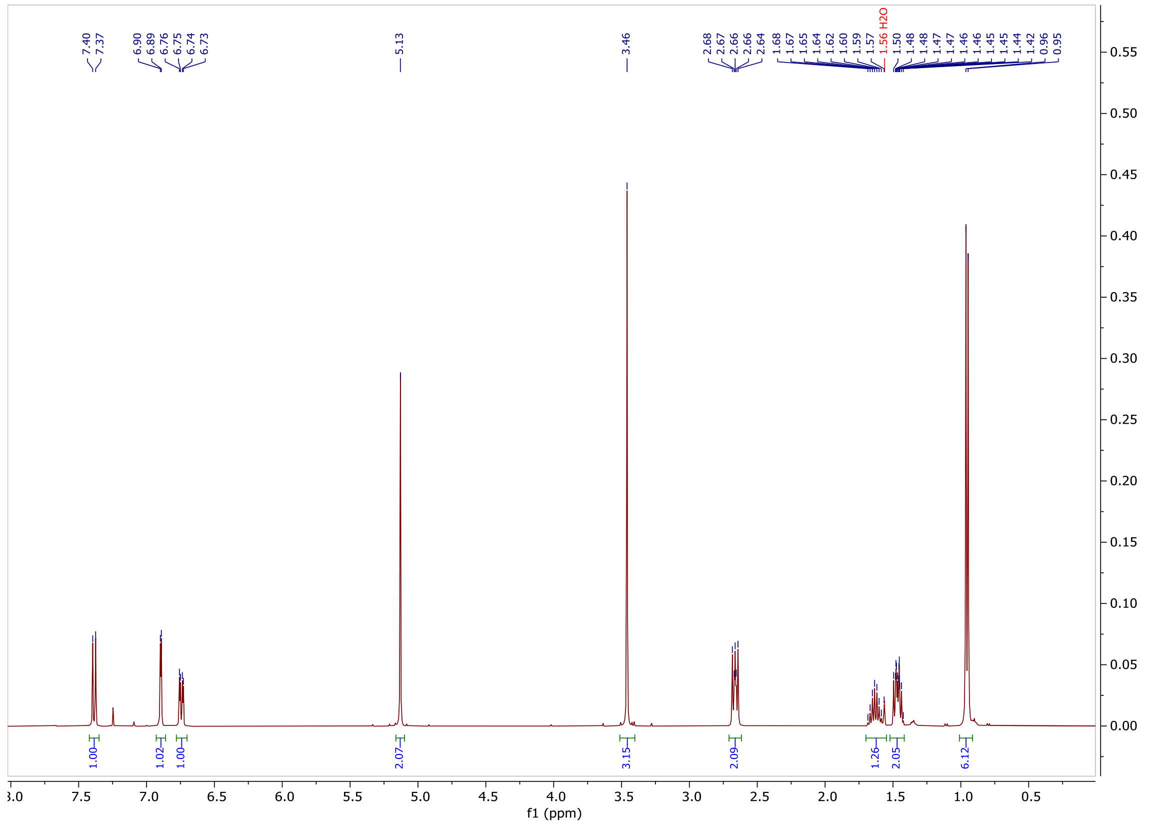

^{13}C-NMR (125 MHz, CDCl3) of compound **41**

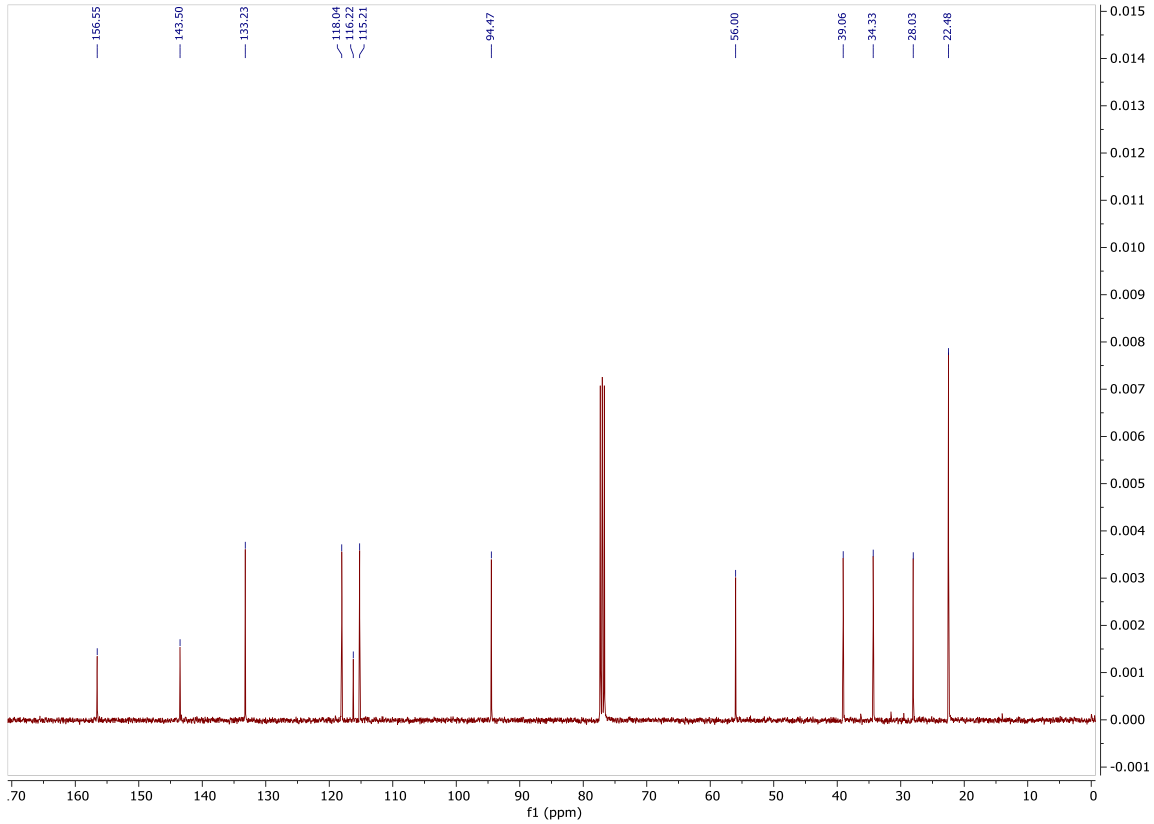

^{1}H-NMR (500 MHz, CDCl3) of compound **42**

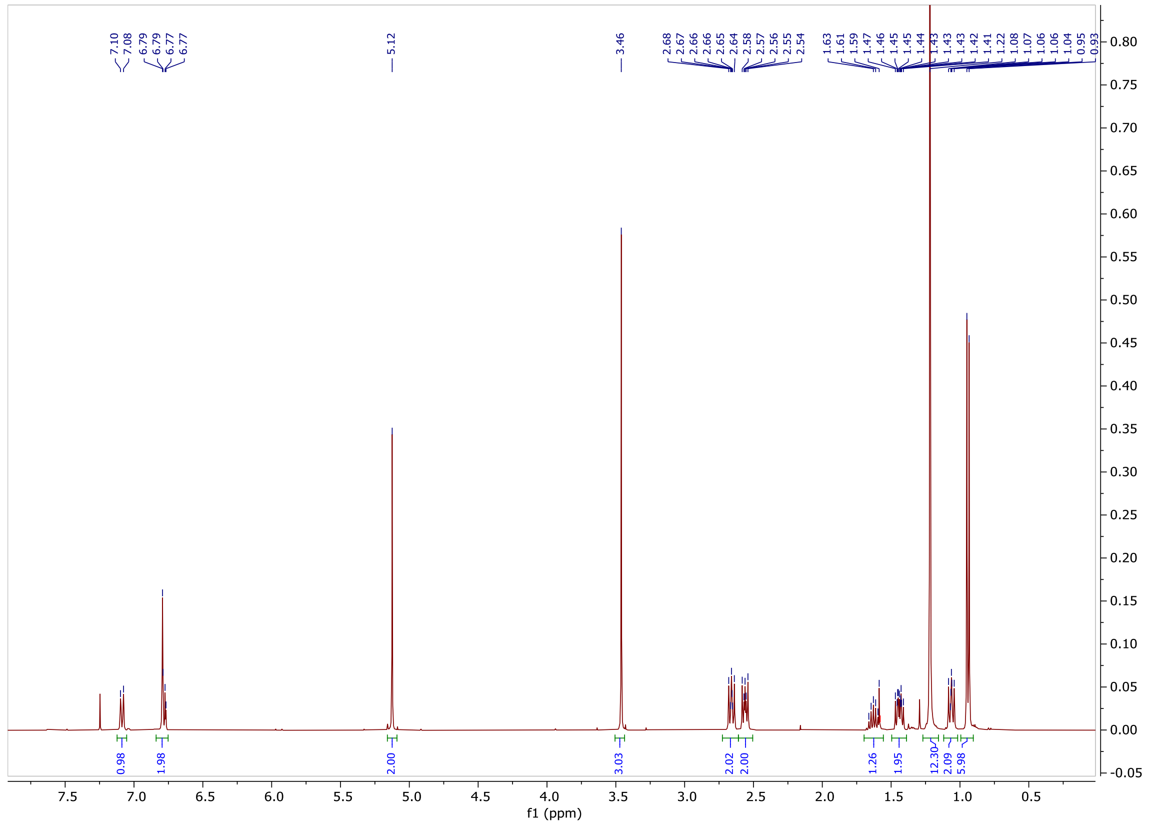

^{13}C-NMR (125 MHz, CDCl3) of compound **42**

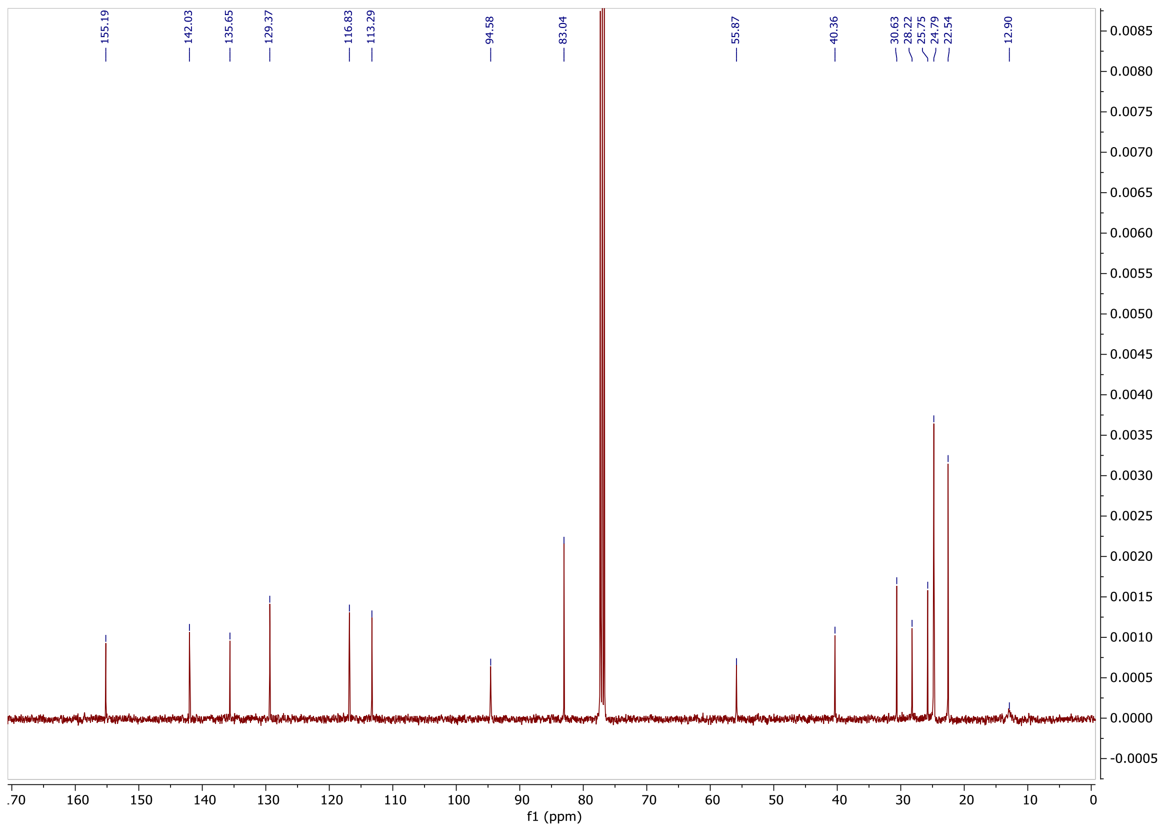

^{1}H-NMR (500 MHz, CDCl3) of compound **43**

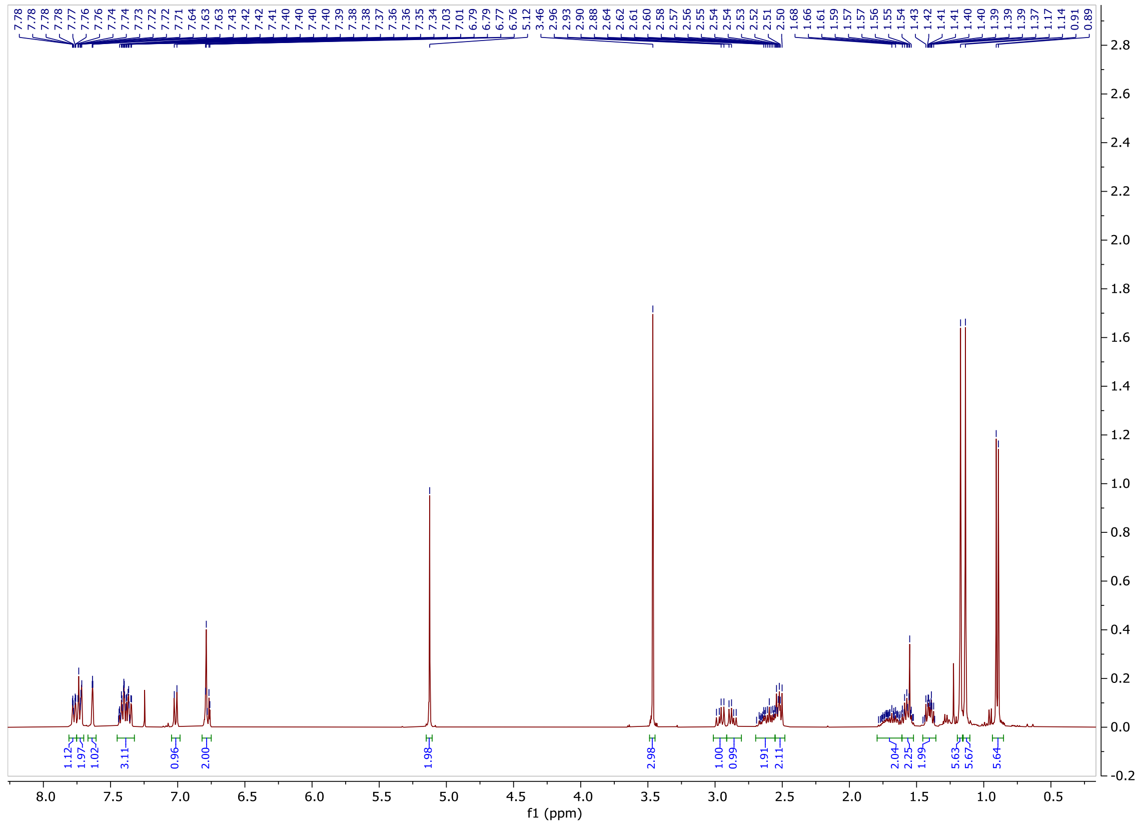

^{13}C-NMR (125 MHz, CDCl3) of compound **43**

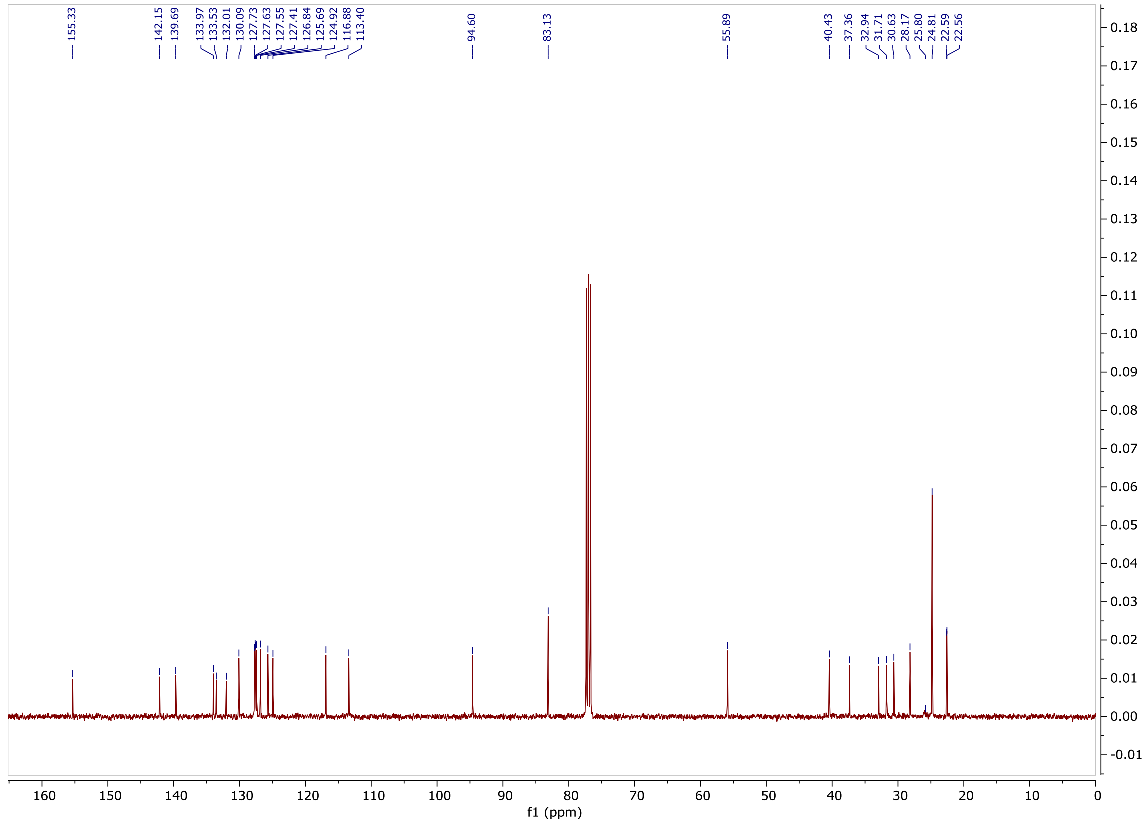

^{1}H-NMR (500 MHz, CDCl3) of compound **44**

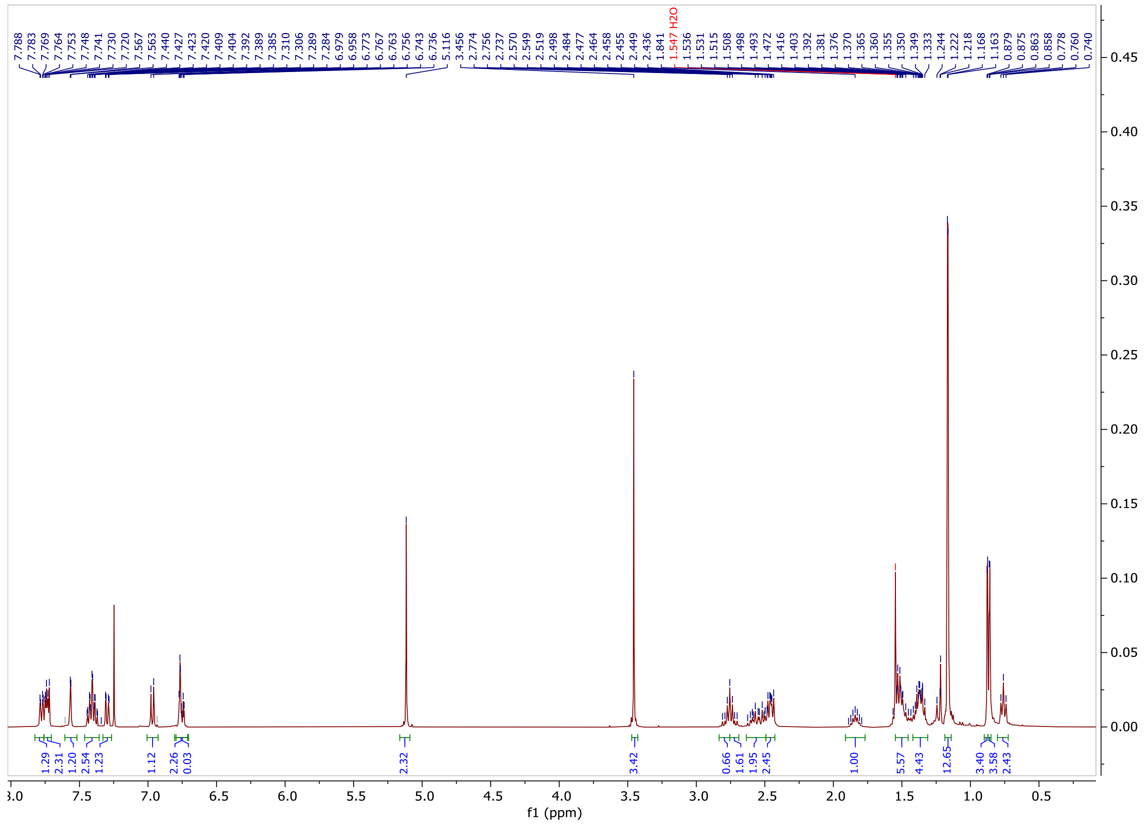

^{13}C-NMR (125 MHz, CDCl3) of compound **44**

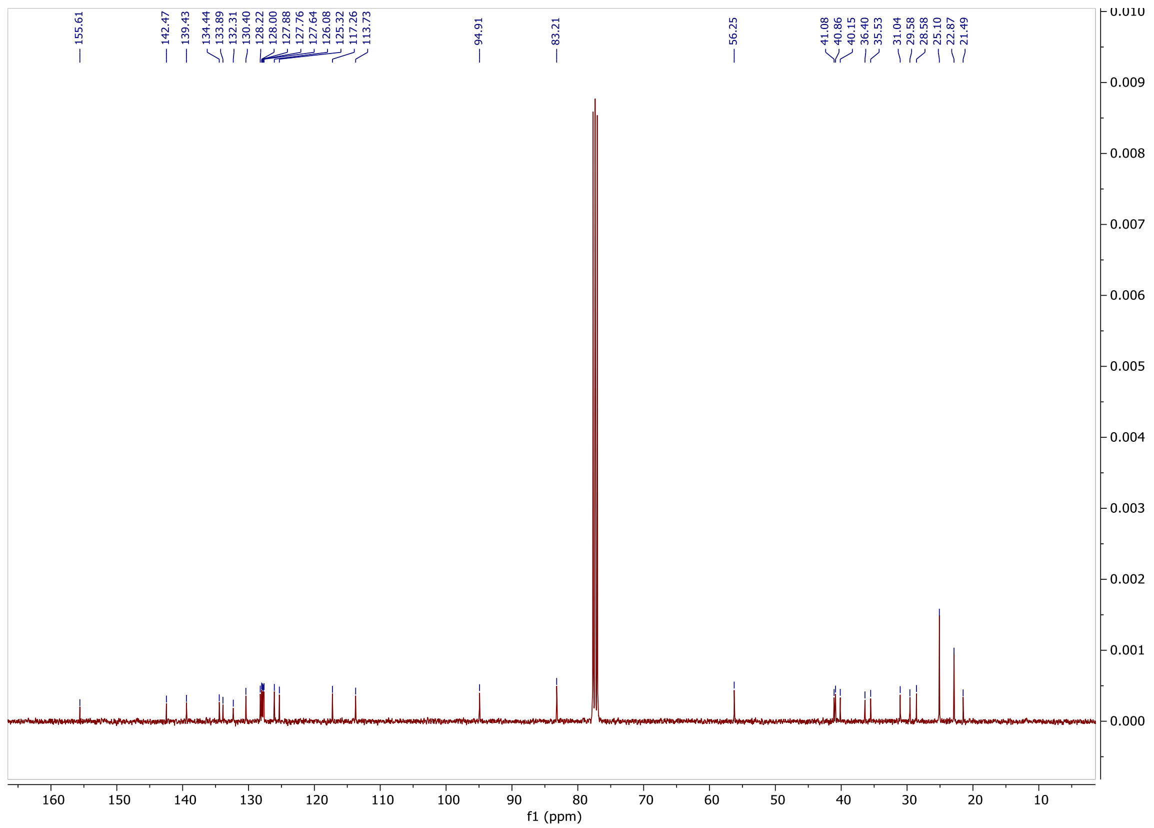

^{1}H-NMR (500 MHz, CDCl3) of compound **45**

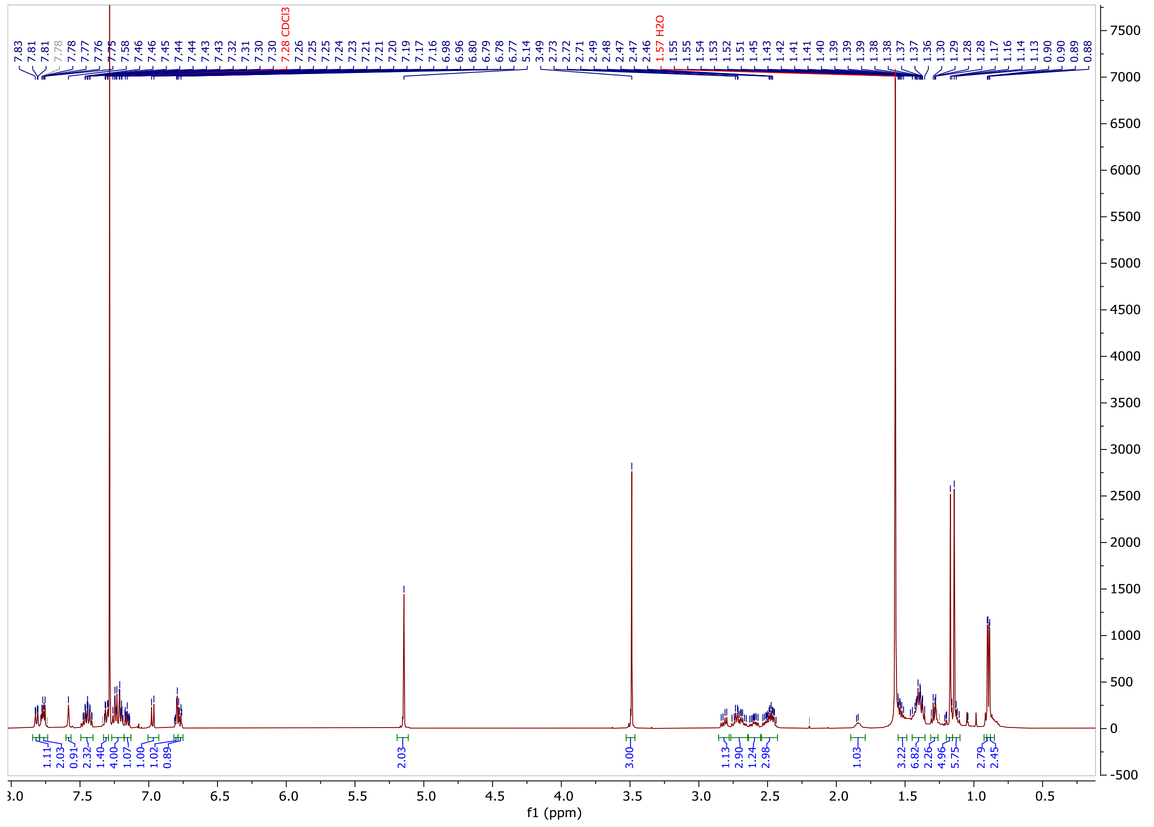

^{13}C-NMR (125 MHz, CDCl3) of compound **45**

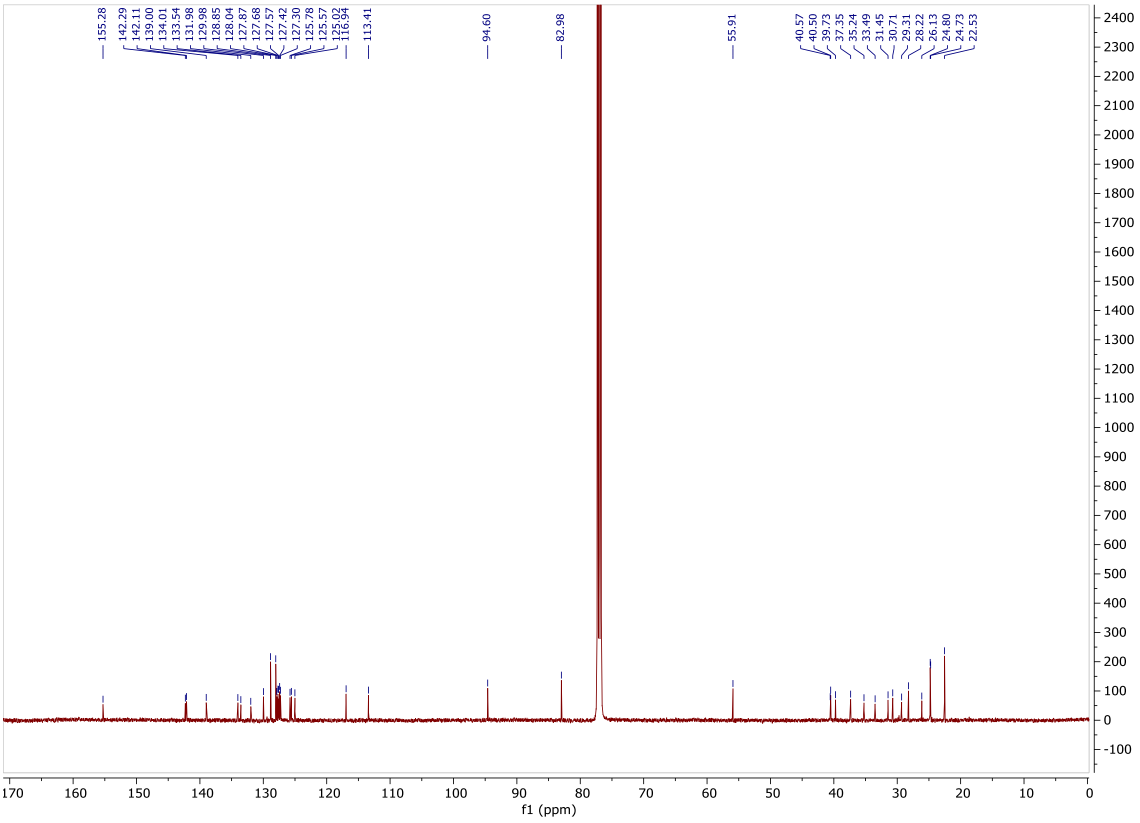

^{1}H-NMR (500 MHz, MeOD) of compound **47**

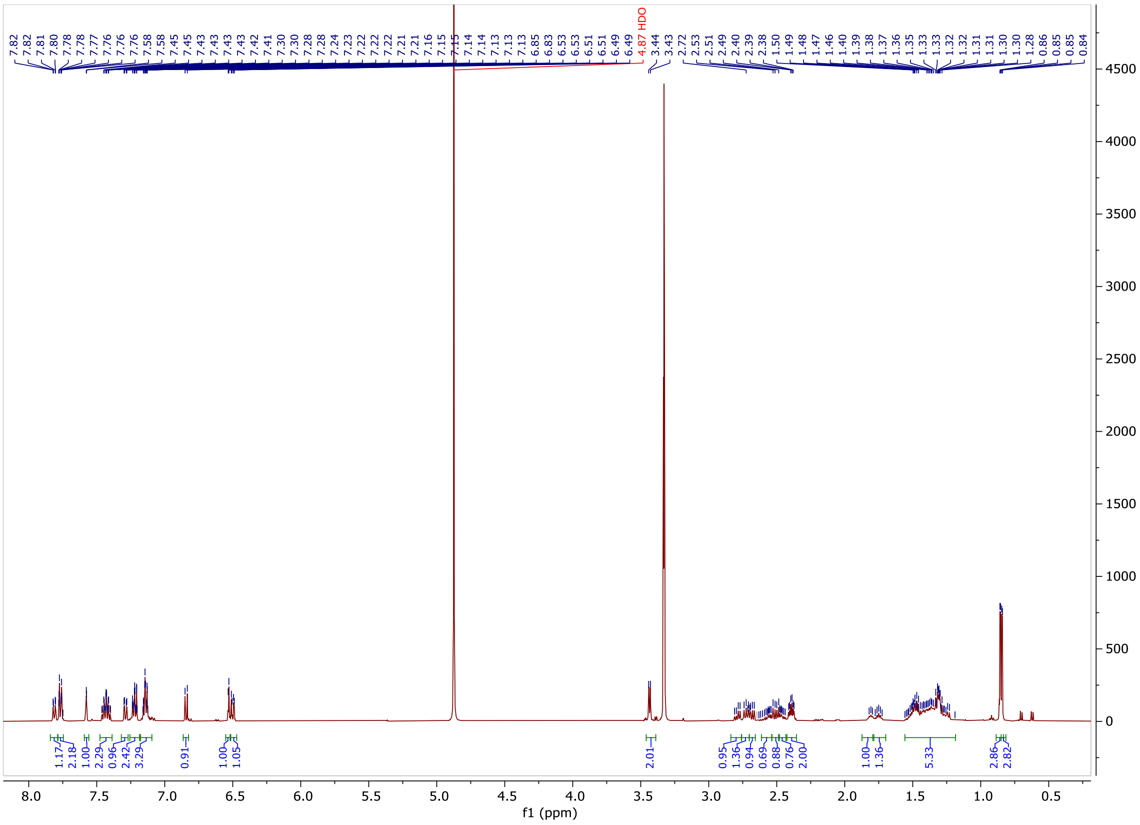

^{13}C-NMR (125 MHz, MeOD) of compound **47**

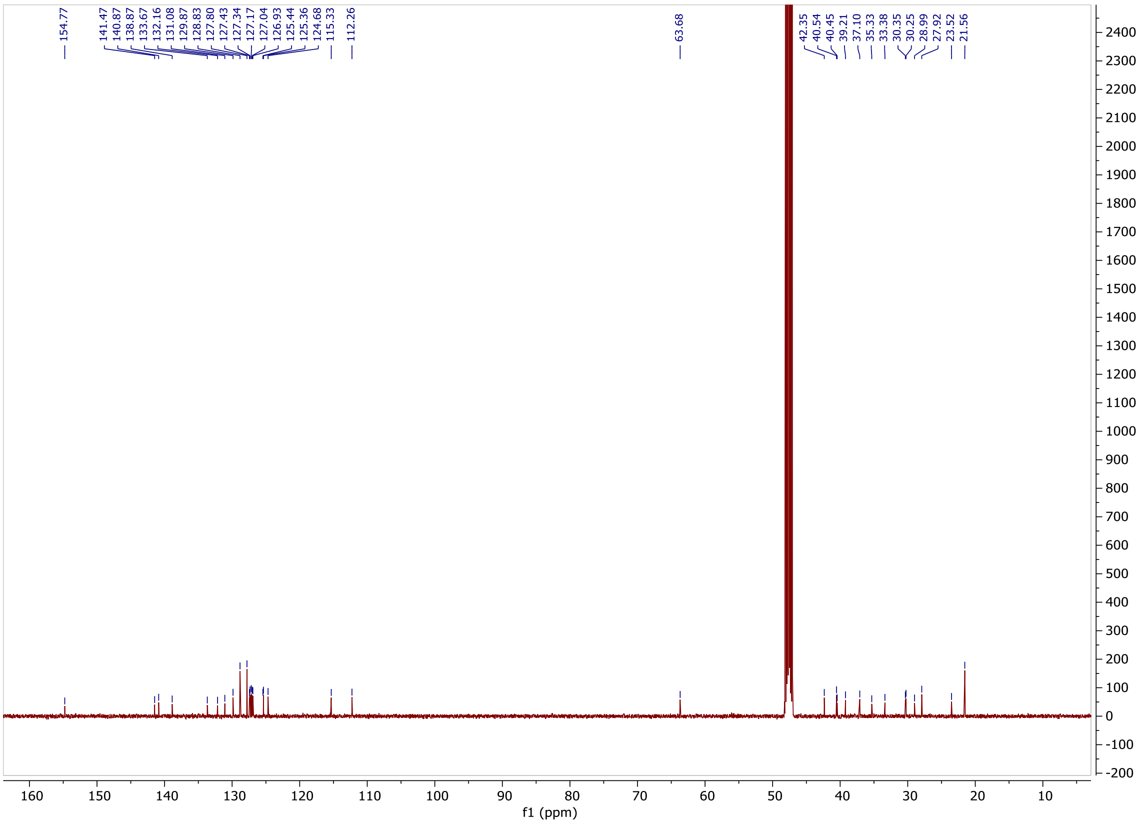

^{1}H-NMR (500 MHz, MeOD) of compound **18**

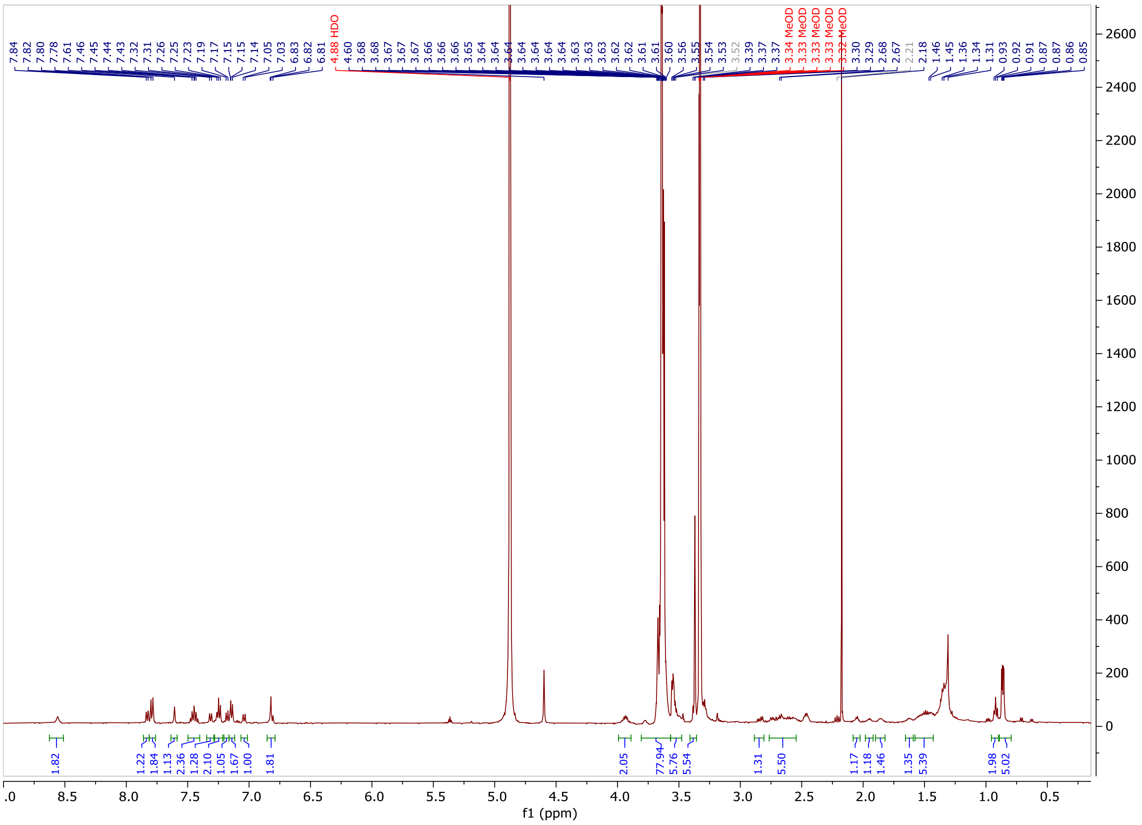

^{13}C-NMR (125 MHz, MeOD) of compound **18**

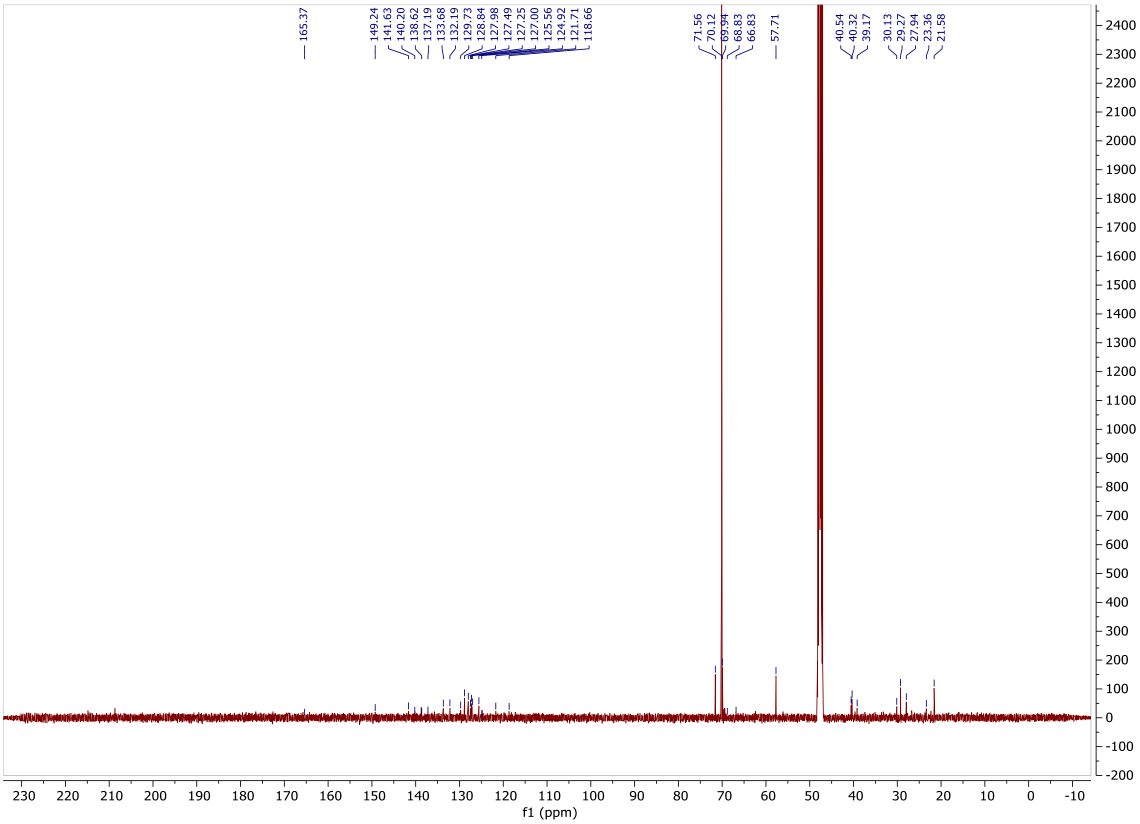

9.0 References

1. Mohamadi, F. *et al.* Macromodel—an integrated software system for modeling organic and bioorganic molecules using molecular mechanics. *J. Comput. Chem.* **11**, 440–467 (1990).

2. Maestro, Version 9.2, Schrödinger, LLC, New York, NY. (2011).

3. Chang, G., Guida, W. C. & Still, W. C. An internal-coordinate Monte Carlo method for searching conformational space. *J. Am. Chem. Soc.* **111**, 4379–4386 (1989).

4. Halgren, T. a. Merck Molecular Force Field.I. Basis, Form, Scope, Parameterization, and Performance of MMFF94*. *J. Comput. Chem.* **17**, 490–519 (1996).

5. Trott, O. & Olson, A. J. AutoDock Vina: Improving the Speed and Accuracy of Docking with a New Scoring Function, Efficient Optimization, and Multithreading. *J. Comput. Chem.* **31**, 455–461 (2010).

6. Nikolic, N. A. & Beak, P. (R)-(+)-2-(Diphenylhydroxymethyl)Pyrrolidine. *Org. Synth.* **74**, 23 (1997).

7. Burns, M. *et al.* Assembly-line synthesis of organic molecules with tailored shapes. *Nature* **513**, 183–188 (2014).

8. Kapeller, D. C. & Hammerschmidt, F. Stability , Reactions , and Mechanistic Studies. *J. Org. Chem.* **74**, 2380–2388 (2009).

9. McNeil, N. M. R. *et al.* Enhanced Glutathione Peroxidase Activity of Water-Soluble and Polyethylene Glycol-Supported Selenides, Related Spirodioxyselenuranes, and Pincer Selenuranes. *J. Org. Chem.* **81**, 7884–7897 (2016).

10. Gan, Y. & Spencer, T. A. Cholesterol surrogates incorporating a benzophenone as part of the sterol tetracycle. *J. Org. Chem.* **71**, 5870–5875 (2006).

11. M. J. Frisch, G. W. Trucks, H. B. Schlegel, G. E. S. *et al.* No Title. *Gaussian 09, Revis. D.01, Gaussian, Inc., Wallingford CT* (2013).

12. Macura, S., Farmer, B. T. & Brown, L. R. An improved method for the determination of cross-relaxation rates from NOE data. *J. Magn. Reson.* **70**, 493–499 (1986).

13. Stoll, R. *et al.* Sequence-specific H-1, N-15, and C-13 assignment of the N- terminal domain of the human oncoprotein MDM2 that binds to p53. *J. Biomol. Nmr* **17**, 91–92 (2000).

14. Riedinger, C. *et al.* Analysis of chemical shift changes reveals the binding modes of isoindolinone inhibitors of the MDM2-p53 interaction. *J. Am. Chem. Soc.* **130**, 16038–16044 (2008).
